# Limited synergy of obesity and hypertension, prevalent risk factors in onset and progression of heart failure with preserved ejection fraction

**DOI:** 10.1111/jcmm.14542

**Published:** 2019-07-31

**Authors:** Maarten M. Brandt, Isabel T. N. Nguyen, Merle M. Krebber, Jens van de Wouw, Michal Mokry, Maarten J. Cramer, Dirk J. Duncker, Marianne C. Verhaar, Jaap A. Joles, Caroline Cheng

**Affiliations:** ^1^ Experimental Cardiology, Department of Cardiology Thoraxcenter Erasmus University Medical Center Rotterdam The Netherlands; ^2^ Department of Nephrology and Hypertension University Medical Center Utrecht Utrecht The Netherlands; ^3^ Epigenomics facility University Medical Center Utrecht Utrecht The Netherlands; ^4^ Regenerative Medicine Center Utrecht University Medical Center Utrecht Utrecht The Netherlands; ^5^ Department of Cardiology University Medical Center Utrecht Utrecht The Netherlands

**Keywords:** ejection fraction, deoxycorticosterone acetate, diastolic function, heart failure, hypertension, obesity

## Abstract

Obesity and hypertension are prevalent comorbidities in heart failure with preserved ejection fraction. To clarify if and how interaction between these comorbidities contributes to development of diastolic dysfunction, lean and obese ZSF1 rats were treated with deoxycorticosterone acetate implants and a high‐salt diet (DS) to induce severe hypertension, or with placebo. In addition to echocardiographic, metabolic and hemodynamic analyses, immunohistochemistry and RNAseq were performed on left ventricular tissue. Obesity negatively affected cardiac output, led to an elevated E/e’ ratio and mildly reduced ejection fraction. DS‐induced hypertension did not affect cardiac output and minimally elevated E/e’ ratio. Diastolic derangements in placebo‐treated obese rats developed in absence of inflammation and fibrosis, yet in presence of oxidative stress and hypertrophic remodelling. In contrast, hypertension triggered apoptosis, inflammation and fibrosis, with limited synergy of the comorbidities observed for inflammation and fibrosis. Transcriptional data suggested that these comorbidities exerted opposite effects on mitochondrial function. In placebo‐treated obese rats, genes involved in fatty acid metabolism were up‐regulated, whereas DS‐induced a down‐regulation of genes involved in oxidative phosphorylation. Overall, limited interaction was observed between these comorbidities in development of diastolic dysfunction. Importantly, differences in obesity‐ and hypertension‐induced cardiac remodelling emphasize the necessity for comorbidity‐specific phenotypical characterization.

## 1 INTRODUCTION

The chronic and progressive condition, in which the heart is unable to maintain cardiac output commensurate with the body's requirements, is referred to as heart failure (HF). Based on ejection fraction, HF can be divided into HF with reduced ejection fraction (HFrEF), marked by the inability to adequately contract during systole, and HF with preserved ejection fraction (HFpEF). HFpEF is associated with impaired cardiac relaxation and increased passive cardiac stiffness, resulting in elevated end‐diastolic pressure and impaired left ventricular (LV) filling.^1^ HFpEF accounts for over 50% of all HF cases in Europe and the United States of America, yet currently no effective treatment is available for this specific form of HF.^1^

This lack of evidence‐based treatment options might find its origin in the complex, multifactorial disease aetiology of HFpEF. Development and progression of HFpEF is associated with a high prevalence of non‐cardiac comorbidities and in previous studies it was proposed that these comorbidities could provoke a state of chronic systemic inflammation, leading to coronary microvascular dysfunction, oxidative stress, enhanced cardiomyocyte stiffness and myocardial fibrosis.^2^ Obesity and hypertension are both highly prevalent (84% and 60%‐80%, respectively) among HFpEF patients.^3^, ^4^ It has been demonstrated that both comorbidities can individually contribute to development of diastolic dysfunction in rodent models. For example, deoxycorticosterone acetate‐ and salt‐induced hypertensive rats developed perivascular fibrosis, and concentric cardiac hypertrophy accompanied by diastolic dysfunction in addition to hypertension.^5^, ^6^ Similarly, in leptin‐deficient murine obesity models, ob/ob mice were shown to develop concentric cardiac remodelling with increased cardiomyocyte size and diastolic dysfunction,^7^, ^8^ secondary to obesity and type 2 diabetes.^9^ Importantly, it is estimated that at least 75% of the incidence of hypertension in the human population is related to obesity,^10^ and both comorbidities thus often present together. The current consensus is that metabolic and hypertensive disease, like other HFpEF‐associated comorbidities, contribute to the systemic state of inflammation,^2^ and as such, potentially act synergistically in development and progression of diastolic dysfunction. However, direct evidence for such a synergistic relation is lacking. More insight in the putative synergistic effects might not only provide a better understanding of the pathogenesis of HFpEF itself, but could also lead to novel insights in the poor performance of antihypertensive drug treatment alone in reducing morbidity or mortality in subsets of (obese) patients with HFpEF.^11^

Here, we tested the hypothesis that metabolic and hypertensive disease act synergistically in development and/or progression of HFpEF. For this purpose, male lean and obese ZSF1 rats, of which the latter were previously shown to develop diastolic dysfunction between week 10 and 20 of natural ageing,^12^, ^13^ were studied from 12 weeks of age. Both lean and obese rats were treated with deoxycorticosterone acetate implants plus high‐salt diet (DS) or placebo from 19 to 26 weeks of age to induce severe hypertension. DS treatment was limited to this period to prevent potential progression to HFrEF. In addition to echocardiographic, metabolic and hemodynamic analyses, immunohistochemistry and RNAseq on LV apex tissue was performed, which enabled us to dissect which pathophysiological and transcriptional adaptations were associated with obesity and its associated biochemical aberrations, and which adaptations with DS‐induced hypertension.

## 2 METHODS

### 2.1 Ethics

All animal studies were approved by the Animal Ethics Committee of the University of Utrecht (CCD: AVD115002016462) and were in accordance with the Dutch Codes of Practice for the Care and Use of Animals for Scientific Purposes. Animal experiments were performed according to ARRIVE (Animal Research: Reporting of In Vivo Experiments) guidelines.

### 2.2 Animal model

**Figure 1:**
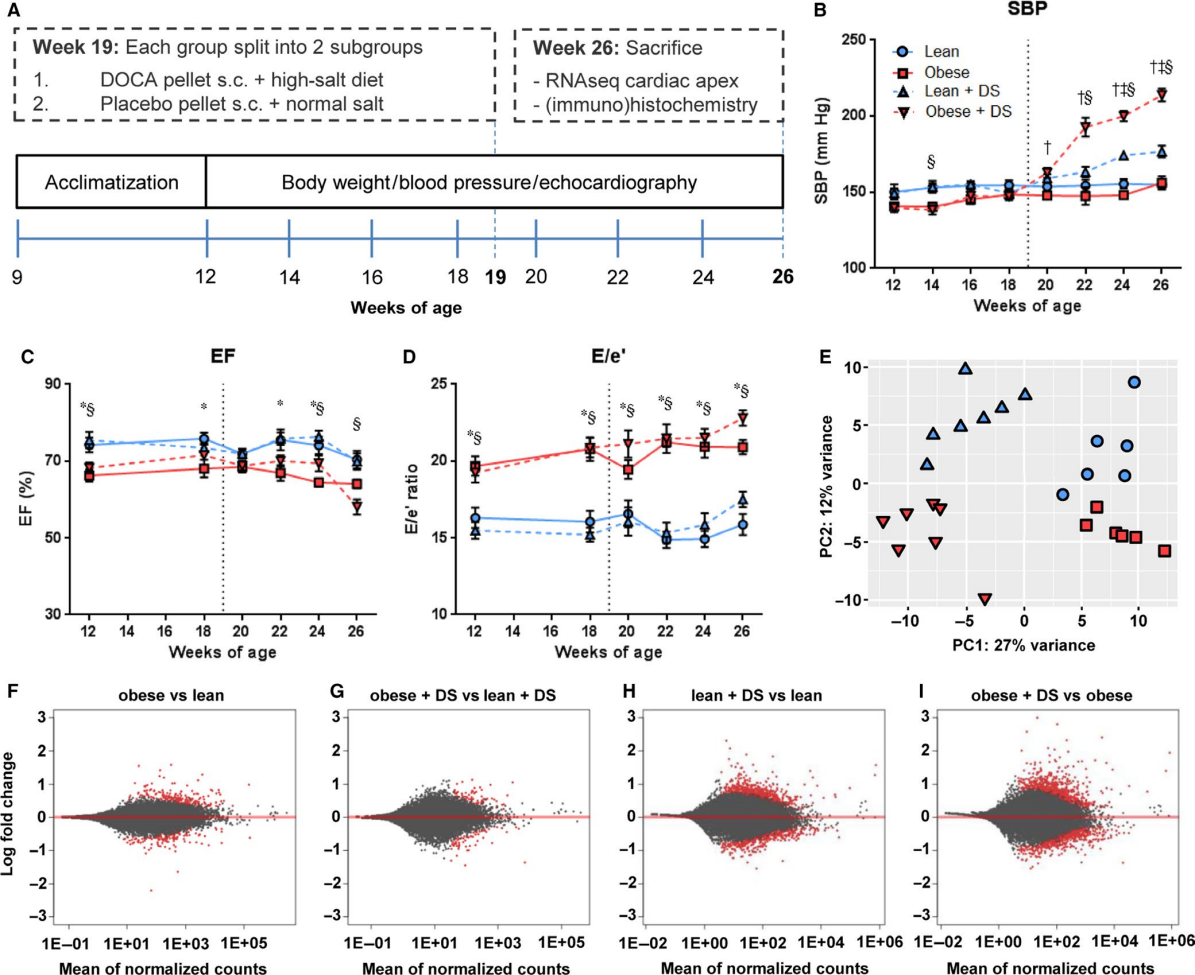
Workflow and model characteristics. (A) Graphical workflow, showing that male lean and obese ZSF1 rats were studied from 12 to 26 weeks of age. Both lean and obese rats were treated with DS or placebo from 19 to 26 weeks of age to trigger severe hypertension. Echocardiographic, metabolic and hemodynamic analyses were done every 2 weeks, till sacrifice at the age of 26 weeks. Longitudinal plots of (B) systolic blood pressure (mm Hg), (C) ejection fraction (%) and (D) E/e’ ratio. n = 6‐7, * obese vs lean, § obese + DS vs lean + DS, ‡ lean + DS vs lean, † obese + DS vs obese *P* < .05. (E) Principal component analysis of expression profiles in cardiac tissue. Symbols denoting subgroups panels B‐E are shown in panel B. (F‐I) Graphic display of differential gene expression in which log2FC is plotted against the mean of normalized counts. Red dots represent the differentially expressed genes (FDR < 0.1), grey dots represent non‐differentially expressed genes

Nine‐week‐old male lean (n = 13) and obese (n = 13) ZSF1 rats were obtained from Charles River and housed in a temperature‐ and humidity‐controlled environment with a 12‐hour light/dark cycle. Rats had access to water and standard chow (CRM‐E; Special Diet Services) ad libitum. Starting at 12 weeks until 26 weeks of age, systolic blood pressure (SBP) measurements via tail‐cuff plethysmography were obtained every 2 weeks. At 12 weeks and every 2 weeks from 18 to 26 weeks of age echocardiography was performed, whereas body weight was measured every week. At 19 weeks of age, lean and obese rats were randomized and either implanted with a deoxycorticosterone acetate pellet (35 mg in lean, 50 mg in obese; Innovative Research of America) and fed a high‐salt diet (6% NaCl) or with a placebo pellet and fed a normal salt diet. At week 26, after the last measurements, the rats were terminated via exsanguination under deep anaesthesia and organs were harvested and weighed (Figure 1A).

### 2.3 Echocardiographic evaluation

Rats were anesthetized with isoflurane (3.5%‐4% for induction and 2%‐2.5% for maintenance). Transthoracic echocardiography was performed with a digital ultrasound machine (model Sonos 5500, Philips Research) and a 15‐MHz linear array transducer (Hewlett Packard). The LV long‐axis dimensions were recorded by 2D echocardiography. Two‐dimensional B‐mode cine loops were recorded in the parasternal long‐axis and the midpapillary short‐axis views. Mitral flow velocity tracings were obtained with pulsed‐wave Doppler above the mitral leaflets. Tissue Doppler imaging (TDI) was used to obtain early (e’) diastolic velocity at the medial mitral annulus. The recordings were averaged from three consecutive heartbeats.

### 2.4 Statistics

Data are presented as mean ± SEM. Groups were compared by 2‐way ANOVA or 2‐way ANOVA for repeated measures, followed by Tukey post hoc test, as appropriate. Statistical significance was accepted when *P* < .05.

Detailed description of the methods is available in the Appendix S1.

## 3 RESULTS

### 3.1 Model characteristics

Male ZSF1 rats, heterozygous (lean) or homozygous (obese) for leptin receptor mutation, were studied from 12 to 26 weeks of age. Both lean and obese rats were treated with DS or placebo from 19 to 26 weeks of age, and echocardiographic, metabolic and hemodynamic analyses were performed every two weeks (Figure 1A). ZSF1 rats with leptin receptor deficiency had significantly more weight gain (Table 1). This weight gain was accompanied by elevated blood cholesterol and triglycerides, with no distinction between placebo‐ and DS‐treated obese rats. An increase in blood glucose levels was observed in obese rats, which was attenuated by DS treatment. As anticipated, DS treatment led to markedly increased urinary sodium excretion. This sodium excretion was significantly higher in DS‐treated obese rats compared with DS‐treated lean rats, but was not different when corrected for body weight. Systolic blood pressure was also elevated in both lean and obese DS‐treated rats, with a synergistic elevation in DS‐treated obese rats (Figure 1B).

**Table 1:** Model characteristics at 26 wk of age

	Lean	Lean + DS	Obese	Obese + DS	*P*‐values		
	(n = 6)	(n = 7)	(n = 6)	(n = 7)	Obesity	DS	Interaction
Tibia length (mm)	40.8 ± 0.5	40.0 ± 0.4	38.1 ± 0.4^a^	39.1 ± 0.6	<.05	.93	.05
Body weight (g)	443 ± 9	417 ± 10	584 ± 13^a^	607 ± 10^b^	<.05	.91	<.05
Cholesterol (mM)	2.2 ± 0.1	2.1 ± 0.1	8.2 ± 0.8^a^	9.1 ± 0.7^b^	<.05	.46	.34
Triglycerides (mM)	1.5 ± 0.2	1.1 ± 0.2	23.8 ± 4.0^a^	20.5 ± 2.5^b^	<.05	.44	.53
Glucose (mM)	9.9 ± 0.5	9.1 ± 0.5	22.5 ± 2.6^a^	14.0 ± 1.6^d^	<.05	<.05	<.05
Natriuresis (μmol/d)	1484 ± 191	10502 ± 1126^c^	3260 ± 422	14749 ± 1463^b^^,^ d	<.05	<.05	.213
Natriuresis (μmol/d/100 g)	338 ± 46	2546 ± 300^c^	544 ± 71	2419 ± 213^d^	.83	<.05	.378
SBP (mm Hg)	153 ± 3	179 ± 4^c^	150 ± 1	213 ± 5^b^^,^ d	<.05	<.05	<.05
Heart rate (bpm)	381 ± 9	362 ± 8	308 ± 9^a^	292 ± 6^b^	<.05	<.05	.81
SV (μL)	212 ± 19	217 ± 23	178 ± 19	187 ± 19	.13	.71	.90
CO (mL/min)	81 ± 7	79 ± 8	55 ± 6	55 ± 5	<.05	.87	.92
CI (mL/min/100 g)	18.2 ± 1.6	18.7 ± 1.7	9.5 ± 1.2^a^	9.0 ± 0.9^b^	<.05	.99	.73
EF (%)	73 ± 2	70 ± 3	65 ± 1^a^	59 ± 2^b^^,^ d	<.05	<.05	.48
E/e’	15.9 ± 0.9	17.5 ± 0.6	20.5 ± 0.6^a^	22.9 ± 0.7^b^^,^ d	<.05	<.05	.55
LVW (mg)	909 ± 27	1139 ± 64	1172 ± 66	1490 ± 116^b^^,^ d	<.05	<.05	.58
HW (LV + RV; mg)	1338 ± 32	1459 ± 48	1474 ± 41	1684 ± 50^b^^,^ d	<.05	<.05	.32

Note

Values are mean ± SEM, followed by main effect‐ (effect obesity/ effect DS) and interaction p‐values, as determined by 2‐way ANOVA.

Abbreviations: 100g, 100g body weight; CI, cardiac index; CO, cardiac output; d, day; HW, heart weight; LVW, left ventricle weight; RV, right ventricle; SBP, systolic blood pressure; SV, stroke volume; TL, tibia length.

a Obese vs lean,

b Obese + DS vs lean + DS,

c Lean + DS vs lean,

d Obese + DS vs obese *P* < .05.

Obesity, in both placebo‐ and DS‐treated rats, caused a minor reduction in ejection fraction (EF), with the lowest EF (59%) observed in the DS‐treated obese group (Figure 1C). Heart rate was reduced in both placebo‐ and DS‐treated obese rats, and cardiac output (CO) tended to be lower in obese vs lean (*P* = .08) and in obese + DS vs lean + DS (*P* = .07). When corrected for body mass (cardiac index), placebo‐ and DS‐treated obese rats appeared unable to match cardiac output to body mass. Obesity and DS‐induced hypertension both tended to increase LV weight (LVW) and total heart weight (HW), but a significant increase was only observed in presence of both comorbidities. Illustrative for diastolic dysfunction, the ratio between peak velocity of early mitral inflow and early diastolic mitral annulus velocity (E/e′) was increased in obese rats (Figure 1D). Surprisingly, both for the reduced cardiac index and the increased E/e’ ratios, no interaction among the comorbidities was observed.

In contrast to the functional findings, which were principally affected by obesity, RNAseq revealed that DS‐induced hypertension caused the most predominant transcriptional variance (Figure 1E). In cardiac tissue of placebo‐treated obese vs placebo‐treated lean rats and DS‐treated obese vs DS‐treated lean rats, a total of 410 and 146 genes were differentially expressed (FDR < 0.1), respectively (Figure 1F‐G, Table S2‐S3). Conversely, in DS‐treated lean rats vs placebo‐treated lean rats and DS‐treated obese rats vs placebo‐treated obese rats, a total of 1258 and 1504 genes were differentially expressed (FDR < 0.1), respectively (Figure 1H‐I, Table S4‐S5).

### 3.2 Mitochondrial gene expression and apoptosis

**Figure 2:**
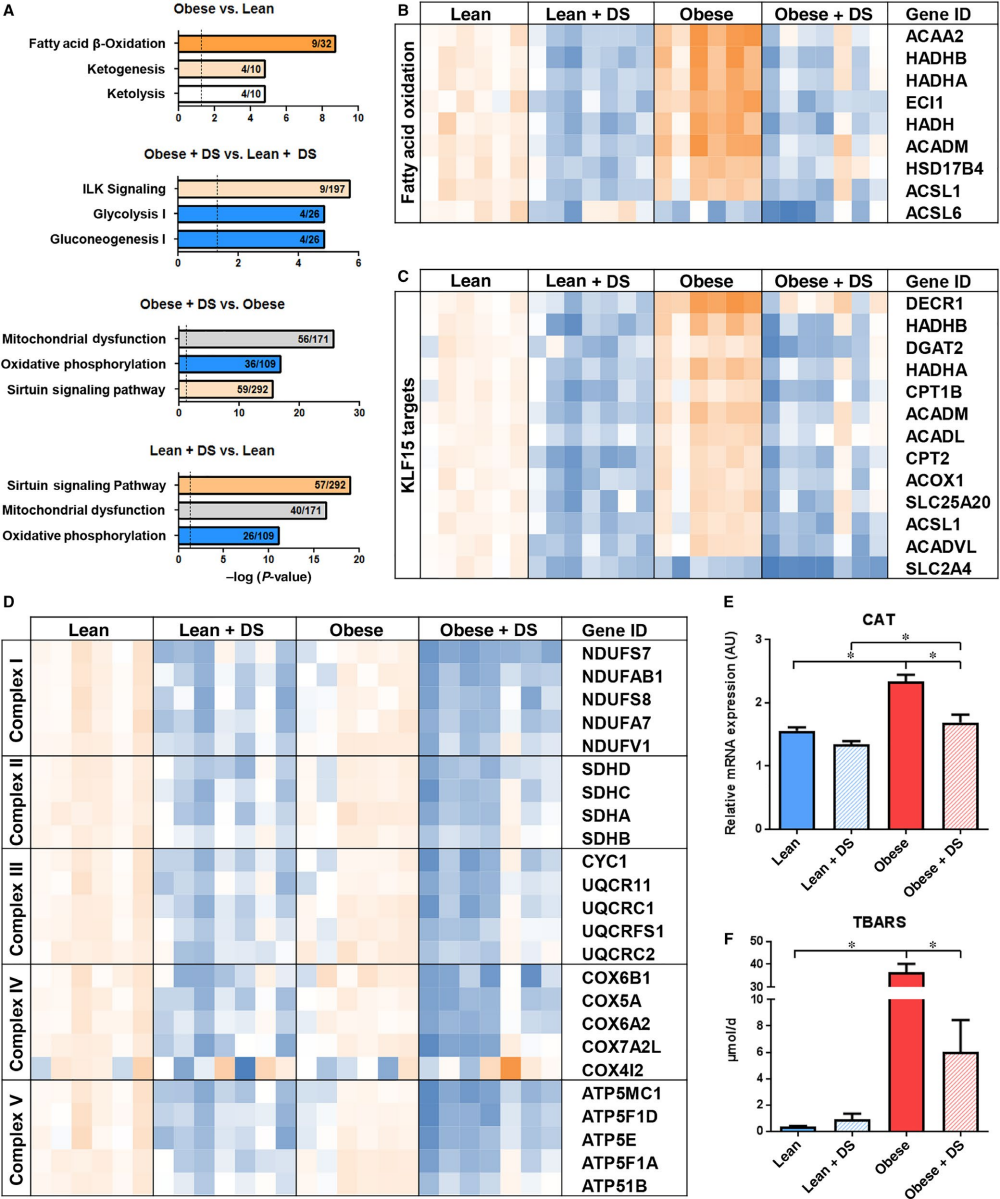
Pathway analysis and mitochondrial gene expression (A) Graphical visualization of 3 most affected pathways according to Ingenuity Pathway Analysis (orange and blue represent pathway activation and repression, respectively, grey represents no direction available). (B) Schematic presentation of RNAseq data for differentially expressed genes involved in fatty acid oxidation, (C) KLF15‐induced transcription (D) and oxidative phosphorylation. Shown is a colour‐based representation of the reads per kilobase million, relative to the average expression in the lean group, for each individual rat (orange and blue represent increased and decreased expression, relatively). (E) QPCR analysis of oxidative stress marker CAT. n = 5‐7, * *P* < .05. (F) Analysis of urinary TBARS excretion as marker of systemic oxidative stress. n = 6‐7, * *P* < .05

Analyses of transcription profiles with Ingenuity Pathway Analysis (IPA) indicated that both risk factors had substantial, yet opposite effects on genes involved in mitochondrial function. In placebo‐treated rats, enzymes involved in fatty acid metabolism and oxidative phosphorylation were up‐regulated in the obese vs lean group (Figure 2A‐B). In contrast, both lean and obese DS‐treated rats had an overall down‐regulation vs placebo‐treated rats of genes involved in oxidative phosphorylation, a phenomenon often observed in mitochondrial dysfunction (Figure 2A and 2D).^14^, ^15^

IPA predicted that the transcriptional regulator Krüppel‐like factor 15 (KLF15), which has been described as a key regulator inducing mitochondrial fatty acid substrate usage,^16^ was the most active upstream regulator in placebo‐treated obese vs lean rats (overlap *P* = 2.66E‐13/Z‐score = 2.95). Interestingly, it was among the most suppressed regulators in DS‐treated rats (overlap *P* = 5.37E‐14/Z‐score=−3.97 and overlap *P* = 7.73E‐17/Z‐score=−3.70 in lean + DS vs lean and obese + DS vs obese, respectively; Figure 2C, Table S6‐S8). qPCR analysis of established KLF15 targets Hydroxyacyl‐CoA Dehydrogenase Trifunctional Multienzyme Complex Subunit Beta (HADHB), Acyl‐CoA Dehydrogenase Medium Chain (ACADM) and 2,4 Dienoyl‐CoA reductase (DECR1), substantiated this observation (Figure S1).

RNAseq data also widely supported the DS‐induced down‐regulation of genes coding for enzymes in oxidative phosphorylation, equally affecting the different complexes of the electron transport chain (Figure 2D). Remarkably, DS‐induced down‐regulation of genes involved in oxidative phosphorylation was not accompanied by elevated transcription of oxidative stress‐responsive factor Catalase (CAT), whereas the increased transcription of fatty acid oxidation (FAO) genes in placebo‐treated obese rats on the other hand was associated with elevated CAT expression (Figure 2E). Systemic oxidative stress, as measured by urinary excretion of thiobarbituric acid reactive substances (TBARS), followed a similar pattern, showing an elevation in placebo‐treated obese rats (Figure 2F).

The adaptations in transcription of mitochondrial genes raised the question whether this led to programmed cell death. Histological analysis of activated Caspase 3, a marker of early apoptosis, indicated no significant changes in sub‐endocardial apoptosis (Figure S2A and C), although the apoptotic effect of DS‐induced hypertension showed a trend (*P* = .05; Table S9). A similar pattern was observed after labelling cells for late apoptosis, using terminal deoxynucleotidyl transferase dUTP nick end labelling (TUNEL). Individually no differences were observed, although overall DS‐induced hypertension stimulated apoptosis (Figure S2B and D, Table S9). Noteworthy, the apoptotic cells were predominantly found in patchy regions (Figure S2E).

### 3.3 Cardiomyocyte hypertrophy and foetal gene expression

**Figure 3:**
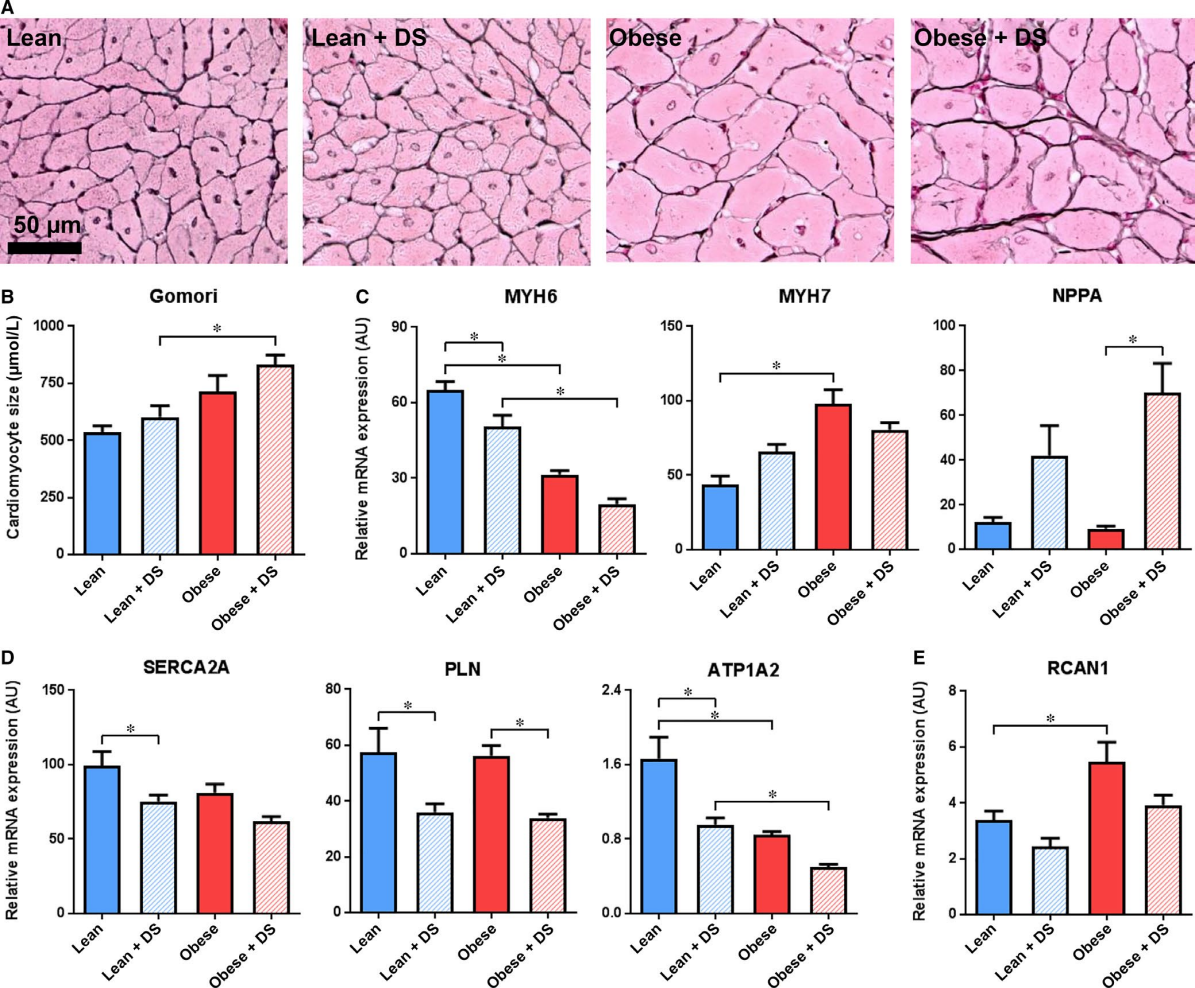
Cardiomyocyte hypertrophy and foetal gene expression (A) Typical examples and (B) quantification of histological staining for cardiac hypertrophy (Gomori). n = 4‐6, * *P* < .05. C, QPCR analysis of cardiac transcription of hypertrophy markers MYH6, MYH7 and NPPA, (D) regulators of cytoplasmic Ca^2+^ levels SERCA2A, PLN and ATP1A2 and (E) transcriptional target of Ca^2+^‐induced NFAT activation RCAN1. n = 5‐7, * *P* < .05

To get a better insight in myocardial hypertrophy in response to the comorbidities, the cross‐sectional area of individual cardiomyocytes was studied. Presence of both comorbidities led to significant cardiomyocyte hypertrophy (Figure 3A‐B). In contrast to the HW and LVW, which were affected by both DS‐induced hypertension and obesity individually, obesity was associated with an elevated cellular cross‐sectional area, but no contribution of DS‐induced hypertension was observed (Table S9).

Transcription of myosin heavy chain α (MYH6) and β (MYH7), defined markers for myocardial hypertrophy,^17^ resembled this predominant obesity‐induced effect on cardiac remodelling. Obesity triggered a down‐regulation of MYH6 and an up‐regulation MYH7 (Figure 3C). DS‐induced hypertension also reduced the expression of MYH6, though the effect size was more than two fold lower than that of obesity and appeared additive, not synergistic (Table S9). Atrial natriuretic peptide (NPPA) on the other hand, like MYH6 and MYH7 a well‐described hypertrophy marker, was up‐regulated solely in DS‐treated rats.

Transcriptional analysis demonstrated that expression of sarco/endoplasmic reticulum Ca^2+^‐ATPase (SERCA2A) and phospholamban (PLN), both involved in calcium reuptake by the sarcoplasmic reticulum during diastole, was affected as well. SERCA2A expression was lowered by both comorbidities, whereas PLN appeared to be principally affected by DS‐induced hypertension (Figure 3D). In line with SERCA2A, transcription of ATPase, Na^+^/K^+^ transporting alpha 2 (ATP1A2), was significantly lower in response to obesity and DS treatment. Of note, both for SERCA2A and ATP1A2, there was no interaction of the comorbidities (Figure 3D, Table S9). Remarkably, regulator of calcineurin 1 (RCAN1), which is a direct Ca^2+^‐dependent transcriptional target of nuclear factor of activated T cells (NFAT), was solely up‐regulated in placebo‐treated obese rats, whereas the opposite response was induced by DS (Figure 3E).

### 3.4 Capillary density

**Figure 4:**
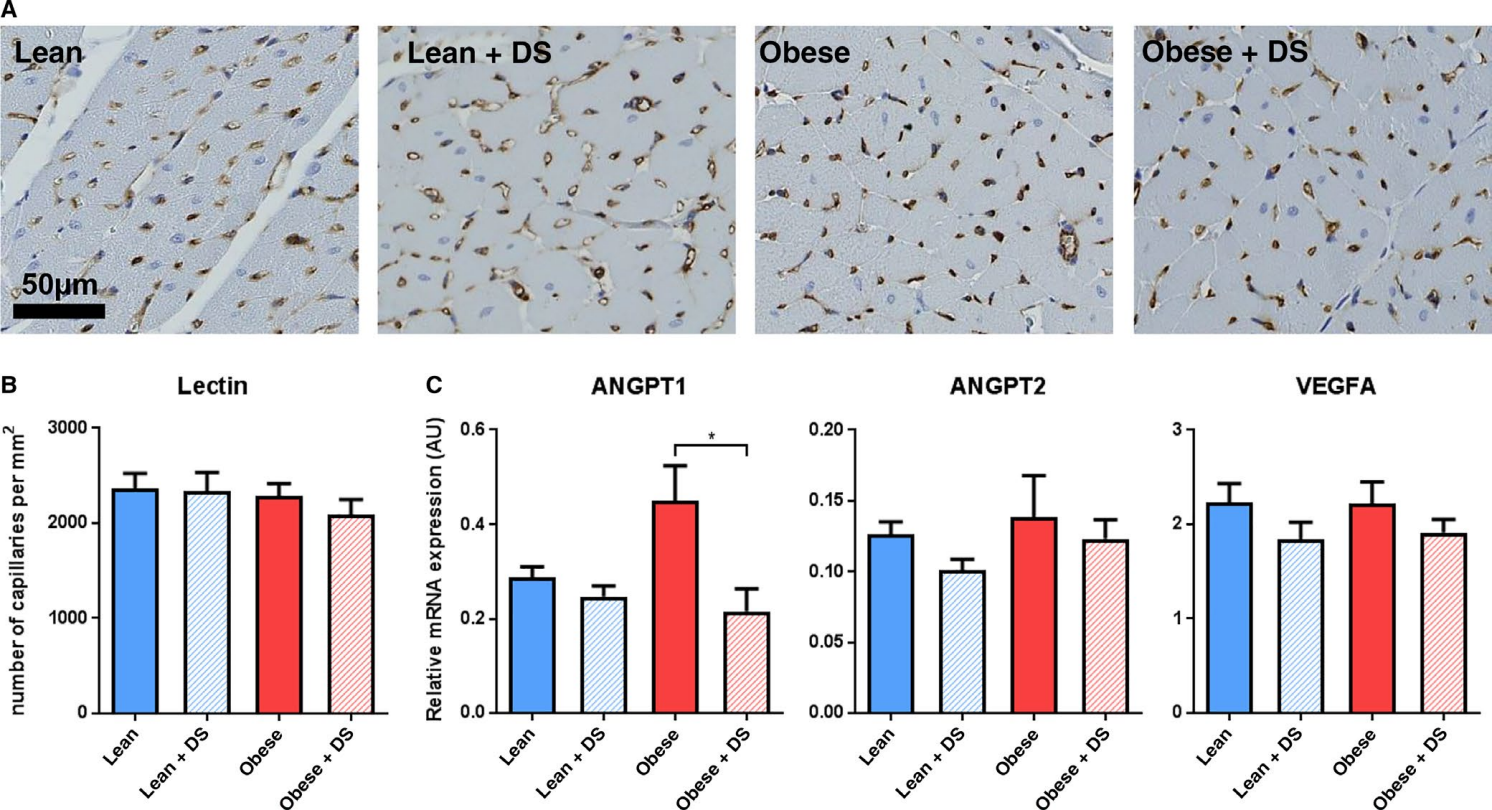
Capillary density. (A) Typical examples and (B) quantification of LV microvasculature (Lectin). n = 4‐6, * *P* < .05. (C) QPCR analysis of cardiac transcription of factors involved in vascular remodelling ANGPT1, ANGPT2 and VEGFA. n = 5‐7, * *P* < .05

To evaluate whether DS‐induced hypertension and obesity affect capillary density, a lectin staining was applied on LV cross sections. Surprisingly, no differences were observed in any of the conditions (Figure 4A‐B). The absence of microvascular adaptations was substantiated by transcription levels of well‐known modulators of microvascular stability Angiopoietin 1 and 2 (ANGPT1 and ANGPT2, respectively), and hypoxia‐associated factor Vascular Endothelial Growth Factor A (VEGFA). Transcription of vascular destabilizing factors ANGPT2 and VEGFA was not affected by the comorbidities (Figure 4C). Interestingly, transcription of ANGPT1, which promotes vascular stability, was increased in placebo‐treated obese rats, but not in DS‐treated obese rats vs their respective lean counterparts.

### 3.5 Inflammatory cells

**Figure 5:**
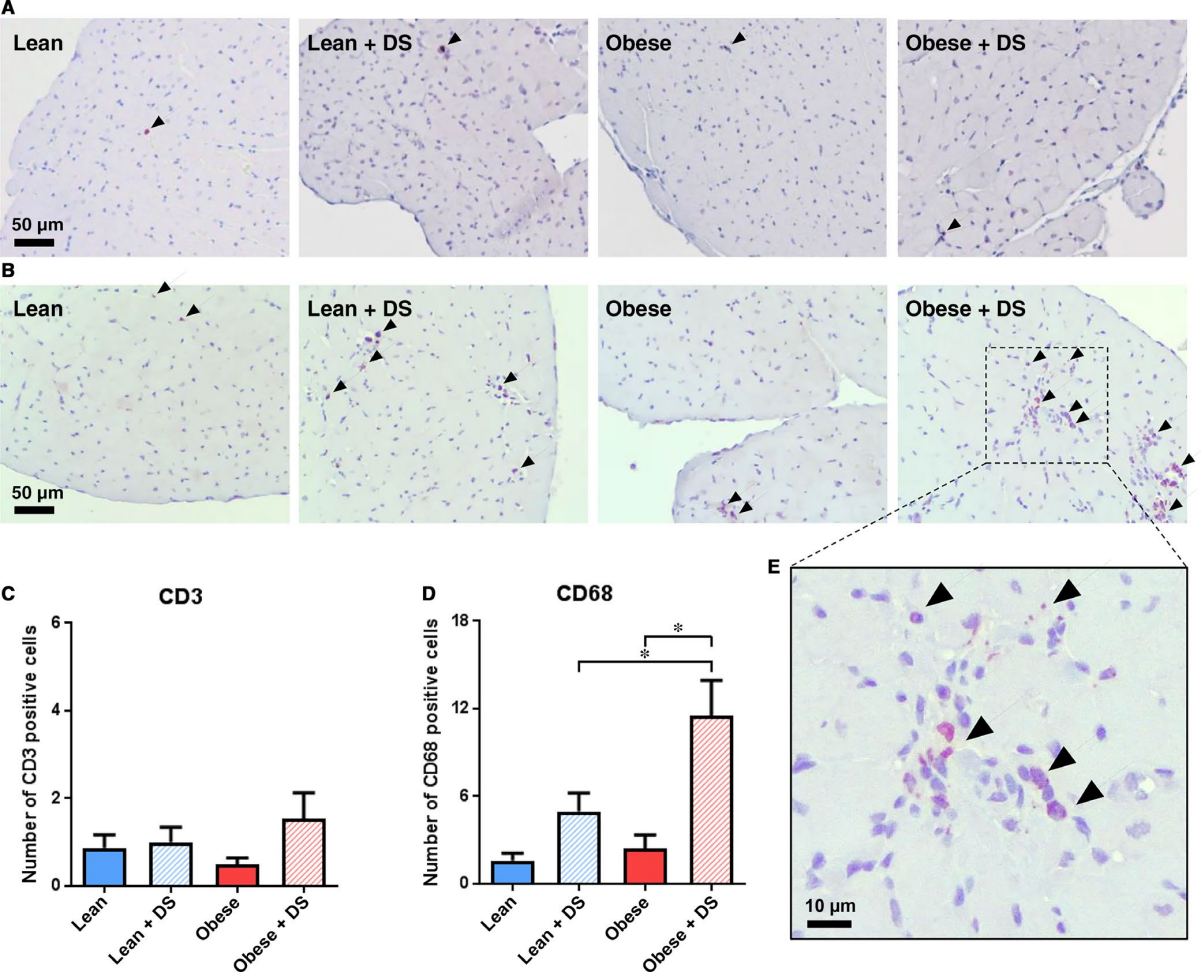
Inflammatory cells. (A) Typical examples of immunological staining for LV T cell (CD3) and (B) macrophage (CD68) presence. Positively labelled cells are depicted with an arrowhead. (C) Quantification of immunological staining for LV T cell (CD3) and (D) macrophage (CD68) presence per image field. n = 4‐7, * *P* < .05. E, Magnified visualization of immunological CD68 labelling in DS‐treated obese rats, illustrative for the observed DS‐induced increase in apoptosis seen in regions with high cellular density

To assess the impact of DS‐induced hypertension and obesity on the cardiac inflammatory state, presence of CD3+ T cells and CD68+ macrophages was evaluated. The number of CD3+ T cells, representing adaptive immunity, was low and not affected by obesity or DS‐induced hypertension (Figure 5A and 5C, Table S9). In contrast, presence of CD68+ macrophages, representing innate immunity, was markedly increased by DS‐induced hypertension (Figure 5B and 5D, Table S9). Obesity alone had no effect, but in combination with DS‐induced hypertension it significantly increased macrophage presence, showing a synergistic trend (interaction *P* = .09). In line with the observed DS‐induced apoptosis, macrophage presence was mostly seen in patchy clusters (Figure 5E).

### 3.6 Fibrosis and TGFβ signalling

**Figure 6:**
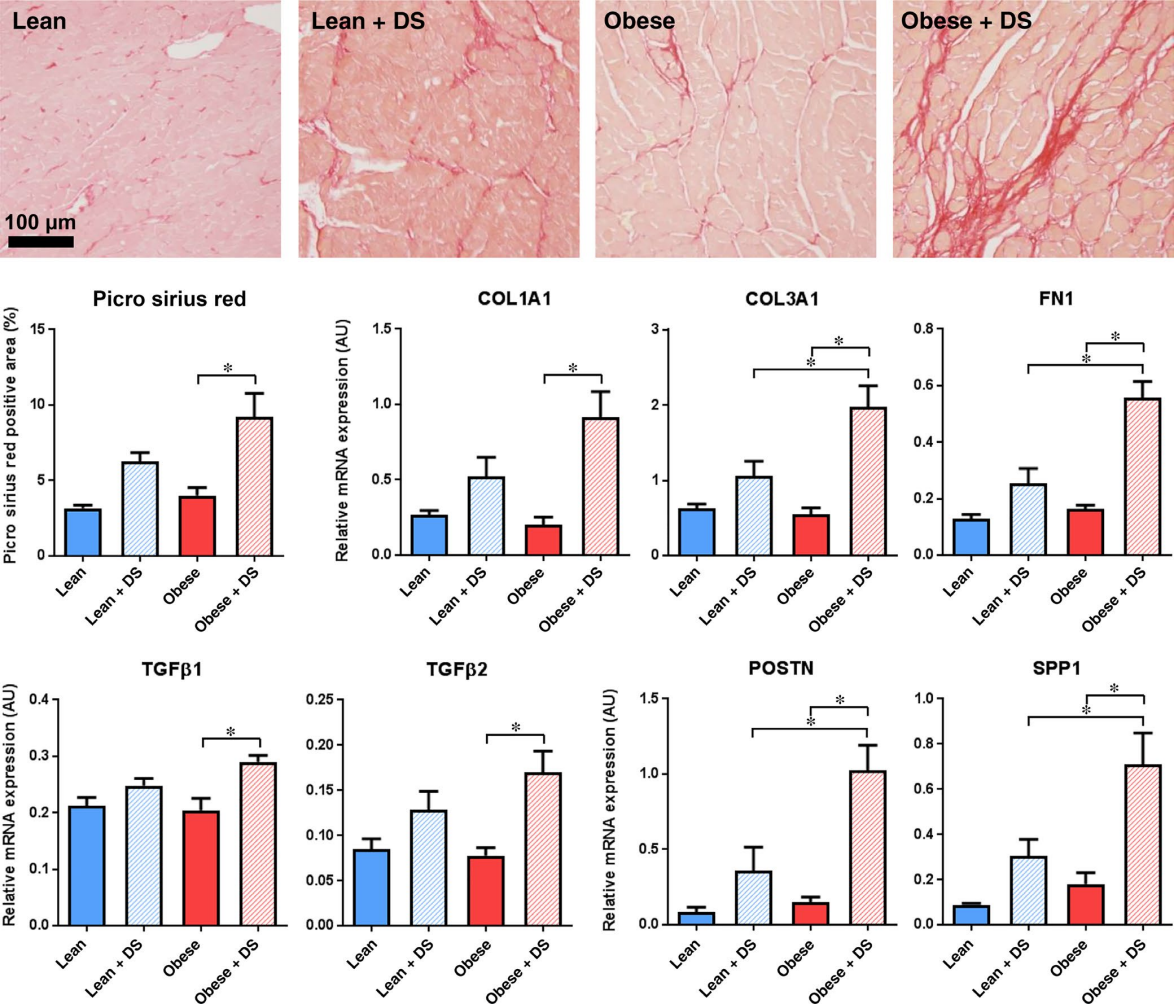
Fibrosis and TGFβ signalling. (A) Typical examples and (B) quantification of histological staining for cardiac collagen deposition (Picro Sirius Red). n = 6‐7, * *P* < .05. (C) QPCR analysis of cardiac transcription of extracellular matrix components COL1A1, COL3A1 and FN1, (D) activators of TGFβ signalling TGFβ1 and TGFβ2 and (E) transcriptional targets of TGFβ signalling POSTN and SPP1. n = 5‐7, * *P* < .05

To evaluate the impact of the comorbidities on the development and progression of fibrosis, collagen deposition was measured. Picro Sirius Red positivity in the placebo‐treated obese ZSF1 rats was not different from the placebo‐treated lean rats. In contrast, DS‐induced hypertension triggered a profound fibrotic response. The DS‐induced fibrosis appeared to be aggravated by obesity, although no significant interaction was observed (interaction *P* = .31; Figure 6A‐B, Table S9). These findings were substantiated by qPCR validation of collagen 1α1 (COL1A1), collagen 3α1 (COL3A1), and fibronectin (FN1) expression. Obesity alone had no effect, whereas DS‐induced hypertension enhanced their transcription. This DS‐induced up‐regulation was aggravated in the presence of obesity (Figure 6C), and significant synergy was observed for COL3A1 and FN1 (Table S9). Based on the overall transcription profiles, IPA predicted high activity of transforming growth factor (TGF)‐β1 (overlap *P* = 2.81E‐11/Z‐score = 4.01 and overlap *P* = 2.95E‐31/Z‐score = 6.24 in lean + DS vs lean and obese + DS vs obese, respectively) and TGFβ2 (overlap *P* = 2.14E‐4/Z‐score = 1.75 and overlap *P* = 1.82E‐6/Z‐score = 2.51 in lean + DS vs lean and obese + DS vs obese, respectively) signalling in the hearts of DS‐treated vs placebo‐treated rats (Table S10‐S11). This predicted activation was accompanied by transcriptional up‐regulation of TGFβ1 and TGFβ2 (Figure 6D) upon DS‐induced hypertension. Moreover, in addition to COL1A1, COL1A3 and FN1, various TGFβ target genes, including periostin (POSTN) and osteopontin 1 (SPP1) were up‐regulated (Figure 6E), of which the latter has recently been demonstrated to have predictive value for clinical outcome in HFpEF.^18^

## 4 DISCUSSION

In line with previous reports,^12^, ^13^ ZSF1 rats with a homozygous leptin receptor mutation in our current study presented with metabolic derangements, including elevated body weight, hypercholesteremia, hyperglycaemia and hypertriglyceridemia. As anticipated, DS treatment triggered severe hypertension in both lean and obese ZSF1 rats, and the combination of these factors enabled us to study if and how obesity and hypertension contribute to the development of diastolic dysfunction at a functional, histological and transcriptional level. From this study, we can conclude that (a) obesity‐associated derangements had the most predominant effect on development of diastolic dysfunction, with no synergy observed between obesity and DS‐induced hypertension; (b) obesity also predominantly affected LV hypertrophic remodelling, which was associated with increased foetal gene expression and Ca^2+^‐mediated transcription; (c) capillary rarefaction was absent in all conditions, whereas inflammation and fibrosis occurred in response to DS‐induced hypertension, with partial synergy observed if in combination with obesity; and that (d) obesity stimulated enhanced expression of genes involved in FAO, whereas DS treatment led to an overall down‐regulation of genes involved in oxidative phosphorylation, implying that the comorbidities triggered different mitochondrial responses. These findings do not support our initial hypothesis and illustrate important phenotypical differences in comorbidity‐induced cardiac adaptation.

Diastolic dysfunction, or more specifically, impaired active relaxation and reduced LV passive compliance leading to increased LV diastolic pressures, is a phenotypical hallmark of HFpEF.^19^, ^20^ A well‐defined echocardiographic parameter to define presence of diastolic dysfunction is the E/e’ ratio,^21^ which was significantly elevated in both placebo‐ and DS‐treated obese rats, compared with their lean counterparts. Similarly, obesity was strongly associated with a lower CO. Interestingly, DS‐induced hypertension had no effect on CO and only a minor effect on E/e’ ratio. Surprisingly, no functional interaction was observed between the comorbidities. These findings, indicating a relatively mild contribution of hypertension on development and progression of diastolic dysfunction and suggesting a more pronounced causal relation with metabolic derangements, could explain why antihypertensive treatment thus far has not established a major survival benefit in HFpEF patients.^11^

The lowering of CO induced by obesity was principally the result of a lower heart rate, which is consistent with diabetes‐induced lowering of resting heart rates,^22^ while—surprisingly—stroke volume was maintained even when both obesity and DS were present. Stroke volume was likely maintained as a result of the bradycardia‐induced increase in diastolic filling time, thereby compensating for the impaired relaxation and the presence of concentric hypertrophy. In addition to stroke volume, it has previously been demonstrated that dynamics in heart rate can also affect indices of diastolic function, though E/e’, which was our primary readout for diastolic function, does not appear to be affected by differences in heart rate.^23^ In addition to E/e’ and CO, EF was minimally affected by either obesity or DS‐induced hypertension. Currently, there are no clear cut‐off values for preserved or reduced EF in rats. However, baseline EF determined by echocardiography in healthy male Sprague‐Dawley rats is 64 ± 7%,^24^ suggesting that the lowest EF of 59 ± 2%, as observed for the DS‐treated obese rats, could be considered as a preserved EF.

In the ZSF1 rats, total HW and LVW reached statistical significance only when both comorbidities were present, although variance was individually affected both by obesity and DS. Remarkably, variance in cardiomyocyte cross‐sectional area was solely affected by obesity, suggesting that the effect of DS treatment on HW might at least in part be secondary to a cellular hypertrophy‐independent process, such as fibrosis. Noteworthy, in none of the hypertrophy‐related parameters, interaction was observed between hypertension and obesity. Transcriptional analysis supported the predominant obesity‐induced myocardial maladaptation, which generally occurs in the presence of reinitiated foetal gene expression and elevated Ca^2+^‐induced transcription.^17^, ^25^ In that regard, a clear obesity‐driven switch from MYH6 to MYH7 was noticed, as well as elevated transcription of RCAN1, a defined target gene of the Ca^2+^‐responsive transcription factor NFAT.^26^ The RNAseq data also underscored the notion that diastolic dysfunction‐associated hypertrophy can develop in absence of a severe increase in cardiac afterload. In the ZSF1 rats, blood pressure‐ and wall stretch‐responsive factor NPPA 27 was exclusively up‐regulated in DS‐treated rats, whereas in placebo‐treated obese rats with an apparent absence of elevated wall stretch, hypertrophy developed. As NPPA has been demonstrated to inhibit NFAT activation,^25^ this DS‐induced up‐regulation of NPPA could explain why RCAN1 was not up‐regulated in DS‐treated rats, even though crucial Ca^2+^‐handling genes (ATP1A2, SERCA2A and PLN) were dysregulated upon DS‐induced hypertension. Besides its role as second messenger in various signal transduction cascades, Ca^2+^—and its removal from the cytosol—plays an important role in active relaxation. As diastolic dysfunction was most prominent in obese animals, yet both obesity and DS treatment lowered the expression of several key Ca^2+^‐handling genes, our results suggest that in this model impaired passive rather than active relaxation contributed to the observed diastolic dysfunction.

In addition to cardiomyocyte hypertrophy, it has been proposed that HFpEF‐associated comorbidities can induce systemic inflammation, leading to endothelial inflammation and oxidative stress, favouring monocyte extravasation, fibrosis and microvascular rarefaction.^2^ Fibrosis is suspected to result from monocyte‐induced TGFβ secretion, and to actively contribute to LV stiffening,^28^ whereas a reduction in capillary density may impair oxygen delivery, limiting systolic and diastolic reserve function,^29^ yet could also lower NO availability which contributes to passive stiffness and LV hypertrophy via impaired Protein Kinase G activation.^2^ In LV sections from HFpEF patients, indeed fibrosis and a lower capillary density were observed,^29^ but, in line with our previous study,^13^ no capillary rarefaction was observed in any of the ZSF1 rats. From the current study, it cannot be excluded that loss of capillaries develops at a later stage of the disease, although these data do suggest that it is not a prerequisite for the development of diastolic dysfunction. Interestingly, even in the absence of capillary rarefaction, a clear inflammatory and fibrotic response was observed. In contrast to the obesity‐driven diastolic dysfunction and cardiomyocyte hypertrophy, inflammation and fibrosis predominantly occurred in DS‐induced hypertensive rats. It has previously been shown that resident cardiac macrophages can respond to hypertension by activation of pro‐inflammatory signalling,^30^ due to exposure to altered mechanical forces.^31^ Additionally, DS‐induced activation of mineralocorticoid receptors could also trigger cardiac inflammation and fibrosis, independent of hypertension,^32^, ^33^ implying that multiple mechanisms could orchestrate the observed response in the hypertensive rats. In placebo‐treated obese rats, no inflammatory or fibrotic responses were observed, suggesting that the increased urinary TBARS excretion in these rats was presumably not derived from vascular and neutrophilic NADPH‐induced oxidative stress.^34^ Interestingly, obesity did significantly aggravate DS‐induced macrophage presence and triggered up‐regulation of many extracellular matrix components with a TGFβ signature,^35^, ^36^ suggesting putative interaction with DS‐induced hypertension for this particular aspect of LV adaptation. However, obesity and DS treatment also showed mild interaction in elevation of the systolic blood pressure. Considering that elevated salt sensitivity has often been demonstrated for obesity,^37^, ^38^, ^39^ this elevation of SBP is not an unexpected interaction. In fact, this may represent an important mechanism through which interaction between these comorbidities occurs. Although the elevated body mass in obese animals mainly consists of fat, the contribution of which to extracellular fluid is limited compared to non‐fat tissue, the normalized urinary sodium excretion data suggests that the observed synergy does not simply develop via elevated food and thus increased salt intake in the obese rats. Nonetheless, from these data it cannot be excluded that the observed increase in inflammation and fibrosis, the only parameters for which significant interaction was observed, was mainly hypertension‐driven. However, the latter would plead the case for limited synergy between obesity and hypertension even more.

The broad range of our analysis enabled us to dissect how functional differences in cardiac response to obesity‐associated aberrations and DS‐induced hypertension were reflected by the distinct transcription profiles. By comparing the transcriptome of placebo‐treated obese rats with that of placebo‐treated lean rats, the most abundant response came from elevated expression of genes encoding enzymes involved in FAO. Although cardiac hypertrophy is associated with decreased FAO and increased glucose metabolism,^40^ this finding was not completely unexpected considering the diabetic background of these rats.^41^ Nonetheless, increased FAO is also evident in HFpEF patients,^42^ and due to the lower oxygen efficiency compared with glucose metabolism,^43^ this could result in a lower ATP bioavailability, which can contribute to hampered energy‐consuming diastolic relaxation.^44^ The increased FAO, which may partly occur in peroxisomes,^45^ might also be causally related to the observed elevation of oxidative stress. As peroxisomes lack a respiratory chain, electrons from FADH_2_ are transferred directly to O_2_, generating H_2_O_2_ and heat.^45^ In line with previous studies,^46^ obesity appeared to induce peroxisomal FAO, as illustrated by higher expression of peroxisomal‐specific Acyl‐Coenzyme A Oxidase 1 (ACOX1) and Acyl‐CoA Dehydrogenase Very Long Chain (ACADVL). The up‐regulation of CAT in placebo‐treated obese rats, which is predominantly involved in peroxisomal H_2_O_2_ conversion,^45^ substantiates the putative peroxisomal source of oxidative stress.

Strikingly, when transcription profiles of DS‐treated rats were compared with those of placebo‐treated rats, an overall down‐regulation of factors involved in oxidative phosphorylation was observed, negatively modulating both fatty acid and glucose oxidation. Others have shown that down‐regulation of only a minimal number of genes involved in oxidative phosphorylation could induce mitochondrial dysfunction.^14^, ^15^ Mitochondrial dysfunction is causally related to myocardial cell death,^47^, ^48^ which might explain the effect of DS on sub‐endocardial TUNEL labelling. The relation between hypertension and mitochondrial dysfunction is well described.^49^, ^50^ However, the present findings illustrate the different effects of two HFpEF comorbidities on energy homeostasis, indicating that in obese rats the mechanisms involved in metabolic derangements, with a putative role in development of diastolic dysfunction, are dependent on the presence or absence of hypertension. These observations emphasize the need for thorough phenotypical characterization of HFpEF patients.^51^

In summary, the present study shows, to our knowledge for the first time, how obesity and hypertension contribute to the development of diastolic dysfunction. Our data show that intrinsic metabolic derangements in ZSF1 rats have the most evident effect on development of diastolic dysfunction. However, from a mitochondrial, fibrotic, and inflammatory point of view, the presence of severe hypertension appears to have a major impact on disease progression. When considering therapeutic interventions, it thus would seem extremely important to anticipate potential pathophysiological differences among HFpEF patients that were thus far grouped together based on the common denominator ‘preserved EF’. For instance, aiming for a switch from fatty acids towards more oxygen‐efficient glucose metabolism, as was previously suggested in HFpEF,^52^ might provide an opening with promising therapeutic potential in obese patients, but may well be far less effective when applied to HFpEF patients with hypertension‐induced mitochondrial dysfunction.

## CONFLICT OF INTEREST

None declared.

## Data Availability

The data that support the findings of this study are available from the corresponding author upon reasonable request.
