# Supplementary material for: Limited synergy of obesity and hypertension, prevalent risk factors in onset and progression of heart failure with preserved ejection fraction
Source: J Cell Mol Med. 2019 Jul 31;23(10):6666–78. doi: 10.1111/jcmm.14542 (PMC6787495; doi:10.1111/jcmm.14542)
Supplement: Supplementary file 1 [file JCMM-23-6666-s001.docx]

**Supplemental data**

**Limited synergy of obesity and hypertension, prevalent risk factors in onset and progression of heart failure with preserved ejection fraction**

Maarten M. Brandt^1,2^*, Isabel T. N. Nguyen^2^*, Merle M. Krebber^2^, Jens van de Wouw^1^, Michal Mokry^3,4^, Maarten J. Cramer^5^, Dirk J. Duncker^1^, Marianne C. Verhaar^2^, Jaap A. Joles^2‡^, Caroline Cheng^1,2‡^

*^‡^: Authors contributed equally

*^1^Experimental Cardiology, Department of Cardiology, Thoraxcenter Erasmus University Medical Center, Rotterdam; ^2^Department of Nephrology and Hypertension, University Medical Center Utrecht, Utrecht; ^3^Epigenomics facility, University Medical Center Utrecht, Utrecht; ^4^Regenerative Medicine Center Utrecht, University Medical Center Utrecht, Utrecht ^5^Department of Cardiology, University Medical Center Utrecht, Utrecht; The Netherlands*

**Corresponding author:**

Caroline Cheng, PhD

Department of Nephrology and Hypertension (F03.223)

University Medical Center Utrecht

PO Box 85500, 3508 GA Utrecht, The Netherlands

T: +31 (0)-88-7557329

E-mail: K.L.Cheng-2@umcutrecht.nl

**Supplemental methods**

*Plasma measurements*

Blood samples of 0.5 ml were drawn from the tail vein into EDTA anticoagulant-coated tubes, while the rats were under anesthesia. Plasma was extracted by centrifugation at 1500 x g for 15 min at 4°C and used for determination of plasma glucose, cholesterol and triglycerides (DiaSys Diagnostic Systems GmbH, Cholesterol FS, Triglycerides FS, Glucose GOD FS, Waterbury, Connecticut, USA).

*Urinary sodium and TBARS excretion*

Rats were housed individually in metabolic cages with access to food and water to collect 24 hours urine at 26 weeks of age. In urine samples, thiobarbituric acid-reactive substances (TBARS; TBARS assay kit, Cayman Chemical, Ann Arbor, Michigan, USA) were measured according to manufacturer’s instructions. Sodium concentrations were determined by flame photometry (Model 420, Sherwood, UK).

*Immunohistochemistry*

Deparaffinized cardiac sections were subjected to 3% hydrogen peroxide in PBS for 30 min, followed by heat-induced antigen retrieval in citrate/HCl buffer (pH 6.0) for 15 min. To visualize capillaries, sections were first blocked in avidin and biotin blocking solution (Abcam, Cambridge, UK). Section were then incubated overnight at 4°C with biotin labelled anti-Lectin from *Bandeiraea simplicifolia* (1:200, Sigma, St. Louis, Missouri, USA), after which HRP-bound streptavidin (1:500, Bio-Rad, Veenendaal, the Netherlands) was added to the samples for 60 min. Finally, 3,3′-diaminobenzidine solution was applied to the sections twice, 6 min each. To visualize early apoptosis, sections were incubated for 60 min at room temperature (RT) with a primary antibody against active Caspase 3 (1:200, BD Biosciences, San Jose, California, USA), followed by 30 min incubation at RT with BrightVision PolyGoat-HRP-anti-Rabbit IgG (Immunologic BV, Duiven, the Netherlands). To visualize innate inflammation, sections were incubated for 60 min at RT with a primary antibody against CD68 (1:500, Abcam), followed by 30 min incubation at RT with Poly-Rabbit-anti-Mouse (Abcam) and 30 min incubation at RT with BrightVision PolyGoat-HRP-anti-Rabbit IgG. To visualize adaptive immunity, sections were first blocked in avidin and biotin blocking solution. Thereafter, sections were incubated for 60 min at RT with a primary antibody against CD3 (1:200, Abcam), followed by 30 min incubation at RT with BrightVision PolyGoat-HRP-anti-Rabbit IgG. For all immunostainings (with the exception of lectin), finally NovaRed buffer was applied to the sections for 10 min, and a counterstaining was performed via a brief dip in hematoxylin. Late apoptosis was fluorescently visualized in deparaffinized cardiac sections, which were incubated 30 min at 37°C in a 20µg/mL proteinase K solution in 10mM Tris/HCL buffer (pH 8.0). After a brief wash, TUNEL labeling solution (Sigma) was applied according to manufacturer’s instruction for 60 min at 37°C. Nuclei were counterstained with DAPI. For all immunostainings, four non-overlapping fields in the sub-endocardium were imaged and analyzed in a blinded fashion.

*Gomori staining*

Gomori staining was performed using the Reticulum Stain Kit (Diagnostic Biosystems, Pleasanton, California, USA) according to manufacturer’s instructions. Slides were counterstained for 3 min using Nuclear Fast Red, and imaged. Cross-sectional areas of cardiomyocytes with clearly visible nuclei, and height to width ratios not exceeding 1:2, were measured in 4 non-overlapping fields in a blinded fashion using Clemex software.

*Picrosirius red staining*

Deparaffinized cardiac sections were stained with Picro Sirius red (Sigma) for 25 min, differentiated in 0.2 HCl and rinsed with aquadest, followed by dehydration. Twenty non-overlapping fields were imaged using a polarizing filter, followed by analysis in a blinded fashion using Adobe Photoshop and ImageJ software as described.[1]

*Identification of gene expression*

RNA sequencing was done as described.[2] Briefly, RNA was isolated from the cardiac apex using the RNeasy isolation kit (Qiagen, Hilden, Germany). NEXTflex Poly(A) Beads (PerkinElmer Applied Genomics, Waltham, Massachusetts, USA) were used to isolate polyadenylated mRNA, from which sequencing libraries were made using the Rapid Directional RNA-seq kit (NEXTflex). Libraries were sequenced using the Nextseq500 platform (Illumina, San Diego, California, USA), producing single end reads of 75bp. Reads were aligned to the human reference genome GRCh37 using STAR version 2.4.2a. Picard’s AddOrReplaceReadGroups (v1.98) was used to add read groups to the BAM files, which were sorted with Sambamba v0.4.5 and transcript abundances were quantified with HTSeq-count version 0.6.1p1 using the union mode. Subsequently, RPKMs were calculated with edgeR’s RPKM function. Differentially expressed genes in the transcriptome data were identified using the DESeq2 package with standard settings.[3]

*Quantitative PCR analysis*

Cardiac apex RNA was reverse transcribed into cDNA using iScript cDNA synthesis kit (Bioline, London, UK). Gene expression was assessed by qPCR using SensiFast SYBR & Fluorecein kit (Bioline) and primers as listed in Supplemental Table 1. Expression levels are relative to the averaged expression of housekeeping genes RPLP0, POLR2A, B2M, and YWHAZ.

*Pathway analysis*

RNAseq results were further analyzed using Ingenuity Pathway Analysis (IPA, Qiagen). IPA was used to identify both differences in pathway dynamics, and upstream regulators (growth factors and transcriptional regulators) of differentially expressed genes. P-values were calculated based on a right-tailed Fisher Exact Test, calculated by IPA.

**Supplemental references**

1. **Bongartz LG, Braam B, Verhaar MC, Cramer MJ, Goldschmeding R, Gaillard CA, Doevendans PA, Joles JA.** Transient nitric oxide reduction induces permanent cardiac systolic dysfunction and worsens kidney damage in rats with chronic kidney disease. *Am J Physiol Regul Integr Comp Physiol*. 2010; 298: R815-23.

2. **Brandt MM, Meddens CA, Louzao-Martinez L, van den Dungen NAM, Lansu NR, Nieuwenhuis EES, Duncker DJ, Verhaar MC, Joles JA, Mokry M, Cheng C.** Chromatin Conformation Links Distal Target Genes to CKD Loci. *J Am Soc Nephrol*. 2018; 29: 462-76.

3. **Love MI, Huber W, Anders S.** Moderated estimation of fold change and dispersion for RNA-seq data with DESeq2. *Genome Biol*. 2014; 15: 550.

**Supplemental figures**

**
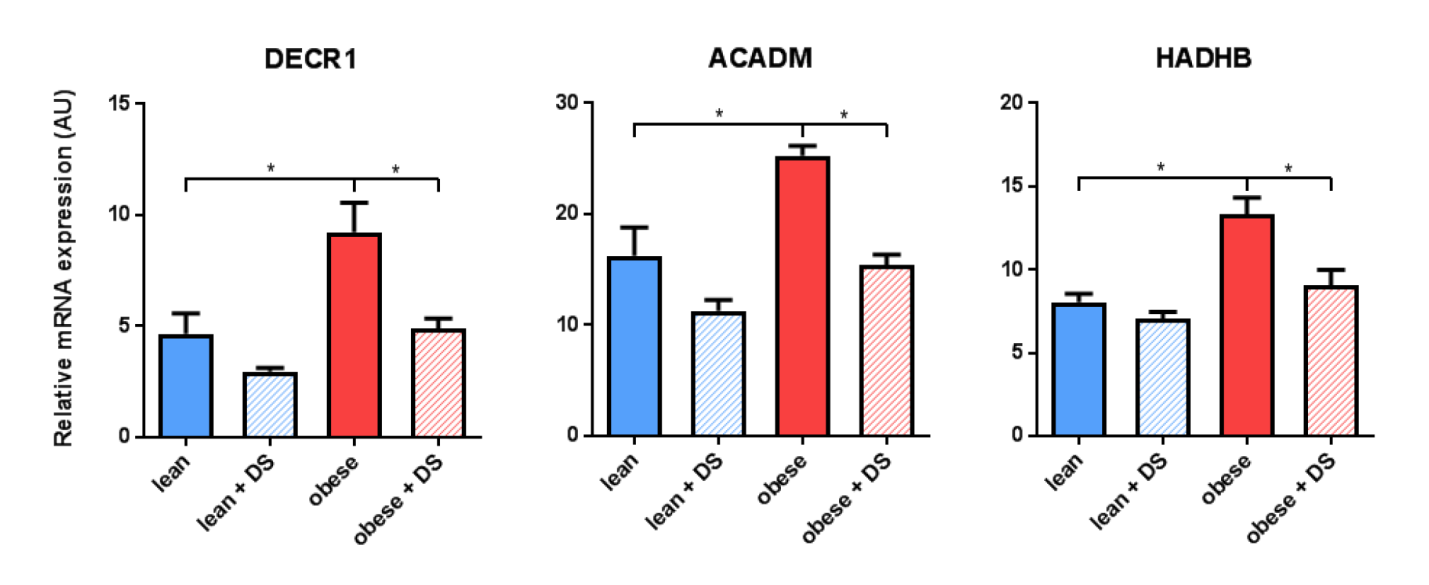
**

**Supplemental figure 1: Transcription of KLF15 target genes. (A)** QPCR results showing expression levels of KLF15 targets DECR1, **(B)** ACADM, and **(C)** HADHB. N=5-7, * P<0.05.


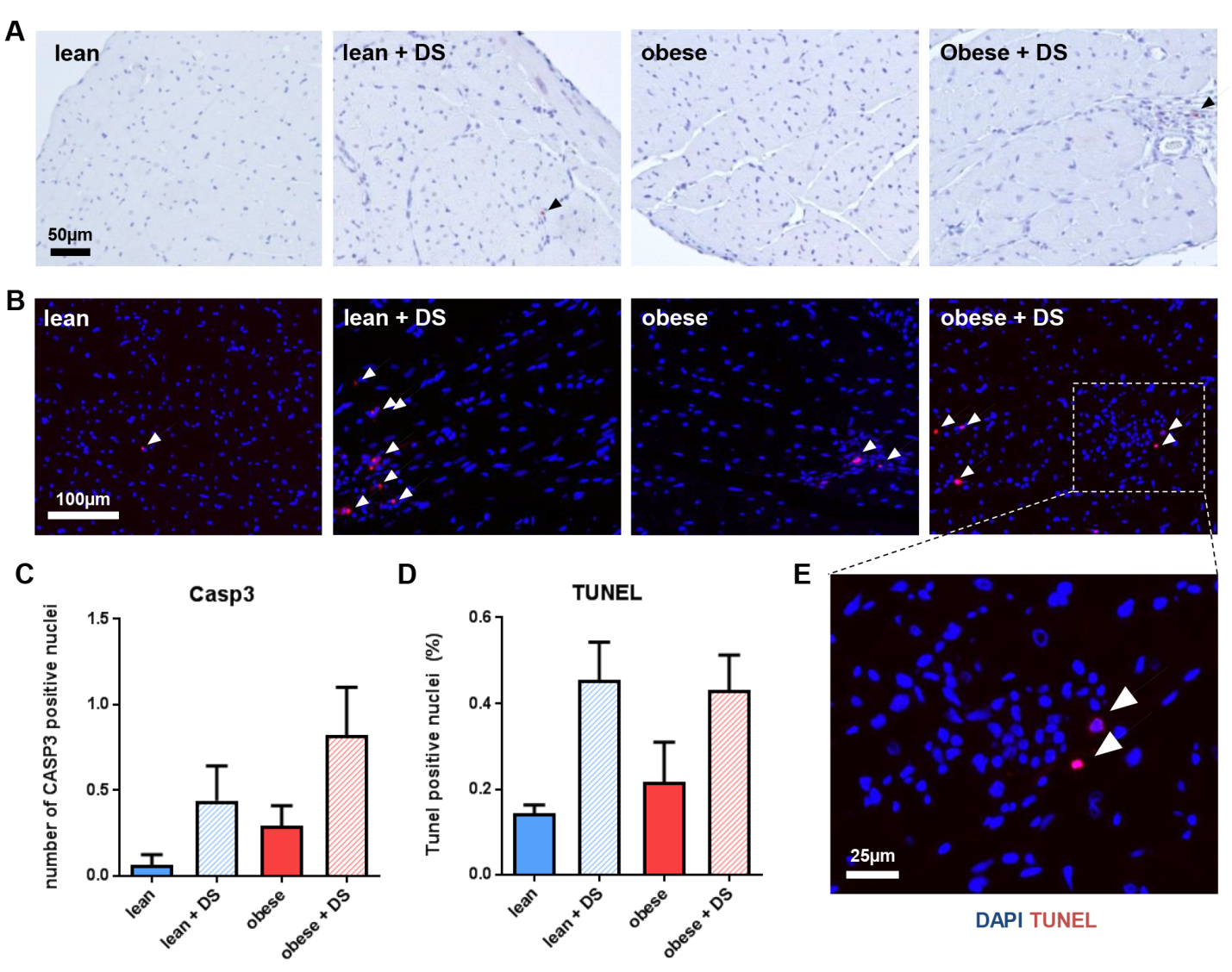


**Supplemental figure 2: Analysis of apoptosis by immunostaining. (A)** Typical examples of histological analysis of LV early apoptosis (activated Caspase3) and **(B)** late apoptosis (TUNEL). Positively labeled cells are depicted with an arrowhead. **(C)** Quantification of histological staining for LV early (activated Caspase3) and **(D)** late (TUNEL) apoptosis. N=4-7. **(E)** Magnified visualization of immunological TUNEL labeling in DS-treated obese rats, illustrative for the observed DS-induced increase in apoptosis seen in regions with high cellular density.

**Supplemental tables**

**Supplemental table 1:** Primer sequences used for (q)PCR.

| Gene | Sense primer sequence | Antisense primer sequence |
| --- | --- | --- |
| RPLP0 | GCTGATAAAGACTGGAGACAAGG | GAAGGAGAAGGGGGAGATGTT |
| POLR2A | CTTTGCTCTTCAACATCCACCT | TCACATTCTTGGCAGATACACC |
| B2M | TCCACCCACCTCAAATAGAAA | GAGCCAGGATGTAGAAAGACCA |
| YWHAZ | CCTGCTGTTTGCTTTCATTATTT | GCCGTCATCTCAAGTTATTTCC |
| MYH6 | CACTCTTCTCCACCTACGCTTC | GCCTTTCTTCTTGCCTCCTT |
| MYH7 | AGAACCAGTCCATCCTCATCAC | TGGCAGCAATAACAGCAAAA |
| NPPA | ACCGAAGATAACAGCCAAATC | AGGGTATTCACCACCTCTCAGT |
| SERCA2A | ACTGTGTGATGTGGAGGAAAT | TGGCTTCTGTTCTTGGATAAG |
| PLN | CAAAGAGTAGCCCACAAAGGA | TCATAGCATCACAAAGGGAACA |
| ATP1A2 | GATGAATGAGGGAAGCAAGAGA | TTTAGGGTAAGGCAAAACTCCA |
| RCAN1 | GAGGAGGAGATGGAGAGAATGAA | AGTCTGGATGATTTTTGGCTTG |
| ANGPT1 | AGTCCATAACCTTGTCAGCCTTT | ATTACTGTCCATCCTCCTCCATT |
| ANGPT2 | ATAACTGGAAAACAGAACCCTCA | ATGTCAAGCACAAGACGGAAC |
| VEGFA | ATTTTTGCTTCCTATTCCCCTCT | TCTCTTCCTTGACTTCTCTCTGG |
| HADHB | TCCAGACCAAGTCAAAGAAGAC | CGATACAACAAACCCGAAAGTG |
| ACADM | GGCTTTACTTGTCTTCCCACAG | CCAGGGTATTTCTCCATCTCAA |
| DECR1 | GATTTGACGGTGGAGAGGAA | GGAGCCTTTTGTCTTTCTGATG |
| CAT | GGTGCTTTTGGATACTTTGAGG | GTGGGTTTCTCTTCTGGCTATG |
| COL1A1 | CGTGACCAAAAACCAAAAGTG | GTGGAGAAAGGAACAGAAAAGG |
| COL3A1 | CTGGTGAATGGAGCAAGACA | GGTGGGTGAAACAGCAAAAA |
| FN1 | TTGATGACACTTCCATTGTTGTT | TGAGTTCTGTGCTACTGCCTTCT |
| TGFB1 | CGCAATCTATGACAAAACCAAA | ACAGCCACTCAGGCGTATC |
| TGFB2 | CATCCCAAATAAGAGCCAAGAG | TGTAGGAGGGCAACAACATTAG |
| POSTN | TGATAGTCTCCTGTGGGGTAGG | GGTAACTGTGTGCTTTGGGTTT |
| SPP1 | AGTGGTTTGCTTTTGCCTGTT | GCATCTGAGTGTTTGCTGTAATG |

**Supplemental table 2:** Differentially expressed genes in obese versus lean rats.

| **ID** | **baseMean** | **log2FoldChange** | **FDR** | **symbol** |
| --- | --- | --- | --- | --- |
| ENSRNOG00000018794 | 531,41 | -1,64 | 5,99E-23 |  |
| ENSRNOG00000049766 | 65,84 | -2,21 | 7,68E-23 | Sctr |
| ENSRNOG00000007545 | 314,89 | 1,58 | 1,21E-17 | Angptl4 |
| ENSRNOG00000010134 | 3651,02 | 1,29 | 4,31E-16 | Acot2 |
| ENSRNOG00000008236 | 2612,61 | 1,12 | 4,31E-16 | Decr1 |
| ENSRNOG00000007290 | 2295,88 | -0,76 | 3,03E-13 | Atp1a2 |
| ENSRNOG00000033893 | 132,47 | 1,42 | 2,84E-12 | Cacna1h |
| ENSRNOG00000014948 | 126,63 | 1,24 | 7,68E-11 | Osgin1 |
| ENSRNOG00000018145 | 5912,01 | 0,64 | 1,88E-10 | Crat |
| ENSRNOG00000017806 | 24,78 | 1,55 | 2,21E-10 | RGD1564827 |
| ENSRNOG00000009845 | 14968,58 | 0,68 | 2,21E-10 | Acadm |
| ENSRNOG00000013766 | 13294,30 | 0,83 | 9,11E-10 | Acaa2 |
| ENSRNOG00000011648 | 6085,16 | -0,84 | 2,43E-09 | Aqp1 |
| ENSRNOG00000024629 | 11025,22 | 0,72 | 3,5E-09 | Hadha |
| ENSRNOG00000017226 | 3522,94 | -0,80 | 8,39E-09 | Slc2a4 |
| ENSRNOG00000009686 | 1115,72 | 0,95 | 8,41E-09 | Aqp7 |
| ENSRNOG00000020308 | 7253,08 | 0,83 | 1,69E-08 | Ech1 |
| ENSRNOG00000001736 | 437,20 | -1,03 | 1,69E-08 | Bdh1 |
| ENSRNOG00000010800 | 13622,85 | 0,75 | 4,53E-08 | Hadhb |
| ENSRNOG00000034290 | 388,81 | -0,84 | 5,23E-08 | Ccl21 |
| ENSRNOG00000002208 | 1655,55 | 0,80 | 6,37E-08 | Shroom3 |
| ENSRNOG00000016983 | 143739,92 | 0,98 | 1,27E-07 | Myh7 |
| ENSRNOG00000046468 | 419,64 | 1,03 | 1,88E-07 | Ptgfr |
| ENSRNOG00000010872 | 1379,09 | -0,76 | 1,91E-07 | Ckb |
| ENSRNOG00000014090 | 2592,45 | 0,70 | 2,38E-07 | Retsat |
| ENSRNOG00000019120 | 179,06 | 1,20 | 2,83E-07 | Hmgcs2 |
| ENSRNOG00000016692 | 3995,39 | 0,62 | 5,26E-07 | Hsdl2 |
| ENSRNOG00000001979 | 8085,23 | 0,80 | 1,26E-06 | Rcan1 |
| ENSRNOG00000029571 | 860,00 | -0,65 | 1,3E-06 | Coq10a |
| ENSRNOG00000021213 | 1712,63 | 0,56 | 1,88E-06 | Lix1l |
| ENSRNOG00000050997 | 684,19 | 0,78 | 1,88E-06 | Ifrd1 |
| ENSRNOG00000002128 | 773,45 | 0,56 | 4,21E-06 | Ppat |
| ENSRNOG00000016388 | 348,21 | 1,02 | 1,06E-05 | Sphkap |
| ENSRNOG00000008831 | 909,21 | -0,73 | 3,84E-05 | Hcn2 |
| ENSRNOG00000008843 | 2336,19 | 0,71 | 4,65E-05 | Eci1 |
| ENSRNOG00000021812 | 136,85 | -0,87 | 4,95E-05 | Scx |
| ENSRNOG00000012966 | 20061,65 | 0,49 | 4,95E-05 | Acadl |
| ENSRNOG00000006116 | 446,37 | -0,89 | 5,19E-05 | Hk2 |
| ENSRNOG00000010697 | 3136,75 | 0,59 | 5,66E-05 | Hadh |
| ENSRNOG00000015840 | 1924,55 | 0,45 | 6,14E-05 | Hsd17b4 |
| ENSRNOG00000007896 | 195,90 | -0,89 | 6,29E-05 | Klhl38 |
| ENSRNOG00000010216 | 1327,28 | 0,56 | 6,29E-05 |  |
| ENSRNOG00000009227 | 280,59 | -0,95 | 6,65E-05 | Aplnr |
| ENSRNOG00000020737 | 269,79 | 0,65 | 6,83E-05 | Cdc25a |
| ENSRNOG00000025350 | 666,58 | 0,56 | 6,98E-05 | Ppp1r13l |
| ENSRNOG00000011427 | 43,15 | -1,09 | 7,98E-05 | Hr |
| ENSRNOG00000016023 | 982,17 | 0,66 | 9,12E-05 | Kank1 |
| ENSRNOG00000006305 | 2860,59 | 0,55 | 9,12E-05 | Slc38a2 |
| ENSRNOG00000034228 | 18,16 | 1,03 | 0,000109 | Pik3c2g |
| ENSRNOG00000009085 | 429,74 | 0,66 | 0,000135 | Prkag2 |
| ENSRNOG00000001414 | 1910,44 | 0,70 | 0,000187 | Serpine1 |
| ENSRNOG00000024039 | 517,80 | -0,60 | 0,000187 | Serinc5 |
| ENSRNOG00000025295 | 1326,77 | 0,39 | 0,000197 | Mavs |
| ENSRNOG00000033627 | 69,58 | 1,02 | 0,000221 |  |
| ENSRNOG00000006972 | 168,20 | 0,71 | 0,000228 | Zfp189 |
| ENSRNOG00000001007 | 160,75 | 0,82 | 0,000228 | Baiap2l1 |
| ENSRNOG00000008118 | 323,30 | 0,57 | 0,000228 | Sync |
| ENSRNOG00000001711 | 158,26 | -0,78 | 0,000228 | Hrasls |
| ENSRNOG00000004009 | 117,56 | -0,87 | 0,000228 | Xpnpep2 |
| ENSRNOG00000039197 | 388,04 | -0,71 | 0,000228 | LOC108348074 |
| ENSRNOG00000006694 | 2791,50 | -0,55 | 0,000235 |  |
| ENSRNOG00000008747 | 456,72 | 0,71 | 0,000256 | Plekha5 |
| ENSRNOG00000047708 | 451,09 | -0,64 | 0,000263 | Gstz1 |
| ENSRNOG00000011189 | 251,37 | -0,74 | 0,000297 | Acy1 |
| ENSRNOG00000011094 | 168,54 | -0,71 | 0,000333 | Efcab6 |
| ENSRNOG00000011976 | 300,17 | 0,61 | 0,000369 | Nudt7 |
| ENSRNOG00000028311 | 48,67 | 0,97 | 0,000395 |  |
| ENSRNOG00000018524 | 547,00 | 0,58 | 0,000462 | Ezr |
| ENSRNOG00000000082 | 1483,23 | 0,56 | 0,00047 | Hltf |
| ENSRNOG00000010596 | 55,12 | -0,98 | 0,000556 |  |
| ENSRNOG00000008369 | 230,99 | -0,74 | 0,000556 | Gimap4 |
| ENSRNOG00000004205 | 638,52 | 0,71 | 0,000566 | Pkdcc |
| ENSRNOG00000018232 | 1188,97 | 0,42 | 0,00058 | Srf |
| ENSRNOG00000016855 | 961,51 | 0,52 | 0,000591 | B3galnt2 |
| ENSRNOG00000004918 | 38,94 | 0,93 | 0,000629 | Kcna4 |
| ENSRNOG00000024066 | 396,60 | -0,66 | 0,000796 | Fundc2 |
| ENSRNOG00000001256 | 988,81 | 0,46 | 0,000817 | Atxn2 |
| ENSRNOG00000003649 | 563,46 | 0,54 | 0,000822 | Qsox1 |
| ENSRNOG00000005854 | 298,03 | 0,81 | 0,000849 | Angpt1 |
| ENSRNOG00000033887 | 259,10 | 0,63 | 0,000858 |  |
| ENSRNOG00000011994 | 697,27 | 0,65 | 0,000874 | Perp |
| ENSRNOG00000038044 | 290,64 | 0,72 | 0,001025 | Tsc22d2 |
| ENSRNOG00000016243 | 16100,13 | 0,48 | 0,001092 | Casq2 |
| ENSRNOG00000016369 | 2056,70 | 0,45 | 0,001167 |  |
| ENSRNOG00000021024 | 475,45 | 0,56 | 0,001256 | Pi4kb |
| ENSRNOG00000002429 | 41,84 | 0,94 | 0,001256 |  |
| ENSRNOG00000000605 | 65,07 | -0,93 | 0,001273 | Hs3st5 |
| ENSRNOG00000021380 | 181,57 | -0,65 | 0,001273 | Fads6 |
| ENSRNOG00000007475 | 704,15 | 0,78 | 0,001301 | Gpihbp1 |
| ENSRNOG00000008755 | 4631,65 | 0,40 | 0,001401 | Acox1 |
| ENSRNOG00000017428 | 1251,22 | 0,60 | 0,001435 | Map1b |
| ENSRNOG00000017765 | 235,27 | -0,62 | 0,001475 | Net1 |
| ENSRNOG00000008364 | 4192,02 | 0,54 | 0,001475 | Cat |
| ENSRNOG00000025757 | 154961,17 | -0,62 | 0,001642 | Myh6 |
| ENSRNOG00000049911 | 40,99 | 0,92 | 0,001664 | LOC102556347 |
| ENSRNOG00000005482 | 183,12 | -0,66 | 0,001812 | Sap30bp |
| ENSRNOG00000048282 | 197,78 | -0,64 | 0,001846 | Mpnd |
| ENSRNOG00000007808 | 38,84 | -0,92 | 0,001911 | Nap1l5 |
| ENSRNOG00000026037 | 134,07 | 0,75 | 0,001911 |  |
| ENSRNOG00000012842 | 366,03 | 0,58 | 0,001981 |  |
| ENSRNOG00000019892 | 633,30 | 0,59 | 0,001996 | Lrrfip1 |
| ENSRNOG00000014166 | 68,09 | -0,82 | 0,002049 | Smoc2 |
| ENSRNOG00000021478 | 579,01 | 0,56 | 0,002049 | Tpd52l1 |
| ENSRNOG00000050585 | 2127,25 | 0,43 | 0,002049 | Pgam1 |
| ENSRNOG00000036732 | 233,42 | 0,73 | 0,002097 |  |
| ENSRNOG00000015003 | 65,55 | 0,84 | 0,002363 | Pex11a |
| ENSRNOG00000001557 | 58,81 | 0,82 | 0,002473 | Cxadr |
| ENSRNOG00000007583 | 4285,22 | 0,45 | 0,002493 | Pygb |
| ENSRNOG00000012443 | 2786,75 | 0,45 | 0,002569 | Cpt2 |
| ENSRNOG00000008142 | 326,09 | 0,61 | 0,00257 | Brpf1 |
| ENSRNOG00000010438 | 6030,54 | 0,37 | 0,002791 | Cpt1b |
| ENSRNOG00000015736 | 410,82 | -0,61 | 0,002939 | Dhrs3 |
| ENSRNOG00000025371 | 504,57 | -0,60 | 0,002965 | Spry1 |
| ENSRNOG00000017120 | 108,07 | -0,76 | 0,002976 | Abhd2 |
| ENSRNOG00000003078 | 1750,48 | 0,33 | 0,003119 | Dcaf6 |
| ENSRNOG00000020871 | 2864,03 | -0,52 | 0,003305 | Ltbp4 |
| ENSRNOG00000016460 | 16826,53 | -0,43 | 0,003405 | Clu |
| ENSRNOG00000020288 | 2608,68 | 0,41 | 0,003953 | Slc25a20 |
| ENSRNOG00000001005 | 84,34 | 0,81 | 0,004056 | Fcer2 |
| ENSRNOG00000008989 | 213,57 | 0,64 | 0,004086 | Cryl1 |
| ENSRNOG00000009741 | 491,35 | -0,65 | 0,004086 | Cyp4a3 |
| ENSRNOG00000005332 | 691,96 | 0,51 | 0,004154 | Csdc2 |
| ENSRNOG00000047046 | 2066,74 | 0,49 | 0,004571 |  |
| ENSRNOG00000029042 | 16833,02 | -0,78 | 0,00465 | ND6 |
| ENSRNOG00000005046 | 1094,10 | -0,47 | 0,005329 | Tspan13 |
| ENSRNOG00000014940 | 12,80 | -0,81 | 0,005524 | Sfrp5 |
| ENSRNOG00000006689 | 376,69 | 0,55 | 0,005703 | Chd7 |
| ENSRNOG00000003537 | 2338,06 | 0,49 | 0,005728 | Spta1 |
| ENSRNOG00000019661 | 38,32 | 0,80 | 0,005983 | Gdf15 |
| ENSRNOG00000046984 | 376,77 | 0,48 | 0,005983 | St6galnac6 |
| ENSRNOG00000047409 | 10,10 | -0,76 | 0,005983 |  |
| ENSRNOG00000018404 | 952,50 | 0,42 | 0,00628 | Aars |
| ENSRNOG00000017637 | 120,62 | 0,76 | 0,006576 | Fbp2 |
| ENSRNOG00000047860 | 535,99 | 0,65 | 0,006837 | Plin5 |
| ENSRNOG00000009480 | 166,75 | 0,76 | 0,007023 |  |
| ENSRNOG00000013663 | 118,54 | -0,62 | 0,00721 | Tmem86a |
| ENSRNOG00000004794 | 218,51 | -0,64 | 0,007417 | Rtn1 |
| ENSRNOG00000020169 | 215,28 | -0,65 | 0,007417 | Gimap8 |
| ENSRNOG00000007461 | 1222,17 | 0,34 | 0,007417 | Klhl41 |
| ENSRNOG00000026119 | 3583,93 | 0,40 | 0,007417 |  |
| ENSRNOG00000021206 | 873,41 | -0,43 | 0,00756 | Pla2g16 |
| ENSRNOG00000010580 | 429,56 | 0,60 | 0,007597 | Acot7 |
| ENSRNOG00000050534 | 501,93 | 0,58 | 0,007597 | Gcnt1 |
| ENSRNOG00000003151 | 1223,07 | 0,31 | 0,007618 | Bfar |
| ENSRNOG00000012840 | 11021,32 | -0,37 | 0,00773 | Sparc |
| ENSRNOG00000048258 | 689,92 | 0,38 | 0,00773 | Cisd2 |
| ENSRNOG00000008210 | 109,77 | -0,82 | 0,00814 | Ky |
| ENSRNOG00000008933 | 1161,88 | 0,47 | 0,00814 | Plbd1 |
| ENSRNOG00000010417 | 40,54 | 0,79 | 0,008381 | Nol4l |
| ENSRNOG00000011977 | 426,36 | 0,50 | 0,008694 | Sema5a |
| ENSRNOG00000005632 | 547,80 | 0,49 | 0,008814 |  |
| ENSRNOG00000005857 | 15,54 | -0,78 | 0,008814 | Lrrc3b |
| ENSRNOG00000010320 | 1514,84 | 0,50 | 0,008814 | Efnb3 |
| ENSRNOG00000016691 | 591,27 | 0,54 | 0,009132 |  |
| ENSRNOG00000014104 | 219,45 | 0,65 | 0,00953 | Myo5b |
| ENSRNOG00000019179 | 469,35 | 0,49 | 0,009772 | Ggta1 |
| ENSRNOG00000009332 | 46,62 | 0,82 | 0,009858 | Rec114 |
| ENSRNOG00000007600 | 444,97 | 0,51 | 0,010078 | Igsf1 |
| ENSRNOG00000011439 | 267,13 | 0,59 | 0,010365 | Grk5 |
| ENSRNOG00000018378 | 698,78 | 0,52 | 0,010907 | Cacnb2 |
| ENSRNOG00000040201 | 81,90 | 0,79 | 0,010907 | Atp6ap1l |
| ENSRNOG00000026745 | 155,99 | -0,66 | 0,010922 | Acsl6 |
| ENSRNOG00000001766 | 598,68 | 0,49 | 0,011028 | Tfrc |
| ENSRNOG00000050315 | 207,09 | -0,71 | 0,011083 | Dcxr |
| ENSRNOG00000033556 | 538,02 | 0,51 | 0,011083 | Spen |
| ENSRNOG00000017513 | 888,16 | 0,43 | 0,011354 | Miga2 |
| ENSRNOG00000003098 | 413,70 | -0,54 | 0,011354 | Prom1 |
| ENSRNOG00000018114 | 7119,64 | 0,38 | 0,011819 | Acadvl |
| ENSRNOG00000046090 | 223,33 | -0,66 | 0,012012 |  |
| ENSRNOG00000019428 | 1106,55 | -0,55 | 0,012476 | Higd1a |
| ENSRNOG00000045665 | 537,64 | 0,46 | 0,012612 | Amigo1 |
| ENSRNOG00000049378 | 276,58 | 0,50 | 0,013027 |  |
| ENSRNOG00000005386 | 1344,36 | -0,54 | 0,013027 | Kitlg |
| ENSRNOG00000025209 | 1011,89 | -0,52 | 0,013027 | Plxnd1 |
| ENSRNOG00000028649 | 99,18 | 0,72 | 0,013027 | Tox3 |
| ENSRNOG00000007060 | 802,31 | 0,42 | 0,013928 | Plin2 |
| ENSRNOG00000016151 | 1160,96 | 0,50 | 0,014549 | Ankrd23 |
| ENSRNOG00000011951 | 205,94 | -0,59 | 0,014815 | Plk2 |
| ENSRNOG00000018292 | 12,03 | -0,70 | 0,015157 |  |
| ENSRNOG00000023509 | 1049,60 | 0,60 | 0,01544 | Irs2 |
| ENSRNOG00000006776 | 1174,25 | 0,49 | 0,015499 | Smyd1 |
| ENSRNOG00000017431 | 422,34 | 0,60 | 0,015615 | RGD1304884 |
| ENSRNOG00000000999 | 458,85 | 0,37 | 0,016749 | Smurf1 |
| ENSRNOG00000011929 | 1042,76 | 0,37 | 0,01675 | Abcd3 |
| ENSRNOG00000046889 | 1059,85 | 0,54 | 0,017032 | Dbi |
| ENSRNOG00000004980 | 619,36 | -0,53 | 0,017032 | Rangrf |
| ENSRNOG00000013793 | 382,03 | -0,54 | 0,01707 | C1qtnf9 |
| ENSRNOG00000025695 | 819,22 | 0,40 | 0,017318 | Tns3 |
| ENSRNOG00000003546 | 445,53 | 0,69 | 0,017412 | Tnfrsf12a |
| ENSRNOG00000018704 | 552,03 | 0,41 | 0,017462 | Nolc1 |
| ENSRNOG00000036835 | 683,76 | -0,39 | 0,017462 | Copz1 |
| ENSRNOG00000027098 | 28,62 | -0,77 | 0,017462 | Sez6l2 |
| ENSRNOG00000009870 | 145,16 | -0,61 | 0,017868 | Tmem88 |
| ENSRNOG00000045670 | 197,16 | -0,50 | 0,017879 | Tnfsf12 |
| ENSRNOG00000016403 | 17,02 | -0,76 | 0,018084 | Olah |
| ENSRNOG00000003170 | 23,78 | -0,75 | 0,018422 | Nlrp3 |
| ENSRNOG00000004621 | 2188,93 | 0,27 | 0,019499 | Rtn4 |
| ENSRNOG00000022946 | 166,58 | 0,63 | 0,019499 | Slc22a3 |
| ENSRNOG00000010777 | 92,93 | 0,69 | 0,019533 | Tox |
| ENSRNOG00000014815 | 10357,86 | -0,32 | 0,019533 | Myoz2 |
| ENSRNOG00000005614 | 1007,01 | -0,51 | 0,020113 | Txn2 |
| ENSRNOG00000019996 | 5724,07 | 0,34 | 0,02022 | Slc16a1 |
| ENSRNOG00000002713 | 321,73 | 0,52 | 0,020755 | Zfp672 |
| ENSRNOG00000001442 | 1699,41 | 0,41 | 0,020755 | Por |
| ENSRNOG00000018069 | 353,70 | -0,62 | 0,020755 | Ngdn |
| ENSRNOG00000005619 | 8,37 | -0,65 | 0,020911 | Misp3 |
| ENSRNOG00000011754 | 40104,22 | 0,34 | 0,021188 | Myom2 |
| ENSRNOG00000005695 | 3037,08 | -0,52 | 0,021222 | Mgp |
| ENSRNOG00000004508 | 617,93 | -0,50 | 0,021222 |  |
| ENSRNOG00000022419 | 213,31 | 0,57 | 0,021584 | Dok7 |
| ENSRNOG00000010633 | 10823,78 | 0,34 | 0,021584 | Acsl1 |
| ENSRNOG00000003357 | 4478,61 | -0,57 | 0,021699 | Col3a1 |
| ENSRNOG00000042229 | 34,25 | -0,74 | 0,02201 | Gimap1 |
| ENSRNOG00000024986 | 127,51 | -0,67 | 0,022215 | Mmrn1 |
| ENSRNOG00000001227 | 474,78 | 0,42 | 0,022527 | Adarb1 |
| ENSRNOG00000046654 | 233,96 | -0,59 | 0,022527 |  |
| ENSRNOG00000020457 | 2914,45 | 0,35 | 0,022954 | Tacc2 |
| ENSRNOG00000001828 | 239,25 | 0,57 | 0,023441 | Stk38l |
| ENSRNOG00000003905 | 213,00 | -0,54 | 0,023676 | Nsf |
| ENSRNOG00000014371 | 511,78 | -0,42 | 0,023676 | Cdh13 |
| ENSRNOG00000018823 | 2463,08 | 0,32 | 0,023676 | Nisch |
| ENSRNOG00000006285 | 461,92 | 0,40 | 0,023899 | Tlk2 |
| ENSRNOG00000013391 | 5803,74 | 0,36 | 0,024194 | Sorbs2 |
| ENSRNOG00000007284 | 114,06 | -0,60 | 0,024278 | Slc2a1 |
| ENSRNOG00000003172 | 435,12 | -0,46 | 0,024414 | Serpinf1 |
| ENSRNOG00000008613 | 582,52 | 0,35 | 0,024815 | Gatad1 |
| ENSRNOG00000014343 | 7,87 | -0,66 | 0,024842 | Anln |
| ENSRNOG00000019372 | 391,22 | -0,44 | 0,024937 | Pc |
| ENSRNOG00000012874 | 381,79 | -0,48 | 0,025103 | Rgma |
| ENSRNOG00000025946 | 24,24 | 0,74 | 0,025381 | Igf2bp2 |
| ENSRNOG00000007326 | 1006,91 | 0,44 | 0,025603 | Prepl |
| ENSRNOG00000015850 | 183,76 | 0,66 | 0,025924 | Rbp7 |
| ENSRNOG00000028064 | 46,03 | 0,73 | 0,025975 | Fhad1 |
| ENSRNOG00000011292 | 879,45 | -0,56 | 0,026357 | Col1a2 |
| ENSRNOG00000017560 | 24,02 | -0,74 | 0,026856 | Mdk |
| ENSRNOG00000014566 | 22,64 | -0,73 | 0,026935 |  |
| ENSRNOG00000015420 | 371,13 | -0,43 | 0,027187 | Stxbp1 |
| ENSRNOG00000003209 | 320,77 | -0,52 | 0,027618 | Pcp4l1 |
| ENSRNOG00000028996 | 7,62 | -0,66 | 0,027824 | Krt1 |
| ENSRNOG00000018110 | 2626,18 | 0,46 | 0,027824 | Svil |
| ENSRNOG00000033389 | 308,33 | -0,54 | 0,027824 | Susd2 |
| ENSRNOG00000021110 | 263,50 | 0,48 | 0,027824 | Mllt11 |
| ENSRNOG00000009538 | 5164,04 | 0,33 | 0,027871 | Etfdh |
| ENSRNOG00000011831 | 368,92 | -0,48 | 0,029968 | Nudt18 |
| ENSRNOG00000019667 | 119,45 | -0,65 | 0,029978 | Ppfibp2 |
| ENSRNOG00000009425 | 23,45 | 0,71 | 0,030315 | Fgf7 |
| ENSRNOG00000001158 | 97,91 | -0,60 | 0,030417 | Abcg1 |
| ENSRNOG00000002828 | 707,63 | 0,40 | 0,030701 | Tob1 |
| ENSRNOG00000016414 | 232,75 | 0,54 | 0,030826 | Slc22a17 |
| ENSRNOG00000022686 | 515,02 | -0,36 | 0,031056 | Zdhhc2 |
| ENSRNOG00000016244 | 1209,20 | 0,39 | 0,03145 | Mical2 |
| ENSRNOG00000003722 | 1072,79 | 0,44 | 0,03145 | Dusp27 |
| ENSRNOG00000026700 | 12727,79 | 0,32 | 0,032119 |  |
| ENSRNOG00000024885 | 201,27 | -0,52 | 0,033926 | Asb10 |
| ENSRNOG00000002171 | 506,08 | 0,55 | 0,036156 |  |
| ENSRNOG00000010708 | 1133,53 | 0,39 | 0,036292 | Gata4 |
| ENSRNOG00000006720 | 373,20 | -0,64 | 0,037233 | Rnpep |
| ENSRNOG00000019807 | 82,41 | 0,57 | 0,037233 | Sufu |
| ENSRNOG00000037645 | 161,77 | 0,66 | 0,03782 | Tceal7 |
| ENSRNOG00000020415 | 586,77 | -0,43 | 0,037909 | Ramp2 |
| ENSRNOG00000046005 | 385,11 | -0,59 | 0,038206 | Scd2 |
| ENSRNOG00000001795 | 1102,75 | 0,31 | 0,038382 |  |
| ENSRNOG00000000955 | 148,95 | 0,54 | 0,038879 | Lnx2 |
| ENSRNOG00000031934 | 1397,41 | 0,49 | 0,038879 | Enah |
| ENSRNOG00000001247 | 1582,92 | 0,52 | 0,039815 | Clip1 |
| ENSRNOG00000018791 | 291,32 | -0,50 | 0,040582 | Dnlz |
| ENSRNOG00000000250 | 310,96 | 0,51 | 0,040986 | Jmjd6 |
| ENSRNOG00000037815 | 877,60 | 0,40 | 0,042364 | Acad10 |
| ENSRNOG00000012658 | 684,88 | 0,42 | 0,042464 | Pdlim3 |
| ENSRNOG00000009756 | 1524,64 | 0,33 | 0,044263 | Pacsin2 |
| ENSRNOG00000006980 | 237,58 | 0,47 | 0,044263 | Vcpip1 |
| ENSRNOG00000021201 | 10733,93 | 0,44 | 0,044263 | Txnip |
| ENSRNOG00000011194 | 815,97 | 0,37 | 0,044702 | Tpp2 |
| ENSRNOG00000030238 | 373,40 | -0,47 | 0,044702 | Fndc5 |
| ENSRNOG00000005729 | 14,44 | -0,68 | 0,044702 | Palm3 |
| ENSRNOG00000022268 | 24,43 | -0,69 | 0,044702 | Pnpla3 |
| ENSRNOG00000019475 | 1407,82 | -0,31 | 0,044702 |  |
| ENSRNOG00000018681 | 39,35 | -0,70 | 0,044702 | Nes |
| ENSRNOG00000009980 | 302,59 | 0,51 | 0,044702 | Plpp1 |
| ENSRNOG00000038625 | 18,07 | -0,66 | 0,044809 | Sbk2 |
| ENSRNOG00000007281 | 7151,90 | 0,41 | 0,044809 | Flnc |
| ENSRNOG00000020956 | 990,01 | -0,36 | 0,045108 | Bcat2 |
| ENSRNOG00000017202 | 6516,17 | 0,34 | 0,045871 |  |
| ENSRNOG00000014011 | 345,64 | -0,48 | 0,04673 | Dll4 |
| ENSRNOG00000007229 | 116,45 | -0,68 | 0,04673 | Nr0b2 |
| ENSRNOG00000007682 | 455,60 | 0,51 | 0,04716 | Gria3 |
| ENSRNOG00000047014 | 699,73 | 0,44 | 0,047264 | Homer1 |
| ENSRNOG00000020030 | 114,46 | -0,62 | 0,048083 | Crlf1 |
| ENSRNOG00000008830 | 3047,84 | 0,30 | 0,048114 | Nfe2l1 |
| ENSRNOG00000006009 | 69,08 | -0,66 | 0,048567 |  |
| ENSRNOG00000017564 | 450,74 | 0,49 | 0,049031 | Mib2 |
| ENSRNOG00000005429 | 28,69 | 0,66 | 0,049366 |  |
| ENSRNOG00000003464 | 112,42 | -0,61 | 0,050022 | Hid1 |
| ENSRNOG00000000081 | 175,96 | 0,59 | 0,050459 | Antxr2 |
| ENSRNOG00000022523 | 1363,62 | 0,42 | 0,051102 | Fkbp5 |
| ENSRNOG00000043094 | 16815,21 | -0,35 | 0,051434 | Oxct1 |
| ENSRNOG00000019662 | 130,62 | -0,56 | 0,051896 | Tm6sf1 |
| ENSRNOG00000048174 | 2207,21 | -0,48 | 0,051896 | Uqcrq |
| ENSRNOG00000004773 | 480,83 | 0,39 | 0,051896 | Yaf2 |
| ENSRNOG00000019622 | 550,75 | -0,48 | 0,052528 | Ackr3 |
| ENSRNOG00000028594 | 608,97 | -0,38 | 0,052528 | Ifnar1 |
| ENSRNOG00000002141 | 264,99 | -0,57 | 0,052528 | Cd200 |
| ENSRNOG00000022069 | 112,21 | 0,51 | 0,052528 |  |
| ENSRNOG00000046891 | 2283,11 | 0,29 | 0,052907 |  |
| ENSRNOG00000032618 | 65,73 | 0,61 | 0,052907 | Mst1r |
| ENSRNOG00000015644 | 499,75 | -0,43 | 0,052907 | Ugcg |
| ENSRNOG00000042978 | 122,95 | -0,55 | 0,053039 | Ncald |
| ENSRNOG00000010161 | 1100,54 | -0,38 | 0,053896 | Myo10 |
| ENSRNOG00000010765 | 3675,42 | 0,24 | 0,054105 | Vcl |
| ENSRNOG00000042419 | 275,66 | -0,43 | 0,054414 | Acyp2 |
| ENSRNOG00000016728 | 46,73 | 0,68 | 0,054414 | Tiam2 |
| ENSRNOG00000033338 | 213,13 | -0,47 | 0,054414 | Gimap6 |
| ENSRNOG00000009354 | 209,64 | -0,55 | 0,054592 | Nrarp |
| ENSRNOG00000001245 | 457,88 | -0,34 | 0,054592 | Pcbp3 |
| ENSRNOG00000001082 | 63,68 | -0,58 | 0,054807 | Abcb9 |
| ENSRNOG00000037204 | 140,92 | -0,53 | 0,057721 | Lyrm9 |
| ENSRNOG00000021166 | 1268,05 | -0,35 | 0,057895 | Ecm1 |
| ENSRNOG00000020696 | 174,84 | -0,47 | 0,057895 | Pmvk |
| ENSRNOG00000018400 | 538,87 | 0,36 | 0,057895 | Golm1 |
| ENSRNOG00000027326 | 433,86 | 0,33 | 0,058085 | Tada2b |
| ENSRNOG00000014616 | 582,29 | 0,42 | 0,058595 | Iars |
| ENSRNOG00000014698 | 12,67 | 0,65 | 0,058699 | Chrnb1 |
| ENSRNOG00000011416 | 63,18 | -0,63 | 0,059346 | Vegfc |
| ENSRNOG00000008492 | 10,49 | -0,61 | 0,05959 | Cfap45 |
| ENSRNOG00000010960 | 1367,47 | 0,33 | 0,05959 | Ankh |
| ENSRNOG00000001825 | 1261,21 | 0,33 | 0,059834 |  |
| ENSRNOG00000049471 | 137,68 | -0,56 | 0,059834 | Steap3 |
| ENSRNOG00000025476 | 285,70 | -0,64 | 0,059834 | Tmem252 |
| ENSRNOG00000017801 | 1923,50 | 0,43 | 0,059834 | Atf4 |
| ENSRNOG00000011503 | 293,50 | 0,47 | 0,06015 | Shb |
| ENSRNOG00000012083 | 374,94 | 0,42 | 0,061938 | St6galnac2 |
| ENSRNOG00000014258 | 43,87 | -0,65 | 0,063425 | Rab32 |
| ENSRNOG00000009099 | 123,77 | 0,48 | 0,063541 |  |
| ENSRNOG00000014688 | 52,17 | 0,66 | 0,063566 |  |
| ENSRNOG00000016338 | 135,00 | -0,49 | 0,0657 | Fam92a1 |
| ENSRNOG00000027455 | 38,53 | -0,63 | 0,065701 | RGD1564804 |
| ENSRNOG00000014597 | 81,33 | -0,61 | 0,066818 | Irs1 |
| ENSRNOG00000020063 | 255,66 | -0,48 | 0,066818 | Nfkbib |
| ENSRNOG00000012721 | 603,27 | 0,41 | 0,067168 | Ednra |
| ENSRNOG00000003832 | 112,35 | -0,59 | 0,069233 | Vash2 |
| ENSRNOG00000019568 | 422,32 | 0,49 | 0,069598 | Jund |
| ENSRNOG00000032487 | 521,48 | -0,41 | 0,069799 | Sts |
| ENSRNOG00000017188 | 462,17 | -0,34 | 0,069895 | Cyp27a1 |
| ENSRNOG00000015417 | 1066,08 | 0,33 | 0,072688 | Kansl3 |
| ENSRNOG00000006110 | 117,52 | 0,54 | 0,07327 | Jph1 |
| ENSRNOG00000017625 | 33,54 | 0,65 | 0,073894 | Htr2b |
| ENSRNOG00000018333 | 613,65 | 0,37 | 0,073894 | Rbm6 |
| ENSRNOG00000020542 | 196,42 | -0,52 | 0,073894 | Krtcap2 |
| ENSRNOG00000006411 | 865,68 | -0,34 | 0,073894 |  |
| ENSRNOG00000008416 | 202,57 | -0,50 | 0,074978 | Gimap5 |
| ENSRNOG00000000924 | 195,74 | 0,59 | 0,07516 | Slc7a1 |
| ENSRNOG00000020533 | 761,90 | -0,29 | 0,07516 | Htra1 |
| ENSRNOG00000011363 | 76,59 | 0,60 | 0,075972 | Napepld |
| ENSRNOG00000016573 | 319,89 | 0,39 | 0,075972 | Dgat2 |
| ENSRNOG00000004819 | 32,37 | -0,65 | 0,075972 | Porcn |
| ENSRNOG00000011714 | 125,32 | -0,46 | 0,078023 | Sat2 |
| ENSRNOG00000005796 | 3562,79 | 0,28 | 0,079512 | Ctnna1 |
| ENSRNOG00000004612 | 4956,32 | 0,30 | 0,079512 | Ppp1cb |
| ENSRNOG00000002229 | 1928,60 | 0,34 | 0,07969 | Adcy5 |
| ENSRNOG00000000465 | 368,00 | -0,40 | 0,07969 | Slc39a7 |
| ENSRNOG00000033330 | 20,14 | -0,65 | 0,07969 |  |
| ENSRNOG00000043031 | 478,71 | 0,46 | 0,07969 | Rd3l |
| ENSRNOG00000021158 | 51,51 | -0,61 | 0,07969 | Nudt22 |
| ENSRNOG00000016367 | 465,55 | 0,41 | 0,081241 | Vasp |
| ENSRNOG00000037085 | 754,85 | 0,42 | 0,081285 | Xirp1 |
| ENSRNOG00000013877 | 94,94 | -0,54 | 0,081367 | Ttc23 |
| ENSRNOG00000001198 | 39,01 | 0,63 | 0,081692 | Fam222a |
| ENSRNOG00000024799 | 637,84 | -0,41 | 0,082195 |  |
| ENSRNOG00000002104 | 360,43 | 0,39 | 0,082286 | Scaf4 |
| ENSRNOG00000032018 | 11,96 | 0,60 | 0,083238 | Tmem200b |
| ENSRNOG00000002218 | 149,01 | 0,48 | 0,083238 | Stbd1 |
| ENSRNOG00000009173 | 204,77 | 0,52 | 0,083238 | Smad6 |
| ENSRNOG00000020616 | 136,73 | -0,57 | 0,084344 | LOC103689986 |
| ENSRNOG00000013764 | 19,80 | -0,64 | 0,085785 | Fam118a |
| ENSRNOG00000019297 | 239,73 | -0,35 | 0,086157 |  |
| ENSRNOG00000006756 | 700,21 | 0,32 | 0,086382 | Maged1 |
| ENSRNOG00000017672 | 310,72 | -0,50 | 0,086384 | Akr1c14 |
| ENSRNOG00000008533 | 140,39 | 0,56 | 0,08672 | Ago2 |
| ENSRNOG00000015233 | 4454,87 | 0,29 | 0,08672 | Etfa |
| ENSRNOG00000008016 | 846,47 | 0,43 | 0,088887 | Ckap4 |
| ENSRNOG00000019749 | 672,68 | 0,36 | 0,089371 | Ube2j2 |
| ENSRNOG00000019390 | 95,03 | -0,62 | 0,089427 | Klhl40 |
| ENSRNOG00000001607 | 996,68 | 0,46 | 0,089648 | Adamts1 |
| ENSRNOG00000030285 | 20,90 | 0,55 | 0,091338 | Epha3 |
| ENSRNOG00000001782 | 534,10 | 0,39 | 0,091518 | Osbpl11 |
| ENSRNOG00000023433 | 457,73 | 0,36 | 0,09221 | Gata6 |
| ENSRNOG00000040108 | 2503,50 | 0,34 | 0,092837 | Cd36 |
| ENSRNOG00000010819 | 224,48 | -0,52 | 0,093477 | Hspa4l |
| ENSRNOG00000020097 | 83,68 | -0,62 | 0,093477 | Inha |
| ENSRNOG00000046202 | 15,54 | -0,60 | 0,093814 | Metrnl |
| ENSRNOG00000015941 | 166,30 | -0,46 | 0,093814 | Fkbp10 |
| ENSRNOG00000034258 | 14433,48 | 0,38 | 0,093814 | Xirp2 |
| ENSRNOG00000019141 | 11,68 | -0,51 | 0,093814 | Ch25h |
| ENSRNOG00000021120 | 174,85 | 0,42 | 0,094652 | Prune |
| ENSRNOG00000018214 | 58,40 | -0,63 | 0,094703 | Bok |
| ENSRNOG00000012723 | 1183,18 | 0,31 | 0,094703 | Trim55 |
| ENSRNOG00000018118 | 349,92 | 0,38 | 0,094703 | Atad3a |
| ENSRNOG00000007587 | 1511,40 | -0,38 | 0,094703 | Tcp11l2 |
| ENSRNOG00000006934 | 100,64 | 0,45 | 0,094789 | Acvr1b |
| ENSRNOG00000011980 | 10,77 | 0,47 | 0,094956 | Lrrc7 |
| ENSRNOG00000027724 | 109,65 | -0,51 | 0,096461 | Plekhf1 |
| ENSRNOG00000006380 | 706,95 | 0,35 | 0,096916 | Srsf6 |
| ENSRNOG00000023991 | 245,33 | -0,47 | 0,097033 | Rab20 |
| ENSRNOG00000007955 | 456,20 | -0,46 | 0,09764 | Timp4 |
| ENSRNOG00000012827 | 593,65 | -0,42 | 0,098054 | Mlf1 |
| ENSRNOG00000001376 | 378,53 | -0,43 | 0,098054 | Mettl7a |
| ENSRNOG00000000805 | 4577,78 | 0,41 | 0,098054 | Gja1 |
| ENSRNOG00000019140 | 182,12 | 0,47 | 0,098331 | Banp |
| ENSRNOG00000000190 | 54,84 | 0,60 | 0,099313 |  |
| ENSRNOG00000001455 | 1587,22 | 0,30 | 0,099313 | Kif13a |

**Supplemental table 3:** Differentially expressed genes in obese + DS versus lean + DS rats.

| **ID** | **baseMean** | **log2FoldChange** | **FDR** | **symbol** |
| --- | --- | --- | --- | --- |
| ENSRNOG00000025757 | 7000,90 | -1,36 | 8,73E-11 | Myh6 |
| ENSRNOG00000006305 | 305,98 | 0,82 | 3,53E-08 | Slc38a2 |
| ENSRNOG00000023465 | 91,55 | -1,45 | 5,78E-08 | LOC500300 |
| ENSRNOG00000016151 | 153,19 | 1,13 | 3,97E-07 | Ankrd23 |
| ENSRNOG00000002256 | 194,46 | -0,84 | 2,92E-06 | Art3 |
| ENSRNOG00000007290 | 118,78 | -1,00 | 6,85E-06 | Atp1a2 |
| ENSRNOG00000023257 | 74,81 | 1,07 | 8,95E-06 | Adamts9 |
| ENSRNOG00000034258 | 2878,44 | 1,05 | 1,39E-05 | Xirp2 |
| ENSRNOG00000018794 | 33,24 | -1,31 | 4,14E-05 |  |
| ENSRNOG00000021267 | 129,06 | -0,86 | 4,14E-05 |  |
| ENSRNOG00000017226 | 224,51 | -0,72 | 4,14E-05 | Slc2a4 |
| ENSRNOG00000011648 | 647,62 | -0,78 | 5,1E-05 | Aqp1 |
| ENSRNOG00000010161 | 106,94 | -1,00 | 6,56E-05 | Myo10 |
| ENSRNOG00000010881 | 304,46 | 0,63 | 6,56E-05 | Trak2 |
| ENSRNOG00000010134 | 152,16 | 0,80 | 0,000176 | Acot2 |
| ENSRNOG00000009741 | 50,08 | -1,16 | 0,00018 | Cyp4a3 |
| ENSRNOG00000008236 | 159,98 | 0,78 | 0,00018 | Decr1 |
| ENSRNOG00000019018 | 183,51 | 0,74 | 0,000235 | Plat |
| ENSRNOG00000012966 | 1518,59 | 0,39 | 0,000278 | Acadl |
| ENSRNOG00000013766 | 759,59 | 0,53 | 0,000383 | Acaa2 |
| ENSRNOG00000000875 | 413,47 | 0,52 | 0,000385 | Fhl1 |
| ENSRNOG00000009845 | 894,17 | 0,48 | 0,00082 | Acadm |
| ENSRNOG00000006694 | 340,57 | -0,62 | 0,000821 |  |
| ENSRNOG00000001979 | 455,65 | 0,56 | 0,001053 | Rcan1 |
| ENSRNOG00000005513 | 57,77 | -0,90 | 0,001053 | Srsf5 |
| ENSRNOG00000020955 | 4525,44 | -0,35 | 0,001082 | Myl3 |
| ENSRNOG00000016326 | 56,50 | -0,96 | 0,001932 | Cx3cl1 |
| ENSRNOG00000050655 | 50,54 | 1,00 | 0,001942 | P4ha1 |
| ENSRNOG00000033261 | 97,28 | -0,88 | 0,002115 | Fam107a |
| ENSRNOG00000026415 | 95,53 | 0,73 | 0,002237 | Col14a1 |
| ENSRNOG00000013391 | 641,47 | 0,56 | 0,002487 | Sorbs2 |
| ENSRNOG00000005614 | 50,11 | -0,86 | 0,002644 | Txn2 |
| ENSRNOG00000023122 | 79,54 | -0,75 | 0,003037 | Apol11a |
| ENSRNOG00000017428 | 177,23 | 0,72 | 0,003678 | Map1b |
| ENSRNOG00000029886 | 578,02 | 0,98 | 0,004279 | Hba1 |
| ENSRNOG00000047708 | 33,94 | -0,96 | 0,004297 | Gstz1 |
| ENSRNOG00000000665 | 137,44 | 0,54 | 0,005915 | Pitpnb |
| ENSRNOG00000020068 | 40,06 | -0,86 | 0,006368 | Ndufaf3 |
| ENSRNOG00000016855 | 72,23 | 0,71 | 0,006548 | B3galnt2 |
| ENSRNOG00000017466 | 351,28 | 0,55 | 0,007185 | Kif5b |
| ENSRNOG00000017801 | 164,85 | 0,55 | 0,007185 | Atf4 |
| ENSRNOG00000014230 | 339,02 | 0,48 | 0,007311 | Map1a |
| ENSRNOG00000031479 | 307,37 | 0,41 | 0,007311 | Hdlbp |
| ENSRNOG00000031934 | 146,13 | 0,73 | 0,008158 | Enah |
| ENSRNOG00000020788 | 225,82 | 0,50 | 0,008696 |  |
| ENSRNOG00000026548 | 92,39 | -0,70 | 0,009042 | Dhrs7c |
| ENSRNOG00000007628 | 142,97 | -0,58 | 0,009111 | Ptp4a3 |
| ENSRNOG00000014030 | 120,74 | 0,67 | 0,010167 | Synm |
| ENSRNOG00000007235 | 367,11 | -0,40 | 0,010719 | Atp5g1 |
| ENSRNOG00000016243 | 1341,89 | 0,37 | 0,012017 | Casq2 |
| ENSRNOG00000003078 | 147,71 | 0,61 | 0,012306 | Dcaf6 |
| ENSRNOG00000009686 | 54,21 | 0,76 | 0,012412 | Aqp7 |
| ENSRNOG00000026119 | 311,41 | 0,51 | 0,014266 |  |
| ENSRNOG00000024212 | 41,89 | 0,80 | 0,015225 | Papd5 |
| ENSRNOG00000016021 | 136,99 | -0,57 | 0,016064 | Lims2 |
| ENSRNOG00000023360 | 42,46 | -0,80 | 0,019732 | Fus |
| ENSRNOG00000047321 | 270,85 | 0,90 | 0,019732 | Hba2 |
| ENSRNOG00000015547 | 41,49 | 0,74 | 0,020017 |  |
| ENSRNOG00000011949 | 313,26 | -0,40 | 0,020017 | Ndufb5 |
| ENSRNOG00000012466 | 73,76 | 0,67 | 0,020029 |  |
| ENSRNOG00000008645 | 59,60 | -0,83 | 0,020029 |  |
| ENSRNOG00000016244 | 98,34 | 0,62 | 0,020634 | Mical2 |
| ENSRNOG00000049424 | 180,40 | 0,88 | 0,020939 |  |
| ENSRNOG00000020619 | 70,00 | -0,62 | 0,023149 | Eif3g |
| ENSRNOG00000020748 | 595,14 | 0,29 | 0,023235 | Map4 |
| ENSRNOG00000002208 | 106,84 | 0,60 | 0,023398 | Shroom3 |
| ENSRNOG00000007034 | 310,62 | 0,44 | 0,023398 | Hipk2 |
| ENSRNOG00000016434 | 39,22 | -0,83 | 0,024192 | Prkd2 |
| ENSRNOG00000014371 | 57,93 | -0,64 | 0,02422 | Cdh13 |
| ENSRNOG00000018359 | 42,15 | 0,79 | 0,024917 | Smad7 |
| ENSRNOG00000004078 | 239,05 | -0,52 | 0,026546 | Eno3 |
| ENSRNOG00000029042 | 1889,41 | -0,45 | 0,026563 | ND6 |
| ENSRNOG00000020719 | 931,68 | -0,30 | 0,028515 | Hrc |
| ENSRNOG00000019342 | 58,91 | -0,77 | 0,028868 | Sult1a1 |
| ENSRNOG00000024568 | 117,02 | -0,61 | 0,033496 | Ndufs7 |
| ENSRNOG00000007587 | 161,12 | -0,59 | 0,034469 | Tcp11l2 |
| ENSRNOG00000001055 | 192,75 | -0,43 | 0,035497 | Rilpl1 |
| ENSRNOG00000017786 | 1973,90 | 0,66 | 0,03584 | Acta1 |
| ENSRNOG00000008144 | 47,48 | -0,80 | 0,036811 | Irf1 |
| ENSRNOG00000014215 | 86,02 | -0,57 | 0,037343 | Klf9 |
| ENSRNOG00000003098 | 35,86 | -0,82 | 0,038885 | Prom1 |
| ENSRNOG00000019851 | 1320,64 | -0,33 | 0,040683 | Cox6a2 |
| ENSRNOG00000000498 | 102,50 | -0,60 | 0,041734 | Anks1a |
| ENSRNOG00000014641 | 194,15 | -0,52 | 0,045385 | Rpl3l |
| ENSRNOG00000009628 | 380,93 | -0,39 | 0,045385 |  |
| ENSRNOG00000043391 | 263,29 | -0,43 | 0,047104 |  |
| ENSRNOG00000025476 | 46,66 | -0,74 | 0,047766 | Tmem252 |
| ENSRNOG00000004496 | 340,82 | 0,44 | 0,047903 | Rock2 |
| ENSRNOG00000031396 | 142,41 | 0,47 | 0,048209 |  |
| ENSRNOG00000012524 | 240,32 | 0,44 | 0,048553 | Zfp91 |
| ENSRNOG00000001607 | 90,34 | 0,54 | 0,049325 | Adamts1 |
| ENSRNOG00000013532 | 312,06 | -0,41 | 0,051423 | Pgam2 |
| ENSRNOG00000016714 | 894,96 | 0,28 | 0,053676 | Nrap |
| ENSRNOG00000014288 | 204,08 | 0,76 | 0,054982 | Fn1 |
| ENSRNOG00000046889 | 71,75 | 0,55 | 0,054982 | Dbi |
| ENSRNOG00000018642 | 42,63 | -0,79 | 0,054982 | Leng8 |
| ENSRNOG00000023509 | 85,19 | 0,62 | 0,05546 | Irs2 |
| ENSRNOG00000007060 | 65,52 | 0,51 | 0,05546 | Plin2 |
| ENSRNOG00000013928 | 1498,08 | 0,25 | 0,059044 | Dsp |
| ENSRNOG00000005802 | 152,52 | 0,54 | 0,059044 | Usp24 |
| ENSRNOG00000007726 | 119,94 | 0,54 | 0,060761 | Mcam |
| ENSRNOG00000016692 | 293,04 | 0,41 | 0,064972 | Hsdl2 |
| ENSRNOG00000042771 | 36,90 | -0,75 | 0,066232 | Apol3 |
| ENSRNOG00000018524 | 45,37 | 0,71 | 0,067477 | Ezr |
| ENSRNOG00000018145 | 300,63 | 0,46 | 0,0704 | Crat |
| ENSRNOG00000014509 | 96,14 | 0,55 | 0,07136 | Sacs |
| ENSRNOG00000016448 | 42,08 | -0,63 | 0,074431 | Eef2k |
| ENSRNOG00000007682 | 48,94 | 0,71 | 0,074929 | Gria3 |
| ENSRNOG00000001766 | 49,82 | 0,74 | 0,075101 | Tfrc |
| ENSRNOG00000050585 | 150,69 | 0,40 | 0,075306 | Pgam1 |
| ENSRNOG00000018250 | 3709,03 | -0,30 | 0,075306 | Tnni3 |
| ENSRNOG00000013793 | 53,81 | -0,69 | 0,078402 | C1qtnf9 |
| ENSRNOG00000020679 | 59,47 | -0,86 | 0,078718 | Icam1 |
| ENSRNOG00000000170 | 43,86 | 0,62 | 0,079493 | Slc30a4 |
| ENSRNOG00000002225 | 50,97 | 0,63 | 0,079493 | Scarb2 |
| ENSRNOG00000019791 | 49,96 | 0,57 | 0,081461 | Sipa1l2 |
| ENSRNOG00000018630 | 755,67 | -0,28 | 0,081461 | Gapdh |
| ENSRNOG00000020173 | 81,25 | -0,64 | 0,081461 | Tie1 |
| ENSRNOG00000029336 | 49,95 | 0,58 | 0,081461 | Zfp180 |
| ENSRNOG00000007518 | 178,55 | 0,40 | 0,08252 | Nckap1 |
| ENSRNOG00000001825 | 88,61 | 0,51 | 0,08252 |  |
| ENSRNOG00000007102 | 167,92 | -0,46 | 0,083638 | Acss1 |
| ENSRNOG00000018698 | 60,16 | 0,61 | 0,083638 | Wac |
| ENSRNOG00000019996 | 396,84 | 0,33 | 0,085819 | Slc16a1 |
| ENSRNOG00000008830 | 223,54 | 0,39 | 0,088287 | Nfe2l1 |
| ENSRNOG00000017672 | 45,26 | -0,63 | 0,091576 | Akr1c14 |
| ENSRNOG00000019094 | 214,44 | -0,45 | 0,091947 |  |
| ENSRNOG00000016588 | 197,16 | -0,35 | 0,091947 |  |
| ENSRNOG00000024849 | 52,27 | 0,62 | 0,091947 | Tor1aip2 |
| ENSRNOG00000005632 | 46,39 | 0,58 | 0,092913 |  |
| ENSRNOG00000018708 | 119,44 | -0,42 | 0,093623 | Ppp1ca |
| ENSRNOG00000031230 | 42,89 | 0,82 | 0,093623 |  |
| ENSRNOG00000018975 | 83,62 | 0,54 | 0,093623 | Atg9a |
| ENSRNOG00000013949 | 1499,15 | -0,24 | 0,093623 | Idh2 |
| ENSRNOG00000016660 | 516,08 | -0,36 | 0,094252 | Cox5b |
| ENSRNOG00000003403 | 91,52 | 0,54 | 0,094252 | Slc35f5 |
| ENSRNOG00000011154 | 423,80 | -0,44 | 0,096181 | Adgrf5 |
| ENSRNOG00000021157 | 39,71 | 0,59 | 0,097265 |  |
| ENSRNOG00000001827 | 45,28 | -0,64 | 0,097265 | Masp1 |
| ENSRNOG00000008414 | 353,82 | -0,33 | 0,097265 | Bsg |
| ENSRNOG00000001142 | 48,00 | -0,65 | 0,097304 | Prkab1 |
| ENSRNOG00000019213 | 131,21 | 0,45 | 0,097585 |  |
| ENSRNOG00000005389 | 67,44 | 0,51 | 0,097585 | Ppp2ca |
| ENSRNOG00000015153 | 50,97 | 0,56 | 0,097585 | Ints3 |
| ENSRNOG00000016831 | 206,26 | 0,42 | 0,097585 | Serpinh1 |
| ENSRNOG00000017414 | 33,24 | -0,69 | 0,099049 | Irf7 |

**Supplemental table 4:** Differentially expressed genes in lean + DS versus lean rats.

| **ID** | **baseMean** | **log2FoldChange** | **FDR** | **symbol** |
| --- | --- | --- | --- | --- |
| ENSRNOG00000048351 | 5213,85 | 1,20 | 5,32E-14 |  |
| ENSRNOG00000021434 | 5,69 | 2,31 | 7,7E-12 | Usp51 |
| ENSRNOG00000032274 | 222,29 | 1,83 | 7,88E-12 |  |
| ENSRNOG00000048730 | 27,56 | 1,90 | 3,66E-10 |  |
| ENSRNOG00000008170 | 1186,68 | -0,64 | 6,32E-10 | Jph2 |
| ENSRNOG00000031506 | 7,53 | 2,08 | 2,75E-09 | LOC100360087 |
| ENSRNOG00000014153 | 197,74 | 0,77 | 2,9E-09 | Lhfp |
| ENSRNOG00000014573 | 61,18 | -1,55 | 4,31E-09 | Ckmt1b |
| ENSRNOG00000001966 | 229,67 | 0,99 | 4,31E-09 |  |
| ENSRNOG00000001271 | 134,19 | 1,06 | 4,31E-09 | Card6 |
| ENSRNOG00000007637 | 217,48 | 1,36 | 1,13E-08 | Acer2 |
| ENSRNOG00000001177 | 801,00 | -0,69 | 2,55E-08 | Acads |
| ENSRNOG00000022196 | 312,15 | 0,88 | 4,46E-08 | Bmpr2 |
| ENSRNOG00000015488 | 128,11 | 1,08 | 4,46E-08 | Tead1 |
| ENSRNOG00000022637 | 7358,47 | 0,95 | 1,24E-07 |  |
| ENSRNOG00000007034 | 651,20 | 0,79 | 1,43E-07 | Hipk2 |
| ENSRNOG00000015753 | 252,45 | -0,69 | 1,63E-07 | Epn1 |
| ENSRNOG00000026511 | 47,06 | 1,58 | 1,76E-07 |  |
| ENSRNOG00000031685 | 483,77 | -1,32 | 2,19E-07 |  |
| ENSRNOG00000008602 | 211,97 | 0,78 | 3,71E-07 | Steap4 |
| ENSRNOG00000046608 | 6,99 | 1,76 | 7,8E-07 |  |
| ENSRNOG00000030478 | 755976,73 | 1,57 | 1,5E-06 |  |
| ENSRNOG00000018666 | 587,58 | -0,70 | 1,71E-06 | Gpsm1 |
| ENSRNOG00000011015 | 144,69 | 0,82 | 1,71E-06 | Hivep2 |
| ENSRNOG00000005126 | 17,84 | 1,48 | 1,71E-06 | Pqlc3 |
| ENSRNOG00000012307 | 7821,24 | -0,37 | 2,35E-06 | Mybpc3 |
| ENSRNOG00000043114 | 119,40 | -0,98 | 5,93E-06 | Tomm7 |
| ENSRNOG00000047746 | 13,84 | 1,62 | 6,28E-06 |  |
| ENSRNOG00000031439 | 81,04 | -1,03 | 6,61E-06 |  |
| ENSRNOG00000018145 | 981,58 | -0,61 | 9,87E-06 | Crat |
| ENSRNOG00000023411 | 29,98 | -1,36 | 1E-05 | Vsig10l |
| ENSRNOG00000009513 | 574,61 | -0,65 | 1E-05 | Akr1b1 |
| ENSRNOG00000014275 | 77,47 | 0,89 | 1,19E-05 |  |
| ENSRNOG00000028834 | 279,88 | 0,78 | 1,28E-05 | Polr2a |
| ENSRNOG00000031205 | 11,73 | 1,52 | 1,81E-05 |  |
| ENSRNOG00000048658 | 8,47 | 1,55 | 1,97E-05 |  |
| ENSRNOG00000033169 | 169,96 | 0,96 | 1,98E-05 | Cpeb4 |
| ENSRNOG00000011782 | 1944,62 | -0,50 | 2,19E-05 | Got2 |
| ENSRNOG00000021808 | 1492,94 | -0,56 | 2,23E-05 | Tecr |
| ENSRNOG00000017823 | 56,03 | 0,97 | 2,47E-05 |  |
| ENSRNOG00000033299 | 4226,40 | 1,40 | 2,67E-05 | ATP8 |
| ENSRNOG00000020788 | 703,70 | -0,54 | 2,72E-05 |  |
| ENSRNOG00000015692 | 190,34 | 0,91 | 2,84E-05 | Taok1 |
| ENSRNOG00000018251 | 190,10 | 0,82 | 3,03E-05 | Mrc1 |
| ENSRNOG00000015385 | 418,48 | -0,70 | 3,03E-05 | Pink1 |
| ENSRNOG00000017866 | 194,18 | -0,65 | 3,2E-05 | Sirt5 |
| ENSRNOG00000048723 | 106,17 | 0,97 | 4,08E-05 | Pros1 |
| ENSRNOG00000009614 | 19,53 | 1,50 | 4,61E-05 | LOC300249 |
| ENSRNOG00000009628 | 1627,77 | -0,50 | 6,21E-05 |  |
| ENSRNOG00000021261 | 15,82 | 1,32 | 7,55E-05 | Rassf2 |
| ENSRNOG00000017440 | 727,46 | 0,99 | 8,45E-05 |  |
| ENSRNOG00000017672 | 141,01 | 0,76 | 9,64E-05 | Akr1c14 |
| ENSRNOG00000030109 | 22,90 | 1,37 | 0,0001 | Kpna5 |
| ENSRNOG00000013793 | 173,62 | 0,76 | 0,0001 | C1qtnf9 |
| ENSRNOG00000017295 | 51,12 | -1,05 | 0,0001 | Sh3glb2 |
| ENSRNOG00000008639 | 713,66 | 0,62 | 0,000104 | Pabpc1 |
| ENSRNOG00000016665 | 67,01 | -0,97 | 0,000104 | Tpra1 |
| ENSRNOG00000049437 | 1288,13 | -0,47 | 0,000112 | Gpc1 |
| ENSRNOG00000015957 | 83,59 | 1,03 | 0,000119 | F13a1 |
| ENSRNOG00000014999 | 108,64 | 0,74 | 0,000119 | Tnpo1 |
| ENSRNOG00000003841 | 3,83 | 1,36 | 0,00012 | Kcnh1 |
| ENSRNOG00000032134 | 2435,16 | -0,49 | 0,00012 | Uqcrc1 |
| ENSRNOG00000033615 | 29965,67 | 0,68 | 0,000158 | ND3 |
| ENSRNOG00000029212 | 152,91 | 1,17 | 0,00016 | Vcan |
| ENSRNOG00000021100 | 18,12 | 1,29 | 0,00018 | Tnfaip8l2 |
| ENSRNOG00000033134 | 69,68 | 0,90 | 0,000181 | Mef2c |
| ENSRNOG00000019810 | 5965,86 | -0,46 | 0,000196 | Des |
| ENSRNOG00000007235 | 1678,95 | -0,64 | 0,000227 | Atp5g1 |
| ENSRNOG00000016866 | 1639,40 | -0,64 | 0,000227 | Fhl2 |
| ENSRNOG00000043451 | 58,61 | 1,39 | 0,000247 | Spp1 |
| ENSRNOG00000043866 | 701814,26 | 1,28 | 0,000248 |  |
| ENSRNOG00000028674 | 320,55 | -0,56 | 0,000248 | Fbxw5 |
| ENSRNOG00000015093 | 1425,36 | 0,52 | 0,000248 | Sparcl1 |
| ENSRNOG00000030715 | 257,11 | 0,74 | 0,00025 | Cfh |
| ENSRNOG00000030954 | 548,51 | 0,70 | 0,00026 | Fat1 |
| ENSRNOG00000012477 | 1779,73 | -0,53 | 0,000271 | Eef1a2 |
| ENSRNOG00000000380 | 43,49 | 0,93 | 0,000271 |  |
| ENSRNOG00000020747 | 43,36 | -1,00 | 0,000332 | Nkx2-5 |
| ENSRNOG00000009956 | 972,78 | 0,49 | 0,000361 | Wnk1 |
| ENSRNOG00000032882 | 36,40 | 1,17 | 0,000367 |  |
| ENSRNOG00000002877 | 657,03 | 0,63 | 0,000374 |  |
| ENSRNOG00000018359 | 129,63 | -0,87 | 0,00038 | Smad7 |
| ENSRNOG00000020607 | 652,56 | -0,53 | 0,000403 | Bckdha |
| ENSRNOG00000015505 | 62,99 | 0,85 | 0,00044 | Mfap5 |
| ENSRNOG00000045821 | 109,88 | -0,83 | 0,00044 | Slc41a3 |
| ENSRNOG00000006839 | 121,83 | 0,61 | 0,000443 | Arl5a |
| ENSRNOG00000042920 | 46,26 | -1,05 | 0,000448 |  |
| ENSRNOG00000002898 | 24,33 | 1,11 | 0,000525 | Nme7 |
| ENSRNOG00000012443 | 493,05 | -0,65 | 0,000533 | Cpt2 |
| ENSRNOG00000042519 | 172,99 | 0,79 | 0,000533 | Peak1 |
| ENSRNOG00000013927 | 58,35 | -1,05 | 0,000533 | Jag2 |
| ENSRNOG00000012457 | 1653,58 | -0,54 | 0,000533 | Cyc1 |
| ENSRNOG00000018848 | 34,41 | -1,14 | 0,000541 | Eif1b |
| ENSRNOG00000026902 | 209,27 | 0,79 | 0,000544 | Lyve1 |
| ENSRNOG00000043201 | 877,13 | -0,51 | 0,000549 | Coq8a |
| ENSRNOG00000025295 | 249,26 | -0,56 | 0,000556 | Mavs |
| ENSRNOG00000032701 | 330,09 | -0,51 | 0,000556 |  |
| ENSRNOG00000011137 | 32,78 | 0,97 | 0,000556 | Zbtb41 |
| ENSRNOG00000009795 | 139,56 | 0,82 | 0,000578 | Nfib |
| ENSRNOG00000020456 | 220,48 | 0,67 | 0,000616 | Nucb2 |
| ENSRNOG00000010488 | 513,75 | 0,45 | 0,000616 | Zmiz1 |
| ENSRNOG00000016837 | 5610,92 | -0,52 | 0,000631 | Ckm |
| ENSRNOG00000001211 | 1052,71 | -0,57 | 0,000638 | RGD1303003 |
| ENSRNOG00000002050 | 888,52 | 0,54 | 0,000656 | Igfbp7 |
| ENSRNOG00000010529 | 92,27 | 0,80 | 0,000656 | Thbs2 |
| ENSRNOG00000002515 | 22,67 | 1,19 | 0,000734 |  |
| ENSRNOG00000011864 | 128,26 | 0,67 | 0,000751 |  |
| ENSRNOG00000013331 | 1985,00 | -0,43 | 0,00078 | Sdha |
| ENSRNOG00000001736 | 132,22 | -0,61 | 0,000785 | Bdh1 |
| ENSRNOG00000021812 | 33,77 | -1,03 | 0,000792 | Scx |
| ENSRNOG00000009248 | 244,54 | 0,60 | 0,000792 | Pnrc2 |
| ENSRNOG00000002177 | 46,93 | 0,98 | 0,000792 | Gnpda2 |
| ENSRNOG00000009196 | 86,10 | 0,85 | 0,0008 | Rc3h2 |
| ENSRNOG00000002194 | 81,26 | -0,88 | 0,000811 | Coq2 |
| ENSRNOG00000021139 | 181,89 | -0,64 | 0,000811 | Esrra |
| ENSRNOG00000006485 | 96,32 | 0,86 | 0,000811 | Topors |
| ENSRNOG00000018553 | 48,83 | -0,94 | 0,000837 | Pitpnm1 |
| ENSRNOG00000017250 | 448,93 | -0,53 | 0,000837 | Gmpr |
| ENSRNOG00000006965 | 313,61 | 0,62 | 0,000837 | Aff4 |
| ENSRNOG00000000190 | 26,92 | 1,26 | 0,000837 |  |
| ENSRNOG00000018630 | 3163,32 | -0,53 | 0,000875 | Gapdh |
| ENSRNOG00000034303 | 78,12 | 0,78 | 0,000875 |  |
| ENSRNOG00000006593 | 249,37 | -0,62 | 0,000875 | Grpel1 |
| ENSRNOG00000015553 | 70,61 | 0,77 | 0,000875 | Gatad2b |
| ENSRNOG00000019506 | 257,24 | -0,56 | 0,000875 | Dnajb2 |
| ENSRNOG00000008904 | 76,40 | 0,78 | 0,000875 | Fli1 |
| ENSRNOG00000028323 | 38,97 | -1,14 | 0,000875 | Dhrsx |
| ENSRNOG00000009012 | 545,96 | -0,54 | 0,000891 |  |
| ENSRNOG00000006995 | 197,75 | 0,56 | 0,000903 | Ano6 |
| ENSRNOG00000009992 | 117,31 | -0,62 | 0,000955 | Rabggtb |
| ENSRNOG00000012709 | 56,56 | -0,92 | 0,000955 | Bag2 |
| ENSRNOG00000046698 | 119,04 | -0,66 | 0,000968 | Gps1 |
| ENSRNOG00000047147 | 120,64 | 0,86 | 0,001013 |  |
| ENSRNOG00000029095 | 100,16 | -0,79 | 0,001013 | Trabd |
| ENSRNOG00000014395 | 14,94 | 1,27 | 0,001061 | Gli3 |
| ENSRNOG00000012779 | 14,42 | 1,24 | 0,001066 | Msr1 |
| ENSRNOG00000018816 | 1076,71 | -0,53 | 0,001066 | Cox5a |
| ENSRNOG00000024239 | 71,60 | -0,88 | 0,001106 | Fam89b |
| ENSRNOG00000019772 | 234,07 | -0,67 | 0,001106 | Dnpep |
| ENSRNOG00000021029 | 17,99 | 1,18 | 0,001123 | Hamp |
| ENSRNOG00000045829 | 294,32 | 1,23 | 0,001126 | Thbs1 |
| ENSRNOG00000029490 | 86,03 | -0,74 | 0,001126 | Znf768 |
| ENSRNOG00000021553 | 24,14 | 1,07 | 0,001127 | Nckap5 |
| ENSRNOG00000012516 | 1525,67 | 0,63 | 0,001141 |  |
| ENSRNOG00000019211 | 26,21 | 1,02 | 0,001179 | Olfml3 |
| ENSRNOG00000006375 | 1245,47 | -0,32 | 0,001209 | Vdac1 |
| ENSRNOG00000011908 | 49,83 | 0,97 | 0,001227 | Asxl2 |
| ENSRNOG00000022946 | 20,28 | -1,18 | 0,001229 | Slc22a3 |
| ENSRNOG00000020466 | 67,22 | -0,89 | 0,001229 | Apba3 |
| ENSRNOG00000009585 | 122,15 | 0,71 | 0,001229 | Tcf20 |
| ENSRNOG00000007706 | 232,79 | 0,80 | 0,001229 | Prkaa2 |
| ENSRNOG00000004622 | 103,32 | 0,73 | 0,001229 |  |
| ENSRNOG00000019522 | 108,10 | -0,68 | 0,00123 | Narfl |
| ENSRNOG00000050727 | 133,56 | 0,91 | 0,001315 |  |
| ENSRNOG00000029971 | 96362,18 | 0,88 | 0,001367 | ND5 |
| ENSRNOG00000009252 | 482,44 | -0,60 | 0,001384 |  |
| ENSRNOG00000011504 | 483,60 | 0,57 | 0,001423 | Akap2 |
| ENSRNOG00000017032 | 7849,46 | -0,40 | 0,001435 | Atp5a1 |
| ENSRNOG00000020097 | 18,30 | -1,19 | 0,00148 | Inha |
| ENSRNOG00000015233 | 923,62 | -0,38 | 0,001493 | Etfa |
| ENSRNOG00000010259 | 9,97 | -1,23 | 0,001502 | Esrrb |
| ENSRNOG00000017087 | 182,75 | -0,65 | 0,001516 | Man1c1 |
| ENSRNOG00000017469 | 182,72 | 0,64 | 0,001563 | Anxa1 |
| ENSRNOG00000029571 | 233,16 | -0,56 | 0,001567 | Coq10a |
| ENSRNOG00000014371 | 194,88 | 0,50 | 0,001569 | Cdh13 |
| ENSRNOG00000018117 | 2076,13 | -0,42 | 0,001579 | Ndufv1 |
| ENSRNOG00000046848 | 71,69 | 0,93 | 0,001588 | PCOLCE2 |
| ENSRNOG00000002075 | 92,83 | 0,79 | 0,001588 | Cnot6l |
| ENSRNOG00000025890 | 242,11 | -0,54 | 0,001595 | Opa3 |
| ENSRNOG00000004464 | 129,10 | 0,74 | 0,001652 | Sel1l |
| ENSRNOG00000014289 | 392,96 | 0,37 | 0,001659 | Arpc2 |
| ENSRNOG00000007302 | 225,09 | 1,02 | 0,001675 | Fbn1 |
| ENSRNOG00000048169 | 477,74 | -0,60 | 0,001675 | Tuba8 |
| ENSRNOG00000048057 | 1302,26 | 0,55 | 0,001702 |  |
| ENSRNOG00000010208 | 63,01 | 1,04 | 0,001702 | Timp1 |
| ENSRNOG00000020373 | 86,26 | -0,69 | 0,001718 | Dap3 |
| ENSRNOG00000003553 | 88,23 | 0,67 | 0,001723 | Efemp1 |
| ENSRNOG00000003882 | 120,18 | 0,85 | 0,001739 | Cep350 |
| ENSRNOG00000014270 | 128,34 | -0,62 | 0,001739 | Rer1 |
| ENSRNOG00000005809 | 106,18 | 0,67 | 0,001805 | Arhgdib |
| ENSRNOG00000002833 | 76,75 | 0,80 | 0,001806 | Gsk3b |
| ENSRNOG00000047653 | 9,38 | -1,23 | 0,001814 | Crybb1 |
| ENSRNOG00000024737 | 233,24 | 0,67 | 0,001819 | Tnrc6a |
| ENSRNOG00000016892 | 84,36 | -0,89 | 0,001822 | Nr2f6 |
| ENSRNOG00000001235 | 536,99 | -0,43 | 0,001845 | Gna12 |
| ENSRNOG00000020300 | 67,99 | 0,93 | 0,001882 | Lsp1 |
| ENSRNOG00000006690 | 74,91 | 0,67 | 0,001882 |  |
| ENSRNOG00000018556 | 85,78 | -0,88 | 0,001898 | Tomm40 |
| ENSRNOG00000003261 | 654,92 | 0,45 | 0,001944 | Usp9x |
| ENSRNOG00000007290 | 631,96 | -0,57 | 0,001989 | Atp1a2 |
| ENSRNOG00000012938 | 120,68 | -0,76 | 0,001989 |  |
| ENSRNOG00000001989 | 30,11 | 1,12 | 0,001989 | Alcam |
| ENSRNOG00000046996 | 338,32 | 0,51 | 0,001989 | Pea15 |
| ENSRNOG00000012999 | 260,29 | -0,52 | 0,002 | Phb2 |
| ENSRNOG00000020138 | 266,11 | -0,56 | 0,002048 | Slc4a3 |
| ENSRNOG00000012563 | 175,01 | 0,73 | 0,002066 | Arhgap29 |
| ENSRNOG00000000277 | 11,98 | 1,17 | 0,002131 | Tet1 |
| ENSRNOG00000005264 | 113,25 | 0,70 | 0,002131 | Sav1 |
| ENSRNOG00000011343 | 2108,94 | -0,37 | 0,002131 |  |
| ENSRNOG00000002565 | 113,74 | 0,64 | 0,002149 |  |
| ENSRNOG00000029876 | 15,51 | 1,17 | 0,002155 | Gucy1a2 |
| ENSRNOG00000019804 | 80,41 | -0,78 | 0,002159 | B3gat3 |
| ENSRNOG00000014522 | 332,37 | -0,68 | 0,002159 | Mlycd |
| ENSRNOG00000046170 | 66,48 | 1,04 | 0,002171 |  |
| ENSRNOG00000046763 | 434,74 | -0,61 | 0,002206 | Adssl1 |
| ENSRNOG00000014288 | 311,49 | 0,99 | 0,002307 | Fn1 |
| ENSRNOG00000020915 | 337,71 | 0,55 | 0,002307 | Setd2 |
| ENSRNOG00000006420 | 274,18 | -0,49 | 0,002307 | Rbm38 |
| ENSRNOG00000017438 | 55,32 | 0,87 | 0,002354 |  |
| ENSRNOG00000018454 | 427,44 | 0,77 | 0,00241 | Apoe |
| ENSRNOG00000024128 | 6214,14 | -0,40 | 0,00241 | Aco2 |
| ENSRNOG00000008626 | 52,23 | 0,77 | 0,002489 | Manea |
| ENSRNOG00000037500 | 30,30 | -0,94 | 0,002592 | Pus1 |
| ENSRNOG00000004841 | 790,39 | 0,60 | 0,002694 | Akap6 |
| ENSRNOG00000019740 | 65,94 | 0,69 | 0,002694 | Hdgfrp3 |
| ENSRNOG00000005703 | 251,76 | 0,67 | 0,002767 | Arfgef1 |
| ENSRNOG00000043391 | 806,71 | 0,54 | 0,002804 |  |
| ENSRNOG00000047137 | 224,01 | 0,59 | 0,002827 | Erbin |
| ENSRNOG00000011912 | 390,71 | -0,42 | 0,002851 | Tmem38a |
| ENSRNOG00000014142 | 72,92 | 0,67 | 0,002898 | Ogfrl1 |
| ENSRNOG00000027520 | 231,01 | 0,48 | 0,003045 | Pls3 |
| ENSRNOG00000017843 | 40,96 | -0,84 | 0,003046 | Polr3k |
| ENSRNOG00000020956 | 243,00 | -0,60 | 0,003046 | Bcat2 |
| ENSRNOG00000042821 | 633,45 | 0,31 | 0,003109 | Cd59 |
| ENSRNOG00000003163 | 1200,10 | -0,44 | 0,003117 | Sdhc |
| ENSRNOG00000038347 | 24,41 | -1,05 | 0,003125 | Usp21 |
| ENSRNOG00000015439 | 85,89 | 0,72 | 0,00314 | Man2a1 |
| ENSRNOG00000013917 | 35,99 | 1,09 | 0,00314 | Igsf10 |
| ENSRNOG00000016559 | 141,61 | -0,61 | 0,00314 | Tm2d2 |
| ENSRNOG00000002443 | 29,77 | 0,87 | 0,00314 | Mfap3 |
| ENSRNOG00000032844 | 128,91 | 0,86 | 0,003258 | RT1-Da |
| ENSRNOG00000007756 | 29,39 | -0,96 | 0,003308 | Atg101 |
| ENSRNOG00000023647 | 6457,34 | -0,36 | 0,003459 |  |
| ENSRNOG00000003302 | 201,81 | -0,55 | 0,003468 | Flcn |
| ENSRNOG00000019485 | 350,88 | -0,45 | 0,003558 | Bckdk |
| ENSRNOG00000015231 | 81,81 | -0,71 | 0,00366 | Mrpl44 |
| ENSRNOG00000006052 | 203,65 | 0,52 | 0,003668 | Sulf2 |
| ENSRNOG00000010676 | 125,36 | 0,63 | 0,003668 | Smarce1 |
| ENSRNOG00000011227 | 123,74 | 0,72 | 0,003668 | Atp1b2 |
| ENSRNOG00000015976 | 275,49 | -0,61 | 0,003672 |  |
| ENSRNOG00000011329 | 595,04 | -0,30 | 0,003741 | Pkm |
| ENSRNOG00000020812 | 315,70 | -0,49 | 0,003807 | Gys1 |
| ENSRNOG00000003103 | 45,19 | 0,85 | 0,003807 |  |
| ENSRNOG00000033261 | 315,07 | 0,84 | 0,003807 | Fam107a |
| ENSRNOG00000018029 | 133,49 | -0,64 | 0,003807 | Doc2g |
| ENSRNOG00000001061 | 22,28 | -0,98 | 0,003807 | Rilpl2 |
| ENSRNOG00000009078 | 226,78 | -0,55 | 0,003807 | Mrpl37 |
| ENSRNOG00000013949 | 5702,52 | -0,33 | 0,003864 | Idh2 |
| ENSRNOG00000007972 | 29,28 | 1,01 | 0,003872 | Rbbp9 |
| ENSRNOG00000012623 | 239,45 | 0,48 | 0,003899 | Arf4 |
| ENSRNOG00000045613 | 4177,03 | -0,52 | 0,003901 |  |
| ENSRNOG00000014541 | 25,20 | 1,12 | 0,003901 |  |
| ENSRNOG00000020386 | 290,86 | 0,61 | 0,003993 | Ash1l |
| ENSRNOG00000042411 | 21,69 | 0,99 | 0,004019 | Rps6ka1 |
| ENSRNOG00000015068 | 113,51 | -0,62 | 0,004057 | Il11ra1 |
| ENSRNOG00000023708 | 42,77 | 0,76 | 0,004175 | Tmem176a |
| ENSRNOG00000009184 | 67,69 | 0,82 | 0,004175 | Foxp1 |
| ENSRNOG00000021096 | 83,41 | -0,69 | 0,004175 | Tmem143 |
| ENSRNOG00000042720 | 136,60 | -0,65 | 0,004175 | Mrpl28 |
| ENSRNOG00000010904 | 160,05 | -0,57 | 0,004238 |  |
| ENSRNOG00000016680 | 174,00 | 0,72 | 0,004238 | Nsd1 |
| ENSRNOG00000001010 | 81,65 | -0,74 | 0,004238 | Tecpr1 |
| ENSRNOG00000034258 | 4443,05 | 0,66 | 0,004515 | Xirp2 |
| ENSRNOG00000031896 | 204,92 | 0,49 | 0,004515 | Chek1 |
| ENSRNOG00000020244 | 630,46 | -0,57 | 0,004636 | Perm1 |
| ENSRNOG00000013397 | 92,21 | 0,78 | 0,004636 | Foxo1 |
| ENSRNOG00000000137 | 8,55 | 1,13 | 0,004636 | Ly86 |
| ENSRNOG00000010620 | 21,05 | 1,03 | 0,004646 | Ndc1 |
| ENSRNOG00000022143 | 7,63 | -1,14 | 0,004646 | Dusp23 |
| ENSRNOG00000015912 | 78,86 | -0,62 | 0,004775 | Ptdss2 |
| ENSRNOG00000001982 | 64,24 | 0,77 | 0,004775 | Cblb |
| ENSRNOG00000007887 | 192,01 | 0,50 | 0,004775 | Elk4 |
| ENSRNOG00000016948 | 39,86 | -0,85 | 0,004914 | Nhlrc2 |
| ENSRNOG00000018903 | 296,21 | 0,68 | 0,004914 | Pik3r1 |
| ENSRNOG00000012405 | 167,75 | 0,64 | 0,004929 | Tcf4 |
| ENSRNOG00000008482 | 153,02 | 0,45 | 0,005177 | Rbms1 |
| ENSRNOG00000006355 | 91,88 | -0,85 | 0,005215 | Akap8l |
| ENSRNOG00000004834 | 67,06 | 0,71 | 0,005215 | Llgl2 |
| ENSRNOG00000049809 | 144,17 | -0,73 | 0,005268 |  |
| ENSRNOG00000013780 | 143,51 | 0,64 | 0,005324 | Nf1 |
| ENSRNOG00000013176 | 29,49 | 0,88 | 0,005434 | Far1 |
| ENSRNOG00000000168 | 80,02 | 0,61 | 0,005434 | Gatm |
| ENSRNOG00000000614 | 88,81 | 0,69 | 0,005452 | Bicc1 |
| ENSRNOG00000011951 | 91,69 | 0,64 | 0,005715 | Plk2 |
| ENSRNOG00000018518 | 31,11 | -0,94 | 0,005774 |  |
| ENSRNOG00000004696 | 123,08 | 0,72 | 0,005887 | Arhgap5 |
| ENSRNOG00000016827 | 151,65 | -0,61 | 0,006032 | Slc38a3 |
| ENSRNOG00000013452 | 266,85 | 0,52 | 0,006033 | Rcn1 |
| ENSRNOG00000008522 | 3,99 | -1,05 | 0,006033 |  |
| ENSRNOG00000020014 | 611,04 | -0,36 | 0,006115 | Myh14 |
| ENSRNOG00000013532 | 1408,32 | -0,58 | 0,006128 | Pgam2 |
| ENSRNOG00000012271 | 295,08 | 0,44 | 0,006128 | Cnot1 |
| ENSRNOG00000020492 | 54,33 | 0,96 | 0,006128 |  |
| ENSRNOG00000025757 | 41157,68 | -0,60 | 0,006155 | Myh6 |
| ENSRNOG00000011654 | 9,15 | 1,10 | 0,00623 | Plk4 |
| ENSRNOG00000003620 | 143,77 | 0,78 | 0,006255 | Fmo3 |
| ENSRNOG00000000645 | 89,18 | 0,64 | 0,006343 | Reep3 |
| ENSRNOG00000009845 | 2638,38 | -0,37 | 0,006365 | Acadm |
| ENSRNOG00000014117 | 39,11 | 0,92 | 0,006485 | Hmox1 |
| ENSRNOG00000002654 | 70,75 | 0,78 | 0,006518 |  |
| ENSRNOG00000049361 | 71,67 | 0,73 | 0,006578 | Gas7 |
| ENSRNOG00000008369 | 109,94 | 0,70 | 0,006779 | Gimap4 |
| ENSRNOG00000009790 | 168,89 | -0,69 | 0,007025 | Kcnk3 |
| ENSRNOG00000045913 | 14,28 | 1,09 | 0,007047 | Prdm16 |
| ENSRNOG00000011071 | 104,03 | 0,60 | 0,007232 | Nt5e |
| ENSRNOG00000007888 | 29,32 | -0,92 | 0,007233 |  |
| ENSRNOG00000010183 | 176,90 | 0,68 | 0,00724 | Fam198b |
| ENSRNOG00000050646 | 373,35 | -0,47 | 0,00724 | Fem1a |
| ENSRNOG00000019213 | 408,25 | -0,49 | 0,00724 |  |
| ENSRNOG00000047218 | 721,59 | 0,41 | 0,007306 | Clic5 |
| ENSRNOG00000013128 | 100,40 | -0,89 | 0,007325 | Tmem179 |
| ENSRNOG00000002286 | 8,18 | 1,09 | 0,007325 |  |
| ENSRNOG00000018835 | 39,74 | 0,97 | 0,007491 | Notch2 |
| ENSRNOG00000016208 | 25,49 | 0,90 | 0,007539 | Setbp1 |
| ENSRNOG00000013565 | 25,20 | 0,88 | 0,007632 | Zfp507 |
| ENSRNOG00000002372 | 47,58 | 0,83 | 0,007953 | Sgcd |
| ENSRNOG00000016190 | 1042,37 | -0,43 | 0,008128 | Coq9 |
| ENSRNOG00000022980 | 982,91 | -0,41 | 0,008181 | Sdhd |
| ENSRNOG00000000562 | 7,58 | 1,08 | 0,008237 |  |
| ENSRNOG00000006532 | 323,15 | 0,58 | 0,008251 | H3f3b |
| ENSRNOG00000029886 | 1416,93 | -0,70 | 0,008251 | Hba1 |
| ENSRNOG00000005841 | 161,77 | 0,44 | 0,008251 | Erp44 |
| ENSRNOG00000046468 | 99,92 | 0,81 | 0,008365 | Ptgfr |
| ENSRNOG00000002256 | 703,95 | 0,40 | 0,008365 | Art3 |
| ENSRNOG00000002407 | 30,11 | 0,94 | 0,008379 | Pdxdc1 |
| ENSRNOG00000011134 | 490,67 | 0,61 | 0,008412 | Lama2 |
| ENSRNOG00000021681 | 311,18 | 0,37 | 0,008627 | Eea1 |
| ENSRNOG00000021287 | 8,90 | -1,08 | 0,008629 | Hexim2 |
| ENSRNOG00000006338 | 108,27 | 0,66 | 0,008643 | Lrp6 |
| ENSRNOG00000017349 | 69,39 | -0,63 | 0,008643 | Tbc1d10b |
| ENSRNOG00000000610 | 340,07 | -0,53 | 0,008716 | Cisd1 |
| ENSRNOG00000007327 | 7,49 | -1,07 | 0,008716 | Pars2 |
| ENSRNOG00000001414 | 510,04 | 0,68 | 0,008963 | Serpine1 |
| ENSRNOG00000024089 | 88,20 | 0,71 | 0,009156 | Fndc3b |
| ENSRNOG00000004402 | 120,39 | 0,50 | 0,009156 | Lpgat1 |
| ENSRNOG00000006938 | 275,03 | -0,41 | 0,00922 |  |
| ENSRNOG00000026217 | 31,72 | -0,89 | 0,00922 | Armc2 |
| ENSRNOG00000001030 | 650,60 | 0,37 | 0,009355 | Tsc22d1 |
| ENSRNOG00000038766 | 30,02 | 0,87 | 0,009372 | Atxn1l |
| ENSRNOG00000030118 | 843,46 | 0,47 | 0,009502 | Msn |
| ENSRNOG00000020769 | 67,64 | 0,80 | 0,009511 | Crebrf |
| ENSRNOG00000018937 | 116,23 | -0,62 | 0,0096 | Gstm2 |
| ENSRNOG00000002035 | 14,36 | 1,04 | 0,009874 | Paqr3 |
| ENSRNOG00000027726 | 145,17 | -0,53 | 0,00993 |  |
| ENSRNOG00000001483 | 53,56 | -0,69 | 0,010212 | Rcc1l |
| ENSRNOG00000032994 | 47,84 | -0,74 | 0,010293 | Myom3 |
| ENSRNOG00000000146 | 260,69 | 0,62 | 0,010462 |  |
| ENSRNOG00000027756 | 66,99 | 0,77 | 0,010529 | Usf3 |
| ENSRNOG00000016983 | 31227,38 | 0,61 | 0,010583 | Myh7 |
| ENSRNOG00000019276 | 411,33 | -0,45 | 0,01066 | RGD735029 |
| ENSRNOG00000039576 | 13,45 | 0,98 | 0,01066 | Suv39h1 |
| ENSRNOG00000006694 | 1114,15 | 0,51 | 0,01066 |  |
| ENSRNOG00000008757 | 19,64 | -1,00 | 0,010672 | Tmem218 |
| ENSRNOG00000045771 | 14,89 | 1,05 | 0,010828 | Chl1 |
| ENSRNOG00000024885 | 47,17 | -0,82 | 0,010829 | Asb10 |
| ENSRNOG00000004473 | 112,37 | 0,67 | 0,010829 | Ppargc1a |
| ENSRNOG00000021090 | 2923,13 | -0,34 | 0,011028 | Pygm |
| ENSRNOG00000003120 | 105,62 | 0,79 | 0,01117 | Prelp |
| ENSRNOG00000012140 | 34,30 | -0,76 | 0,011193 | Cep89 |
| ENSRNOG00000003689 | 306,09 | 0,38 | 0,011257 | Nono |
| ENSRNOG00000033940 | 127,14 | 0,82 | 0,011365 | Adgrl4 |
| ENSRNOG00000007905 | 672,74 | -0,41 | 0,01138 | Itga7 |
| ENSRNOG00000003679 | 156,58 | 0,62 | 0,01138 | Med13 |
| ENSRNOG00000006094 | 87,40 | 0,73 | 0,011432 | Cd44 |
| ENSRNOG00000049056 | 157,02 | -0,62 | 0,011499 |  |
| ENSRNOG00000009412 | 49,60 | 0,72 | 0,011588 | Lrch1 |
| ENSRNOG00000009466 | 285,64 | -0,50 | 0,011679 | Unc45b |
| ENSRNOG00000005907 | 4,52 | -1,03 | 0,011883 | Rad18 |
| ENSRNOG00000034134 | 14,33 | 1,04 | 0,011883 | Cpm |
| ENSRNOG00000018604 | 537,55 | -0,35 | 0,011917 | Tufm |
| ENSRNOG00000002280 | 131,06 | 0,53 | 0,011967 | Sh3bgrl |
| ENSRNOG00000010362 | 315,52 | 0,65 | 0,011974 | Anxa2 |
| ENSRNOG00000011853 | 217,70 | 0,47 | 0,012098 | Mbd2 |
| ENSRNOG00000017251 | 245,23 | 0,38 | 0,012098 | Ik |
| ENSRNOG00000037967 | 17,64 | -0,91 | 0,012169 | Thap7 |
| ENSRNOG00000021200 | 403,19 | -0,50 | 0,012186 | Hfe2 |
| ENSRNOG00000004610 | 376,20 | 0,55 | 0,012186 | Lum |
| ENSRNOG00000006980 | 77,50 | 0,78 | 0,012257 | Vcpip1 |
| ENSRNOG00000004840 | 899,90 | 0,59 | 0,012314 |  |
| ENSRNOG00000011282 | 439,47 | -0,33 | 0,012458 | Ppp2r1a |
| ENSRNOG00000003258 | 51,57 | 0,73 | 0,012458 | Cdc73 |
| ENSRNOG00000029911 | 30,37 | 0,98 | 0,012458 | Cilp |
| ENSRNOG00000014178 | 194,95 | -0,41 | 0,01259 | Acad9 |
| ENSRNOG00000003139 | 223,61 | 0,43 | 0,012724 | Smc1a |
| ENSRNOG00000015573 | 52,25 | 0,64 | 0,012895 | Ctbs |
| ENSRNOG00000003732 | 28,84 | 0,96 | 0,012922 | Flrt2 |
| ENSRNOG00000025679 | 172,33 | -0,45 | 0,012955 | Stk40 |
| ENSRNOG00000017745 | 174,05 | -0,40 | 0,013072 | Cog4 |
| ENSRNOG00000012148 | 188,17 | 0,68 | 0,013134 | Trio |
| ENSRNOG00000015118 | 44,26 | 0,67 | 0,013417 | Cpped1 |
| ENSRNOG00000005695 | 1300,16 | 0,62 | 0,013417 | Mgp |
| ENSRNOG00000031814 | 90,92 | 0,74 | 0,013951 | Sort1 |
| ENSRNOG00000016748 | 44,74 | -0,75 | 0,013951 | Poll |
| ENSRNOG00000036692 | 11,39 | -1,02 | 0,014006 | Gcgr |
| ENSRNOG00000014747 | 57,63 | 0,88 | 0,014047 |  |
| ENSRNOG00000018129 | 778,10 | -0,49 | 0,014155 | Ndufab1 |
| ENSRNOG00000013679 | 52,73 | -0,76 | 0,014905 | Sema4d |
| ENSRNOG00000048812 | 799,73 | -0,57 | 0,014905 | Gpx1 |
| ENSRNOG00000038436 | 146,42 | 0,73 | 0,014905 | RGD1307100 |
| ENSRNOG00000033545 | 13,22 | 1,00 | 0,014905 |  |
| ENSRNOG00000010134 | 416,97 | -0,56 | 0,014988 | Acot2 |
| ENSRNOG00000008877 | 118,60 | -0,56 | 0,015136 |  |
| ENSRNOG00000000560 | 121,34 | -0,56 | 0,015137 | Lrrc20 |
| ENSRNOG00000012655 | 14,46 | 1,00 | 0,015143 | Adamts6 |
| ENSRNOG00000013451 | 185,72 | 0,40 | 0,015154 | Drosha |
| ENSRNOG00000013963 | 834,99 | 0,42 | 0,015154 | Il6st |
| ENSRNOG00000018095 | 54,57 | -0,74 | 0,015178 | Nkiras2 |
| ENSRNOG00000019549 | 305,48 | 0,54 | 0,015178 | Akap12 |
| ENSRNOG00000017854 | 1330,37 | -0,39 | 0,015178 | Ucp2 |
| ENSRNOG00000018181 | 178,74 | -0,44 | 0,015178 | Stk25 |
| ENSRNOG00000018012 | 74,73 | 0,72 | 0,015523 | Tulp4 |
| ENSRNOG00000023237 | 5,81 | 1,00 | 0,015575 |  |
| ENSRNOG00000010608 | 18,82 | 0,94 | 0,015585 | Cep162 |
| ENSRNOG00000010217 | 222,89 | 0,52 | 0,015781 | Prrc2b |
| ENSRNOG00000011387 | 30,34 | 0,89 | 0,016435 | Tet3 |
| ENSRNOG00000015158 | 47,71 | 0,72 | 0,016435 | Pikfyve |
| ENSRNOG00000003520 | 753,50 | -0,42 | 0,016663 | Prdx2 |
| ENSRNOG00000004303 | 584,07 | 0,44 | 0,016823 | Timp3 |
| ENSRNOG00000036604 | 169,53 | 0,49 | 0,016823 | Ifit2 |
| ENSRNOG00000037627 | 41,10 | 0,74 | 0,016823 | Trappc1 |
| ENSRNOG00000018414 | 124,31 | 0,70 | 0,017139 | Csf1r |
| ENSRNOG00000005504 | 355,44 | 0,38 | 0,017266 | Pkp4 |
| ENSRNOG00000011977 | 125,10 | 0,66 | 0,017284 | Sema5a |
| ENSRNOG00000002750 | 56,21 | 0,86 | 0,017284 |  |
| ENSRNOG00000017513 | 166,21 | -0,51 | 0,017425 | Miga2 |
| ENSRNOG00000033402 | 42,19 | 0,82 | 0,017425 | LOC501110 |
| ENSRNOG00000004059 | 4,92 | -0,98 | 0,017425 | Ptrhd1 |
| ENSRNOG00000006444 | 677,82 | -0,37 | 0,017425 | Fkbp4 |
| ENSRNOG00000002403 | 45,31 | 0,80 | 0,017425 | Fam129a |
| ENSRNOG00000014462 | 117,56 | 0,53 | 0,017559 | Fnta |
| ENSRNOG00000021652 | 28,72 | 0,79 | 0,017832 |  |
| ENSRNOG00000017189 | 106,32 | -0,68 | 0,017871 |  |
| ENSRNOG00000036913 | 272,55 | 0,57 | 0,018128 | RGD1309621 |
| ENSRNOG00000014529 | 48,37 | 0,73 | 0,018255 | Ube2q2 |
| ENSRNOG00000027191 | 215,89 | 0,71 | 0,018295 | Birc6 |
| ENSRNOG00000014722 | 122,20 | 0,52 | 0,018295 | Raph1 |
| ENSRNOG00000013072 | 39,37 | 0,96 | 0,018295 | Plxna4 |
| ENSRNOG00000011781 | 68,44 | -0,55 | 0,018324 | Oplah |
| ENSRNOG00000018618 | 149,53 | 0,53 | 0,018334 |  |
| ENSRNOG00000010783 | 20,93 | -0,88 | 0,018497 | Mak16 |
| ENSRNOG00000018516 | 55,54 | -0,73 | 0,018674 | Impa2 |
| ENSRNOG00000034198 | 129,42 | 0,60 | 0,018686 | Tceal9 |
| ENSRNOG00000004726 | 1033,61 | -0,41 | 0,01874 | Mapkapk2 |
| ENSRNOG00000018345 | 220,70 | 0,47 | 0,018746 | Abce1 |
| ENSRNOG00000003825 | 51,18 | 0,65 | 0,018893 | Wdr75 |
| ENSRNOG00000026928 | 6,07 | 0,99 | 0,018893 |  |
| ENSRNOG00000002104 | 100,90 | 0,49 | 0,018893 | Scaf4 |
| ENSRNOG00000018755 | 125,75 | -0,68 | 0,019013 | Acss2 |
| ENSRNOG00000012785 | 29,74 | -0,80 | 0,019017 | Armc10 |
| ENSRNOG00000005486 | 72,45 | 0,83 | 0,019383 |  |
| ENSRNOG00000048278 | 14,97 | 0,99 | 0,019405 |  |
| ENSRNOG00000015337 | 85,49 | 0,63 | 0,019418 |  |
| ENSRNOG00000019429 | 76,94 | 0,54 | 0,019434 |  |
| ENSRNOG00000010438 | 1200,78 | -0,40 | 0,019579 | Cpt1b |
| ENSRNOG00000007300 | 16,24 | 0,95 | 0,01962 | C1qtnf6 |
| ENSRNOG00000004526 | 295,21 | -0,53 | 0,01962 | Cox7a2l |
| ENSRNOG00000017226 | 1035,49 | -0,44 | 0,01962 | Slc2a4 |
| ENSRNOG00000004099 | 153,05 | 0,42 | 0,01962 | R3hdm1 |
| ENSRNOG00000015787 | 11,13 | -0,97 | 0,019701 | P3h4 |
| ENSRNOG00000008176 | 2716,95 | 0,99 | 0,019772 | Nppa |
| ENSRNOG00000010558 | 163,13 | -0,51 | 0,019786 | Ppif |
| ENSRNOG00000006966 | 193,34 | 0,44 | 0,019786 | Nfia |
| ENSRNOG00000000648 | 210,82 | 0,47 | 0,019811 | Jmjd1c |
| ENSRNOG00000047931 | 1415,18 | 0,60 | 0,019834 | Tmsb4x |
| ENSRNOG00000011775 | 40,80 | 0,86 | 0,019968 | Mfap3l |
| ENSRNOG00000046740 | 3,75 | 0,89 | 0,0201 |  |
| ENSRNOG00000011154 | 1305,89 | 0,53 | 0,02023 | Adgrf5 |
| ENSRNOG00000023661 | 43,61 | 0,82 | 0,020244 | Celf2 |
| ENSRNOG00000015194 | 11,13 | 0,91 | 0,020244 |  |
| ENSRNOG00000010865 | 134,22 | 0,54 | 0,020244 |  |
| ENSRNOG00000000299 | 82,30 | 0,74 | 0,020244 | Foxo3 |
| ENSRNOG00000049604 | 16,25 | -0,89 | 0,020338 | Mvk |
| ENSRNOG00000050343 | 23,74 | 0,83 | 0,020338 |  |
| ENSRNOG00000002028 | 270,77 | -0,51 | 0,020389 | Tmem50b |
| ENSRNOG00000012080 | 134,48 | 0,64 | 0,020418 |  |
| ENSRNOG00000016937 | 613,13 | -0,29 | 0,020418 | Mtfr1l |
| ENSRNOG00000002303 | 19,12 | -0,86 | 0,020418 | Kcnj12 |
| ENSRNOG00000000216 | 87,71 | 0,67 | 0,020494 |  |
| ENSRNOG00000010510 | 39,69 | 0,89 | 0,020511 |  |
| ENSRNOG00000001719 | 652,32 | -0,35 | 0,020511 | Psmd2 |
| ENSRNOG00000024563 | 46,38 | 0,67 | 0,02059 | Ganc |
| ENSRNOG00000003905 | 90,65 | 0,60 | 0,02059 | Nsf |
| ENSRNOG00000015904 | 267,07 | -0,63 | 0,02059 | Wfdc1 |
| ENSRNOG00000002680 | 1362,44 | 0,36 | 0,02059 | Lamc1 |
| ENSRNOG00000020659 | 138,29 | -0,54 | 0,020864 | Mrpl4 |
| ENSRNOG00000015153 | 150,09 | -0,50 | 0,020916 | Ints3 |
| ENSRNOG00000050414 | 10,70 | 0,96 | 0,021082 |  |
| ENSRNOG00000010958 | 668,06 | -0,30 | 0,021124 | Prdx3 |
| ENSRNOG00000021380 | 45,92 | -0,77 | 0,021124 | Fads6 |
| ENSRNOG00000003622 | 53,89 | 0,83 | 0,021161 | Cybb |
| ENSRNOG00000010964 | 481,23 | 0,46 | 0,021161 | Akap13 |
| ENSRNOG00000005614 | 261,50 | -0,56 | 0,021161 | Txn2 |
| ENSRNOG00000002461 | 277,51 | 0,63 | 0,021161 | Nid1 |
| ENSRNOG00000016217 | 24,93 | 0,82 | 0,021161 | Gtpbp4 |
| ENSRNOG00000015734 | 221,79 | 0,51 | 0,021377 | Ube3a |
| ENSRNOG00000012763 | 37,99 | -0,72 | 0,021461 | Cwf19l1 |
| ENSRNOG00000002232 | 105,62 | 0,68 | 0,021461 | Aff1 |
| ENSRNOG00000017579 | 69,61 | 0,77 | 0,021461 | Mylip |
| ENSRNOG00000008259 | 90,92 | 0,70 | 0,021461 |  |
| ENSRNOG00000010633 | 2217,54 | -0,35 | 0,021461 | Acsl1 |
| ENSRNOG00000011413 | 943,69 | -0,36 | 0,021461 | Scp2 |
| ENSRNOG00000000341 | 51,21 | 0,72 | 0,021461 | Nid2 |
| ENSRNOG00000018343 | 856,68 | -0,39 | 0,021461 | Isca1 |
| ENSRNOG00000030317 | 362,00 | 0,40 | 0,021544 |  |
| ENSRNOG00000012406 | 146,73 | -0,48 | 0,021544 | Pcbp4 |
| ENSRNOG00000004583 | 35758,54 | -0,40 | 0,021959 | Mb |
| ENSRNOG00000000413 | 4494,97 | -0,34 | 0,022035 | Pln |
| ENSRNOG00000001182 | 113,11 | -0,63 | 0,02227 | Ndufv3 |
| ENSRNOG00000018690 | 9,25 | 0,96 | 0,022305 | Rgs17 |
| ENSRNOG00000013443 | 323,71 | 0,37 | 0,022345 | Tm9sf3 |
| ENSRNOG00000045859 | 9,25 | 0,97 | 0,022345 |  |
| ENSRNOG00000029996 | 5,59 | 0,93 | 0,022345 |  |
| ENSRNOG00000010274 | 94,53 | 0,55 | 0,022397 | Smc4 |
| ENSRNOG00000019428 | 284,95 | -0,62 | 0,022507 | Higd1a |
| ENSRNOG00000007377 | 49,41 | 0,82 | 0,022507 | Slit3 |
| ENSRNOG00000011913 | 57,12 | 0,65 | 0,022518 | Cp |
| ENSRNOG00000012749 | 144,47 | 0,66 | 0,022607 | C1qb |
| ENSRNOG00000020289 | 107,09 | -0,54 | 0,022607 | Akt1s1 |
| ENSRNOG00000020347 | 152,43 | 0,43 | 0,022756 | Nup98 |
| ENSRNOG00000017635 | 551,50 | -0,31 | 0,022831 | Inpp5a |
| ENSRNOG00000000042 | 37,69 | 0,72 | 0,022831 | Xpr1 |
| ENSRNOG00000011293 | 36,46 | -0,72 | 0,022831 | Hpf1 |
| ENSRNOG00000002258 | 10,01 | -0,96 | 0,022855 | Tmem150c |
| ENSRNOG00000005929 | 6,08 | -0,96 | 0,022964 | Them6 |
| ENSRNOG00000050547 | 286,39 | -0,48 | 0,022964 | Syngr2 |
| ENSRNOG00000025570 | 177,09 | 0,42 | 0,023 | Os9 |
| ENSRNOG00000007040 | 289,42 | -0,46 | 0,023 | Timm17a |
| ENSRNOG00000011290 | 19,39 | -0,87 | 0,023 |  |
| ENSRNOG00000014478 | 95,66 | 0,67 | 0,023119 | Fndc3a |
| ENSRNOG00000031364 | 2013,08 | -0,21 | 0,023159 | Kif1c |
| ENSRNOG00000004481 | 75,66 | 0,64 | 0,023159 | Adss |
| ENSRNOG00000008374 | 9,26 | 0,96 | 0,023159 | Nipsnap1 |
| ENSRNOG00000023393 | 100,35 | 0,57 | 0,023315 | Nup214 |
| ENSRNOG00000042037 | 138,09 | -0,46 | 0,023315 | Smim7 |
| ENSRNOG00000008533 | 40,05 | 0,73 | 0,023315 | Ago2 |
| ENSRNOG00000007808 | 9,05 | -0,96 | 0,023315 | Nap1l5 |
| ENSRNOG00000019404 | 289,82 | -0,52 | 0,023315 | Hhatl |
| ENSRNOG00000033119 | 367,67 | 0,53 | 0,023315 | Plcb4 |
| ENSRNOG00000028356 | 1720,06 | -0,54 | 0,023421 | Chchd10 |
| ENSRNOG00000018224 | 17,89 | -0,95 | 0,02362 |  |
| ENSRNOG00000033765 | 724,79 | -0,35 | 0,023911 | Eif1 |
| ENSRNOG00000014204 | 242,60 | -0,45 | 0,023911 | Pacsin3 |
| ENSRNOG00000013326 | 20,16 | 0,85 | 0,023928 | Hmbox1 |
| ENSRNOG00000026044 | 14,55 | 0,91 | 0,023928 | Prrg1 |
| ENSRNOG00000049221 | 92,46 | -0,52 | 0,023928 | Kdm4b |
| ENSRNOG00000019939 | 112,06 | 0,63 | 0,024093 |  |
| ENSRNOG00000003495 | 505,23 | 0,34 | 0,024166 | Prpf8 |
| ENSRNOG00000005564 | 78,44 | 0,62 | 0,024243 | Ubn2 |
| ENSRNOG00000002467 | 1291,45 | -0,30 | 0,024352 |  |
| ENSRNOG00000002292 | 223,51 | 0,46 | 0,024538 | Hnrnpd |
| ENSRNOG00000015085 | 964,56 | -0,39 | 0,025021 | Dmpk |
| ENSRNOG00000002339 | 15,92 | 0,94 | 0,025166 | Mark1 |
| ENSRNOG00000031855 | 57,53 | 0,61 | 0,025166 | Actr3b |
| ENSRNOG00000017671 | 34,28 | 0,82 | 0,025223 | Rasa3 |
| ENSRNOG00000009595 | 8,73 | -0,95 | 0,025241 | Zbtb48 |
| ENSRNOG00000030245 | 498,62 | -0,35 | 0,025252 | Tango2 |
| ENSRNOG00000022472 | 18,81 | -0,84 | 0,025252 | Snap47 |
| ENSRNOG00000003434 | 37,36 | 0,79 | 0,025252 | Trove2 |
| ENSRNOG00000030116 | 131,54 | -0,50 | 0,025252 |  |
| ENSRNOG00000005257 | 858,98 | -0,33 | 0,025252 | Prkaca |
| ENSRNOG00000013994 | 56,56 | 0,75 | 0,025252 | Enpp1 |
| ENSRNOG00000002450 | 49,77 | 0,65 | 0,025252 | Rrp15 |
| ENSRNOG00000048955 | 116,04 | -0,82 | 0,025395 |  |
| ENSRNOG00000012325 | 222,94 | -0,41 | 0,025621 | Adk |
| ENSRNOG00000017036 | 90,45 | 0,51 | 0,02577 | Sec11c |
| ENSRNOG00000020479 | 301,99 | 0,40 | 0,02577 | Pik3c2a |
| ENSRNOG00000008713 | 10,18 | 0,94 | 0,025797 | Slc41a2 |
| ENSRNOG00000007892 | 222,82 | 0,50 | 0,025876 | Tram1 |
| ENSRNOG00000037664 | 111,04 | -0,61 | 0,025937 |  |
| ENSRNOG00000011619 | 126,54 | 0,67 | 0,026037 | Myo9a |
| ENSRNOG00000008610 | 20,23 | 0,88 | 0,02619 | Mbip |
| ENSRNOG00000004249 | 18,73 | 0,92 | 0,026227 | Tlr7 |
| ENSRNOG00000005130 | 4089,19 | -0,38 | 0,026239 | Ogdh |
| ENSRNOG00000047262 | 4,63 | 0,75 | 0,026276 |  |
| ENSRNOG00000013195 | 20,64 | -0,85 | 0,026276 | Ruvbl1 |
| ENSRNOG00000009258 | 13,09 | 0,92 | 0,026788 | Cdk6 |
| ENSRNOG00000014665 | 153,39 | -0,43 | 0,026847 | Dhdds |
| ENSRNOG00000019918 | 42,15 | -0,69 | 0,02697 | Coasy |
| ENSRNOG00000003969 | 4,26 | -0,91 | 0,02697 | Fam20a |
| ENSRNOG00000029958 | 4,89 | 0,89 | 0,027229 |  |
| ENSRNOG00000023760 | 11,61 | 0,92 | 0,027352 | Plekhm3 |
| ENSRNOG00000004794 | 97,87 | 0,62 | 0,027352 | Rtn1 |
| ENSRNOG00000019465 | 349,64 | 0,44 | 0,027352 | Gnai3 |
| ENSRNOG00000004677 | 87,13 | 0,74 | 0,027352 | Zeb2 |
| ENSRNOG00000008897 | 145,48 | -0,50 | 0,027352 | Gga1 |
| ENSRNOG00000001335 | 37,88 | 0,80 | 0,027363 | Zkscan1 |
| ENSRNOG00000013260 | 24,45 | 0,80 | 0,027363 | Calr3 |
| ENSRNOG00000016334 | 93,95 | 0,59 | 0,027363 | Ptbp3 |
| ENSRNOG00000014836 | 102,88 | 0,54 | 0,027363 | Ralgapb |
| ENSRNOG00000001515 | 153,19 | 0,51 | 0,027623 | Zak |
| ENSRNOG00000008964 | 96,51 | -0,47 | 0,027744 |  |
| ENSRNOG00000011559 | 204,84 | 0,44 | 0,027965 | Cnn3 |
| ENSRNOG00000000595 | 3,97 | -0,91 | 0,028116 | Traf3ip2 |
| ENSRNOG00000001645 | 294,93 | 0,55 | 0,028362 | Filip1l |
| ENSRNOG00000001493 | 29,13 | -0,79 | 0,028362 |  |
| ENSRNOG00000018829 | 24,72 | -0,83 | 0,028393 | RGD1308134 |
| ENSRNOG00000007719 | 25,92 | 0,87 | 0,028418 | Ccnc |
| ENSRNOG00000019648 | 181,68 | 0,54 | 0,028418 |  |
| ENSRNOG00000010027 | 25,62 | 0,83 | 0,028418 | Atr |
| ENSRNOG00000017060 | 5226,28 | 0,43 | 0,028428 | Ryr2 |
| ENSRNOG00000028422 | 73,66 | 0,65 | 0,028724 | Rmnd5a |
| ENSRNOG00000018317 | 70,22 | 0,62 | 0,02874 | Aak1 |
| ENSRNOG00000017993 | 48,28 | -0,70 | 0,028764 | Abcb10 |
| ENSRNOG00000008421 | 22,25 | 0,91 | 0,028764 | Klhl5 |
| ENSRNOG00000004063 | 14,47 | 0,87 | 0,028788 | Sh3pxd2b |
| ENSRNOG00000021298 | 67,55 | 0,53 | 0,028902 | Dstyk |
| ENSRNOG00000001285 | 24960,11 | -0,31 | 0,029121 | Atp2a2 |
| ENSRNOG00000014753 | 73,94 | 0,68 | 0,029132 | Tcf7l1 |
| ENSRNOG00000049350 | 5,52 | 0,80 | 0,029167 |  |
| ENSRNOG00000009460 | 135,35 | 0,39 | 0,029167 | Pdzd8 |
| ENSRNOG00000004057 | 83,05 | 0,67 | 0,029203 | Ccdc88a |
| ENSRNOG00000016181 | 54,60 | 0,68 | 0,029261 | Tbx20 |
| ENSRNOG00000018168 | 99,70 | -0,54 | 0,029277 | Klc4 |
| ENSRNOG00000007427 | 40,80 | -0,69 | 0,029327 | Entpd6 |
| ENSRNOG00000023278 | 79,97 | -0,54 | 0,029328 | Sec61a2 |
| ENSRNOG00000022781 | 213,01 | 0,53 | 0,029868 | Ccser2 |
| ENSRNOG00000019062 | 21,75 | 0,76 | 0,030078 |  |
| ENSRNOG00000008741 | 214,24 | 0,45 | 0,030188 | Camsap2 |
| ENSRNOG00000001484 | 84,86 | 0,61 | 0,030217 | Gatsl2 |
| ENSRNOG00000008298 | 97,26 | 0,59 | 0,030249 | Dock7 |
| ENSRNOG00000014187 | 47,12 | 0,75 | 0,030282 | Igf1r |
| ENSRNOG00000012236 | 91,37 | -0,58 | 0,030427 | Hddc3 |
| ENSRNOG00000003148 | 353,87 | 0,48 | 0,030536 | Timp2 |
| ENSRNOG00000013507 | 108,15 | 0,54 | 0,030536 | Selt |
| ENSRNOG00000004398 | 27,87 | 0,82 | 0,030536 | Pkhd1l1 |
| ENSRNOG00000007399 | 4,94 | -0,92 | 0,030536 |  |
| ENSRNOG00000009686 | 150,97 | -0,60 | 0,030536 | Aqp7 |
| ENSRNOG00000042501 | 114,68 | -0,54 | 0,030536 | RGD1564379 |
| ENSRNOG00000020206 | 967,44 | -0,30 | 0,030667 | Ctsd |
| ENSRNOG00000016484 | 89,96 | -0,62 | 0,030684 | Gstk1 |
| ENSRNOG00000043288 | 4,21 | -0,89 | 0,03069 | Smim13 |
| ENSRNOG00000033623 | 22,60 | -0,80 | 0,03069 | Pigx |
| ENSRNOG00000000279 | 134,54 | -0,54 | 0,031025 | Rtn4ip1 |
| ENSRNOG00000009211 | 6,87 | 0,90 | 0,03129 | C3ar1 |
| ENSRNOG00000028335 | 58,64 | 0,84 | 0,031591 | Fat4 |
| ENSRNOG00000022769 | 64,05 | 0,67 | 0,031591 |  |
| ENSRNOG00000009841 | 90,10 | 0,54 | 0,031591 |  |
| ENSRNOG00000007673 | 219,24 | 0,44 | 0,031591 | Ppig |
| ENSRNOG00000006151 | 12,70 | 0,87 | 0,031701 | Reg3b |
| ENSRNOG00000042123 | 9,26 | 0,91 | 0,031707 |  |
| ENSRNOG00000015036 | 453,83 | 0,84 | 0,031722 | Ctgf |
| ENSRNOG00000010630 | 33,47 | 0,85 | 0,031722 | Prcp |
| ENSRNOG00000002653 | 24,75 | -0,86 | 0,031799 | Kcnk2 |
| ENSRNOG00000014806 | 244,72 | -0,43 | 0,032105 | Pnkd |
| ENSRNOG00000016029 | 171,67 | 0,49 | 0,032246 | Rb1 |
| ENSRNOG00000002585 | 92,40 | 0,55 | 0,032246 | Cul4b |
| ENSRNOG00000021338 | 97,03 | -0,51 | 0,032363 | Tmem132a |
| ENSRNOG00000000977 | 14,23 | -0,89 | 0,032363 | Pnpla6 |
| ENSRNOG00000021102 | 613,17 | -0,31 | 0,032401 | Scn1b |
| ENSRNOG00000018114 | 1440,41 | -0,35 | 0,032401 | Acadvl |
| ENSRNOG00000013912 | 20,48 | 0,85 | 0,032633 | Slc30a7 |
| ENSRNOG00000018087 | 1007,67 | 0,43 | 0,032934 | Vim |
| ENSRNOG00000001773 | 53,67 | -0,66 | 0,032934 | Senp2 |
| ENSRNOG00000012840 | 3805,40 | 0,33 | 0,032934 | Sparc |
| ENSRNOG00000011971 | 280,83 | 0,49 | 0,033025 | C1s |
| ENSRNOG00000001086 | 59,97 | -0,60 | 0,033196 | Vps37b |
| ENSRNOG00000014526 | 34,43 | -0,70 | 0,033262 | Cars2 |
| ENSRNOG00000003873 | 367,22 | 0,44 | 0,033295 | Cpd |
| ENSRNOG00000012710 | 136,37 | -0,48 | 0,033318 | Ubac2 |
| ENSRNOG00000037645 | 45,23 | 0,79 | 0,033376 | Tceal7 |
| ENSRNOG00000008115 | 26,32 | 0,77 | 0,033376 | Arhgap11a |
| ENSRNOG00000019462 | 62,47 | 0,54 | 0,033376 |  |
| ENSRNOG00000037249 | 82,07 | -0,58 | 0,033376 |  |
| ENSRNOG00000004295 | 41,96 | 0,81 | 0,033409 |  |
| ENSRNOG00000006180 | 387,39 | 0,33 | 0,033409 | Pum2 |
| ENSRNOG00000045961 | 98,68 | 0,47 | 0,033449 | Lyrm7 |
| ENSRNOG00000037283 | 85,96 | -0,66 | 0,033593 |  |
| ENSRNOG00000004205 | 94,52 | -0,68 | 0,033593 | Pkdcc |
| ENSRNOG00000005544 | 75,33 | 0,55 | 0,033608 |  |
| ENSRNOG00000030210 | 49,91 | 0,74 | 0,033981 | Fndc1 |
| ENSRNOG00000024272 | 23,59 | 0,77 | 0,033981 | Ino80d |
| ENSRNOG00000015941 | 68,19 | 0,58 | 0,034033 | Fkbp10 |
| ENSRNOG00000046283 | 38,64 | -0,72 | 0,034312 | Tmem41a |
| ENSRNOG00000026407 | 5,43 | 0,88 | 0,034312 | Fam184a |
| ENSRNOG00000048282 | 50,41 | -0,65 | 0,034365 | Mpnd |
| ENSRNOG00000013014 | 18,42 | -0,88 | 0,034365 | Cyba |
| ENSRNOG00000018531 | 54,73 | -0,68 | 0,034423 | Mrps11 |
| ENSRNOG00000000657 | 83,55 | 0,67 | 0,034661 | Nek7 |
| ENSRNOG00000016740 | 71,25 | 0,60 | 0,034819 | Fam210a |
| ENSRNOG00000009808 | 18,71 | 0,85 | 0,034826 | Ift80 |
| ENSRNOG00000029778 | 15,34 | 0,85 | 0,034886 | Maob |
| ENSRNOG00000012660 | 102,92 | 0,85 | 0,03528 | Postn |
| ENSRNOG00000000900 | 21,27 | 0,78 | 0,035327 | Tpst1 |
| ENSRNOG00000047287 | 58,32 | 0,54 | 0,035392 | Nucks1 |
| ENSRNOG00000022607 | 14,65 | -0,86 | 0,035466 |  |
| ENSRNOG00000043189 | 47,43 | -0,65 | 0,035563 | Trub2 |
| ENSRNOG00000050748 | 86,35 | -0,57 | 0,035563 | Dpp9 |
| ENSRNOG00000011077 | 455,09 | 0,41 | 0,035563 | Tjp1 |
| ENSRNOG00000009419 | 187,42 | 0,56 | 0,035563 | Ptprg |
| ENSRNOG00000015450 | 19,29 | -0,84 | 0,035671 | Ppp1r16a |
| ENSRNOG00000036693 | 34,57 | -0,69 | 0,035691 | Slc25a10 |
| ENSRNOG00000002644 | 26,81 | 0,75 | 0,035881 | Utp18 |
| ENSRNOG00000026569 | 14,39 | 0,85 | 0,035881 | LOC102555377 |
| ENSRNOG00000026589 | 75,07 | 0,65 | 0,036027 | Dpy19l1 |
| ENSRNOG00000003897 | 611,52 | 0,74 | 0,036027 | Col1a1 |
| ENSRNOG00000016541 | 116,39 | 0,68 | 0,036027 | Enc1 |
| ENSRNOG00000010437 | 11,58 | 0,87 | 0,036027 |  |
| ENSRNOG00000005987 | 48,69 | -0,57 | 0,036039 | Suox |
| ENSRNOG00000049982 | 13,13 | -0,89 | 0,036039 | Pex26 |
| ENSRNOG00000015633 | 509,05 | 0,30 | 0,036221 | Cul3 |
| ENSRNOG00000002229 | 398,18 | -0,33 | 0,036221 | Adcy5 |
| ENSRNOG00000001547 | 23,98 | 0,78 | 0,036221 | Agps |
| ENSRNOG00000001172 | 716,61 | -0,27 | 0,036221 | Rnf10 |
| ENSRNOG00000048651 | 7,37 | -0,89 | 0,036472 | Nrtn |
| ENSRNOG00000020433 | 611,72 | 0,42 | 0,036753 | Actn4 |
| ENSRNOG00000007975 | 79,01 | 0,65 | 0,037079 | Ncoa2 |
| ENSRNOG00000013744 | 74,61 | 0,67 | 0,037225 | Akip1 |
| ENSRNOG00000019097 | 150,37 | -0,40 | 0,037635 | Bap1 |
| ENSRNOG00000018233 | 1027,35 | -0,30 | 0,037635 | Gas6 |
| ENSRNOG00000020723 | 120,65 | 0,55 | 0,037635 | Pten |
| ENSRNOG00000019962 | 19,58 | -0,82 | 0,037771 | Sars2 |
| ENSRNOG00000011054 | 75,86 | 0,54 | 0,038004 | Laptm5 |
| ENSRNOG00000006733 | 53,72 | 0,59 | 0,038127 | Srgap2 |
| ENSRNOG00000047439 | 13,55 | 0,86 | 0,03816 | Pot1b |
| ENSRNOG00000013186 | 421,24 | -0,30 | 0,03816 | G3bp1 |
| ENSRNOG00000033498 | 25,48 | -0,80 | 0,038469 | Cib1 |
| ENSRNOG00000016558 | 28,69 | -0,80 | 0,038469 | Pllp |
| ENSRNOG00000018781 | 25,65 | -0,76 | 0,03855 | Map1s |
| ENSRNOG00000007436 | 5,46 | 0,89 | 0,03855 | LOC100158225 |
| ENSRNOG00000020288 | 507,42 | -0,40 | 0,038687 | Slc25a20 |
| ENSRNOG00000012486 | 18,38 | 0,88 | 0,039064 | Prim2 |
| ENSRNOG00000000867 | 209,01 | -0,36 | 0,039064 | Vars |
| ENSRNOG00000011076 | 194,97 | 0,54 | 0,039097 | Ank2 |
| ENSRNOG00000037446 | 140,16 | -0,67 | 0,039097 | Pxmp2 |
| ENSRNOG00000000075 | 26,40 | 0,83 | 0,039152 | Mtf2 |
| ENSRNOG00000001065 | 151,49 | 0,52 | 0,039152 | Cyth3 |
| ENSRNOG00000005480 | 279,72 | -0,34 | 0,039254 | Ybx3 |
| ENSRNOG00000032659 | 14,95 | 0,87 | 0,039358 | Plcl1 |
| ENSRNOG00000020440 | 36,10 | 0,73 | 0,039497 | Fads2 |
| ENSRNOG00000002093 | 102,75 | 0,64 | 0,039497 | Tgfbr3 |
| ENSRNOG00000039472 | 6,86 | 0,81 | 0,039497 |  |
| ENSRNOG00000000314 | 239,98 | 0,30 | 0,03979 | Sec63 |
| ENSRNOG00000007246 | 38,84 | 0,61 | 0,039816 | Atxn7 |
| ENSRNOG00000013786 | 394,24 | 0,48 | 0,039869 |  |
| ENSRNOG00000001120 | 115,04 | 0,55 | 0,039869 | Med13l |
| ENSRNOG00000014532 | 31,11 | 0,82 | 0,039869 | Lbp |
| ENSRNOG00000047179 | 1011,13 | 0,29 | 0,039869 | Aplp2 |
| ENSRNOG00000008637 | 38,68 | 0,67 | 0,040024 | Strada |
| ENSRNOG00000030759 | 8,45 | 0,88 | 0,040024 | Nhs |
| ENSRNOG00000008180 | 92,17 | 0,44 | 0,040024 | Lyn |
| ENSRNOG00000019113 | 457,29 | 0,35 | 0,04034 | Hnrnpk |
| ENSRNOG00000019996 | 1203,38 | -0,28 | 0,04034 | Slc16a1 |
| ENSRNOG00000012149 | 7,30 | 0,87 | 0,04034 | Gpsm2 |
| ENSRNOG00000009928 | 438,45 | -0,43 | 0,040634 | Bckdhb |
| ENSRNOG00000042114 | 5,41 | -0,88 | 0,040634 | Erich1 |
| ENSRNOG00000014044 | 108,99 | -0,45 | 0,040723 | Pank4 |
| ENSRNOG00000024345 | 70,40 | 0,70 | 0,040753 | Pard3b |
| ENSRNOG00000013876 | 92,98 | -0,50 | 0,040753 | Mipep |
| ENSRNOG00000005924 | 125,02 | 0,55 | 0,040753 | Dstnl1 |
| ENSRNOG00000004688 | 68,63 | 0,68 | 0,040753 | Rbfox2 |
| ENSRNOG00000019497 | 246,84 | -0,39 | 0,040753 | Mrpl17 |
| ENSRNOG00000016744 | 118,47 | 0,51 | 0,04142 | Chd6 |
| ENSRNOG00000004554 | 2218,07 | 0,30 | 0,041564 | Dcn |
| ENSRNOG00000019342 | 194,02 | 0,68 | 0,041642 | Sult1a1 |
| ENSRNOG00000028594 | 214,21 | 0,36 | 0,041694 | Ifnar1 |
| ENSRNOG00000047800 | 30,26 | 0,80 | 0,041879 | C5ar1 |
| ENSRNOG00000005798 | 236,21 | -0,49 | 0,041879 | Cav3 |
| ENSRNOG00000001344 | 1229,62 | -0,30 | 0,041914 | Aldh2 |
| ENSRNOG00000016603 | 163,25 | -0,57 | 0,042004 | Rtn2 |
| ENSRNOG00000032048 | 24,35 | 0,82 | 0,042123 | Zfp462 |
| ENSRNOG00000022419 | 32,36 | -0,71 | 0,042123 | Dok7 |
| ENSRNOG00000007102 | 706,59 | -0,38 | 0,042302 | Acss1 |
| ENSRNOG00000043348 | 5,46 | 0,82 | 0,042629 | Rpl39 |
| ENSRNOG00000033155 | 117,45 | 0,41 | 0,042767 |  |
| ENSRNOG00000005099 | 11,35 | -0,84 | 0,042794 | Top3a |
| ENSRNOG00000018020 | 105,31 | -0,47 | 0,043172 | Apbb1 |
| ENSRNOG00000033192 | 91,13 | 0,62 | 0,043217 | Osmr |
| ENSRNOG00000021688 | 14,74 | -0,85 | 0,043217 |  |
| ENSRNOG00000004026 | 141,87 | 0,56 | 0,043263 | Atp2b1 |
| ENSRNOG00000021872 | 15,50 | -0,81 | 0,043303 | Zfp213 |
| ENSRNOG00000004284 | 207,11 | 0,46 | 0,043303 | Btg1 |
| ENSRNOG00000024322 | 29,14 | 0,78 | 0,043303 | Shroom2 |
| ENSRNOG00000010872 | 390,44 | -0,49 | 0,043303 | Ckb |
| ENSRNOG00000000866 | 80,64 | 0,58 | 0,043322 | Rbmx |
| ENSRNOG00000021157 | 81,86 | 0,46 | 0,043808 |  |
| ENSRNOG00000021128 | 101,01 | -0,54 | 0,043808 | Kcnj11 |
| ENSRNOG00000010830 | 13470,58 | -0,40 | 0,044036 | Slc25a4 |
| ENSRNOG00000009421 | 662,11 | -0,37 | 0,044099 | Ivd |
| ENSRNOG00000002175 | 34,61 | 0,65 | 0,044099 | Clock |
| ENSRNOG00000014182 | 2339,16 | 0,38 | 0,044109 | Tns1 |
| ENSRNOG00000009422 | 160,87 | -0,42 | 0,044132 | Hmgcl |
| ENSRNOG00000047321 | 642,83 | -0,58 | 0,04433 | Hba2 |
| ENSRNOG00000030683 | 104,38 | 0,55 | 0,044513 | Vps26a |
| ENSRNOG00000013668 | 149,27 | 0,60 | 0,044925 | Capg |
| ENSRNOG00000005826 | 16,51 | 0,85 | 0,044925 | RGD1562420 |
| ENSRNOG00000024907 | 340,76 | 0,41 | 0,044925 | Tnrc6b |
| ENSRNOG00000011358 | 392,43 | 0,46 | 0,044925 | Hipk3 |
| ENSRNOG00000002418 | 96,79 | 0,70 | 0,045125 | Tgfb2 |
| ENSRNOG00000012346 | 229,42 | 0,57 | 0,045181 | Epb41l2 |
| ENSRNOG00000019419 | 11,39 | -0,83 | 0,045342 | Taf6l |
| ENSRNOG00000017564 | 74,69 | -0,62 | 0,045342 | Mib2 |
| ENSRNOG00000012184 | 179,15 | -0,43 | 0,045342 | Urgcp |
| ENSRNOG00000009449 | 190,29 | -0,48 | 0,04541 | Trim35 |
| ENSRNOG00000017608 | 57,94 | 0,70 | 0,045571 | C2cd3 |
| ENSRNOG00000024796 | 52,66 | -0,70 | 0,045571 | Lrrc47 |
| ENSRNOG00000019048 | 892,33 | -0,36 | 0,045609 | Sod2 |
| ENSRNOG00000018044 | 4259,38 | -0,28 | 0,045687 | Phyh |
| ENSRNOG00000008187 | 10,28 | 0,87 | 0,045693 | Ubash3b |
| ENSRNOG00000014426 | 25,60 | 0,84 | 0,04587 | Lox |
| ENSRNOG00000009047 | 6,78 | -0,86 | 0,04587 | Sln |
| ENSRNOG00000012806 | 177,22 | 0,47 | 0,046132 | Rbbp6 |
| ENSRNOG00000006991 | 152,44 | -0,49 | 0,046132 |  |
| ENSRNOG00000013766 | 2176,31 | -0,35 | 0,046207 | Acaa2 |
| ENSRNOG00000014235 | 18,90 | -0,79 | 0,046389 | Zgpat |
| ENSRNOG00000028261 | 27,79 | 0,73 | 0,046389 | Tppp |
| ENSRNOG00000001090 | 101,82 | 0,53 | 0,046389 | Stard13 |
| ENSRNOG00000001582 | 25,18 | 0,82 | 0,046389 | Bach1 |
| ENSRNOG00000001979 | 1305,64 | -0,38 | 0,046389 | Rcan1 |
| ENSRNOG00000048719 | 23,02 | -0,77 | 0,046416 | Dtnbp1 |
| ENSRNOG00000014452 | 34,46 | 0,77 | 0,046416 | Zfhx3 |
| ENSRNOG00000026008 | 12,07 | -0,82 | 0,046416 | Gtf2e1 |
| ENSRNOG00000019692 | 7,69 | -0,86 | 0,04642 | Metrn |
| ENSRNOG00000014625 | 1070,43 | -0,48 | 0,046836 | Atp5d |
| ENSRNOG00000015619 | 155,75 | 0,44 | 0,047113 | Agfg1 |
| ENSRNOG00000017714 | 15,74 | -0,84 | 0,047182 | Usp3 |
| ENSRNOG00000015332 | 17,56 | -0,81 | 0,047182 | Thoc1 |
| ENSRNOG00000030712 | 170,54 | -0,52 | 0,047494 | RT1-A2 |
| ENSRNOG00000005151 | 6,24 | -0,86 | 0,047551 | Dync2li1 |
| ENSRNOG00000009385 | 14,97 | 0,85 | 0,047683 | Pik3cg |
| ENSRNOG00000039890 | 7,21 | -0,85 | 0,047683 | Abcg3l3 |
| ENSRNOG00000019205 | 293,19 | -0,46 | 0,047683 | Gnpat |
| ENSRNOG00000009832 | 189,65 | -0,37 | 0,047683 | Slc39a14 |
| ENSRNOG00000009848 | 6,90 | 0,85 | 0,047683 | Il18 |
| ENSRNOG00000017342 | 92,73 | -0,51 | 0,047683 | Zdhhc7 |
| ENSRNOG00000036960 | 303,50 | 0,51 | 0,047683 | Abcc9 |
| ENSRNOG00000014597 | 42,38 | 0,73 | 0,048008 | Irs1 |
| ENSRNOG00000011142 | 217,23 | -0,39 | 0,048054 | Cyb5b |
| ENSRNOG00000028746 | 268,16 | 0,39 | 0,048054 | Gsto1 |
| ENSRNOG00000001875 | 67,19 | -0,54 | 0,048144 | Smpd4 |
| ENSRNOG00000017420 | 18,13 | 0,75 | 0,048219 | Nudt6 |
| ENSRNOG00000025443 | 880,01 | -0,46 | 0,048595 | Map1lc3a |
| ENSRNOG00000011445 | 8,55 | -0,85 | 0,048634 | Nkain1 |
| ENSRNOG00000000799 | 450,30 | -0,31 | 0,048634 | Abcf1 |
| ENSRNOG00000021440 | 485,69 | -0,41 | 0,048705 | Pptc7 |
| ENSRNOG00000016275 | 28,80 | -0,77 | 0,048714 | Ttr |
| ENSRNOG00000026605 | 49,97 | 0,77 | 0,04877 | Ifi27l2b |
| ENSRNOG00000049452 | 61,56 | 0,58 | 0,049297 | Nebl |
| ENSRNOG00000000327 | 33,34 | 0,67 | 0,049333 | Hace1 |
| ENSRNOG00000017265 | 213,30 | 0,42 | 0,049502 | Tmem131 |
| ENSRNOG00000004291 | 817,05 | 0,31 | 0,049562 | Cltc |
| ENSRNOG00000031135 | 254,79 | 0,37 | 0,049562 | Smarcc2 |
| ENSRNOG00000033913 | 3,58 | 0,68 | 0,049701 |  |
| ENSRNOG00000037274 | 24,93 | 0,80 | 0,049701 |  |
| ENSRNOG00000014504 | 97,91 | 0,65 | 0,049701 | Il1r1 |
| ENSRNOG00000019749 | 124,89 | -0,56 | 0,050416 | Ube2j2 |
| ENSRNOG00000003134 | 46,30 | 0,72 | 0,050416 | Slc4a4 |
| ENSRNOG00000008676 | 336,47 | 0,51 | 0,050515 | Emp1 |
| ENSRNOG00000010960 | 280,68 | -0,37 | 0,050515 | Ankh |
| ENSRNOG00000011518 | 26,62 | 0,83 | 0,050634 | Dusp26 |
| ENSRNOG00000001825 | 256,26 | -0,39 | 0,050671 |  |
| ENSRNOG00000027032 | 23,44 | -0,74 | 0,050674 | Rhbdd3 |
| ENSRNOG00000029855 | 23,05 | -0,79 | 0,050754 | Letmd1 |
| ENSRNOG00000021052 | 179,77 | 0,45 | 0,050754 | Patl1 |
| ENSRNOG00000039630 | 174,18 | 0,53 | 0,050754 |  |
| ENSRNOG00000017428 | 334,67 | 0,57 | 0,050791 | Map1b |
| ENSRNOG00000049424 | 446,27 | -0,61 | 0,050838 |  |
| ENSRNOG00000011512 | 30,74 | 0,83 | 0,050961 |  |
| ENSRNOG00000047625 | 71,29 | -0,63 | 0,051068 | Atg4d |
| ENSRNOG00000006939 | 344,02 | -0,49 | 0,05107 | Ndufa7 |
| ENSRNOG00000010838 | 124,40 | -0,52 | 0,051108 | Araf |
| ENSRNOG00000034254 | 1704,29 | 0,30 | 0,05117 | Actb |
| ENSRNOG00000016165 | 34,97 | 0,82 | 0,051415 |  |
| ENSRNOG00000009311 | 29,43 | 0,68 | 0,051492 | Fstl3 |
| ENSRNOG00000031053 | 12231,46 | 0,59 | 0,052127 | ND4L |
| ENSRNOG00000015869 | 249,45 | -0,41 | 0,052127 | Pccb |
| ENSRNOG00000015560 | 89,52 | 0,57 | 0,052172 | Emsy |
| ENSRNOG00000020593 | 4,27 | -0,83 | 0,052172 | Gipc3 |
| ENSRNOG00000000108 | 40,46 | 0,59 | 0,052172 | Aga |
| ENSRNOG00000020996 | 45,34 | -0,55 | 0,052259 |  |
| ENSRNOG00000014866 | 26,15 | 0,79 | 0,052259 | Pign |
| ENSRNOG00000026930 | 1214,27 | -0,29 | 0,052555 |  |
| ENSRNOG00000008839 | 53,56 | 0,68 | 0,052591 | Pparg |
| ENSRNOG00000003833 | 76,74 | -0,56 | 0,052763 | Nenf |
| ENSRNOG00000020436 | 166,90 | -0,54 | 0,052763 |  |
| ENSRNOG00000024145 | 21,25 | -0,82 | 0,053287 | Trim65 |
| ENSRNOG00000028227 | 116,67 | 0,59 | 0,053287 | Pbrm1 |
| ENSRNOG00000032487 | 133,72 | -0,48 | 0,053487 | Sts |
| ENSRNOG00000013252 | 46,60 | 0,70 | 0,053902 | Cwc27 |
| ENSRNOG00000005101 | 156,46 | 0,39 | 0,053975 | LOC100912115 |
| ENSRNOG00000012470 | 22,83 | 0,78 | 0,054451 | Zc3h12c |
| ENSRNOG00000002178 | 67,53 | -0,58 | 0,054451 | Mrps18c |
| ENSRNOG00000002730 | 274,78 | 0,52 | 0,054462 | Rgs5 |
| ENSRNOG00000019773 | 130,07 | -0,47 | 0,05471 |  |
| ENSRNOG00000002956 | 80,67 | 0,54 | 0,05471 | Stim2 |
| ENSRNOG00000008441 | 202,86 | 0,32 | 0,05471 |  |
| ENSRNOG00000028530 | 21,38 | 0,77 | 0,05471 |  |
| ENSRNOG00000002563 | 83,60 | 0,57 | 0,054814 | Mcts1 |
| ENSRNOG00000001585 | 91,89 | 0,55 | 0,054983 | Nrip1 |
| ENSRNOG00000049507 | 14,88 | 0,83 | 0,054983 | Sept10 |
| ENSRNOG00000003840 | 13,20 | 0,82 | 0,054983 | Slit2 |
| ENSRNOG00000016818 | 61,79 | -0,55 | 0,055244 | Fgfr3 |
| ENSRNOG00000006378 | 164,96 | 0,53 | 0,055399 | Mga |
| ENSRNOG00000004309 | 147,44 | 0,49 | 0,055399 |  |
| ENSRNOG00000001254 | 469,39 | 0,35 | 0,055399 | Col6a2 |
| ENSRNOG00000047768 | 1485,23 | -0,36 | 0,055549 | Lamb2 |
| ENSRNOG00000017286 | 132,85 | -0,50 | 0,055687 | Ephx2 |
| ENSRNOG00000007315 | 129,82 | 0,44 | 0,055842 | Thoc2 |
| ENSRNOG00000000498 | 446,19 | -0,35 | 0,055842 | Anks1a |
| ENSRNOG00000049912 | 780,06 | -0,54 | 0,055842 | Atp5e |
| ENSRNOG00000014050 | 132,11 | -0,44 | 0,056131 | Ptges2 |
| ENSRNOG00000031127 | 20,42 | 0,72 | 0,056265 | Snrpe |
| ENSRNOG00000023152 | 31,52 | -0,73 | 0,056344 | Tmem201 |
| ENSRNOG00000048915 | 459,20 | -0,42 | 0,056365 | Twf2 |
| ENSRNOG00000028872 | 102,34 | 0,51 | 0,056365 | Rai14 |
| ENSRNOG00000019425 | 92,56 | 0,44 | 0,056449 | Gabarapl2 |
| ENSRNOG00000037673 | 7,33 | 0,79 | 0,057023 |  |
| ENSRNOG00000048264 | 11,58 | -0,79 | 0,057129 | LOC684828 |
| ENSRNOG00000001203 | 38,52 | -0,63 | 0,057667 | Rrp1 |
| ENSRNOG00000004918 | 13,03 | 0,83 | 0,057948 | Kcna4 |
| ENSRNOG00000017832 | 133,11 | 0,46 | 0,057978 | Snx2 |
| ENSRNOG00000034272 | 46,09 | 0,54 | 0,058439 | Pias1 |
| ENSRNOG00000014287 | 229,15 | -0,52 | 0,058549 | Stk11 |
| ENSRNOG00000011566 | 35,04 | 0,61 | 0,058601 | Cecr2 |
| ENSRNOG00000016047 | 1213,67 | 0,41 | 0,058648 | Macf1 |
| ENSRNOG00000014720 | 28,02 | 0,75 | 0,059097 | Srbd1 |
| ENSRNOG00000004686 | 387,35 | -0,31 | 0,059097 | Spop |
| ENSRNOG00000000803 | 88,31 | 0,54 | 0,059097 |  |
| ENSRNOG00000048101 | 5,07 | -0,83 | 0,059391 | Zfp397 |
| ENSRNOG00000006766 | 142,88 | -0,49 | 0,059576 | Laptm4b |
| ENSRNOG00000015516 | 14,31 | 0,80 | 0,060092 | Cbwd1 |
| ENSRNOG00000014815 | 2829,44 | -0,23 | 0,060092 | Myoz2 |
| ENSRNOG00000002277 | 378,89 | 0,36 | 0,060183 |  |
| ENSRNOG00000003667 | 262,65 | 0,54 | 0,060626 |  |
| ENSRNOG00000026277 | 25,26 | 0,71 | 0,060626 | Zc3h6 |
| ENSRNOG00000004262 | 6,02 | 0,64 | 0,060626 | LOC102546716 |
| ENSRNOG00000021526 | 95,92 | -0,55 | 0,060643 | Slc25a34 |
| ENSRNOG00000005938 | 73,05 | -0,60 | 0,060912 |  |
| ENSRNOG00000010980 | 44,03 | 0,71 | 0,060935 | Proser1 |
| ENSRNOG00000003917 | 10,31 | -0,82 | 0,060935 | Uck2 |
| ENSRNOG00000012438 | 20,25 | 0,79 | 0,060935 | Larp6 |
| ENSRNOG00000018118 | 65,17 | -0,51 | 0,060942 | Atad3a |
| ENSRNOG00000010800 | 2204,82 | -0,47 | 0,060942 | Hadhb |
| ENSRNOG00000016423 | 460,40 | 0,33 | 0,060942 | Tacc1 |
| ENSRNOG00000025053 | 479,65 | 0,53 | 0,06096 | Lrp1 |
| ENSRNOG00000045738 | 122,47 | -0,48 | 0,06096 | Ak4 |
| ENSRNOG00000003357 | 1994,79 | 0,61 | 0,061427 | Col3a1 |
| ENSRNOG00000000851 | 522,18 | -0,26 | 0,061527 | Bag6 |
| ENSRNOG00000020423 | 195,03 | 0,51 | 0,061527 | Apc |
| ENSRNOG00000016963 | 472,91 | 0,31 | 0,061692 | Trip12 |
| ENSRNOG00000031299 | 4,61 | -0,81 | 0,062251 |  |
| ENSRNOG00000018211 | 114,65 | -0,56 | 0,062571 | Urod |
| ENSRNOG00000006749 | 101,23 | 0,52 | 0,062783 | Tmtc3 |
| ENSRNOG00000017852 | 189,19 | 0,41 | 0,062783 | Nars |
| ENSRNOG00000050024 | 5,73 | 0,76 | 0,062783 | Ms4a4a |
| ENSRNOG00000002593 | 68,42 | 0,56 | 0,062968 | Esrrg |
| ENSRNOG00000032840 | 8,78 | 0,82 | 0,062999 | Sumo4 |
| ENSRNOG00000001559 | 113,35 | -0,46 | 0,063127 | Mtx2 |
| ENSRNOG00000013884 | 29,02 | 0,77 | 0,063263 | Psd3 |
| ENSRNOG00000008598 | 57,25 | 0,62 | 0,063678 | Btbd7 |
| ENSRNOG00000015389 | 100,57 | 0,55 | 0,063803 | Stag1 |
| ENSRNOG00000039463 | 5,81 | 0,77 | 0,063803 |  |
| ENSRNOG00000012379 | 72,83 | -0,67 | 0,063803 | Wdr18 |
| ENSRNOG00000014137 | 166,31 | 0,54 | 0,06406 | Fbln1 |
| ENSRNOG00000010947 | 65,77 | 0,66 | 0,064254 | Mmp14 |
| ENSRNOG00000037514 | 81,70 | 0,53 | 0,064454 | Qser1 |
| ENSRNOG00000025997 | 180,60 | 0,33 | 0,064498 | Mrrf |
| ENSRNOG00000020878 | 78,95 | -0,43 | 0,064654 | Keap1 |
| ENSRNOG00000019407 | 33,20 | -0,64 | 0,064953 | Dmap1 |
| ENSRNOG00000015461 | 169,33 | 0,50 | 0,065366 | Serpine2 |
| ENSRNOG00000017249 | 44,22 | 0,73 | 0,065401 | Zfp366 |
| ENSRNOG00000026050 | 22,33 | -0,74 | 0,065419 | Epb41l4a |
| ENSRNOG00000020310 | 14,61 | -0,75 | 0,065606 | Grik5 |
| ENSRNOG00000033641 | 19,58 | 0,81 | 0,065606 |  |
| ENSRNOG00000002538 | 8,59 | 0,81 | 0,065606 | Epb41l5 |
| ENSRNOG00000013647 | 7,55 | -0,81 | 0,065606 | Polm |
| ENSRNOG00000003248 | 76,07 | 0,65 | 0,065606 | Mpzl1 |
| ENSRNOG00000047499 | 37,06 | -0,60 | 0,065606 | Rbm15 |
| ENSRNOG00000008289 | 4544,50 | -0,24 | 0,065606 | Slc25a3 |
| ENSRNOG00000005904 | 84,17 | 0,57 | 0,065748 | Cdc27 |
| ENSRNOG00000014369 | 26,07 | -0,70 | 0,066066 | Slc27a4 |
| ENSRNOG00000020837 | 59,93 | -0,64 | 0,066066 | Cd300lg |
| ENSRNOG00000016257 | 11,25 | 0,80 | 0,066066 | Cotl1 |
| ENSRNOG00000005772 | 8,02 | 0,81 | 0,066066 | Hacd4 |
| ENSRNOG00000003268 | 62,20 | 0,57 | 0,066066 | Maml1 |
| ENSRNOG00000006116 | 137,79 | -0,42 | 0,066066 | Hk2 |
| ENSRNOG00000003234 | 432,59 | -0,40 | 0,066066 | Mgrn1 |
| ENSRNOG00000001653 | 129,52 | -0,43 | 0,066088 | St3gal6 |
| ENSRNOG00000013285 | 89,18 | -0,44 | 0,066131 | Imp4 |
| ENSRNOG00000009783 | 53,38 | 0,66 | 0,066131 | Camk2g |
| ENSRNOG00000047046 | 361,97 | -0,56 | 0,066131 |  |
| ENSRNOG00000015634 | 83,19 | 0,49 | 0,066458 |  |
| ENSRNOG00000012879 | 7298,20 | -0,33 | 0,066458 | Fabp3 |
| ENSRNOG00000009889 | 472,74 | -0,29 | 0,066458 | Pgm1 |
| ENSRNOG00000012354 | 87,65 | 0,41 | 0,066458 | Trim23 |
| ENSRNOG00000019459 | 596,88 | -0,41 | 0,066458 | Oaz1 |
| ENSRNOG00000012074 | 415,47 | 0,47 | 0,066516 | Ifngr1 |
| ENSRNOG00000017567 | 28,48 | -0,71 | 0,066629 |  |
| ENSRNOG00000022943 | 21,12 | -0,75 | 0,066629 | Dgka |
| ENSRNOG00000007457 | 411,06 | 0,42 | 0,066636 | Serping1 |
| ENSRNOG00000020298 | 1256,16 | -0,23 | 0,066636 | Bag3 |
| ENSRNOG00000013717 | 42,02 | 0,61 | 0,066636 | Bmp6 |
| ENSRNOG00000012017 | 64,01 | 0,46 | 0,066636 | Otulin |
| ENSRNOG00000037268 | 87,10 | 0,51 | 0,067069 |  |
| ENSRNOG00000022736 | 28,65 | 0,60 | 0,067129 | Cdkn2aip |
| ENSRNOG00000010895 | 133,20 | 0,45 | 0,067157 | Tmem30a |
| ENSRNOG00000021005 | 174,50 | -0,42 | 0,067413 | Mrpl16 |
| ENSRNOG00000017133 | 263,09 | -0,47 | 0,067413 | LOC306766 |
| ENSRNOG00000028302 | 80,57 | 0,44 | 0,067413 | Smarcb1 |
| ENSRNOG00000034066 | 987,74 | -0,33 | 0,067413 | Hspa8 |
| ENSRNOG00000018281 | 1853,98 | -0,26 | 0,0678 | Uqcrfs1 |
| ENSRNOG00000000565 | 25,46 | 0,74 | 0,067841 | Sgpl1 |
| ENSRNOG00000020029 | 92,98 | -0,55 | 0,067885 | Mcrip2 |
| ENSRNOG00000033824 | 43,20 | 0,57 | 0,068214 | Gpd2 |
| ENSRNOG00000022812 | 76,60 | 0,55 | 0,068485 | Ercc5 |
| ENSRNOG00000050348 | 51,39 | -0,63 | 0,068555 | LOC684270 |
| ENSRNOG00000026616 | 229,72 | -0,54 | 0,068555 | Ndufb2 |
| ENSRNOG00000003403 | 261,83 | -0,39 | 0,068555 | Slc35f5 |
| ENSRNOG00000001215 | 12,39 | -0,77 | 0,068555 | RGD1309594 |
| ENSRNOG00000004496 | 777,14 | 0,43 | 0,068555 | Rock2 |
| ENSRNOG00000036934 | 80,62 | -0,44 | 0,068555 | Abhd12 |
| ENSRNOG00000015479 | 81,58 | -0,56 | 0,068706 | Mrps34 |
| ENSRNOG00000047433 | 30,94 | 0,73 | 0,068706 |  |
| ENSRNOG00000002210 | 67,38 | 0,46 | 0,068706 | Hsd17b11 |
| ENSRNOG00000018207 | 19,59 | 0,76 | 0,068706 | Dynlt1 |
| ENSRNOG00000019424 | 8,37 | 0,79 | 0,068706 | Aspdh |
| ENSRNOG00000000432 | 19,26 | -0,75 | 0,068742 | Fkbpl |
| ENSRNOG00000000489 | 278,26 | -0,38 | 0,068755 |  |
| ENSRNOG00000028278 | 51,80 | 0,53 | 0,068866 |  |
| ENSRNOG00000004831 | 99,88 | 0,57 | 0,069409 | Arid2 |
| ENSRNOG00000048580 | 12,41 | -0,78 | 0,069529 | Trip6 |
| ENSRNOG00000017311 | 241,75 | -0,36 | 0,069529 | Me3 |
| ENSRNOG00000008256 | 128,89 | -0,48 | 0,069591 | Mrpl38 |
| ENSRNOG00000019036 | 168,42 | -0,45 | 0,069591 | Ldhd |
| ENSRNOG00000020363 | 93,41 | -0,41 | 0,069694 | Med25 |
| ENSRNOG00000025174 | 174,22 | 0,50 | 0,069694 | Kat6a |
| ENSRNOG00000017548 | 36,52 | -0,60 | 0,069717 | Fam53a |
| ENSRNOG00000011491 | 145,77 | 0,51 | 0,069717 | Dnajc13 |
| ENSRNOG00000038146 | 12,93 | 0,78 | 0,069867 |  |
| ENSRNOG00000021042 | 254,73 | -0,45 | 0,069886 | Psmd4 |
| ENSRNOG00000025476 | 148,72 | 0,70 | 0,070478 | Tmem252 |
| ENSRNOG00000020345 | 88,39 | -0,44 | 0,070478 | Slc25a42 |
| ENSRNOG00000019180 | 56,44 | 0,55 | 0,070478 | Acsl4 |
| ENSRNOG00000008787 | 97,24 | 0,45 | 0,070478 |  |
| ENSRNOG00000020178 | 120,29 | -0,62 | 0,070581 | Cope |
| ENSRNOG00000018886 | 6,50 | -0,80 | 0,070581 | Aaed1 |
| ENSRNOG00000012329 | 204,38 | -0,50 | 0,070581 | Saraf |
| ENSRNOG00000029614 | 11,98 | 0,80 | 0,070581 | Robo1 |
| ENSRNOG00000001720 | 77,02 | -0,54 | 0,070581 | Hes1 |
| ENSRNOG00000026963 | 1139,28 | 0,26 | 0,071836 | Hsp90b1 |
| ENSRNOG00000019930 | 122,62 | -0,50 | 0,071904 | Rhot2 |
| ENSRNOG00000008037 | 56,32 | -0,59 | 0,072071 | Tmed1 |
| ENSRNOG00000022910 | 160,15 | 0,41 | 0,072144 | Emcn |
| ENSRNOG00000049985 | 52,47 | -0,58 | 0,072144 | Gprasp1 |
| ENSRNOG00000018668 | 275,57 | 0,45 | 0,072517 | Glg1 |
| ENSRNOG00000012671 | 6,96 | 0,79 | 0,072517 | Gan |
| ENSRNOG00000028629 | 342,93 | -0,27 | 0,072794 | Akt1 |
| ENSRNOG00000010780 | 308,04 | 0,29 | 0,072806 | Dlc1 |
| ENSRNOG00000028430 | 15,12 | 0,69 | 0,072806 | LOC257650 |
| ENSRNOG00000007432 | 37,66 | -0,59 | 0,072806 | Polr3b |
| ENSRNOG00000011129 | 80,57 | 0,53 | 0,072853 |  |
| ENSRNOG00000011936 | 18,23 | -0,74 | 0,072965 | Abhd14a |
| ENSRNOG00000015655 | 246,81 | 0,43 | 0,073232 | Ptgfrn |
| ENSRNOG00000051011 | 3,76 | 0,76 | 0,073261 |  |
| ENSRNOG00000008816 | 79,82 | 0,74 | 0,07334 | Gpnmb |
| ENSRNOG00000007047 | 47,64 | 0,67 | 0,07334 | Eps8 |
| ENSRNOG00000004282 | 55,10 | 0,59 | 0,073525 | Arl4a |
| ENSRNOG00000004300 | 22,97 | 0,66 | 0,073551 | Gtf2a1 |
| ENSRNOG00000011899 | 94,01 | -0,49 | 0,073554 |  |
| ENSRNOG00000027012 | 118,56 | 0,51 | 0,073814 | Usp54 |
| ENSRNOG00000020353 | 78,66 | 0,57 | 0,074045 | Sh3pxd2a |
| ENSRNOG00000025843 | 13,73 | -0,74 | 0,074194 | Ccdc102a |
| ENSRNOG00000038625 | 4,41 | -0,78 | 0,074351 | Sbk2 |
| ENSRNOG00000018009 | 21,06 | 0,78 | 0,074351 | Rab8b |
| ENSRNOG00000010116 | 43,81 | -0,65 | 0,074351 | Leo1 |
| ENSRNOG00000008075 | 23,77 | 0,68 | 0,074357 | Ift74 |
| ENSRNOG00000036911 | 11,99 | 0,75 | 0,074372 | Bicd1 |
| ENSRNOG00000006787 | 15,33 | 0,78 | 0,074377 | Dhcr24 |
| ENSRNOG00000024578 | 10,48 | 0,79 | 0,074454 | Ttyh2 |
| ENSRNOG00000023446 | 10,74 | 0,77 | 0,074911 | Btbd8 |
| ENSRNOG00000005326 | 20,49 | 0,71 | 0,074911 | Cnrip1 |
| ENSRNOG00000005413 | 28,39 | 0,68 | 0,075071 | Creb3l1 |
| ENSRNOG00000013456 | 21,90 | -0,68 | 0,075087 | Ighmbp2 |
| ENSRNOG00000000635 | 49,09 | 0,59 | 0,075185 |  |
| ENSRNOG00000000437 | 217,62 | -0,35 | 0,075236 | Agpat1 |
| ENSRNOG00000013828 | 55,74 | -0,56 | 0,075455 | Sirt3 |
| ENSRNOG00000017441 | 184,02 | 0,46 | 0,07593 | Tpm3 |
| ENSRNOG00000027901 | 13,61 | -0,79 | 0,076082 | Mrm1 |
| ENSRNOG00000003063 | 31,32 | -0,59 | 0,076203 | Phka1 |
| ENSRNOG00000021255 | 3,65 | -0,74 | 0,076203 | Smox |
| ENSRNOG00000011216 | 203,15 | 0,37 | 0,076601 | Tbl1xr1 |
| ENSRNOG00000011189 | 69,98 | -0,53 | 0,07671 | Acy1 |
| ENSRNOG00000023383 | 802,26 | 0,28 | 0,07671 | Ddx3x |
| ENSRNOG00000004273 | 110,94 | 0,53 | 0,076726 | Ifitm1 |
| ENSRNOG00000010427 | 424,49 | 0,39 | 0,076831 | Ipo7 |
| ENSRNOG00000030910 | 5,37 | -0,77 | 0,076993 | Grik4 |
| ENSRNOG00000019333 | 466,02 | 0,34 | 0,077013 | Hipk1 |
| ENSRNOG00000006987 | 15,80 | 0,76 | 0,077013 | Tmem263 |
| ENSRNOG00000025071 | 130,41 | 0,44 | 0,077201 | Qrich1 |
| ENSRNOG00000005330 | 191,99 | 0,50 | 0,077201 | Crebbp |
| ENSRNOG00000002112 | 81,44 | 0,46 | 0,077201 | Zfp644 |
| ENSRNOG00000020994 | 273,23 | -0,39 | 0,077298 | Slc25a39 |
| ENSRNOG00000018552 | 37,03 | -0,55 | 0,077533 | Slc25a38 |
| ENSRNOG00000001500 | 40,78 | 0,73 | 0,077551 | Rab4b |
| ENSRNOG00000008218 | 89,85 | -0,57 | 0,077551 | Atp6v0e2 |
| ENSRNOG00000021565 | 52,32 | -0,58 | 0,077655 |  |
| ENSRNOG00000014582 | 129,78 | -0,44 | 0,07794 | Mrpl18 |
| ENSRNOG00000008843 | 388,63 | -0,41 | 0,0783 | Eci1 |
| ENSRNOG00000014163 | 9,02 | -0,78 | 0,078486 | Zfp536 |
| ENSRNOG00000028611 | 6,97 | 0,76 | 0,078486 |  |
| ENSRNOG00000003486 | 38,17 | 0,54 | 0,078743 | Mnda |
| ENSRNOG00000047250 | 270,47 | 0,31 | 0,078743 | Gmfb |
| ENSRNOG00000037638 | 195,77 | -0,36 | 0,078743 | Timm50 |
| ENSRNOG00000008329 | 655,31 | -0,41 | 0,078743 | Ndufb11 |
| ENSRNOG00000012820 | 216,12 | 0,43 | 0,078743 | Add3 |
| ENSRNOG00000016578 | 49,91 | -0,57 | 0,078743 | Mtpap |
| ENSRNOG00000011416 | 31,28 | 0,67 | 0,078954 | Vegfc |
| ENSRNOG00000021866 | 179,60 | -0,36 | 0,079121 | Bola3 |
| ENSRNOG00000003730 | 121,36 | 0,51 | 0,079284 |  |
| ENSRNOG00000010771 | 272,22 | 0,46 | 0,079698 | Pkd1 |
| ENSRNOG00000008159 | 40,74 | 0,59 | 0,079765 | Msantd3 |
| ENSRNOG00000011969 | 350,34 | 0,44 | 0,079942 | Dock9 |
| ENSRNOG00000014087 | 108,97 | -0,40 | 0,080136 | Kifc3 |
| ENSRNOG00000032463 | 431,57 | 0,33 | 0,080136 | Rap1a |
| ENSRNOG00000047656 | 227,97 | -0,37 | 0,080626 | Gaa |
| ENSRNOG00000020947 | 89,43 | -0,49 | 0,080808 | Egln2 |
| ENSRNOG00000005667 | 25,87 | 0,62 | 0,081116 |  |
| ENSRNOG00000008416 | 88,28 | 0,60 | 0,081279 | Gimap5 |
| ENSRNOG00000018529 | 183,39 | -0,44 | 0,081508 | Csnk1g2 |
| ENSRNOG00000016254 | 45,94 | 0,57 | 0,081584 | Sema4c |
| ENSRNOG00000046654 | 61,78 | -0,60 | 0,081636 |  |
| ENSRNOG00000006779 | 50,29 | 0,54 | 0,081739 | Crot |
| ENSRNOG00000006267 | 54,62 | 0,63 | 0,081749 |  |
| ENSRNOG00000009110 | 94,44 | 0,49 | 0,081749 | Psen1 |
| ENSRNOG00000013314 | 16,06 | 0,75 | 0,082239 | Avl9 |
| ENSRNOG00000042829 | 48,85 | -0,67 | 0,082524 |  |
| ENSRNOG00000009514 | 10,29 | 0,78 | 0,082714 | Mme |
| ENSRNOG00000042916 | 96,85 | 0,59 | 0,082979 | Rwdd1 |
| ENSRNOG00000020297 | 77,55 | 0,52 | 0,082979 | Gon4l |
| ENSRNOG00000025731 | 12,90 | 0,73 | 0,083229 | LOC100359583 |
| ENSRNOG00000050636 | 74,53 | -0,54 | 0,083477 |  |
| ENSRNOG00000010487 | 11,02 | 0,77 | 0,083477 |  |
| ENSRNOG00000015133 | 143,21 | 0,61 | 0,083477 | Kmt2a |
| ENSRNOG00000002541 | 113,76 | 0,47 | 0,083477 | Pds5a |
| ENSRNOG00000005998 | 5,97 | 0,66 | 0,083477 | Smoc1 |
| ENSRNOG00000013055 | 73,70 | 0,56 | 0,083477 | Zfyve16 |
| ENSRNOG00000009341 | 12,69 | 0,77 | 0,084005 | Hivep3 |
| ENSRNOG00000043085 | 24,80 | 0,74 | 0,084069 | Clstn2 |
| ENSRNOG00000017108 | 18,18 | -0,72 | 0,084081 | Syngr1 |
| ENSRNOG00000008245 | 69,66 | 0,52 | 0,084081 | Ptgis |
| ENSRNOG00000030469 | 4,91 | 0,71 | 0,084542 |  |
| ENSRNOG00000008214 | 55,89 | 0,50 | 0,084848 | Fbxo9 |
| ENSRNOG00000050585 | 436,84 | -0,25 | 0,085006 | Pgam1 |
| ENSRNOG00000009805 | 51,14 | 0,58 | 0,085026 | Wasf2 |
| ENSRNOG00000005618 | 67,04 | -0,62 | 0,085188 | Fmc1 |
| ENSRNOG00000000246 | 125,25 | -0,44 | 0,085188 | Amz2 |
| ENSRNOG00000020623 | 49,84 | -0,59 | 0,085188 | Aldh16a1 |
| ENSRNOG00000010566 | 16,18 | 0,75 | 0,085384 | Cep44 |
| ENSRNOG00000019850 | 296,95 | -0,30 | 0,085447 | Speg |
| ENSRNOG00000009250 | 14,36 | 0,72 | 0,085447 | Pts |
| ENSRNOG00000039183 | 3,90 | 0,63 | 0,085541 | RGD1310335 |
| ENSRNOG00000013712 | 120,17 | -0,41 | 0,085541 | Tex261 |
| ENSRNOG00000027773 | 100,88 | 0,48 | 0,085541 | Ppp4r3a |
| ENSRNOG00000000845 | 150,85 | -0,45 | 0,085541 |  |
| ENSRNOG00000013579 | 35,40 | 0,68 | 0,085721 | LOC100359916 |
| ENSRNOG00000029582 | 5,60 | 0,76 | 0,085721 | Lrch2 |
| ENSRNOG00000015658 | 984,78 | 0,41 | 0,085721 | Sorbs1 |
| ENSRNOG00000047605 | 173,04 | 0,38 | 0,085721 | Ptprk |
| ENSRNOG00000048114 | 29,61 | -0,69 | 0,085834 |  |
| ENSRNOG00000005331 | 174,35 | -0,39 | 0,085875 | Vapb |
| ENSRNOG00000024671 | 189,54 | 0,48 | 0,085887 | Dmxl1 |
| ENSRNOG00000010832 | 15,68 | 0,72 | 0,085932 | Pdgfrl |
| ENSRNOG00000017075 | 3,65 | -0,73 | 0,085932 | Slc35e2b |
| ENSRNOG00000020705 | 8,11 | -0,76 | 0,086194 | Rnls |
| ENSRNOG00000049585 | 11,49 | 0,75 | 0,086224 |  |
| ENSRNOG00000020707 | 78,77 | 0,53 | 0,086224 |  |
| ENSRNOG00000010748 | 2038,50 | 0,24 | 0,086397 | Mtus1 |
| ENSRNOG00000006778 | 9,08 | 0,77 | 0,086659 | Mmp19 |
| ENSRNOG00000014135 | 6,96 | 0,76 | 0,086761 | Rab11fip4 |
| ENSRNOG00000004980 | 166,60 | -0,46 | 0,086833 | Rangrf |
| ENSRNOG00000021569 | 9,84 | 0,65 | 0,086833 | Tiam1 |
| ENSRNOG00000023473 | 11,90 | 0,75 | 0,086833 |  |
| ENSRNOG00000028225 | 497,42 | -0,32 | 0,086962 | Tnni3k |
| ENSRNOG00000008622 | 25,84 | 0,67 | 0,087253 | Creb5 |
| ENSRNOG00000018964 | 21,47 | -0,67 | 0,087257 | Gss |
| ENSRNOG00000009987 | 164,77 | 0,34 | 0,087392 | Akap11 |
| ENSRNOG00000020955 | 17773,10 | -0,32 | 0,087392 | Myl3 |
| ENSRNOG00000012975 | 28,26 | -0,60 | 0,087392 | Ggcx |
| ENSRNOG00000007548 | 63,84 | 0,52 | 0,087392 | Polr3f |
| ENSRNOG00000043123 | 63,85 | -0,46 | 0,087446 |  |
| ENSRNOG00000002275 | 94,62 | 0,37 | 0,087619 | Fip1l1 |
| ENSRNOG00000020782 | 45,49 | 0,49 | 0,087999 | Pspc1 |
| ENSRNOG00000010888 | 10,05 | -0,76 | 0,089115 | Ankrd33b |
| ENSRNOG00000008016 | 161,90 | -0,36 | 0,089637 | Ckap4 |
| ENSRNOG00000022847 | 63,19 | 0,60 | 0,089637 | Dopey1 |
| ENSRNOG00000005387 | 149,54 | 0,42 | 0,089727 | Rbm3 |
| ENSRNOG00000017863 | 231,12 | 0,35 | 0,089858 | Zeb1 |
| ENSRNOG00000018989 | 140,62 | -0,40 | 0,089872 |  |
| ENSRNOG00000024629 | 1983,98 | -0,25 | 0,090137 | Hadha |
| ENSRNOG00000005334 | 39,93 | 0,72 | 0,090432 | Acvr2a |
| ENSRNOG00000003792 | 80,67 | 0,50 | 0,090432 | Med14 |
| ENSRNOG00000016109 | 121,15 | -0,37 | 0,09055 | Neurl4 |
| ENSRNOG00000026679 | 443,37 | -0,43 | 0,090648 | Scn4b |
| ENSRNOG00000009094 | 606,50 | -0,40 | 0,091188 | Nudt4 |
| ENSRNOG00000007324 | 249,61 | 0,44 | 0,091188 | Plxna2 |
| ENSRNOG00000010077 | 209,81 | -0,35 | 0,091188 | Smarcd3 |
| ENSRNOG00000007997 | 99,68 | 0,56 | 0,091214 |  |
| ENSRNOG00000002721 | 73,01 | -0,51 | 0,091214 | Ndufb4 |
| ENSRNOG00000004812 | 96,91 | 0,56 | 0,091357 | Sema6d |
| ENSRNOG00000045765 | 153,65 | -0,36 | 0,091382 | MGC94207 |
| ENSRNOG00000007104 | 197,61 | 0,42 | 0,09198 | Itpr1 |
| ENSRNOG00000031031 | 99,72 | 0,47 | 0,092126 | Zfp292 |
| ENSRNOG00000023803 | 3237,98 | 0,30 | 0,092311 | Cmya5 |
| ENSRNOG00000005292 | 204,44 | 0,46 | 0,092321 | Trip11 |
| ENSRNOG00000030213 | 156,19 | 0,44 | 0,092887 | Vps13c |
| ENSRNOG00000005711 | 59,59 | 0,56 | 0,093158 | Ptprd |
| ENSRNOG00000000922 | 16,72 | 0,72 | 0,093514 | Sumf2 |
| ENSRNOG00000034037 | 180,55 | 0,42 | 0,093614 | Zfp266 |
| ENSRNOG00000018099 | 82,40 | 0,58 | 0,093614 |  |
| ENSRNOG00000016945 | 186,73 | 0,50 | 0,093614 | Pla2g2a |
| ENSRNOG00000010849 | 36,91 | -0,52 | 0,093807 | Adprhl2 |
| ENSRNOG00000016923 | 121,48 | -0,41 | 0,093807 | Clptm1l |
| ENSRNOG00000018665 | 14,49 | -0,71 | 0,09417 | Bud13 |
| ENSRNOG00000024309 | 465,65 | -0,30 | 0,094192 | LOC688869 |
| ENSRNOG00000026649 | 57,42 | 0,56 | 0,094192 | Dnmt3a |
| ENSRNOG00000001866 | 143,59 | 0,46 | 0,09426 |  |
| ENSRNOG00000018457 | 151,40 | -0,36 | 0,09426 | Ptpa |
| ENSRNOG00000010593 | 75,71 | -0,47 | 0,094781 | Ctnnal1 |
| ENSRNOG00000022681 | 38,70 | -0,64 | 0,095158 | RGD1561113 |
| ENSRNOG00000047386 | 189,88 | 0,56 | 0,095158 |  |
| ENSRNOG00000017446 | 484,06 | -0,46 | 0,095368 | Ndufs8 |
| ENSRNOG00000019536 | 113,42 | -0,54 | 0,095368 | Smim3 |
| ENSRNOG00000001843 | 90,31 | 0,59 | 0,09592 | Bcl6 |
| ENSRNOG00000049327 | 33,00 | 0,59 | 0,096303 |  |
| ENSRNOG00000006419 | 46,02 | -0,50 | 0,096344 | Aven |
| ENSRNOG00000025539 | 81,76 | 0,60 | 0,096344 | Vps13a |
| ENSRNOG00000027049 | 1129,45 | -0,44 | 0,096344 | LOC689271 |
| ENSRNOG00000019393 | 138,50 | 0,45 | 0,096422 |  |
| ENSRNOG00000007081 | 494,38 | 0,34 | 0,096787 | Xdh |
| ENSRNOG00000002926 | 169,52 | 0,50 | 0,096787 | Uap1l2 |
| ENSRNOG00000003990 | 49,42 | -0,52 | 0,097405 | Grb2 |
| ENSRNOG00000003493 | 14,36 | 0,75 | 0,097828 |  |
| ENSRNOG00000006435 | 48,52 | 0,54 | 0,097955 | Tor1b |
| ENSRNOG00000018873 | 81,20 | 0,58 | 0,09799 | Fam168a |
| ENSRNOG00000031938 | 19,93 | 0,62 | 0,098099 |  |
| ENSRNOG00000016372 | 723,24 | -0,32 | 0,098295 | Slc12a7 |
| ENSRNOG00000016867 | 31,17 | -0,65 | 0,098407 | Zfp346 |
| ENSRNOG00000017874 | 45,59 | 0,67 | 0,098457 | Cd53 |
| ENSRNOG00000018706 | 13,10 | 0,71 | 0,098657 | Il15ra |
| ENSRNOG00000003879 | 212,92 | -0,29 | 0,098657 | Rnf167 |
| ENSRNOG00000011952 | 572,76 | -0,39 | 0,098989 | Samm50 |
| ENSRNOG00000034140 | 9,28 | 0,75 | 0,098989 |  |
| ENSRNOG00000025028 | 68,07 | 0,49 | 0,098989 | Prkdc |
| ENSRNOG00000013140 | 239,17 | 0,48 | 0,099149 | Pdzd2 |
| ENSRNOG00000038489 | 169,53 | 0,42 | 0,099149 | Sumo3 |
| ENSRNOG00000010549 | 111,24 | -0,50 | 0,099609 | Tspo |
| ENSRNOG00000050949 | 63,06 | -0,50 | 0,099657 | Ttc39c |
| ENSRNOG00000002871 | 60,88 | 0,65 | 0,099657 | Rbm25l1 |
| ENSRNOG00000014610 | 37,91 | 0,69 | 0,099657 | Anpep |
| ENSRNOG00000028241 | 133,22 | -0,43 | 0,099657 |  |
| ENSRNOG00000001930 | 41,43 | 0,56 | 0,099687 | Ccdc50 |
| ENSRNOG00000038449 | 12,49 | 0,72 | 0,099687 | Tigd2 |
| ENSRNOG00000024019 | 17,61 | 0,66 | 0,099687 | Ccdc6 |
| ENSRNOG00000026646 | 135,86 | -0,45 | 0,099687 | Ndufs5 |
| ENSRNOG00000002248 | 129,58 | 0,50 | 0,099806 | Fryl |
| ENSRNOG00000005577 | 52,70 | -0,55 | 0,099978 | Desi1 |
| ENSRNOG00000013301 | 16,86 | -0,67 | 0,099978 | Bean1 |
| ENSRNOG00000008414 | 1382,05 | -0,31 | 0,099978 | Bsg |
| ENSRNOG00000008301 | 481,20 | 0,43 | 0,099978 | Tagln2 |

**Supplemental table 5:** Differentially expressed genes in obese + DS versus obese rats.

| **ID** | **baseMean** | **log2FoldChange** | **FDR** | **symbol** |
| --- | --- | --- | --- | --- |
| ENSRNOG00000012094 | 105,53 | 2,80 | 4,75E-26 | Ltbp2 |
| ENSRNOG00000031890 | 85,16 | 2,04 | 5,76E-18 | Ncam1 |
| ENSRNOG00000017440 | 1053,30 | 1,46 | 1,35E-17 |  |
| ENSRNOG00000003357 | 2187,05 | 1,72 | 8,96E-17 | Col3a1 |
| ENSRNOG00000012840 | 3519,52 | 0,79 | 1,05E-15 | Sparc |
| ENSRNOG00000034258 | 8754,70 | 1,44 | 1,95E-15 | Xirp2 |
| ENSRNOG00000049070 | 21,60 | 3,01 | 7,23E-15 |  |
| ENSRNOG00000014288 | 566,95 | 1,68 | 7,82E-15 | Fn1 |
| ENSRNOG00000015488 | 124,36 | 1,50 | 2,25E-14 | Tead1 |
| ENSRNOG00000007034 | 854,86 | 1,02 | 4,13E-14 | Hipk2 |
| ENSRNOG00000004610 | 437,17 | 1,21 | 7,83E-14 | Lum |
| ENSRNOG00000011292 | 385,52 | 1,53 | 1,06E-13 | Col1a2 |
| ENSRNOG00000025757 | 20272,21 | -1,37 | 2,09E-13 | Myh6 |
| ENSRNOG00000010183 | 233,97 | 1,12 | 1,02E-12 | Fam198b |
| ENSRNOG00000005695 | 1109,76 | 1,20 | 1,33E-12 | Mgp |
| ENSRNOG00000008176 | 3789,14 | 2,40 | 2,13E-12 | Nppa |
| ENSRNOG00000012516 | 1424,37 | 1,06 | 2,34E-12 |  |
| ENSRNOG00000026415 | 273,90 | 1,28 | 3,58E-12 | Col14a1 |
| ENSRNOG00000012660 | 224,38 | 2,33 | 6,43E-12 | Postn |
| ENSRNOG00000015461 | 201,88 | 1,03 | 2,02E-11 | Serpine2 |
| ENSRNOG00000030478 | 842215,90 | 1,95 | 2,09E-11 |  |
| ENSRNOG00000047433 | 50,71 | 2,09 | 2,29E-11 |  |
| ENSRNOG00000008843 | 556,19 | -1,06 | 3,12E-11 | Eci1 |
| ENSRNOG00000025053 | 569,02 | 0,98 | 3,57E-11 | Lrp1 |
| ENSRNOG00000000525 | 203,88 | 1,50 | 4,15E-11 | Pi16 |
| ENSRNOG00000021434 | 9,27 | 2,58 | 6,47E-11 | Usp51 |
| ENSRNOG00000021157 | 107,56 | 1,37 | 1,07E-10 |  |
| ENSRNOG00000010134 | 981,99 | -1,13 | 1,18E-10 | Acot2 |
| ENSRNOG00000015547 | 123,87 | 1,14 | 1,27E-10 |  |
| ENSRNOG00000009795 | 173,70 | 1,23 | 1,76E-10 | Nfib |
| ENSRNOG00000039668 | 87,99 | 1,73 | 2,71E-10 | Col8a1 |
| ENSRNOG00000002052 | 549,80 | 1,07 | 8,34E-10 | Ccdc80 |
| ENSRNOG00000008680 | 94,97 | 1,69 | 8,63E-10 | Loxl1 |
| ENSRNOG00000029212 | 156,75 | 1,38 | 1,29E-09 | Vcan |
| ENSRNOG00000018630 | 2771,51 | -0,72 | 2,6E-09 | Gapdh |
| ENSRNOG00000006094 | 99,74 | 1,23 | 2,9E-09 | Cd44 |
| ENSRNOG00000002050 | 836,57 | 1,02 | 4,08E-09 | Igfbp7 |
| ENSRNOG00000013917 | 57,51 | 1,86 | 4,34E-09 | Igsf10 |
| ENSRNOG00000010362 | 321,96 | 1,03 | 4,95E-09 | Anxa2 |
| ENSRNOG00000009628 | 1408,86 | -0,84 | 5,06E-09 |  |
| ENSRNOG00000010697 | 728,86 | -0,97 | 5,46E-09 | Hadh |
| ENSRNOG00000047768 | 1564,51 | -0,72 | 8,51E-09 | Lamb2 |
| ENSRNOG00000016866 | 1750,65 | -0,92 | 1,7E-08 | Fhl2 |
| ENSRNOG00000008301 | 509,15 | 0,91 | 1,95E-08 | Tagln2 |
| ENSRNOG00000010438 | 1413,28 | -0,71 | 2,45E-08 | Cpt1b |
| ENSRNOG00000010800 | 3331,28 | -0,95 | 2,55E-08 | Hadhb |
| ENSRNOG00000037815 | 191,01 | -1,01 | 3,05E-08 | Acad10 |
| ENSRNOG00000007302 | 244,63 | 1,54 | 3,51E-08 | Fbn1 |
| ENSRNOG00000032274 | 254,41 | 1,98 | 3,83E-08 |  |
| ENSRNOG00000018251 | 243,29 | 1,16 | 6,09E-08 | Mrc1 |
| ENSRNOG00000048351 | 5442,11 | 0,88 | 6,09E-08 |  |
| ENSRNOG00000007235 | 1307,80 | -0,76 | 6,26E-08 | Atp5g1 |
| ENSRNOG00000047046 | 470,07 | -0,94 | 6,81E-08 |  |
| ENSRNOG00000003172 | 148,61 | 1,01 | 6,94E-08 | Serpinf1 |
| ENSRNOG00000004205 | 150,07 | -1,10 | 6,94E-08 | Pkdcc |
| ENSRNOG00000008639 | 779,68 | 0,71 | 7,78E-08 | Pabpc1 |
| ENSRNOG00000017513 | 200,70 | -0,89 | 8,79E-08 | Miga2 |
| ENSRNOG00000020030 | 57,25 | 1,67 | 1,17E-07 | Crlf1 |
| ENSRNOG00000013532 | 1066,63 | -0,69 | 1,28E-07 | Pgam2 |
| ENSRNOG00000022196 | 412,68 | 0,91 | 1,68E-07 | Bmpr2 |
| ENSRNOG00000047931 | 1152,24 | 0,91 | 2,91E-07 | Tmsb4x |
| ENSRNOG00000018114 | 1658,76 | -0,74 | 3,29E-07 | Acadvl |
| ENSRNOG00000003736 | 287,03 | 1,09 | 3,45E-07 | Col5a2 |
| ENSRNOG00000001254 | 499,91 | 0,70 | 5,13E-07 | Col6a2 |
| ENSRNOG00000001211 | 963,00 | -0,66 | 8,55E-07 | RGD1303003 |
| ENSRNOG00000007290 | 329,19 | -0,87 | 8,7E-07 | Atp1a2 |
| ENSRNOG00000013766 | 3614,53 | -0,67 | 1,08E-06 | Acaa2 |
| ENSRNOG00000020308 | 1867,61 | -0,86 | 1,09E-06 | Ech1 |
| ENSRNOG00000017032 | 7822,17 | -0,61 | 1,1E-06 | Atp5a1 |
| ENSRNOG00000046608 | 6,13 | 2,01 | 1,18E-06 |  |
| ENSRNOG00000020423 | 223,39 | 0,91 | 1,39E-06 | Apc |
| ENSRNOG00000016151 | 533,08 | 0,86 | 1,39E-06 | Ankrd23 |
| ENSRNOG00000008173 | 66,35 | 1,28 | 1,48E-06 | Sesn3 |
| ENSRNOG00000002947 | 238,76 | 1,07 | 1,72E-06 | Dpt |
| ENSRNOG00000024629 | 2903,74 | -0,67 | 1,77E-06 | Hadha |
| ENSRNOG00000003897 | 765,59 | 1,69 | 1,95E-06 | Col1a1 |
| ENSRNOG00000005825 | 374,92 | 1,09 | 1,99E-06 | Lyz2 |
| ENSRNOG00000008816 | 120,18 | 1,49 | 2,11E-06 | Gpnmb |
| ENSRNOG00000043201 | 818,19 | -0,83 | 2,42E-06 | Coq8a |
| ENSRNOG00000020607 | 628,61 | -0,66 | 2,81E-06 | Bckdha |
| ENSRNOG00000045613 | 3976,39 | -0,67 | 2,93E-06 |  |
| ENSRNOG00000009845 | 4015,31 | -0,57 | 3,39E-06 | Acadm |
| ENSRNOG00000022637 | 8057,11 | 0,84 | 3,43E-06 |  |
| ENSRNOG00000017428 | 566,33 | 0,76 | 3,43E-06 | Map1b |
| ENSRNOG00000024128 | 6281,65 | -0,63 | 3,47E-06 | Aco2 |
| ENSRNOG00000024568 | 395,69 | -0,89 | 4,34E-06 | Ndufs7 |
| ENSRNOG00000002461 | 265,99 | 1,00 | 5,24E-06 | Nid1 |
| ENSRNOG00000012443 | 677,30 | -0,67 | 6,21E-06 | Cpt2 |
| ENSRNOG00000014153 | 208,71 | 0,88 | 6,75E-06 | Lhfp |
| ENSRNOG00000032134 | 2412,20 | -0,66 | 6,75E-06 | Uqcrc1 |
| ENSRNOG00000002244 | 239,83 | 1,10 | 7,32E-06 | Pdgfra |
| ENSRNOG00000003338 | 126,45 | 1,07 | 7,95E-06 | Pmp22 |
| ENSRNOG00000048430 | 637,96 | -0,58 | 8,23E-06 | Myo18b |
| ENSRNOG00000018087 | 1078,43 | 0,76 | 8,75E-06 | Vim |
| ENSRNOG00000008924 | 314,31 | 0,82 | 8,91E-06 | Arhgef12 |
| ENSRNOG00000006420 | 227,28 | -0,89 | 1,09E-05 | Rbm38 |
| ENSRNOG00000022781 | 245,28 | 0,81 | 1,2E-05 | Ccser2 |
| ENSRNOG00000018145 | 1470,33 | -0,76 | 1,3E-05 | Crat |
| ENSRNOG00000008289 | 4359,14 | -0,39 | 1,47E-05 | Slc25a3 |
| ENSRNOG00000005614 | 153,04 | -0,96 | 1,57E-05 | Txn2 |
| ENSRNOG00000033299 | 5354,53 | 1,64 | 1,61E-05 | ATP8 |
| ENSRNOG00000012879 | 7710,46 | -0,55 | 2,01E-05 | Fabp3 |
| ENSRNOG00000012124 | 108,58 | -0,90 | 2,01E-05 | Trappc13 |
| ENSRNOG00000028930 | 165,32 | 1,00 | 2,16E-05 | Dab2 |
| ENSRNOG00000013331 | 2105,36 | -0,53 | 2,42E-05 | Sdha |
| ENSRNOG00000012471 | 129,81 | 1,81 | 2,45E-05 | Thbs4 |
| ENSRNOG00000012749 | 148,24 | 0,99 | 2,55E-05 | C1qb |
| ENSRNOG00000009422 | 174,07 | -0,93 | 2,67E-05 | Hmgcl |
| ENSRNOG00000004516 | 97,42 | 1,19 | 2,82E-05 | Itgbl1 |
| ENSRNOG00000025174 | 208,11 | 0,76 | 2,82E-05 | Kat6a |
| ENSRNOG00000034134 | 17,71 | 1,58 | 2,82E-05 | Cpm |
| ENSRNOG00000014230 | 1061,34 | 0,52 | 3,34E-05 | Map1a |
| ENSRNOG00000007338 | 141,44 | 1,06 | 3,41E-05 | Fbln2 |
| ENSRNOG00000027434 | 713,30 | -0,58 | 3,71E-05 | Fitm2 |
| ENSRNOG00000013949 | 5015,41 | -0,45 | 4,38E-05 | Idh2 |
| ENSRNOG00000004583 | 32177,22 | -0,48 | 4,38E-05 | Mb |
| ENSRNOG00000000658 | 597,28 | -0,61 | 4,8E-05 | Acacb |
| ENSRNOG00000001030 | 731,37 | 0,57 | 4,89E-05 | Tsc22d1 |
| ENSRNOG00000013452 | 259,63 | 0,81 | 4,89E-05 | Rcn1 |
| ENSRNOG00000017866 | 202,36 | -0,81 | 4,93E-05 | Sirt5 |
| ENSRNOG00000032708 | 88,37 | 1,18 | 5,23E-05 | RT1-Bb |
| ENSRNOG00000009686 | 302,76 | -0,81 | 5,23E-05 | Aqp7 |
| ENSRNOG00000020716 | 158,00 | 0,86 | 5,26E-05 | Axl |
| ENSRNOG00000030715 | 226,32 | 0,99 | 5,3E-05 | Cfh |
| ENSRNOG00000014625 | 880,73 | -0,69 | 5,39E-05 | Atp5d |
| ENSRNOG00000016576 | 39,67 | 1,23 | 5,58E-05 | Carmil1 |
| ENSRNOG00000017307 | 166,49 | 0,78 | 5,64E-05 | Prss23 |
| ENSRNOG00000003302 | 196,14 | -0,82 | 6,05E-05 | Flcn |
| ENSRNOG00000009513 | 581,46 | -0,49 | 6,23E-05 | Akr1b1 |
| ENSRNOG00000043866 | 624259,80 | 1,40 | 6,85E-05 |  |
| ENSRNOG00000049361 | 95,53 | 1,17 | 6,89E-05 | Gas7 |
| ENSRNOG00000010510 | 56,80 | 1,35 | 6,89E-05 |  |
| ENSRNOG00000021174 | 515,88 | -0,72 | 6,96E-05 | Macrod1 |
| ENSRNOG00000015957 | 86,34 | 0,97 | 7,06E-05 | F13a1 |
| ENSRNOG00000002028 | 241,80 | -0,73 | 7,52E-05 | Tmem50b |
| ENSRNOG00000000170 | 134,93 | 0,82 | 7,88E-05 | Slc30a4 |
| ENSRNOG00000046705 | 403,31 | 0,77 | 7,88E-05 |  |
| ENSRNOG00000006738 | 76,39 | 1,14 | 7,88E-05 | Fbxo32 |
| ENSRNOG00000047860 | 121,86 | -1,14 | 7,98E-05 | Plin5 |
| ENSRNOG00000019404 | 238,24 | -0,89 | 8,16E-05 | Hhatl |
| ENSRNOG00000047137 | 261,33 | 0,76 | 8,66E-05 | Erbin |
| ENSRNOG00000010830 | 12062,40 | -0,54 | 8,86E-05 | Slc25a4 |
| ENSRNOG00000009956 | 1165,06 | 0,78 | 8,93E-05 | Wnk1 |
| ENSRNOG00000017874 | 52,46 | 1,27 | 9,49E-05 | Cd53 |
| ENSRNOG00000012002 | 315,12 | 0,62 | 9,49E-05 | Iqgap1 |
| ENSRNOG00000008336 | 17,94 | 1,65 | 9,49E-05 | Tnfrsf11b |
| ENSRNOG00000014522 | 392,81 | -0,73 | 9,49E-05 | Mlycd |
| ENSRNOG00000016837 | 4631,49 | -0,65 | 9,8E-05 | Ckm |
| ENSRNOG00000020456 | 181,03 | 0,83 | 0,00011 | Nucb2 |
| ENSRNOG00000013974 | 97,34 | -0,90 | 0,000115 | Fahd2a |
| ENSRNOG00000008749 | 160,58 | 1,16 | 0,000116 | Col5a1 |
| ENSRNOG00000003486 | 65,67 | 1,33 | 0,000121 | Mnda |
| ENSRNOG00000013786 | 428,30 | 0,67 | 0,000122 |  |
| ENSRNOG00000002176 | 28,07 | 1,48 | 0,000125 | Nectin3 |
| ENSRNOG00000002434 | 61,05 | 1,31 | 0,000127 | Tmem100 |
| ENSRNOG00000007726 | 354,60 | 0,84 | 0,000127 | Mcam |
| ENSRNOG00000046996 | 336,23 | 0,76 | 0,000128 | Pea15 |
| ENSRNOG00000026548 | 305,31 | -0,90 | 0,00013 | Dhrs7c |
| ENSRNOG00000050720 | 113,24 | 0,99 | 0,000131 |  |
| ENSRNOG00000009811 | 215,12 | 0,70 | 0,000138 | Cnih1 |
| ENSRNOG00000010966 | 1913,04 | 0,39 | 0,000143 | Itgb1 |
| ENSRNOG00000049585 | 27,11 | 1,65 | 0,000148 |  |
| ENSRNOG00000018129 | 677,76 | -0,59 | 0,000149 | Ndufab1 |
| ENSRNOG00000006614 | 115,71 | 0,78 | 0,000152 |  |
| ENSRNOG00000007637 | 201,08 | 1,21 | 0,000152 | Acer2 |
| ENSRNOG00000019648 | 212,49 | 0,84 | 0,000152 |  |
| ENSRNOG00000042499 | 166,87 | 0,81 | 0,000152 | Tmsb10 |
| ENSRNOG00000007583 | 1073,31 | -0,57 | 0,000152 | Pygb |
| ENSRNOG00000024849 | 160,56 | 0,84 | 0,000161 | Tor1aip2 |
| ENSRNOG00000022980 | 914,45 | -0,52 | 0,00017 | Sdhd |
| ENSRNOG00000016021 | 432,06 | -0,67 | 0,000171 | Lims2 |
| ENSRNOG00000018816 | 991,22 | -0,62 | 0,000175 | Cox5a |
| ENSRNOG00000016794 | 61,30 | -1,16 | 0,000176 | Phyhd1 |
| ENSRNOG00000001979 | 2195,26 | -0,67 | 0,000176 | Rcan1 |
| ENSRNOG00000002358 | 150,20 | 0,68 | 0,000183 | Scpep1 |
| ENSRNOG00000002848 | 1139,76 | 0,93 | 0,000185 | Maoa |
| ENSRNOG00000000060 | 9,27 | 1,63 | 0,000189 | Cplx1 |
| ENSRNOG00000019206 | 16,24 | 1,46 | 0,000199 | Nupr1 |
| ENSRNOG00000004537 | 165,78 | -0,77 | 0,000204 | Rxrg |
| ENSRNOG00000028137 | 57,05 | 1,31 | 0,000204 | Mki67 |
| ENSRNOG00000047746 | 12,80 | 1,61 | 0,000204 |  |
| ENSRNOG00000007905 | 749,26 | -0,51 | 0,000206 | Itga7 |
| ENSRNOG00000049912 | 701,91 | -0,66 | 0,000212 | Atp5e |
| ENSRNOG00000012477 | 1861,61 | -0,67 | 0,000213 | Eef1a2 |
| ENSRNOG00000019018 | 593,95 | 0,69 | 0,000213 | Plat |
| ENSRNOG00000000498 | 348,36 | -0,86 | 0,000214 | Anks1a |
| ENSRNOG00000016050 | 193,56 | 0,66 | 0,000214 | Fgfr1 |
| ENSRNOG00000030118 | 798,39 | 0,62 | 0,000217 | Msn |
| ENSRNOG00000015233 | 1068,78 | -0,55 | 0,000219 | Etfa |
| ENSRNOG00000011977 | 187,22 | 0,76 | 0,000236 | Sema5a |
| ENSRNOG00000016356 | 1845,39 | -0,50 | 0,000236 | Got1 |
| ENSRNOG00000003261 | 791,03 | 0,60 | 0,00026 | Usp9x |
| ENSRNOG00000020719 | 3088,68 | -0,49 | 0,00026 | Hrc |
| ENSRNOG00000022790 | 2499,78 | -0,48 | 0,000264 |  |
| ENSRNOG00000014137 | 220,43 | 0,88 | 0,000269 | Fbln1 |
| ENSRNOG00000037514 | 99,72 | 0,96 | 0,000309 | Qser1 |
| ENSRNOG00000012307 | 8282,39 | -0,37 | 0,000333 | Mybpc3 |
| ENSRNOG00000046848 | 66,15 | 1,02 | 0,000336 | PCOLCE2 |
| ENSRNOG00000038436 | 149,68 | 0,82 | 0,000365 | RGD1307100 |
| ENSRNOG00000009196 | 95,12 | 1,07 | 0,000372 | Rc3h2 |
| ENSRNOG00000003917 | 12,69 | -1,54 | 0,000375 | Uck2 |
| ENSRNOG00000012881 | 160,54 | 1,04 | 0,000382 | Fgl2 |
| ENSRNOG00000016680 | 165,33 | 0,82 | 0,000391 | Nsd1 |
| ENSRNOG00000019462 | 71,38 | 1,01 | 0,000392 |  |
| ENSRNOG00000017311 | 216,57 | -0,79 | 0,000392 | Me3 |
| ENSRNOG00000030210 | 48,94 | 1,19 | 0,000409 | Fndc1 |
| ENSRNOG00000016208 | 28,62 | 1,39 | 0,000438 | Setbp1 |
| ENSRNOG00000005600 | 29,18 | 1,20 | 0,00044 | Nr4a2 |
| ENSRNOG00000001120 | 137,85 | 0,74 | 0,000442 | Med13l |
| ENSRNOG00000002746 | 84,59 | 0,99 | 0,000476 | Fstl1 |
| ENSRNOG00000015093 | 1241,94 | 0,68 | 0,000481 | Sparcl1 |
| ENSRNOG00000012481 | 22,88 | -1,39 | 0,000482 | Ppm1j |
| ENSRNOG00000010958 | 634,51 | -0,50 | 0,000482 | Prdx3 |
| ENSRNOG00000015085 | 797,67 | -0,50 | 0,000487 | Dmpk |
| ENSRNOG00000001823 | 100,59 | 0,83 | 0,0005 | St6gal1 |
| ENSRNOG00000000860 | 254,36 | -0,61 | 0,000506 | Vwa7 |
| ENSRNOG00000001177 | 953,28 | -0,60 | 0,000507 | Acads |
| ENSRNOG00000013391 | 2096,54 | 0,47 | 0,000509 | Sorbs2 |
| ENSRNOG00000026942 | 107,24 | 0,87 | 0,000513 | RGD1311595 |
| ENSRNOG00000012616 | 172,93 | 0,63 | 0,000526 | Ppt1 |
| ENSRNOG00000016610 | 184,01 | -0,78 | 0,000526 | Arhgap1 |
| ENSRNOG00000010233 | 309,18 | 0,78 | 0,000541 | Cald1 |
| ENSRNOG00000008877 | 111,89 | -0,85 | 0,000543 |  |
| ENSRNOG00000020723 | 124,23 | 0,77 | 0,000545 | Pten |
| ENSRNOG00000005841 | 157,89 | 0,64 | 0,000565 | Erp44 |
| ENSRNOG00000015953 | 348,13 | -0,56 | 0,000596 | Oaz2 |
| ENSRNOG00000037227 | 50,62 | 1,03 | 0,000596 | Yes1 |
| ENSRNOG00000015852 | 144,58 | 0,84 | 0,000597 | Arhgap35 |
| ENSRNOG00000045829 | 215,91 | 1,31 | 0,000615 | Thbs1 |
| ENSRNOG00000000648 | 247,83 | 0,57 | 0,000615 | Jmjd1c |
| ENSRNOG00000009102 | 640,88 | 0,41 | 0,000627 | Fermt2 |
| ENSRNOG00000010331 | 1339,05 | 0,44 | 0,000637 | Ctsb |
| ENSRNOG00000012184 | 207,81 | -0,59 | 0,000649 | Urgcp |
| ENSRNOG00000012804 | 183,21 | 0,84 | 0,000677 | C1qc |
| ENSRNOG00000026842 | 3154,92 | -0,47 | 0,000679 | Nnt |
| ENSRNOG00000023465 | 199,03 | -1,00 | 0,000681 | LOC500300 |
| ENSRNOG00000013744 | 79,95 | 1,22 | 0,000686 | Akip1 |
| ENSRNOG00000018454 | 530,20 | 0,91 | 0,000686 | Apoe |
| ENSRNOG00000002680 | 1569,14 | 0,42 | 0,000701 | Lamc1 |
| ENSRNOG00000002229 | 462,53 | -0,60 | 0,000709 | Adcy5 |
| ENSRNOG00000001249 | 515,04 | 0,67 | 0,000713 | Col6a1 |
| ENSRNOG00000020353 | 80,47 | 0,90 | 0,000756 | Sh3pxd2a |
| ENSRNOG00000007316 | 1035,16 | -0,46 | 0,000789 | Idh3B |
| ENSRNOG00000001982 | 72,26 | 0,97 | 0,000801 | Cblb |
| ENSRNOG00000025539 | 103,15 | 1,04 | 0,000801 | Vps13a |
| ENSRNOG00000002730 | 248,94 | 0,81 | 0,000828 | Rgs5 |
| ENSRNOG00000033593 | 148,30 | 0,78 | 0,000848 | Osbpl9 |
| ENSRNOG00000008755 | 1180,93 | -0,48 | 0,000865 | Acox1 |
| ENSRNOG00000009037 | 74,08 | 1,13 | 0,000869 | Sulf1 |
| ENSRNOG00000004311 | 14,76 | -1,49 | 0,000869 | Gpr182 |
| ENSRNOG00000009351 | 169,41 | -0,59 | 0,000872 | Srp68 |
| ENSRNOG00000015496 | 185,64 | 0,71 | 0,000874 | Tpm4 |
| ENSRNOG00000043357 | 83,04 | 1,05 | 0,000876 | Zfp407 |
| ENSRNOG00000006997 | 1213,57 | 0,42 | 0,000877 | App |
| ENSRNOG00000003815 | 773,64 | -0,47 | 0,000887 | Slc25a11 |
| ENSRNOG00000015505 | 62,38 | 1,25 | 0,000887 | Mfap5 |
| ENSRNOG00000022373 | 104,49 | -0,90 | 0,000887 | Dennd4b |
| ENSRNOG00000008180 | 102,53 | 0,85 | 0,000902 | Lyn |
| ENSRNOG00000010240 | 17,43 | 1,47 | 0,000909 | Fam46a |
| ENSRNOG00000015385 | 443,06 | -0,73 | 0,000909 | Pink1 |
| ENSRNOG00000011667 | 207,51 | -0,77 | 0,000909 | Fastk |
| ENSRNOG00000009536 | 186,97 | -0,62 | 0,000928 | Pgp |
| ENSRNOG00000022686 | 157,18 | 0,66 | 0,000945 | Zdhhc2 |
| ENSRNOG00000024089 | 105,11 | 0,96 | 0,000962 | Fndc3b |
| ENSRNOG00000032882 | 45,70 | 1,22 | 0,000969 |  |
| ENSRNOG00000007457 | 387,29 | 0,82 | 0,000969 | Serping1 |
| ENSRNOG00000002994 | 704,68 | -0,57 | 0,000979 |  |
| ENSRNOG00000005130 | 3873,29 | -0,53 | 0,000979 | Ogdh |
| ENSRNOG00000018029 | 136,58 | -0,69 | 0,000991 | Doc2g |
| ENSRNOG00000008676 | 360,58 | 0,79 | 0,001001 | Emp1 |
| ENSRNOG00000009076 | 29,52 | 1,43 | 0,001001 | Ttpal |
| ENSRNOG00000025001 | 81,99 | 0,88 | 0,001002 | Pcolce |
| ENSRNOG00000001925 | 29,73 | 1,24 | 0,00102 |  |
| ENSRNOG00000042274 | 112,28 | -0,94 | 0,001031 | Fbxo31 |
| ENSRNOG00000016603 | 128,90 | -0,81 | 0,001099 | Rtn2 |
| ENSRNOG00000015904 | 183,44 | -0,91 | 0,001099 | Wfdc1 |
| ENSRNOG00000017020 | 33,72 | 1,16 | 0,001117 | Inpp5d |
| ENSRNOG00000016010 | 129,54 | -0,85 | 0,001117 | Mul1 |
| ENSRNOG00000014482 | 89,93 | 0,89 | 0,00113 | Slf2 |
| ENSRNOG00000005851 | 161,18 | 0,72 | 0,001242 |  |
| ENSRNOG00000019276 | 344,16 | -0,62 | 0,001293 | RGD735029 |
| ENSRNOG00000028017 | 163,22 | -0,67 | 0,001296 | Tmem109 |
| ENSRNOG00000024689 | 86,26 | 0,94 | 0,001325 | Hopx |
| ENSRNOG00000019981 | 252,37 | 0,50 | 0,001333 | Sdf4 |
| ENSRNOG00000010633 | 2628,89 | -0,56 | 0,001345 | Acsl1 |
| ENSRNOG00000002180 | 53,55 | 1,03 | 0,001368 | Tbc1d1 |
| ENSRNOG00000018382 | 67,21 | 0,83 | 0,001371 | Inpp4b |
| ENSRNOG00000031312 | 138,84 | 0,68 | 0,001371 | Tnfrsf1a |
| ENSRNOG00000000599 | 398,02 | 0,57 | 0,001383 | Lama4 |
| ENSRNOG00000019205 | 255,57 | -0,65 | 0,001392 | Gnpat |
| ENSRNOG00000009012 | 636,19 | -0,55 | 0,001451 |  |
| ENSRNOG00000002632 | 1403,17 | -0,43 | 0,001456 | Naca |
| ENSRNOG00000002669 | 452,00 | -0,61 | 0,001462 | Cluh |
| ENSRNOG00000015346 | 156,13 | -0,66 | 0,001462 | Obsl1 |
| ENSRNOG00000002089 | 66,10 | 0,92 | 0,001566 | Ccng2 |
| ENSRNOG00000009466 | 300,50 | -0,56 | 0,001566 | Unc45b |
| ENSRNOG00000013963 | 895,42 | 0,58 | 0,001596 | Il6st |
| ENSRNOG00000000146 | 317,53 | 0,64 | 0,001596 |  |
| ENSRNOG00000028356 | 1504,60 | -0,52 | 0,001617 | Chchd10 |
| ENSRNOG00000012080 | 127,83 | 0,80 | 0,001617 |  |
| ENSRNOG00000008079 | 478,24 | -0,51 | 0,001621 | Ugp2 |
| ENSRNOG00000006338 | 133,37 | 0,77 | 0,001621 | Lrp6 |
| ENSRNOG00000012014 | 147,24 | -0,67 | 0,001664 | Fam160b2 |
| ENSRNOG00000027191 | 224,08 | 0,77 | 0,001714 | Birc6 |
| ENSRNOG00000019749 | 160,42 | -0,63 | 0,001822 | Ube2j2 |
| ENSRNOG00000002525 | 7,42 | 1,41 | 0,001862 | Ptgs2 |
| ENSRNOG00000008170 | 1257,78 | -0,55 | 0,00187 | Jph2 |
| ENSRNOG00000026930 | 1145,57 | -0,49 | 0,00187 |  |
| ENSRNOG00000019333 | 488,68 | 0,57 | 0,001895 | Hipk1 |
| ENSRNOG00000002075 | 93,49 | 0,79 | 0,001929 | Cnot6l |
| ENSRNOG00000007804 | 24,34 | 1,36 | 0,001947 | C1galt1 |
| ENSRNOG00000043451 | 108,72 | 1,38 | 0,001962 | Spp1 |
| ENSRNOG00000005332 | 160,34 | -0,83 | 0,001962 | Csdc2 |
| ENSRNOG00000005679 | 27,05 | 1,18 | 0,001962 | Fap |
| ENSRNOG00000046799 | 214,48 | -0,64 | 0,001962 | Phb |
| ENSRNOG00000021200 | 374,06 | -0,64 | 0,001975 | Hfe2 |
| ENSRNOG00000029911 | 28,29 | 1,40 | 0,001975 | Cilp |
| ENSRNOG00000011912 | 308,90 | -0,69 | 0,001979 | Tmem38a |
| ENSRNOG00000005437 | 15,46 | 1,32 | 0,001992 | Rida |
| ENSRNOG00000004481 | 78,62 | 0,94 | 0,002016 | Adss |
| ENSRNOG00000004206 | 130,93 | -0,75 | 0,00203 | Glrx5 |
| ENSRNOG00000011752 | 96,12 | 1,02 | 0,002035 |  |
| ENSRNOG00000002722 | 217,48 | 0,64 | 0,002035 | Sec14l1 |
| ENSRNOG00000011762 | 59,48 | 0,76 | 0,00205 | Elf1 |
| ENSRNOG00000014610 | 45,22 | 1,15 | 0,002067 | Anpep |
| ENSRNOG00000050697 | 46,65 | 1,09 | 0,002067 | Ctsz |
| ENSRNOG00000014573 | 68,91 | -1,12 | 0,002105 | Ckmt1b |
| ENSRNOG00000010274 | 108,29 | 0,80 | 0,002125 | Smc4 |
| ENSRNOG00000010635 | 89,74 | 0,85 | 0,002194 | Igfbp4 |
| ENSRNOG00000033615 | 33091,01 | 0,94 | 0,002238 | ND3 |
| ENSRNOG00000016369 | 524,24 | -0,51 | 0,002263 |  |
| ENSRNOG00000010475 | 26,60 | 1,14 | 0,002263 | Casp3 |
| ENSRNOG00000002713 | 70,85 | -1,01 | 0,002339 | Zfp672 |
| ENSRNOG00000006375 | 1204,65 | -0,48 | 0,002339 | Vdac1 |
| ENSRNOG00000013443 | 330,59 | 0,48 | 0,002359 | Tm9sf3 |
| ENSRNOG00000015696 | 51,93 | -0,93 | 0,002387 | Cdk5rap1 |
| ENSRNOG00000013538 | 156,18 | 0,64 | 0,002392 | Capza1 |
| ENSRNOG00000012738 | 194,09 | -0,63 | 0,002395 | Eif3m |
| ENSRNOG00000018239 | 294,48 | -0,51 | 0,002409 | Dhrs4 |
| ENSRNOG00000015644 | 166,10 | 0,86 | 0,002426 | Ugcg |
| ENSRNOG00000001285 | 21860,85 | -0,47 | 0,002435 | Atp2a2 |
| ENSRNOG00000018371 | 54,71 | 1,05 | 0,002478 | Tubb6 |
| ENSRNOG00000032404 | 22,95 | 1,34 | 0,002478 |  |
| ENSRNOG00000003679 | 191,63 | 0,62 | 0,002478 | Med13 |
| ENSRNOG00000003723 | 521,06 | 0,46 | 0,002482 | Wdr26 |
| ENSRNOG00000012816 | 40,39 | -1,08 | 0,002492 |  |
| ENSRNOG00000004840 | 799,54 | 0,70 | 0,0026 |  |
| ENSRNOG00000029682 | 153,30 | 0,77 | 0,0026 | Clic1 |
| ENSRNOG00000011971 | 305,46 | 0,64 | 0,002623 | C1s |
| ENSRNOG00000025443 | 874,04 | -0,56 | 0,002786 | Map1lc3a |
| ENSRNOG00000024799 | 197,75 | 0,73 | 0,002791 |  |
| ENSRNOG00000017637 | 28,85 | -1,08 | 0,002799 | Fbp2 |
| ENSRNOG00000013135 | 210,83 | 0,68 | 0,002799 | Ptpn12 |
| ENSRNOG00000002459 | 236,03 | -0,65 | 0,002938 | Fbxo40 |
| ENSRNOG00000011782 | 1981,79 | -0,51 | 0,002964 | Got2 |
| ENSRNOG00000011054 | 79,82 | 0,84 | 0,002964 | Laptm5 |
| ENSRNOG00000020344 | 20,79 | 1,29 | 0,002964 | Sntb2 |
| ENSRNOG00000006694 | 713,35 | 0,48 | 0,002976 |  |
| ENSRNOG00000018181 | 158,25 | -0,70 | 0,002977 | Stk25 |
| ENSRNOG00000042519 | 186,89 | 0,73 | 0,003069 | Peak1 |
| ENSRNOG00000003905 | 64,86 | 0,83 | 0,003108 | Nsf |
| ENSRNOG00000007706 | 231,07 | 0,69 | 0,003119 | Prkaa2 |
| ENSRNOG00000013572 | 21,32 | 1,35 | 0,003119 | Lxn |
| ENSRNOG00000017418 | 467,83 | -0,54 | 0,003119 | Rps3 |
| ENSRNOG00000046547 | 441,13 | -0,66 | 0,003124 | Rbm24 |
| ENSRNOG00000014090 | 698,00 | -0,58 | 0,003128 | Retsat |
| ENSRNOG00000003809 | 113,88 | 0,81 | 0,003185 | Sat1 |
| ENSRNOG00000026364 | 46,99 | 1,01 | 0,003185 |  |
| ENSRNOG00000016190 | 1015,67 | -0,43 | 0,003205 | Coq9 |
| ENSRNOG00000016896 | 393,90 | 0,62 | 0,003205 | Rpl3 |
| ENSRNOG00000006976 | 26,97 | -1,21 | 0,003298 | Med6 |
| ENSRNOG00000010881 | 1033,85 | 0,39 | 0,003298 | Trak2 |
| ENSRNOG00000007356 | 284,63 | 0,53 | 0,003306 | Chmp3 |
| ENSRNOG00000016758 | 35,77 | 1,16 | 0,003307 | Loxl2 |
| ENSRNOG00000002886 | 228,35 | 0,62 | 0,003307 | Myh10 |
| ENSRNOG00000002407 | 31,62 | 1,11 | 0,003583 | Pdxdc1 |
| ENSRNOG00000000413 | 4120,97 | -0,44 | 0,003632 | Pln |
| ENSRNOG00000002833 | 89,94 | 0,95 | 0,003726 | Gsk3b |
| ENSRNOG00000033169 | 205,39 | 0,71 | 0,003743 | Cpeb4 |
| ENSRNOG00000012999 | 286,75 | -0,55 | 0,003743 | Phb2 |
| ENSRNOG00000006683 | 1750,88 | 0,37 | 0,003743 |  |
| ENSRNOG00000016133 | 168,10 | 0,58 | 0,003743 | Sumo1 |
| ENSRNOG00000019728 | 11,25 | 1,33 | 0,003744 | Itgam |
| ENSRNOG00000015329 | 153,79 | -0,72 | 0,00378 | Kpna2 |
| ENSRNOG00000007628 | 422,19 | -0,51 | 0,003802 | Ptp4a3 |
| ENSRNOG00000002981 | 68,92 | 0,98 | 0,003802 |  |
| ENSRNOG00000014779 | 165,46 | 0,72 | 0,003802 | Pdcd4 |
| ENSRNOG00000015049 | 912,55 | -0,43 | 0,003873 | Scn5a |
| ENSRNOG00000019939 | 100,90 | 0,99 | 0,004001 |  |
| ENSRNOG00000003148 | 300,33 | 0,54 | 0,004008 | Timp2 |
| ENSRNOG00000017188 | 81,82 | -0,71 | 0,004008 | Cyp27a1 |
| ENSRNOG00000003434 | 46,78 | 0,92 | 0,004008 | Trove2 |
| ENSRNOG00000021267 | 335,15 | -0,54 | 0,004012 |  |
| ENSRNOG00000000893 | 121,94 | 0,66 | 0,004016 | Tmem248 |
| ENSRNOG00000016281 | 1909,96 | 0,46 | 0,004039 | Col4a1 |
| ENSRNOG00000017469 | 166,20 | 0,82 | 0,004051 | Anxa1 |
| ENSRNOG00000010984 | 542,82 | -0,47 | 0,004067 | Anxa11 |
| ENSRNOG00000011987 | 103,37 | 0,75 | 0,004072 | Cd2ap |
| ENSRNOG00000004284 | 174,88 | 0,72 | 0,004094 | Btg1 |
| ENSRNOG00000022725 | 23,32 | -1,19 | 0,004272 | Iba57 |
| ENSRNOG00000006331 | 50,67 | 0,99 | 0,004272 | Elovl5 |
| ENSRNOG00000011501 | 191,44 | 0,70 | 0,004272 | Atp1b3 |
| ENSRNOG00000025350 | 171,42 | -0,58 | 0,004298 | Ppp1r13l |
| ENSRNOG00000017133 | 312,96 | -0,51 | 0,00446 | LOC306766 |
| ENSRNOG00000032701 | 308,10 | -0,53 | 0,004478 |  |
| ENSRNOG00000009252 | 489,93 | -0,45 | 0,004536 |  |
| ENSRNOG00000026679 | 411,38 | -0,70 | 0,004559 | Scn4b |
| ENSRNOG00000019048 | 853,62 | -0,41 | 0,004572 | Sod2 |
| ENSRNOG00000019485 | 317,75 | -0,55 | 0,004572 | Bckdk |
| ENSRNOG00000020464 | 102,81 | -0,82 | 0,004576 | Mrpl54 |
| ENSRNOG00000016023 | 270,10 | -0,49 | 0,004576 | Kank1 |
| ENSRNOG00000011559 | 188,96 | 0,58 | 0,004583 | Cnn3 |
| ENSRNOG00000004464 | 129,22 | 0,78 | 0,004595 | Sel1l |
| ENSRNOG00000019518 | 6,28 | -1,28 | 0,004612 | Pde4c |
| ENSRNOG00000026700 | 3273,78 | -0,37 | 0,004629 |  |
| ENSRNOG00000014030 | 401,54 | 0,55 | 0,004704 | Synm |
| ENSRNOG00000014453 | 847,16 | 0,39 | 0,004705 | Anxa5 |
| ENSRNOG00000049911 | 8,63 | -1,31 | 0,004708 | LOC102556347 |
| ENSRNOG00000029042 | 3969,46 | 0,73 | 0,004708 | ND6 |
| ENSRNOG00000005678 | 574,06 | 0,51 | 0,004762 | Lamb1 |
| ENSRNOG00000005330 | 237,78 | 0,70 | 0,004803 | Crebbp |
| ENSRNOG00000008709 | 47,14 | 0,93 | 0,004921 | Arhgap32 |
| ENSRNOG00000015869 | 236,99 | -0,68 | 0,004924 | Pccb |
| ENSRNOG00000050519 | 1014,02 | 0,30 | 0,004966 | Rhoa |
| ENSRNOG00000019465 | 362,19 | 0,62 | 0,005097 | Gnai3 |
| ENSRNOG00000018282 | 117,58 | 0,80 | 0,005097 | Gda |
| ENSRNOG00000003120 | 100,60 | 1,04 | 0,005319 | Prelp |
| ENSRNOG00000005587 | 1247,88 | -0,39 | 0,005319 | Suclg1 |
| ENSRNOG00000004078 | 737,79 | -0,55 | 0,005333 | Eno3 |
| ENSRNOG00000034015 | 408,50 | 0,48 | 0,005428 | Capn2 |
| ENSRNOG00000006828 | 72,77 | 0,80 | 0,005469 | Baz1a |
| ENSRNOG00000019418 | 160,63 | -0,67 | 0,005497 | Lrrc4b |
| ENSRNOG00000006898 | 86,36 | -0,85 | 0,005514 | Mrps16 |
| ENSRNOG00000048169 | 393,82 | -0,65 | 0,005514 | Tuba8 |
| ENSRNOG00000020386 | 330,14 | 0,62 | 0,005514 | Ash1l |
| ENSRNOG00000010673 | 72,14 | -0,73 | 0,005536 | Eral1 |
| ENSRNOG00000021808 | 1446,16 | -0,49 | 0,005578 | Tecr |
| ENSRNOG00000016334 | 119,32 | 0,77 | 0,005661 | Ptbp3 |
| ENSRNOG00000007350 | 45,40 | 1,07 | 0,005698 | Rac2 |
| ENSRNOG00000016573 | 72,79 | -0,78 | 0,005718 | Dgat2 |
| ENSRNOG00000021084 | 27,13 | 1,21 | 0,005739 |  |
| ENSRNOG00000007459 | 468,25 | 0,53 | 0,005752 | Pcnx1 |
| ENSRNOG00000036742 | 2577,24 | -0,35 | 0,005772 | Uqcrc2 |
| ENSRNOG00000020955 | 14715,52 | -0,48 | 0,005863 | Myl3 |
| ENSRNOG00000001436 | 369,52 | -0,44 | 0,005873 | Ywhag |
| ENSRNOG00000013346 | 62,78 | -0,79 | 0,005967 | Asb14 |
| ENSRNOG00000023651 | 6,62 | -1,28 | 0,005967 |  |
| ENSRNOG00000010154 | 30,90 | -1,01 | 0,005999 | Zc3hc1 |
| ENSRNOG00000017022 | 179,60 | 0,66 | 0,006016 | Cerk |
| ENSRNOG00000000585 | 295,69 | -0,61 | 0,006113 | Amd1 |
| ENSRNOG00000007518 | 564,67 | 0,40 | 0,006131 | Nckap1 |
| ENSRNOG00000020242 | 9,18 | -1,27 | 0,006136 | Art5 |
| ENSRNOG00000010488 | 537,55 | 0,54 | 0,006136 | Zmiz1 |
| ENSRNOG00000027520 | 241,42 | 0,55 | 0,006226 | Pls3 |
| ENSRNOG00000029339 | 20,00 | -1,20 | 0,006304 | LOC100363268 |
| ENSRNOG00000001793 | 441,46 | 0,59 | 0,006306 | Heg1 |
| ENSRNOG00000004726 | 996,10 | -0,37 | 0,006306 | Mapkapk2 |
| ENSRNOG00000004290 | 232,74 | 0,58 | 0,006306 | Grb10 |
| ENSRNOG00000007419 | 45,33 | 1,03 | 0,006317 | Pank3 |
| ENSRNOG00000023647 | 5947,49 | -0,39 | 0,006385 |  |
| ENSRNOG00000043189 | 48,07 | -0,88 | 0,00639 | Trub2 |
| ENSRNOG00000021440 | 564,17 | -0,48 | 0,00639 | Pptc7 |
| ENSRNOG00000003730 | 144,86 | 0,68 | 0,006418 |  |
| ENSRNOG00000001276 | 347,34 | -0,50 | 0,006547 | Pcnt |
| ENSRNOG00000018117 | 2191,46 | -0,43 | 0,006547 | Ndufv1 |
| ENSRNOG00000021090 | 3037,88 | -0,47 | 0,006552 | Pygm |
| ENSRNOG00000007793 | 179,40 | 0,72 | 0,006566 | Pnrc1 |
| ENSRNOG00000007476 | 39,14 | 1,14 | 0,006566 |  |
| ENSRNOG00000034025 | 43,54 | 1,06 | 0,006566 | Ptprj |
| ENSRNOG00000003163 | 1126,30 | -0,46 | 0,006566 | Sdhc |
| ENSRNOG00000018343 | 853,04 | -0,44 | 0,006589 | Isca1 |
| ENSRNOG00000028711 | 94,96 | -0,76 | 0,006675 | Dgat1 |
| ENSRNOG00000012329 | 196,87 | -0,69 | 0,006778 | Saraf |
| ENSRNOG00000010389 | 3259,46 | -0,32 | 0,006835 | Ndrg2 |
| ENSRNOG00000008414 | 1124,72 | -0,41 | 0,006891 | Bsg |
| ENSRNOG00000009340 | 30,33 | 1,09 | 0,006891 | Zbtb6 |
| ENSRNOG00000017619 | 151,12 | 0,76 | 0,00698 | Aldh1a1 |
| ENSRNOG00000002840 | 13916,29 | -0,29 | 0,00698 | Atp5b |
| ENSRNOG00000004508 | 191,50 | 0,79 | 0,006994 |  |
| ENSRNOG00000019598 | 1212,17 | -0,54 | 0,00707 | Vegfa |
| ENSRNOG00000011358 | 510,21 | 0,49 | 0,007202 | Hipk3 |
| ENSRNOG00000026425 | 196,85 | -0,50 | 0,00722 | Ppp6r2 |
| ENSRNOG00000019438 | 43,28 | -0,93 | 0,007246 | Rnf31 |
| ENSRNOG00000032844 | 107,93 | 0,83 | 0,007283 | RT1-Da |
| ENSRNOG00000014797 | 43,89 | 0,85 | 0,007285 | Tmbim1 |
| ENSRNOG00000025476 | 77,88 | 0,85 | 0,007396 | Tmem252 |
| ENSRNOG00000012303 | 381,35 | -0,63 | 0,007396 | Apobec2 |
| ENSRNOG00000006965 | 355,76 | 0,47 | 0,007396 | Aff4 |
| ENSRNOG00000002115 | 621,65 | -0,50 | 0,007423 | Sod1 |
| ENSRNOG00000025680 | 297,97 | 0,43 | 0,007443 |  |
| ENSRNOG00000004496 | 1061,70 | 0,49 | 0,007447 | Rock2 |
| ENSRNOG00000011123 | 4,68 | -1,22 | 0,007461 |  |
| ENSRNOG00000009039 | 69,68 | -0,79 | 0,007461 | Trappc12 |
| ENSRNOG00000010473 | 218,49 | -0,49 | 0,007461 | Cand2 |
| ENSRNOG00000011696 | 126,14 | 0,62 | 0,007559 | Lifr |
| ENSRNOG00000011227 | 111,47 | 0,76 | 0,007575 | Atp1b2 |
| ENSRNOG00000008245 | 65,93 | 0,87 | 0,007575 | Ptgis |
| ENSRNOG00000029798 | 12,83 | 1,18 | 0,007626 | Lrrc4c |
| ENSRNOG00000000845 | 132,16 | -0,69 | 0,007626 |  |
| ENSRNOG00000004245 | 232,64 | -0,53 | 0,007806 | Mgst3 |
| ENSRNOG00000009421 | 525,93 | -0,52 | 0,007817 | Ivd |
| ENSRNOG00000024907 | 327,53 | 0,48 | 0,007819 | Tnrc6b |
| ENSRNOG00000019036 | 139,65 | -0,83 | 0,007918 | Ldhd |
| ENSRNOG00000019263 | 94,76 | 0,67 | 0,007918 |  |
| ENSRNOG00000010977 | 64,71 | 0,83 | 0,007973 | Igfbp6 |
| ENSRNOG00000020878 | 65,14 | -0,89 | 0,0081 | Keap1 |
| ENSRNOG00000003841 | 4,76 | 1,18 | 0,008107 | Kcnh1 |
| ENSRNOG00000046763 | 425,84 | -0,47 | 0,008193 | Adssl1 |
| ENSRNOG00000009184 | 62,91 | 0,83 | 0,008367 | Foxp1 |
| ENSRNOG00000006939 | 326,66 | -0,55 | 0,008458 | Ndufa7 |
| ENSRNOG00000001653 | 121,73 | -0,72 | 0,008623 | St3gal6 |
| ENSRNOG00000015021 | 24,29 | -1,06 | 0,00879 | Naxd |
| ENSRNOG00000017406 | 55,20 | 0,86 | 0,008833 |  |
| ENSRNOG00000013878 | 31,30 | 1,16 | 0,008833 | Hmgxb4 |
| ENSRNOG00000019211 | 28,03 | 1,13 | 0,008891 | Olfml3 |
| ENSRNOG00000042576 | 18,24 | 1,17 | 0,008891 | Tcp11l1 |
| ENSRNOG00000020138 | 231,87 | -0,55 | 0,008948 | Slc4a3 |
| ENSRNOG00000048730 | 22,70 | 1,18 | 0,008976 |  |
| ENSRNOG00000021199 | 14,94 | 1,22 | 0,008976 | Fcgr1a |
| ENSRNOG00000016460 | 4224,48 | 0,32 | 0,00902 | Clu |
| ENSRNOG00000017983 | 168,75 | -0,60 | 0,009055 | Ubac1 |
| ENSRNOG00000001966 | 248,17 | 0,69 | 0,009084 |  |
| ENSRNOG00000002877 | 661,00 | 0,43 | 0,009172 |  |
| ENSRNOG00000017751 | 89,65 | 0,68 | 0,009176 | Arl6ip1 |
| ENSRNOG00000020298 | 1261,21 | -0,46 | 0,009194 | Bag3 |
| ENSRNOG00000045892 | 787,99 | 0,46 | 0,009368 | Cfl2 |
| ENSRNOG00000004709 | 135,04 | 0,62 | 0,009368 | Foxn3 |
| ENSRNOG00000012193 | 149,40 | 0,74 | 0,009368 |  |
| ENSRNOG00000002750 | 70,07 | 1,06 | 0,009401 |  |
| ENSRNOG00000010529 | 101,94 | 0,89 | 0,009419 | Thbs2 |
| ENSRNOG00000017446 | 432,26 | -0,49 | 0,009419 | Ndufs8 |
| ENSRNOG00000001870 | 69,92 | -0,82 | 0,009419 | Lztr1 |
| ENSRNOG00000002385 | 57,65 | 1,00 | 0,009454 | Prg4 |
| ENSRNOG00000018268 | 4,90 | 1,08 | 0,009506 | Hhip |
| ENSRNOG00000006305 | 1078,59 | 0,43 | 0,009571 | Slc38a2 |
| ENSRNOG00000005196 | 100,19 | 0,67 | 0,009658 | Slc12a6 |
| ENSRNOG00000011913 | 74,39 | 1,00 | 0,009797 | Cp |
| ENSRNOG00000018997 | 221,27 | -0,89 | 0,009804 | Myh7b |
| ENSRNOG00000002418 | 121,08 | 0,83 | 0,009837 | Tgfb2 |
| ENSRNOG00000021125 | 314,20 | -0,49 | 0,009837 | Prdx5 |
| ENSRNOG00000010711 | 115,95 | 0,73 | 0,009874 | Dicer1 |
| ENSRNOG00000000633 | 699,32 | -0,58 | 0,009897 | Rhobtb1 |
| ENSRNOG00000046502 | 333,93 | -0,41 | 0,009897 | Lonp1 |
| ENSRNOG00000037242 | 46,48 | 0,95 | 0,00995 |  |
| ENSRNOG00000014496 | 449,75 | -0,51 | 0,009976 | Coro6 |
| ENSRNOG00000015692 | 211,77 | 0,59 | 0,010011 | Taok1 |
| ENSRNOG00000024194 | 100,07 | 0,87 | 0,010011 | Rsf1 |
| ENSRNOG00000007769 | 246,56 | -0,61 | 0,010011 | March2 |
| ENSRNOG00000023433 | 110,34 | -0,58 | 0,010338 | Gata6 |
| ENSRNOG00000021062 | 85,56 | 0,85 | 0,010338 | Fxyd5 |
| ENSRNOG00000012658 | 162,20 | -0,71 | 0,010411 | Pdlim3 |
| ENSRNOG00000006839 | 133,22 | 0,69 | 0,010522 | Arl5a |
| ENSRNOG00000019459 | 545,89 | -0,57 | 0,010579 | Oaz1 |
| ENSRNOG00000003049 | 420,36 | -0,48 | 0,010687 | Hsd17b10 |
| ENSRNOG00000033372 | 184,70 | 0,57 | 0,010734 | Klhl24 |
| ENSRNOG00000020769 | 68,17 | 0,85 | 0,010817 | Crebrf |
| ENSRNOG00000050727 | 97,97 | 0,80 | 0,010817 |  |
| ENSRNOG00000018293 | 71,34 | -0,67 | 0,010817 | Strip1 |
| ENSRNOG00000001501 | 12,89 | -1,19 | 0,01094 | Snrpa |
| ENSRNOG00000018250 | 11925,41 | -0,41 | 0,01094 | Tnni3 |
| ENSRNOG00000025295 | 333,97 | -0,50 | 0,01094 | Mavs |
| ENSRNOG00000009878 | 69,56 | 0,74 | 0,011013 | Crtap |
| ENSRNOG00000043381 | 214,81 | -0,51 | 0,011159 | Cyth1 |
| ENSRNOG00000027151 | 34,14 | 0,96 | 0,011173 | Lrrc58 |
| ENSRNOG00000019319 | 62,84 | 0,74 | 0,011173 | Fchsd2 |
| ENSRNOG00000007372 | 26,83 | 1,05 | 0,011176 | Casp1 |
| ENSRNOG00000008082 | 47,93 | -0,93 | 0,01121 | Rgs6 |
| ENSRNOG00000016561 | 7,56 | 1,13 | 0,011258 | Ns5atp9 |
| ENSRNOG00000003029 | 1220,10 | 0,36 | 0,011748 | Calr |
| ENSRNOG00000028717 | 406,65 | -0,52 | 0,0118 | Ndufb7 |
| ENSRNOG00000045884 | 24,78 | -1,11 | 0,011838 |  |
| ENSRNOG00000018118 | 76,74 | -0,81 | 0,012087 | Atad3a |
| ENSRNOG00000036960 | 286,63 | 0,59 | 0,012119 | Abcc9 |
| ENSRNOG00000015866 | 69,92 | -0,72 | 0,012191 | Hint2 |
| ENSRNOG00000024671 | 214,01 | 0,62 | 0,012322 | Dmxl1 |
| ENSRNOG00000018655 | 128,42 | -0,67 | 0,012338 | Adsl |
| ENSRNOG00000028594 | 175,28 | 0,55 | 0,012361 | Ifnar1 |
| ENSRNOG00000015734 | 259,95 | 0,63 | 0,012361 | Ube3a |
| ENSRNOG00000002926 | 139,03 | 0,79 | 0,012374 | Uap1l2 |
| ENSRNOG00000013514 | 256,98 | -0,57 | 0,012374 | Maf1 |
| ENSRNOG00000020068 | 106,43 | -0,67 | 0,012398 | Ndufaf3 |
| ENSRNOG00000025899 | 58,18 | -0,96 | 0,012596 | Thada |
| ENSRNOG00000018755 | 148,40 | -0,57 | 0,01264 | Acss2 |
| ENSRNOG00000030963 | 18,78 | -1,17 | 0,01264 | LOC108351137 |
| ENSRNOG00000003882 | 138,41 | 0,66 | 0,012667 | Cep350 |
| ENSRNOG00000049653 | 45,96 | 1,09 | 0,012678 |  |
| ENSRNOG00000025324 | 47,69 | 0,85 | 0,012815 | Spire1 |
| ENSRNOG00000020288 | 677,19 | -0,41 | 0,012815 | Slc25a20 |
| ENSRNOG00000007324 | 276,05 | 0,61 | 0,012833 | Plxna2 |
| ENSRNOG00000020029 | 80,82 | -0,83 | 0,012938 | Mcrip2 |
| ENSRNOG00000016592 | 860,53 | 0,40 | 0,013077 | Gnai2 |
| ENSRNOG00000018012 | 95,22 | 0,71 | 0,013087 | Tulp4 |
| ENSRNOG00000014543 | 140,55 | 0,60 | 0,013131 | Tbc1d2b |
| ENSRNOG00000011504 | 529,24 | 0,54 | 0,013137 | Akap2 |
| ENSRNOG00000013956 | 39,97 | 1,09 | 0,013194 | Rnf38 |
| ENSRNOG00000013110 | 59,65 | -0,71 | 0,013194 | Nudt2 |
| ENSRNOG00000019428 | 185,29 | -0,57 | 0,013331 | Higd1a |
| ENSRNOG00000012074 | 326,27 | 0,62 | 0,013386 | Ifngr1 |
| ENSRNOG00000028746 | 226,52 | 0,50 | 0,013565 | Gsto1 |
| ENSRNOG00000008236 | 789,97 | -0,55 | 0,01368 | Decr1 |
| ENSRNOG00000002194 | 100,35 | -0,66 | 0,013688 | Coq2 |
| ENSRNOG00000048096 | 26,52 | 1,04 | 0,013812 |  |
| ENSRNOG00000020897 | 284,72 | -0,56 | 0,013836 | Prpf19 |
| ENSRNOG00000010320 | 390,65 | -0,53 | 0,013836 | Efnb3 |
| ENSRNOG00000014289 | 443,25 | 0,42 | 0,013836 | Arpc2 |
| ENSRNOG00000011015 | 136,17 | 0,67 | 0,013836 | Hivep2 |
| ENSRNOG00000001719 | 666,17 | -0,37 | 0,014043 | Psmd2 |
| ENSRNOG00000014996 | 20,28 | 1,02 | 0,014068 | Katna1 |
| ENSRNOG00000004841 | 760,61 | 0,49 | 0,014125 | Akap6 |
| ENSRNOG00000021139 | 204,76 | -0,60 | 0,014148 | Esrra |
| ENSRNOG00000034180 | 19,62 | 1,08 | 0,014307 | LOC100360781 |
| ENSRNOG00000004812 | 105,06 | 0,68 | 0,01434 | Sema6d |
| ENSRNOG00000002345 | 8,88 | 1,16 | 0,014369 | Rasgef1b |
| ENSRNOG00000003732 | 21,15 | 1,12 | 0,014485 | Flrt2 |
| ENSRNOG00000000645 | 89,36 | 0,71 | 0,014572 | Reep3 |
| ENSRNOG00000019851 | 4401,99 | -0,52 | 0,014572 | Cox6a2 |
| ENSRNOG00000015971 | 137,48 | 0,66 | 0,014625 | Slc12a2 |
| ENSRNOG00000004172 | 478,37 | -0,48 | 0,014688 | Pdk2 |
| ENSRNOG00000010945 | 421,94 | 0,44 | 0,014746 | Ywhab |
| ENSRNOG00000023781 | 1829,04 | -0,30 | 0,014828 | Plec |
| ENSRNOG00000012807 | 109,26 | 0,63 | 0,014867 | C1qa |
| ENSRNOG00000008103 | 3654,63 | -0,29 | 0,014867 | Mdh1 |
| ENSRNOG00000001376 | 111,71 | 0,65 | 0,01496 | Mettl7a |
| ENSRNOG00000018838 | 16,60 | -1,09 | 0,015042 | Paox |
| ENSRNOG00000011796 | 441,96 | 0,50 | 0,015042 | C1r |
| ENSRNOG00000002135 | 571,25 | -0,37 | 0,015074 | Sgcb |
| ENSRNOG00000000064 | 411,16 | -0,57 | 0,015148 | Atp5i |
| ENSRNOG00000006632 | 154,32 | 0,62 | 0,015277 | Rps6ka3 |
| ENSRNOG00000038686 | 119,85 | 0,63 | 0,015409 | Ap1s2 |
| ENSRNOG00000010646 | 27,75 | 0,96 | 0,015409 | Tmem229b |
| ENSRNOG00000046186 | 5,81 | 1,16 | 0,015473 |  |
| ENSRNOG00000022838 | 46,27 | -0,81 | 0,015634 | Cnksr1 |
| ENSRNOG00000018241 | 268,51 | -0,52 | 0,015646 | Ank1 |
| ENSRNOG00000018020 | 113,53 | -0,75 | 0,016042 | Apbb1 |
| ENSRNOG00000011339 | 339,72 | 0,49 | 0,016042 | Slk |
| ENSRNOG00000018507 | 41,14 | 0,92 | 0,016089 | Gfpt1 |
| ENSRNOG00000017780 | 53,53 | -0,89 | 0,016251 | Akr7a2 |
| ENSRNOG00000020304 | 45,78 | -0,79 | 0,01645 | Pdcd11 |
| ENSRNOG00000008533 | 70,12 | 0,82 | 0,01645 | Ago2 |
| ENSRNOG00000039197 | 99,95 | 0,68 | 0,016486 | LOC108348074 |
| ENSRNOG00000006542 | 497,69 | -0,45 | 0,016923 | Atp6v0c |
| ENSRNOG00000002373 | 369,93 | -0,35 | 0,01699 | Akap1 |
| ENSRNOG00000016181 | 73,82 | 0,80 | 0,01699 | Tbx20 |
| ENSRNOG00000003729 | 29,69 | -0,96 | 0,017092 |  |
| ENSRNOG00000000440 | 97,59 | 0,57 | 0,017317 | Pbx2 |
| ENSRNOG00000006170 | 10,75 | 1,15 | 0,017317 | Bach2 |
| ENSRNOG00000050806 | 19,82 | 1,03 | 0,017317 | LOC100365259 |
| ENSRNOG00000012346 | 212,13 | 0,63 | 0,017317 | Epb41l2 |
| ENSRNOG00000011619 | 130,79 | 0,73 | 0,017412 | Myo9a |
| ENSRNOG00000008141 | 750,68 | -0,66 | 0,017479 | Nppb |
| ENSRNOG00000027938 | 220,68 | 0,61 | 0,017546 | RGD1562037 |
| ENSRNOG00000000394 | 87,83 | 0,71 | 0,017546 | Srgn |
| ENSRNOG00000003999 | 18,11 | 1,10 | 0,017596 | Adcy3 |
| ENSRNOG00000016206 | 29,73 | 1,00 | 0,017616 | Uvrag |
| ENSRNOG00000049541 | 926,15 | -0,40 | 0,017646 |  |
| ENSRNOG00000018901 | 71,45 | 0,69 | 0,017648 | Rab14 |
| ENSRNOG00000014130 | 6,19 | 1,06 | 0,017798 | Cks2 |
| ENSRNOG00000015078 | 351,00 | 0,48 | 0,017805 | Ifitm3 |
| ENSRNOG00000005492 | 107,60 | 0,65 | 0,017861 | Hpcal1 |
| ENSRNOG00000006937 | 31,67 | 0,98 | 0,017924 |  |
| ENSRNOG00000014479 | 260,74 | 0,61 | 0,017936 | Cttnbp2nl |
| ENSRNOG00000002191 | 15,00 | -1,12 | 0,018052 | LOC498368 |
| ENSRNOG00000031643 | 134,14 | 0,64 | 0,018171 | Dchs1 |
| ENSRNOG00000008557 | 98,18 | -0,65 | 0,018171 | Abcb8 |
| ENSRNOG00000050646 | 361,35 | -0,55 | 0,01827 | Fem1a |
| ENSRNOG00000047247 | 335,36 | -0,61 | 0,01827 | Ptprs |
| ENSRNOG00000010299 | 109,35 | 0,65 | 0,01827 | Eps15 |
| ENSRNOG00000024243 | 5,81 | 1,08 | 0,018316 | Cadm4 |
| ENSRNOG00000009756 | 370,68 | -0,53 | 0,018359 | Pacsin2 |
| ENSRNOG00000000593 | 89,23 | 0,61 | 0,018632 | Rev3l |
| ENSRNOG00000018246 | 41,07 | -0,87 | 0,018636 | Tdp2 |
| ENSRNOG00000030351 | 6,87 | 1,13 | 0,0194 |  |
| ENSRNOG00000003312 | 40,47 | 1,05 | 0,019582 |  |
| ENSRNOG00000010859 | 78,02 | 0,76 | 0,019649 | Zyg11b |
| ENSRNOG00000003717 | 73,91 | 0,73 | 0,019688 | Cnih4 |
| ENSRNOG00000008415 | 19,18 | 1,02 | 0,019719 | Nab2 |
| ENSRNOG00000028103 | 314,45 | -0,52 | 0,019771 | Psmd3 |
| ENSRNOG00000007888 | 29,84 | -1,03 | 0,01979 |  |
| ENSRNOG00000001752 | 31,17 | 0,96 | 0,01983 | Nrros |
| ENSRNOG00000033722 | 77,74 | -0,86 | 0,019969 | Rnf207 |
| ENSRNOG00000028292 | 81,32 | -0,70 | 0,02023 | Gart |
| ENSRNOG00000002635 | 52,11 | -0,78 | 0,0203 | Dexi |
| ENSRNOG00000007447 | 40,14 | -1,02 | 0,02034 | Pla2g4b |
| ENSRNOG00000015941 | 52,60 | 0,81 | 0,020482 | Fkbp10 |
| ENSRNOG00000003929 | 47,00 | 0,83 | 0,020512 | Pcdh19 |
| ENSRNOG00000047625 | 71,37 | -0,71 | 0,020612 | Atg4d |
| ENSRNOG00000011157 | 497,33 | 0,42 | 0,020778 | Jak1 |
| ENSRNOG00000008056 | 55,63 | -0,90 | 0,020805 | Ankrd9 |
| ENSRNOG00000018281 | 1814,15 | -0,31 | 0,020953 | Uqcrfs1 |
| ENSRNOG00000016541 | 128,85 | 0,69 | 0,020992 | Enc1 |
| ENSRNOG00000011614 | 105,55 | 0,59 | 0,021105 | Tmcc1 |
| ENSRNOG00000023700 | 39,09 | -0,90 | 0,021112 | RGD1311847 |
| ENSRNOG00000001608 | 4,97 | -1,10 | 0,021112 | Nxpe3 |
| ENSRNOG00000000130 | 299,62 | -0,60 | 0,021324 | Dnajb5 |
| ENSRNOG00000017579 | 51,36 | 0,82 | 0,021484 | Mylip |
| ENSRNOG00000017294 | 73,03 | 0,72 | 0,021515 | Zranb1 |
| ENSRNOG00000012831 | 62,39 | -0,71 | 0,021718 |  |
| ENSRNOG00000005802 | 491,26 | 0,49 | 0,021718 | Usp24 |
| ENSRNOG00000046261 | 11,29 | 1,12 | 0,021742 | Acp5 |
| ENSRNOG00000007519 | 82,37 | 0,72 | 0,02186 | Tmem43 |
| ENSRNOG00000010298 | 190,33 | -0,53 | 0,02186 | Xbp1 |
| ENSRNOG00000017337 | 20,58 | 0,97 | 0,021975 |  |
| ENSRNOG00000002803 | 92,23 | 0,69 | 0,021982 |  |
| ENSRNOG00000045728 | 86,45 | 0,73 | 0,021997 |  |
| ENSRNOG00000020457 | 770,43 | -0,33 | 0,021997 | Tacc2 |
| ENSRNOG00000003211 | 140,37 | -0,57 | 0,022118 | Srp9 |
| ENSRNOG00000016165 | 26,38 | 1,04 | 0,022139 |  |
| ENSRNOG00000014532 | 22,61 | 0,90 | 0,022156 | Lbp |
| ENSRNOG00000016754 | 43,67 | -0,82 | 0,022156 | Bcs1l |
| ENSRNOG00000015439 | 78,41 | 0,63 | 0,02223 | Man2a1 |
| ENSRNOG00000012397 | 75,51 | 0,77 | 0,022334 | Zmym4 |
| ENSRNOG00000009585 | 110,31 | 0,61 | 0,022375 | Tcf20 |
| ENSRNOG00000007827 | 29,54 | 0,95 | 0,022538 | Cox4i2 |
| ENSRNOG00000011825 | 217,50 | 0,39 | 0,022685 | Ndufb3 |
| ENSRNOG00000006783 | 13,18 | -1,10 | 0,022685 | Neb |
| ENSRNOG00000015068 | 108,11 | -0,65 | 0,022834 | Il11ra1 |
| ENSRNOG00000025731 | 12,46 | 1,03 | 0,022834 | LOC100359583 |
| ENSRNOG00000028208 | 55,13 | -0,77 | 0,022834 | Mief2 |
| ENSRNOG00000023453 | 113,98 | 0,63 | 0,022834 | Lrba |
| ENSRNOG00000005711 | 69,25 | 0,75 | 0,022844 | Ptprd |
| ENSRNOG00000024786 | 14,94 | -1,10 | 0,023036 | Asb6 |
| ENSRNOG00000007040 | 262,67 | -0,46 | 0,023038 | Timm17a |
| ENSRNOG00000003069 | 132,47 | 0,76 | 0,023089 | Cd38 |
| ENSRNOG00000003341 | 34,13 | -0,84 | 0,023308 |  |
| ENSRNOG00000010361 | 43,86 | 0,82 | 0,023348 | Kif3b |
| ENSRNOG00000049437 | 1324,30 | -0,42 | 0,023348 | Gpc1 |
| ENSRNOG00000046493 | 183,17 | -0,51 | 0,023348 | Pnpo |
| ENSRNOG00000020813 | 149,24 | 0,56 | 0,023348 | Ltbp3 |
| ENSRNOG00000018057 | 82,47 | -0,76 | 0,023348 | Mrpl2 |
| ENSRNOG00000045961 | 86,90 | 0,61 | 0,023442 | Lyrm7 |
| ENSRNOG00000019162 | 11,76 | -1,08 | 0,023679 | Emc9 |
| ENSRNOG00000015456 | 32,35 | -0,88 | 0,023679 | Zfp787 |
| ENSRNOG00000015202 | 41,10 | 0,81 | 0,023679 | Dnajc5 |
| ENSRNOG00000015642 | 1377,63 | -0,29 | 0,023982 | Pabpc4 |
| ENSRNOG00000017692 | 90,34 | -0,65 | 0,02401 | Ppp1r37 |
| ENSRNOG00000014597 | 33,57 | 1,06 | 0,02401 | Irs1 |
| ENSRNOG00000021525 | 117,76 | 0,70 | 0,02401 | Nbeal1 |
| ENSRNOG00000007359 | 8,55 | -1,10 | 0,024022 | Gca |
| ENSRNOG00000012368 | 7,05 | -1,10 | 0,024022 | Dnajc17 |
| ENSRNOG00000020657 | 119,85 | 0,60 | 0,024044 | Shc1 |
| ENSRNOG00000018211 | 104,12 | -0,86 | 0,024336 | Urod |
| ENSRNOG00000001517 | 455,93 | -0,34 | 0,024399 | Pdk1 |
| ENSRNOG00000026034 | 300,17 | 0,48 | 0,024399 |  |
| ENSRNOG00000013505 | 1028,74 | -0,32 | 0,024399 | Vdac2 |
| ENSRNOG00000014948 | 35,63 | -0,90 | 0,024399 | Osgin1 |
| ENSRNOG00000018044 | 3887,35 | -0,30 | 0,024594 | Phyh |
| ENSRNOG00000001064 | 175,61 | 0,54 | 0,024594 | Sbno1 |
| ENSRNOG00000007102 | 526,05 | -0,52 | 0,024708 | Acss1 |
| ENSRNOG00000001055 | 580,03 | -0,38 | 0,024888 | Rilpl1 |
| ENSRNOG00000010266 | 5,08 | 1,09 | 0,024888 | Cd180 |
| ENSRNOG00000003721 | 32,73 | -0,85 | 0,024888 | Paqr4 |
| ENSRNOG00000018666 | 594,91 | -0,38 | 0,025065 | Gpsm1 |
| ENSRNOG00000014080 | 12,89 | 1,07 | 0,025077 | Kif23 |
| ENSRNOG00000050156 | 5,74 | 1,08 | 0,025156 |  |
| ENSRNOG00000019743 | 94,57 | -0,65 | 0,025156 | Tmem63b |
| ENSRNOG00000047179 | 1054,96 | 0,34 | 0,025156 | Aplp2 |
| ENSRNOG00000027880 | 50,00 | -0,79 | 0,025156 | Nbeal2 |
| ENSRNOG00000014617 | 349,92 | 0,41 | 0,025164 | Tgoln2 |
| ENSRNOG00000013324 | 528,34 | 0,57 | 0,025171 | Cdh5 |
| ENSRNOG00000001128 | 521,69 | -0,45 | 0,025278 | Tesc |
| ENSRNOG00000018414 | 111,04 | 0,67 | 0,025311 | Csf1r |
| ENSRNOG00000009823 | 73,81 | -0,65 | 0,025311 | Naa38 |
| ENSRNOG00000014395 | 16,34 | 1,09 | 0,025421 | Gli3 |
| ENSRNOG00000008377 | 80,11 | -0,76 | 0,025532 | Wdtc1 |
| ENSRNOG00000016062 | 158,94 | -0,64 | 0,025593 | Snta1 |
| ENSRNOG00000042467 | 11,43 | -1,08 | 0,025593 | Ttc4 |
| ENSRNOG00000012036 | 11,55 | 1,04 | 0,025872 | Pcsk5 |
| ENSRNOG00000031802 | 184,22 | 0,59 | 0,026028 |  |
| ENSRNOG00000018835 | 35,15 | 1,02 | 0,026116 | Notch2 |
| ENSRNOG00000016827 | 126,78 | -0,82 | 0,026116 | Slc38a3 |
| ENSRNOG00000009705 | 7,54 | 1,05 | 0,026116 | Lck |
| ENSRNOG00000005249 | 68,94 | 0,92 | 0,0262 | Snx6 |
| ENSRNOG00000018989 | 120,00 | -0,70 | 0,0262 |  |
| ENSRNOG00000018618 | 134,03 | 0,55 | 0,026343 |  |
| ENSRNOG00000001700 | 124,78 | -0,64 | 0,026343 | Whrn |
| ENSRNOG00000006609 | 711,84 | 0,42 | 0,026375 |  |
| ENSRNOG00000003712 | 168,83 | 0,53 | 0,02642 | Ppp4r3b |
| ENSRNOG00000006309 | 64,66 | 0,80 | 0,026433 |  |
| ENSRNOG00000027756 | 61,19 | 0,84 | 0,026433 | Usf3 |
| ENSRNOG00000014173 | 168,10 | 0,56 | 0,026441 | Smc3 |
| ENSRNOG00000031233 | 38,95 | -0,79 | 0,026525 | Mapk12 |
| ENSRNOG00000013720 | 124,17 | 0,75 | 0,026947 | Aebp1 |
| ENSRNOG00000012210 | 91,51 | 0,71 | 0,026976 | Sptlc2 |
| ENSRNOG00000004559 | 231,44 | 0,49 | 0,027028 |  |
| ENSRNOG00000021918 | 22,72 | 0,93 | 0,027128 | Cep76 |
| ENSRNOG00000001815 | 917,29 | -0,30 | 0,027189 | Eif4a2 |
| ENSRNOG00000037645 | 85,47 | 0,79 | 0,027296 | Tceal7 |
| ENSRNOG00000004052 | 348,93 | 0,50 | 0,027352 |  |
| ENSRNOG00000017286 | 173,56 | -0,52 | 0,027352 | Ephx2 |
| ENSRNOG00000007916 | 216,24 | 0,57 | 0,02744 | Ptk2 |
| ENSRNOG00000023803 | 3378,41 | 0,35 | 0,02744 | Cmya5 |
| ENSRNOG00000005984 | 26,05 | 0,93 | 0,02744 | Etv6 |
| ENSRNOG00000019627 | 32,56 | 0,85 | 0,027544 | Mybpc2 |
| ENSRNOG00000020244 | 609,79 | -0,45 | 0,027544 | Perm1 |
| ENSRNOG00000018232 | 310,43 | -0,40 | 0,027544 | Srf |
| ENSRNOG00000025184 | 7,02 | 1,03 | 0,027544 | Prss35 |
| ENSRNOG00000003134 | 41,93 | 0,83 | 0,027544 | Slc4a4 |
| ENSRNOG00000005823 | 39,97 | 0,80 | 0,027544 | Utp20 |
| ENSRNOG00000025644 | 21,66 | 0,88 | 0,02771 | LOC499331 |
| ENSRNOG00000019525 | 1016,97 | -0,44 | 0,02776 | Hspa9 |
| ENSRNOG00000030712 | 162,18 | -0,49 | 0,02776 | RT1-A2 |
| ENSRNOG00000009354 | 62,55 | 0,79 | 0,0279 | Nrarp |
| ENSRNOG00000004602 | 88,31 | 0,57 | 0,0279 | Rnf145 |
| ENSRNOG00000009790 | 156,70 | -0,66 | 0,028268 | Kcnk3 |
| ENSRNOG00000027784 | 27,22 | 0,98 | 0,028292 | Tsku |
| ENSRNOG00000003084 | 344,07 | -0,40 | 0,028372 | Parp1 |
| ENSRNOG00000011541 | 288,00 | 0,46 | 0,028634 | Cygb |
| ENSRNOG00000020962 | 74,26 | -0,59 | 0,028634 |  |
| ENSRNOG00000013128 | 131,87 | -0,56 | 0,028634 | Tmem179 |
| ENSRNOG00000019850 | 330,89 | -0,46 | 0,028863 | Speg |
| ENSRNOG00000008283 | 49,32 | 0,84 | 0,028873 | B4galt5 |
| ENSRNOG00000022599 | 72,79 | -0,68 | 0,028873 |  |
| ENSRNOG00000004686 | 338,81 | -0,37 | 0,028906 | Spop |
| ENSRNOG00000030790 | 258,14 | 0,44 | 0,029004 | Ctnnd1 |
| ENSRNOG00000008678 | 25,22 | 1,00 | 0,029062 | Antxr1 |
| ENSRNOG00000027988 | 87,33 | 0,85 | 0,029062 | Zhx3 |
| ENSRNOG00000005126 | 14,35 | 1,07 | 0,029062 | Pqlc3 |
| ENSRNOG00000008193 | 99,49 | 0,64 | 0,029062 | Cr1l |
| ENSRNOG00000017174 | 17,07 | 1,03 | 0,029062 |  |
| ENSRNOG00000034161 | 66,70 | -0,84 | 0,029148 | LOC688869 |
| ENSRNOG00000009207 | 27,40 | 0,82 | 0,029148 | Spata2 |
| ENSRNOG00000010091 | 148,56 | 0,59 | 0,029148 | Efcab14 |
| ENSRNOG00000014209 | 87,68 | 0,55 | 0,02922 | Utp6 |
| ENSRNOG00000009325 | 136,52 | 0,56 | 0,029222 | Fuca1 |
| ENSRNOG00000017060 | 5801,74 | 0,35 | 0,029222 | Ryr2 |
| ENSRNOG00000023257 | 276,68 | 0,54 | 0,029275 | Adamts9 |
| ENSRNOG00000012266 | 50,15 | 0,75 | 0,029396 | Zcchc17 |
| ENSRNOG00000000812 | 198,53 | -0,51 | 0,029412 | RGD1302996 |
| ENSRNOG00000017621 | 35,02 | -0,75 | 0,029421 | Spns1 |
| ENSRNOG00000048723 | 88,23 | 0,69 | 0,029493 | Pros1 |
| ENSRNOG00000028357 | 132,05 | -0,64 | 0,029684 | Lrrc14b |
| ENSRNOG00000004289 | 56,89 | 0,87 | 0,030137 |  |
| ENSRNOG00000011134 | 510,21 | 0,54 | 0,030137 | Lama2 |
| ENSRNOG00000010580 | 104,88 | -0,75 | 0,030445 | Acot7 |
| ENSRNOG00000010208 | 56,80 | 0,99 | 0,030635 | Timp1 |
| ENSRNOG00000011949 | 964,60 | -0,40 | 0,030676 | Ndufb5 |
| ENSRNOG00000019758 | 169,18 | -0,57 | 0,030682 | Ipo13 |
| ENSRNOG00000017154 | 82,84 | 0,66 | 0,030682 | Atp11a |
| ENSRNOG00000019727 | 18,02 | -1,01 | 0,030795 | Dph3 |
| ENSRNOG00000001009 | 114,66 | -0,57 | 0,030862 | Bri3 |
| ENSRNOG00000018708 | 364,97 | -0,41 | 0,030862 | Ppp1ca |
| ENSRNOG00000009175 | 35,09 | -0,80 | 0,031191 | Jagn1 |
| ENSRNOG00000001584 | 33,31 | 0,91 | 0,031272 | Map3k7cl |
| ENSRNOG00000046918 | 223,92 | 0,46 | 0,031546 | Apoo |
| ENSRNOG00000016655 | 18,88 | -1,00 | 0,031677 | Pex6 |
| ENSRNOG00000002074 | 36,19 | 0,81 | 0,031702 | Rest |
| ENSRNOG00000011419 | 1463,21 | -0,44 | 0,031978 | Aldh6a1 |
| ENSRNOG00000020345 | 97,23 | -0,72 | 0,031996 | Slc25a42 |
| ENSRNOG00000030654 | 71,44 | -0,74 | 0,031996 | Man2c1 |
| ENSRNOG00000007997 | 103,26 | 0,63 | 0,032122 |  |
| ENSRNOG00000016156 | 12,48 | 0,96 | 0,032246 | Nptxr |
| ENSRNOG00000003491 | 61,33 | 0,69 | 0,032326 | Prkca |
| ENSRNOG00000019041 | 587,65 | -0,42 | 0,032702 | Psme1 |
| ENSRNOG00000009266 | 247,41 | 0,46 | 0,032805 | Anp32b |
| ENSRNOG00000002041 | 69,87 | 0,71 | 0,03295 | Boc |
| ENSRNOG00000011677 | 41,30 | 0,89 | 0,032951 | Slc39a10 |
| ENSRNOG00000002177 | 36,91 | 0,88 | 0,033004 | Gnpda2 |
| ENSRNOG00000002141 | 80,87 | 0,81 | 0,033004 | Cd200 |
| ENSRNOG00000015160 | 16,00 | 1,05 | 0,033004 | Gem |
| ENSRNOG00000014836 | 123,16 | 0,53 | 0,0331 | Ralgapb |
| ENSRNOG00000004677 | 95,47 | 0,71 | 0,033317 | Zeb2 |
| ENSRNOG00000014182 | 2544,67 | 0,32 | 0,033317 | Tns1 |
| ENSRNOG00000008329 | 583,68 | -0,42 | 0,033444 | Ndufb11 |
| ENSRNOG00000017231 | 134,75 | 0,61 | 0,033517 | Adam9 |
| ENSRNOG00000048278 | 12,26 | 1,05 | 0,033573 |  |
| ENSRNOG00000019297 | 74,41 | 0,64 | 0,03358 |  |
| ENSRNOG00000017087 | 170,98 | -0,59 | 0,034178 | Man1c1 |
| ENSRNOG00000010319 | 99,43 | 0,56 | 0,034178 | Lcp1 |
| ENSRNOG00000000480 | 49,55 | -0,76 | 0,034241 | Phf1 |
| ENSRNOG00000003873 | 453,37 | 0,47 | 0,034439 | Cpd |
| ENSRNOG00000004763 | 115,76 | 0,52 | 0,034439 | Sirpa |
| ENSRNOG00000006000 | 99,28 | 0,58 | 0,034548 | Cdk12 |
| ENSRNOG00000020022 | 15,28 | -1,02 | 0,034548 | Psmc3ip |
| ENSRNOG00000002248 | 126,98 | 0,57 | 0,034548 | Fryl |
| ENSRNOG00000018126 | 55,08 | 0,92 | 0,034557 | Abca1 |
| ENSRNOG00000011261 | 22,14 | -1,01 | 0,034557 | Ttc14 |
| ENSRNOG00000007202 | 42,17 | 0,82 | 0,03484 | Sema3d |
| ENSRNOG00000001205 | 151,13 | -0,58 | 0,03484 | Agpat3 |
| ENSRNOG00000014936 | 204,07 | 0,47 | 0,034904 | Ifitm2 |
| ENSRNOG00000023919 | 280,68 | -0,51 | 0,034927 | Gbas |
| ENSRNOG00000008536 | 37587,28 | -0,31 | 0,034927 | Actc1 |
| ENSRNOG00000036604 | 153,78 | 0,69 | 0,034927 | Ifit2 |
| ENSRNOG00000010068 | 101,38 | -0,63 | 0,035095 | Plpp7 |
| ENSRNOG00000021270 | 27,28 | 0,83 | 0,035095 | Trmt6 |
| ENSRNOG00000012457 | 1480,03 | -0,45 | 0,035162 | Cyc1 |
| ENSRNOG00000024039 | 137,20 | 0,56 | 0,035162 | Serinc5 |
| ENSRNOG00000008933 | 305,03 | -0,44 | 0,035162 | Plbd1 |
| ENSRNOG00000050543 | 175,63 | -0,47 | 0,035162 | Safb |
| ENSRNOG00000033176 | 6,12 | -1,04 | 0,035175 | Zrsr2 |
| ENSRNOG00000031506 | 5,07 | 1,00 | 0,035175 | LOC100360087 |
| ENSRNOG00000000541 | 237,17 | -0,57 | 0,035175 | Glo1 |
| ENSRNOG00000001091 | 157,08 | -0,45 | 0,035315 | Hip1r |
| ENSRNOG00000014999 | 96,82 | 0,56 | 0,035315 | Tnpo1 |
| ENSRNOG00000018687 | 90,92 | -0,70 | 0,035327 | Fbxw2 |
| ENSRNOG00000002871 | 73,07 | 0,77 | 0,035519 | Rbm25l1 |
| ENSRNOG00000019977 | 43,41 | 0,94 | 0,03574 | Ptprf |
| ENSRNOG00000003150 | 343,94 | -0,40 | 0,03574 | Mpc2 |
| ENSRNOG00000030213 | 174,73 | 0,53 | 0,03574 | Vps13c |
| ENSRNOG00000020497 | 143,68 | -0,53 | 0,035753 | Plekha1 |
| ENSRNOG00000030183 | 80,42 | 0,93 | 0,035894 | Plod2 |
| ENSRNOG00000022392 | 1153,44 | -0,31 | 0,036059 | Hspb8 |
| ENSRNOG00000049784 | 159,08 | -0,44 | 0,036165 | Tsnax |
| ENSRNOG00000001712 | 33,15 | -0,85 | 0,036165 | Alg3 |
| ENSRNOG00000012956 | 1086,80 | 0,29 | 0,036174 | Tgm2 |
| ENSRNOG00000003846 | 237,31 | 0,45 | 0,036197 | Pitpna |
| ENSRNOG00000019780 | 19,83 | 1,02 | 0,036197 | Sypl2 |
| ENSRNOG00000005703 | 287,78 | 0,47 | 0,036197 | Arfgef1 |
| ENSRNOG00000019930 | 110,45 | -0,69 | 0,036623 | Rhot2 |
| ENSRNOG00000009552 | 566,70 | 0,37 | 0,036779 | Serinc3 |
| ENSRNOG00000027839 | 26,38 | 0,88 | 0,03684 | Ptk2b |
| ENSRNOG00000011852 | 54,68 | -0,86 | 0,036943 | Myo6 |
| ENSRNOG00000030954 | 630,81 | 0,48 | 0,036943 | Fat1 |
| ENSRNOG00000030237 | 780,13 | -0,39 | 0,037043 | Cox7c |
| ENSRNOG00000010912 | 82,20 | -0,66 | 0,037081 | Mrps25 |
| ENSRNOG00000011952 | 468,66 | -0,43 | 0,03717 | Samm50 |
| ENSRNOG00000036576 | 44,52 | 0,83 | 0,037245 | Zdhhc6 |
| ENSRNOG00000013577 | 42,76 | 0,75 | 0,037251 | Kdelr3 |
| ENSRNOG00000010017 | 88,35 | 0,64 | 0,037251 | Wee1 |
| ENSRNOG00000016090 | 66,82 | 0,66 | 0,037281 | Mtmr10 |
| ENSRNOG00000033772 | 22,47 | 0,91 | 0,037462 | Serpinb9 |
| ENSRNOG00000000457 | 58,16 | 0,85 | 0,037462 | Tap1 |
| ENSRNOG00000011781 | 78,64 | -0,60 | 0,037462 | Oplah |
| ENSRNOG00000015320 | 492,65 | -0,44 | 0,037757 | Atp5g2 |
| ENSRNOG00000012524 | 777,15 | 0,36 | 0,037757 | Zfp91 |
| ENSRNOG00000014076 | 161,81 | 0,49 | 0,037869 | Mbnl1 |
| ENSRNOG00000016731 | 95,21 | 0,70 | 0,038 | Tpm2 |
| ENSRNOG00000043167 | 89,56 | 0,63 | 0,038091 | Itga9 |
| ENSRNOG00000003855 | 211,36 | -0,49 | 0,038091 | Dnaja3 |
| ENSRNOG00000031669 | 39,14 | 0,92 | 0,038299 | Lpp |
| ENSRNOG00000014997 | 542,02 | 0,36 | 0,038303 | Igf2r |
| ENSRNOG00000014806 | 248,11 | -0,49 | 0,038344 | Pnkd |
| ENSRNOG00000020105 | 29,97 | -0,92 | 0,038344 | Klhl30 |
| ENSRNOG00000004737 | 259,37 | 0,61 | 0,038528 | Cd48 |
| ENSRNOG00000016779 | 266,94 | 0,38 | 0,038588 | Fam120a |
| ENSRNOG00000002795 | 73,58 | -0,60 | 0,038658 | Cog1 |
| ENSRNOG00000009439 | 1234,72 | 0,31 | 0,038658 | Eef1a1 |
| ENSRNOG00000033887 | 65,90 | -0,67 | 0,038742 |  |
| ENSRNOG00000023373 | 139,96 | 0,47 | 0,038815 | Sec24b |
| ENSRNOG00000004361 | 25,29 | -0,91 | 0,038831 | Parp10 |
| ENSRNOG00000033280 | 3905,13 | 0,32 | 0,039115 | Pam |
| ENSRNOG00000019773 | 125,80 | -0,53 | 0,039278 |  |
| ENSRNOG00000019147 | 118,54 | 0,58 | 0,039278 | Stom |
| ENSRNOG00000001596 | 2303,05 | -0,37 | 0,039362 | Atp5g3 |
| ENSRNOG00000020871 | 727,41 | 0,44 | 0,039412 | Ltbp4 |
| ENSRNOG00000047800 | 27,61 | 0,89 | 0,039659 | C5ar1 |
| ENSRNOG00000013604 | 400,62 | -0,45 | 0,039659 | Gpx4 |
| ENSRNOG00000006938 | 241,61 | -0,60 | 0,039735 |  |
| ENSRNOG00000027360 | 34,84 | -0,90 | 0,039735 | Srsf7 |
| ENSRNOG00000011329 | 593,62 | -0,38 | 0,039822 | Pkm |
| ENSRNOG00000029490 | 89,00 | -0,66 | 0,039846 | Znf768 |
| ENSRNOG00000039807 | 17,26 | -1,02 | 0,039993 | Fam193b |
| ENSRNOG00000002271 | 827,84 | 0,31 | 0,040204 | Slain2 |
| ENSRNOG00000010852 | 41,51 | 0,84 | 0,040224 | Nup205 |
| ENSRNOG00000003597 | 1101,96 | -0,40 | 0,040358 | Tuba4a |
| ENSRNOG00000015420 | 106,80 | 0,58 | 0,040358 | Stxbp1 |
| ENSRNOG00000001820 | 23,82 | 0,89 | 0,040386 | Med21 |
| ENSRNOG00000010783 | 32,53 | 0,89 | 0,04049 | Mak16 |
| ENSRNOG00000012207 | 3329,52 | 0,31 | 0,040595 | Dst |
| ENSRNOG00000028545 | 16,92 | 0,95 | 0,040643 |  |
| ENSRNOG00000008741 | 238,01 | 0,46 | 0,040789 | Camsap2 |
| ENSRNOG00000004110 | 99,76 | 0,51 | 0,040789 | Trib2 |
| ENSRNOG00000000341 | 39,37 | 0,81 | 0,040789 | Nid2 |
| ENSRNOG00000016543 | 168,73 | -0,50 | 0,040789 | Trim63 |
| ENSRNOG00000005861 | 92,43 | 0,64 | 0,040789 | Hsd11b1 |
| ENSRNOG00000015162 | 262,06 | -0,44 | 0,040872 | Lonp2 |
| ENSRNOG00000010461 | 22,74 | 0,80 | 0,0409 | Gpx8 |
| ENSRNOG00000013694 | 19,81 | 0,86 | 0,040903 | Ntng2 |
| ENSRNOG00000015416 | 78,56 | 0,70 | 0,04096 | Nabp1 |
| ENSRNOG00000005809 | 93,17 | 0,59 | 0,041378 | Arhgdib |
| ENSRNOG00000014297 | 100,75 | -0,72 | 0,04163 | Sdc4 |
| ENSRNOG00000016695 | 190,69 | 0,66 | 0,041868 | Mmp2 |
| ENSRNOG00000042829 | 41,35 | -0,85 | 0,042105 |  |
| ENSRNOG00000009928 | 397,46 | -0,41 | 0,042124 | Bckdhb |
| ENSRNOG00000014665 | 123,69 | -0,61 | 0,042189 | Dhdds |
| ENSRNOG00000013456 | 19,79 | -0,92 | 0,042189 | Ighmbp2 |
| ENSRNOG00000005633 | 7,64 | -1,01 | 0,042362 | Zbtb49 |
| ENSRNOG00000014050 | 136,69 | -0,57 | 0,042561 | Ptges2 |
| ENSRNOG00000002099 | 256,79 | 0,47 | 0,042584 |  |
| ENSRNOG00000013913 | 73,98 | 0,67 | 0,042871 |  |
| ENSRNOG00000016013 | 58,32 | 0,63 | 0,042875 | Gprc5b |
| ENSRNOG00000006412 | 108,66 | 0,54 | 0,043292 | Zhx1 |
| ENSRNOG00000000804 | 240,19 | -0,37 | 0,043463 | Mrps18b |
| ENSRNOG00000020684 | 229,81 | 0,51 | 0,043463 | Vat1 |
| ENSRNOG00000015206 | 64,80 | -0,70 | 0,043535 | Alad |
| ENSRNOG00000019740 | 69,00 | 0,74 | 0,043535 | Hdgfrp3 |
| ENSRNOG00000043114 | 121,65 | -0,53 | 0,043721 | Tomm7 |
| ENSRNOG00000046005 | 101,54 | 0,64 | 0,044046 | Scd2 |
| ENSRNOG00000021203 | 47,03 | 0,73 | 0,044053 | Atl3 |
| ENSRNOG00000018505 | 166,60 | -0,55 | 0,044111 | Cidea |
| ENSRNOG00000006865 | 894,26 | -0,34 | 0,044111 | Laptm4a |
| ENSRNOG00000020942 | 35,27 | 0,88 | 0,044111 | Plekha4 |
| ENSRNOG00000006154 | 25,25 | 0,91 | 0,044111 |  |
| ENSRNOG00000000974 | 47,25 | -0,79 | 0,044111 | Zfp358 |
| ENSRNOG00000002198 | 8,02 | 1,00 | 0,044373 |  |
| ENSRNOG00000017718 | 5,27 | -1,00 | 0,044373 | Tmco6 |
| ENSRNOG00000010666 | 13,61 | 1,00 | 0,044373 | Wisp2 |
| ENSRNOG00000008652 | 72,72 | 0,74 | 0,044399 | Phip |
| ENSRNOG00000021166 | 339,75 | 0,39 | 0,044505 | Ecm1 |
| ENSRNOG00000007377 | 50,98 | 0,86 | 0,044595 | Slit3 |
| ENSRNOG00000004540 | 29,08 | 0,84 | 0,044671 | Clec3b |
| ENSRNOG00000005762 | 92,37 | -0,54 | 0,044671 | Rab22a |
| ENSRNOG00000008461 | 51,75 | -0,73 | 0,044809 |  |
| ENSRNOG00000004442 | 118,99 | -0,56 | 0,044873 | RGD1311756 |
| ENSRNOG00000001399 | 61,82 | 0,73 | 0,044962 | Tbx5 |
| ENSRNOG00000018372 | 45,27 | -0,76 | 0,045095 | Cul9 |
| ENSRNOG00000042848 | 534,34 | 0,37 | 0,045302 |  |
| ENSRNOG00000014901 | 94,84 | 0,71 | 0,045398 | Uggt1 |
| ENSRNOG00000018461 | 209,31 | 0,57 | 0,045398 | Pdgfrb |
| ENSRNOG00000012406 | 149,69 | -0,61 | 0,045463 | Pcbp4 |
| ENSRNOG00000049785 | 67,49 | -0,80 | 0,045464 | Ranbp3 |
| ENSRNOG00000001645 | 323,53 | 0,57 | 0,045713 | Filip1l |
| ENSRNOG00000010814 | 102,14 | 0,53 | 0,045713 |  |
| ENSRNOG00000048101 | 7,67 | -0,99 | 0,045713 | Zfp397 |
| ENSRNOG00000014078 | 826,51 | -0,38 | 0,045713 | Ndufb8 |
| ENSRNOG00000012806 | 199,34 | 0,46 | 0,045713 | Rbbp6 |
| ENSRNOG00000007862 | 1796,62 | -0,34 | 0,045713 | Acat1 |
| ENSRNOG00000008820 | 92,10 | 0,70 | 0,045713 |  |
| ENSRNOG00000005606 | 134,79 | 0,52 | 0,045842 | Med1 |
| ENSRNOG00000012405 | 135,59 | 0,56 | 0,046088 | Tcf4 |
| ENSRNOG00000004378 | 27,65 | 0,97 | 0,046088 | Abca5 |
| ENSRNOG00000006180 | 423,95 | 0,37 | 0,046397 | Pum2 |
| ENSRNOG00000016790 | 135,76 | 0,45 | 0,046485 | Kmt5b |
| ENSRNOG00000005195 | 896,72 | 0,40 | 0,046532 | Cst3 |
| ENSRNOG00000014285 | 166,38 | 0,52 | 0,046563 | Ssh2 |
| ENSRNOG00000002956 | 85,13 | 0,57 | 0,046563 | Stim2 |
| ENSRNOG00000011953 | 136,88 | 0,41 | 0,046563 | Supt16h |
| ENSRNOG00000016581 | 22,03 | 0,99 | 0,046737 | Serpinb1a |
| ENSRNOG00000009968 | 14,50 | -0,99 | 0,046737 | Ercc8 |
| ENSRNOG00000004273 | 130,17 | 0,71 | 0,046737 | Ifitm1 |
| ENSRNOG00000003219 | 71,40 | 0,75 | 0,046864 | Trim16 |
| ENSRNOG00000018553 | 55,92 | -0,74 | 0,046965 | Pitpnm1 |
| ENSRNOG00000024823 | 22,35 | -0,95 | 0,046965 |  |
| ENSRNOG00000021526 | 85,81 | -0,81 | 0,046965 | Slc25a34 |
| ENSRNOG00000017851 | 670,68 | -0,38 | 0,047099 | Etfb |
| ENSRNOG00000017786 | 6726,27 | 0,46 | 0,047138 | Acta1 |
| ENSRNOG00000010637 | 64,51 | 0,70 | 0,047458 | Tbc1d5 |
| ENSRNOG00000005418 | 251,10 | -0,39 | 0,047608 | Trap1 |
| ENSRNOG00000005264 | 101,10 | 0,61 | 0,047652 | Sav1 |
| ENSRNOG00000011343 | 1876,41 | -0,41 | 0,047741 |  |
| ENSRNOG00000012808 | 132,24 | -0,54 | 0,047963 | Tmem259 |
| ENSRNOG00000020869 | 116,97 | -0,52 | 0,047992 | mrpl9 |
| ENSRNOG00000000841 | 168,49 | -0,53 | 0,048067 | Ddx39b |
| ENSRNOG00000018317 | 75,57 | 0,61 | 0,048151 | Aak1 |
| ENSRNOG00000001804 | 132,04 | 0,59 | 0,048431 |  |
| ENSRNOG00000020956 | 178,92 | -0,59 | 0,048505 | Bcat2 |
| ENSRNOG00000039593 | 37,85 | 0,80 | 0,048505 | Ecscr |
| ENSRNOG00000018011 | 71,40 | 0,57 | 0,048505 | Fam208b |
| ENSRNOG00000016180 | 263,22 | 0,43 | 0,048505 | Pdp1 |
| ENSRNOG00000011754 | 10855,91 | -0,24 | 0,048505 | Myom2 |
| ENSRNOG00000003890 | 298,44 | 0,48 | 0,048505 | Nap1l1 |
| ENSRNOG00000014454 | 65,25 | -0,64 | 0,048505 | Ap1m1 |
| ENSRNOG00000049402 | 115,66 | 0,60 | 0,048505 | Nbl1 |
| ENSRNOG00000000574 | 15,35 | 0,91 | 0,048598 |  |
| ENSRNOG00000020650 | 9,70 | -0,99 | 0,048598 | Slc17a7 |
| ENSRNOG00000008904 | 77,08 | 0,73 | 0,048598 | Fli1 |
| ENSRNOG00000000187 | 45,18 | 0,89 | 0,048755 | Csf2rb |
| ENSRNOG00000015977 | 145,47 | 0,45 | 0,048761 | Zfp609 |
| ENSRNOG00000033123 | 5,02 | -0,98 | 0,048761 | Pcdhb20 |
| ENSRNOG00000016538 | 19,52 | 0,98 | 0,048852 | Itga8 |
| ENSRNOG00000019472 | 9,69 | 0,98 | 0,048852 | Ackr2 |
| ENSRNOG00000000596 | 117,66 | 0,55 | 0,04887 | Fyn |
| ENSRNOG00000000900 | 21,23 | 0,96 | 0,049026 | Tpst1 |
| ENSRNOG00000013683 | 501,87 | 0,45 | 0,049343 | S1pr1 |
| ENSRNOG00000011476 | 14,68 | -0,97 | 0,049458 | Nars2 |
| ENSRNOG00000023383 | 892,37 | 0,28 | 0,04961 | Ddx3x |
| ENSRNOG00000005730 | 152,92 | 0,57 | 0,049838 | Pcmtd1 |
| ENSRNOG00000002280 | 145,64 | 0,58 | 0,049838 | Sh3bgrl |
| ENSRNOG00000001843 | 87,69 | 0,62 | 0,049919 | Bcl6 |
| ENSRNOG00000002841 | 293,13 | 0,44 | 0,050021 | Cdc42bpa |
| ENSRNOG00000017752 | 344,00 | -0,43 | 0,0501 | Mccc2 |
| ENSRNOG00000017832 | 142,93 | 0,53 | 0,0501 | Snx2 |
| ENSRNOG00000049471 | 45,43 | 0,87 | 0,050266 | Steap3 |
| ENSRNOG00000045859 | 8,82 | 0,95 | 0,050395 |  |
| ENSRNOG00000028945 | 696,01 | -0,34 | 0,050395 | Tmem182 |
| ENSRNOG00000033824 | 34,16 | 0,76 | 0,050479 | Gpd2 |
| ENSRNOG00000049378 | 106,02 | 0,47 | 0,050512 |  |
| ENSRNOG00000000488 | 13,50 | -0,94 | 0,050512 | Hmga1 |
| ENSRNOG00000017704 | 101,10 | 0,59 | 0,050512 | Sema3f |
| ENSRNOG00000031679 | 108,97 | -0,54 | 0,050554 |  |
| ENSRNOG00000001409 | 128,50 | -0,50 | 0,050554 | Gnb2 |
| ENSRNOG00000009080 | 399,80 | 0,38 | 0,050578 | Atp6v1d |
| ENSRNOG00000002450 | 32,22 | -0,79 | 0,050578 | Rrp15 |
| ENSRNOG00000033215 | 93,62 | 0,69 | 0,050668 | RT1-Db1 |
| ENSRNOG00000005470 | 35,69 | 0,81 | 0,050696 | Atxn3 |
| ENSRNOG00000016351 | 16,73 | 0,96 | 0,050712 | Frrs1 |
| ENSRNOG00000021725 | 69,58 | 0,68 | 0,050712 | Unc119b |
| ENSRNOG00000030245 | 501,63 | -0,36 | 0,050743 | Tango2 |
| ENSRNOG00000020479 | 291,70 | 0,48 | 0,05084 | Pik3c2a |
| ENSRNOG00000015753 | 302,05 | -0,42 | 0,050906 | Epn1 |
| ENSRNOG00000015594 | 8,21 | 0,97 | 0,051001 | Rftn2 |
| ENSRNOG00000011112 | 6,41 | -0,98 | 0,051086 | Fam161b |
| ENSRNOG00000004578 | 6,54 | 0,90 | 0,051086 | Cthrc1 |
| ENSRNOG00000037563 | 29,60 | 0,90 | 0,051168 | Cd68 |
| ENSRNOG00000012777 | 57,92 | -0,68 | 0,051459 |  |
| ENSRNOG00000033378 | 145,57 | -0,44 | 0,051459 | Rnf123 |
| ENSRNOG00000010558 | 165,02 | -0,50 | 0,051566 | Ppif |
| ENSRNOG00000013668 | 125,39 | 0,60 | 0,051601 | Capg |
| ENSRNOG00000008444 | 23,66 | 0,91 | 0,051601 | Cbl |
| ENSRNOG00000017546 | 1211,17 | -0,39 | 0,051754 | Mylk3 |
| ENSRNOG00000031031 | 103,29 | 0,69 | 0,051754 | Zfp292 |
| ENSRNOG00000008392 | 138,77 | -0,52 | 0,051754 | Sbf1 |
| ENSRNOG00000042980 | 272,66 | -0,40 | 0,051754 | Adam19 |
| ENSRNOG00000010372 | 54,16 | -0,69 | 0,051754 |  |
| ENSRNOG00000000777 | 30,99 | -0,88 | 0,051871 | RT1-S3 |
| ENSRNOG00000006325 | 226,20 | -0,53 | 0,051871 |  |
| ENSRNOG00000004317 | 6,28 | -0,97 | 0,051951 | Vipr2 |
| ENSRNOG00000009566 | 88,26 | 0,57 | 0,052024 | Phf12 |
| ENSRNOG00000042951 | 54,34 | 0,70 | 0,052147 | Xrn1 |
| ENSRNOG00000006663 | 133,51 | -0,65 | 0,052147 | Usp2 |
| ENSRNOG00000020185 | 79,90 | -0,54 | 0,052231 | Wdr6 |
| ENSRNOG00000038218 | 299,38 | -0,45 | 0,052231 | Ndufc1 |
| ENSRNOG00000032902 | 442,01 | -0,37 | 0,052438 | Ybx1-ps3 |
| ENSRNOG00000003682 | 5,83 | -0,96 | 0,052881 | Hook2 |
| ENSRNOG00000039214 | 11,46 | -0,97 | 0,052945 | RGD1305455 |
| ENSRNOG00000017443 | 28,73 | 0,95 | 0,053631 | Tmem87b |
| ENSRNOG00000018322 | 219,02 | 0,47 | 0,05367 | Picalm |
| ENSRNOG00000003224 | 22,66 | -0,93 | 0,053883 | Nudt16l1 |
| ENSRNOG00000032254 | 114,81 | 0,54 | 0,053883 | Sin3a |
| ENSRNOG00000008416 | 56,52 | 0,63 | 0,053883 | Gimap5 |
| ENSRNOG00000019097 | 169,99 | -0,41 | 0,053883 | Bap1 |
| ENSRNOG00000011077 | 497,23 | 0,38 | 0,054158 | Tjp1 |
| ENSRNOG00000020499 | 146,35 | -0,52 | 0,054158 |  |
| ENSRNOG00000004458 | 6,04 | 0,87 | 0,054216 | Ston2 |
| ENSRNOG00000000062 | 33,79 | 0,79 | 0,054216 | Pcgf3 |
| ENSRNOG00000008441 | 210,92 | 0,43 | 0,05472 |  |
| ENSRNOG00000021035 | 274,16 | -0,44 | 0,054764 | Rpl18 |
| ENSRNOG00000016088 | 47,11 | -0,81 | 0,054813 | Cdk10 |
| ENSRNOG00000019859 | 24,04 | 0,85 | 0,054826 | Pla2g15 |
| ENSRNOG00000011365 | 13,93 | 0,96 | 0,055003 |  |
| ENSRNOG00000000803 | 103,38 | 0,59 | 0,055003 |  |
| ENSRNOG00000018009 | 23,37 | 0,84 | 0,055003 | Rab8b |
| ENSRNOG00000012799 | 31,80 | 0,77 | 0,055003 | Prkaa1 |
| ENSRNOG00000026900 | 75,60 | -0,58 | 0,055124 | Ppil2 |
| ENSRNOG00000013689 | 23,99 | -0,87 | 0,055124 | Vps18 |
| ENSRNOG00000006593 | 245,71 | -0,43 | 0,055197 | Grpel1 |
| ENSRNOG00000010549 | 121,63 | -0,53 | 0,055415 | Tspo |
| ENSRNOG00000005184 | 50,88 | -0,81 | 0,055499 | Nop10 |
| ENSRNOG00000028733 | 6,83 | -0,96 | 0,055713 | Prkar1b |
| ENSRNOG00000042536 | 52,43 | 0,73 | 0,055713 | Pde4d |
| ENSRNOG00000010038 | 352,30 | -0,46 | 0,055713 | Psmc5 |
| ENSRNOG00000047194 | 37,09 | 0,90 | 0,05599 | Arl13b |
| ENSRNOG00000000248 | 247,96 | -0,48 | 0,05599 | Srsf2 |
| ENSRNOG00000007407 | 224,40 | 0,47 | 0,05599 | Ndufa12 |
| ENSRNOG00000000996 | 89,41 | -0,51 | 0,056182 | Arpc1a |
| ENSRNOG00000004613 | 33,16 | 0,79 | 0,056189 | Gpm6b |
| ENSRNOG00000014786 | 4,91 | 0,89 | 0,056283 | Ccne1 |
| ENSRNOG00000023661 | 39,35 | 0,81 | 0,056385 | Celf2 |
| ENSRNOG00000043486 | 38,15 | 0,77 | 0,05639 | Tnfrsf26 |
| ENSRNOG00000005281 | 35,30 | -0,72 | 0,05639 | Stx16 |
| ENSRNOG00000011154 | 966,34 | 0,43 | 0,056461 | Adgrf5 |
| ENSRNOG00000010960 | 336,05 | -0,48 | 0,056864 | Ankh |
| ENSRNOG00000018916 | 22,09 | -0,88 | 0,056977 | Bcl7c |
| ENSRNOG00000018946 | 61,18 | 0,73 | 0,057112 | Trim33 |
| ENSRNOG00000009300 | 169,71 | 0,53 | 0,057112 | LOC103690017 |
| ENSRNOG00000001971 | 21,26 | 0,86 | 0,057112 | Bbx |
| ENSRNOG00000006052 | 165,14 | 0,56 | 0,057112 | Sulf2 |
| ENSRNOG00000000704 | 45,66 | 0,76 | 0,057112 | Cmklr1 |
| ENSRNOG00000016810 | 38,34 | 0,71 | 0,057112 | Stmn1 |
| ENSRNOG00000000957 | 471,09 | 0,35 | 0,057112 | Rpl21 |
| ENSRNOG00000017250 | 450,08 | -0,43 | 0,057112 | Gmpr |
| ENSRNOG00000016265 | 119,58 | 0,56 | 0,057112 | Acsl5 |
| ENSRNOG00000011137 | 34,54 | 0,81 | 0,0574 | Zbtb41 |
| ENSRNOG00000029971 | 140960,34 | 0,78 | 0,057571 | ND5 |
| ENSRNOG00000037446 | 121,66 | -0,64 | 0,057583 | Pxmp2 |
| ENSRNOG00000030238 | 60,15 | -0,70 | 0,057583 | Fndc5 |
| ENSRNOG00000033426 | 450,00 | -0,37 | 0,057583 | Cdc37 |
| ENSRNOG00000017466 | 1157,06 | 0,41 | 0,057876 | Kif5b |
| ENSRNOG00000001259 | 19,77 | -0,93 | 0,057931 | Cux2 |
| ENSRNOG00000029980 | 228,18 | -0,38 | 0,058014 | Zbtb16 |
| ENSRNOG00000009987 | 185,56 | 0,45 | 0,05815 | Akap11 |
| ENSRNOG00000038200 | 13,15 | -0,95 | 0,058215 |  |
| ENSRNOG00000032922 | 27,40 | 0,84 | 0,058362 | Dclk1 |
| ENSRNOG00000021243 | 16,05 | 0,93 | 0,058521 | Siglec1 |
| ENSRNOG00000027049 | 982,56 | -0,37 | 0,059076 | LOC689271 |
| ENSRNOG00000008425 | 158,59 | 0,58 | 0,059076 | Nav1 |
| ENSRNOG00000018659 | 100,05 | 0,72 | 0,059162 | Csf1 |
| ENSRNOG00000006726 | 11,27 | 0,92 | 0,05932 | Zfp9 |
| ENSRNOG00000037655 | 104,91 | -0,57 | 0,059344 | Gatb |
| ENSRNOG00000003546 | 119,11 | -0,68 | 0,059758 | Tnfrsf12a |
| ENSRNOG00000048924 | 30,86 | 0,91 | 0,059766 | Islr |
| ENSRNOG00000017941 | 865,47 | 0,32 | 0,059962 | Optn |
| ENSRNOG00000003359 | 279,44 | -0,42 | 0,06012 | Ogt |
| ENSRNOG00000045771 | 10,38 | 0,95 | 0,060303 | Chl1 |
| ENSRNOG00000024800 | 17,05 | 0,89 | 0,060537 |  |
| ENSRNOG00000022162 | 62,61 | 0,68 | 0,060632 | Pbx3 |
| ENSRNOG00000017895 | 788,57 | -0,33 | 0,061066 | Eno1 |
| ENSRNOG00000014743 | 91,35 | -0,54 | 0,06138 | Hagh |
| ENSRNOG00000006077 | 94,37 | -0,52 | 0,061648 | Snx5 |
| ENSRNOG00000048237 | 195,96 | -0,52 | 0,061794 | Tcta |
| ENSRNOG00000004305 | 37,19 | 0,67 | 0,061966 | Abl2 |
| ENSRNOG00000010964 | 584,65 | 0,34 | 0,062017 | Akap13 |
| ENSRNOG00000003694 | 36,39 | 0,80 | 0,062034 | Prox1 |
| ENSRNOG00000017414 | 89,51 | -0,55 | 0,062034 | Irf7 |
| ENSRNOG00000012149 | 7,82 | 0,89 | 0,062034 | Gpsm2 |
| ENSRNOG00000039417 | 101,78 | -0,60 | 0,062034 | Dda1 |
| ENSRNOG00000000816 | 89,55 | 0,56 | 0,062176 | Ppp1r18 |
| ENSRNOG00000034303 | 100,08 | 0,73 | 0,062176 |  |
| ENSRNOG00000002881 | 69,02 | 0,69 | 0,062286 | Ddr2 |
| ENSRNOG00000000247 | 29,10 | -0,85 | 0,062286 | Mfsd11 |
| ENSRNOG00000007125 | 124,77 | 0,45 | 0,062386 | Vps54 |
| ENSRNOG00000030317 | 382,46 | 0,35 | 0,062637 |  |
| ENSRNOG00000003125 | 40,53 | -0,73 | 0,062637 | Rogdi |
| ENSRNOG00000014183 | 38,07 | 0,79 | 0,063014 | Gnaq |
| ENSRNOG00000010427 | 472,45 | 0,39 | 0,063128 | Ipo7 |
| ENSRNOG00000023053 | 104,20 | 0,57 | 0,063627 | Ice1 |
| ENSRNOG00000003720 | 16,18 | 0,91 | 0,063637 | Prrx1 |
| ENSRNOG00000001996 | 222,95 | 0,45 | 0,063637 | Ythdc1 |
| ENSRNOG00000017979 | 21,20 | -0,87 | 0,063637 | Mrto4 |
| ENSRNOG00000014398 | 30,04 | 0,79 | 0,063945 | Scara5 |
| ENSRNOG00000025716 | 6,10 | -0,94 | 0,064095 | Lsm10 |
| ENSRNOG00000009963 | 272,03 | -0,37 | 0,06411 | Ctps1 |
| ENSRNOG00000004912 | 117,51 | 0,56 | 0,06411 | Itgav |
| ENSRNOG00000014613 | 87,52 | 0,62 | 0,06411 | Ddah1 |
| ENSRNOG00000015077 | 62,28 | -0,71 | 0,064163 | Acsf3 |
| ENSRNOG00000005938 | 82,93 | -0,67 | 0,064386 |  |
| ENSRNOG00000019387 | 83,01 | 0,47 | 0,064386 | Ifi30 |
| ENSRNOG00000001515 | 189,16 | 0,54 | 0,064473 | Zak |
| ENSRNOG00000013057 | 17,52 | 0,93 | 0,064473 | Prc1 |
| ENSRNOG00000017536 | 38,73 | -0,80 | 0,064473 | Cdc16 |
| ENSRNOG00000027914 | 53,91 | -0,69 | 0,064602 | Plscr3 |
| ENSRNOG00000015896 | 134,82 | -0,46 | 0,064745 | Rbpms2 |
| ENSRNOG00000017189 | 139,96 | -0,62 | 0,064751 |  |
| ENSRNOG00000011853 | 200,02 | 0,41 | 0,064896 | Mbd2 |
| ENSRNOG00000002693 | 20,99 | 0,82 | 0,06503 | Nme1 |
| ENSRNOG00000013356 | 175,13 | 0,40 | 0,065119 | Snapin |
| ENSRNOG00000012630 | 209,84 | 0,60 | 0,065257 | Rhoc |
| ENSRNOG00000007405 | 14,59 | -0,92 | 0,06526 | Krba1 |
| ENSRNOG00000001335 | 39,02 | 0,83 | 0,065341 | Zkscan1 |
| ENSRNOG00000019383 | 287,87 | -0,57 | 0,06578 | Tef |
| ENSRNOG00000018227 | 68,39 | 0,72 | 0,065798 | Mfsd14b |
| ENSRNOG00000034198 | 150,58 | 0,47 | 0,066006 | Tceal9 |
| ENSRNOG00000002625 | 61,12 | 0,73 | 0,066006 | Ptpn4 |
| ENSRNOG00000031827 | 182,15 | -0,42 | 0,066379 | Arih2 |
| ENSRNOG00000019536 | 113,11 | -0,62 | 0,066431 | Smim3 |
| ENSRNOG00000049507 | 13,54 | 0,93 | 0,066903 | Sept10 |
| ENSRNOG00000007439 | 8,74 | -0,93 | 0,066903 | Unc13d |
| ENSRNOG00000013718 | 425,08 | 0,30 | 0,067006 | Herc2 |
| ENSRNOG00000000867 | 218,29 | -0,49 | 0,067041 | Vars |
| ENSRNOG00000016256 | 122,83 | 0,52 | 0,067048 | Myo9b |
| ENSRNOG00000028227 | 104,60 | 0,62 | 0,067351 | Pbrm1 |
| ENSRNOG00000020269 | 112,62 | -0,54 | 0,067351 | Sugp2 |
| ENSRNOG00000036658 | 57,08 | -0,66 | 0,067412 | Tbcd |
| ENSRNOG00000006995 | 168,81 | 0,49 | 0,067576 | Ano6 |
| ENSRNOG00000016475 | 35,00 | 0,81 | 0,067576 | Nt5c3b |
| ENSRNOG00000026564 | 10,54 | 0,92 | 0,067594 | Ptpdc1 |
| ENSRNOG00000024450 | 22,36 | 0,92 | 0,067599 | Poc1b |
| ENSRNOG00000015106 | 7,11 | 0,84 | 0,0677 |  |
| ENSRNOG00000018468 | 66,99 | -0,66 | 0,06782 | Ldb1 |
| ENSRNOG00000042821 | 672,42 | 0,32 | 0,06783 | Cd59 |
| ENSRNOG00000004517 | 47,48 | 0,65 | 0,067943 | Igf1 |
| ENSRNOG00000013791 | 51,84 | 0,77 | 0,067943 | Enpp3 |
| ENSRNOG00000009598 | 62,01 | -0,62 | 0,068227 | Ncaph2 |
| ENSRNOG00000028872 | 99,53 | 0,66 | 0,068227 | Rai14 |
| ENSRNOG00000015128 | 125,97 | 0,45 | 0,068644 | Sae1 |
| ENSRNOG00000009360 | 11,92 | 0,93 | 0,06871 | Sh3bp1 |
| ENSRNOG00000025625 | 268,97 | 0,52 | 0,068796 | Rnase4 |
| ENSRNOG00000028358 | 67,02 | 0,66 | 0,068796 | Edem3 |
| ENSRNOG00000011877 | 11,17 | 0,92 | 0,068823 |  |
| ENSRNOG00000010642 | 69,41 | 0,59 | 0,06922 | Lysmd2 |
| ENSRNOG00000004132 | 146,86 | 0,48 | 0,06922 | Lasp1 |
| ENSRNOG00000024111 | 6,71 | 0,89 | 0,06922 | Cage1 |
| ENSRNOG00000011346 | 275,70 | 0,47 | 0,06922 | Ehd2 |
| ENSRNOG00000007323 | 28,01 | -0,80 | 0,06922 | Ric8b |
| ENSRNOG00000022331 | 8,60 | -0,93 | 0,069462 | Ccdc78 |
| ENSRNOG00000050044 | 507,71 | 0,34 | 0,069462 | Ptp4a2 |
| ENSRNOG00000011316 | 21,69 | 0,92 | 0,069752 | Fam167a |
| ENSRNOG00000018459 | 9,08 | 0,90 | 0,069829 |  |
| ENSRNOG00000001059 | 35,92 | 0,66 | 0,07008 | Usp42 |
| ENSRNOG00000013809 | 117,42 | 0,52 | 0,07008 | Kbtbd2 |
| ENSRNOG00000017607 | 46,21 | 0,71 | 0,07008 | Faf2 |
| ENSRNOG00000001344 | 1407,39 | -0,27 | 0,070311 | Aldh2 |
| ENSRNOG00000011216 | 219,43 | 0,40 | 0,070311 | Tbl1xr1 |
| ENSRNOG00000020748 | 1921,53 | 0,20 | 0,070406 | Map4 |
| ENSRNOG00000004873 | 128,00 | 0,48 | 0,070569 | Prkch |
| ENSRNOG00000022772 | 85,94 | 0,58 | 0,070813 | Prickle1 |
| ENSRNOG00000021086 | 10,30 | 0,92 | 0,070869 | Dtx4 |
| ENSRNOG00000001789 | 90,07 | 0,49 | 0,070973 | Zfp148 |
| ENSRNOG00000000728 | 176,59 | 0,50 | 0,070985 | Clic2 |
| ENSRNOG00000019290 | 38,71 | -0,71 | 0,071024 | Pskh1 |
| ENSRNOG00000012786 | 83,24 | 0,49 | 0,071111 | Pgrmc1 |
| ENSRNOG00000046345 | 328,35 | 0,37 | 0,071476 | Rpn1 |
| ENSRNOG00000029879 | 70,76 | -0,61 | 0,071476 |  |
| ENSRNOG00000020836 | 57,68 | -0,65 | 0,071476 | Rorc |
| ENSRNOG00000019491 | 19,93 | -0,87 | 0,07229 | Stard10 |
| ENSRNOG00000037505 | 179,53 | -0,42 | 0,072309 | Ulk1 |
| ENSRNOG00000002467 | 1486,59 | -0,29 | 0,072583 |  |
| ENSRNOG00000020169 | 58,77 | 0,68 | 0,073322 | Gimap8 |
| ENSRNOG00000021441 | 31,16 | 0,88 | 0,073322 | Reln |
| ENSRNOG00000010224 | 97,93 | 0,61 | 0,073359 | Rab30 |
| ENSRNOG00000000824 | 37,81 | 0,75 | 0,073614 | Dse |
| ENSRNOG00000020393 | 80,35 | 0,56 | 0,073614 | Rhog |
| ENSRNOG00000022946 | 39,27 | -0,81 | 0,07364 | Slc22a3 |
| ENSRNOG00000008564 | 20,33 | -0,89 | 0,07364 | Tmem222 |
| ENSRNOG00000007981 | 132,21 | -0,45 | 0,073676 | Klhl23 |
| ENSRNOG00000008620 | 27,81 | 0,78 | 0,073773 | Smad3 |
| ENSRNOG00000012568 | 17,13 | 0,86 | 0,073774 | Madd |
| ENSRNOG00000017863 | 239,40 | 0,41 | 0,073962 | Zeb1 |
| ENSRNOG00000010997 | 86,61 | 0,52 | 0,073962 | Ednrb |
| ENSRNOG00000003268 | 58,61 | 0,62 | 0,073962 | Maml1 |
| ENSRNOG00000001547 | 31,84 | 0,79 | 0,074512 | Agps |
| ENSRNOG00000008144 | 79,78 | 0,63 | 0,074557 | Irf1 |
| ENSRNOG00000002208 | 478,55 | -0,43 | 0,074557 | Shroom3 |
| ENSRNOG00000012280 | 7,11 | 0,80 | 0,074557 | Ptx3 |
| ENSRNOG00000000304 | 584,38 | 0,25 | 0,075015 | Cd164 |
| ENSRNOG00000010750 | 30,59 | 0,78 | 0,075341 | Twistnb |
| ENSRNOG00000004524 | 40,37 | 0,62 | 0,076275 | Desi2 |
| ENSRNOG00000049511 | 72,95 | -0,79 | 0,076466 |  |
| ENSRNOG00000015417 | 265,93 | -0,44 | 0,076509 | Kansl3 |
| ENSRNOG00000019482 | 47,79 | 0,71 | 0,076509 | Gnao1 |
| ENSRNOG00000048057 | 1358,99 | 0,37 | 0,076817 |  |
| ENSRNOG00000016588 | 487,63 | 0,31 | 0,076859 |  |
| ENSRNOG00000010993 | 92,81 | 0,50 | 0,076859 | Dpm1 |
| ENSRNOG00000014084 | 163,91 | 0,50 | 0,076863 | Sp1 |
| ENSRNOG00000036688 | 487,67 | 0,30 | 0,076898 | Arhgdia |
| ENSRNOG00000006178 | 1152,78 | 0,24 | 0,077069 | Dync1h1 |
| ENSRNOG00000014744 | 53,88 | 0,62 | 0,077149 | Pacs2 |
| ENSRNOG00000020994 | 266,14 | -0,38 | 0,077719 | Slc25a39 |
| ENSRNOG00000001890 | 136,42 | -0,55 | 0,077719 | Txnrd2 |
| ENSRNOG00000010765 | 947,51 | -0,29 | 0,078102 | Vcl |
| ENSRNOG00000015249 | 34,49 | -0,77 | 0,078501 | Taf8 |
| ENSRNOG00000018715 | 78,12 | 0,55 | 0,07866 | Clec10a |
| ENSRNOG00000020300 | 58,97 | 0,65 | 0,079154 | Lsp1 |
| ENSRNOG00000011491 | 155,07 | 0,41 | 0,079154 | Dnajc13 |
| ENSRNOG00000014361 | 9,16 | 0,88 | 0,079208 | Edn1 |
| ENSRNOG00000017889 | 9,80 | -0,90 | 0,079435 |  |
| ENSRNOG00000002643 | 34,93 | 0,85 | 0,079435 | Ugdh |
| ENSRNOG00000042189 | 179,69 | 0,42 | 0,07955 | Rab31 |
| ENSRNOG00000021478 | 149,35 | -0,58 | 0,079645 | Tpd52l1 |
| ENSRNOG00000006146 | 893,28 | -0,45 | 0,079803 | Trim54 |
| ENSRNOG00000010895 | 139,02 | 0,61 | 0,080025 | Tmem30a |
| ENSRNOG00000016589 | 365,63 | -0,35 | 0,080053 | Mlf2 |
| ENSRNOG00000038883 | 540,24 | -0,26 | 0,08016 | LOC294154 |
| ENSRNOG00000000137 | 9,30 | 0,88 | 0,080225 | Ly86 |
| ENSRNOG00000014013 | 345,44 | 0,36 | 0,080302 | Map4k4 |
| ENSRNOG00000016952 | 606,12 | -0,36 | 0,080433 | Uqcr11 |
| ENSRNOG00000025970 | 4,69 | -0,89 | 0,080433 |  |
| ENSRNOG00000001062 | 79,55 | 0,56 | 0,080582 | Kmt5a |
| ENSRNOG00000024345 | 71,38 | 0,56 | 0,08086 | Pard3b |
| ENSRNOG00000012227 | 16,02 | -0,89 | 0,08086 | Stambp |
| ENSRNOG00000043031 | 114,83 | -0,65 | 0,081254 | Rd3l |
| ENSRNOG00000016033 | 59,66 | -0,59 | 0,081254 | Endog |
| ENSRNOG00000027408 | 80,81 | -0,55 | 0,081277 | Ppid |
| ENSRNOG00000005357 | 57,04 | -0,59 | 0,081495 | Ctc1 |
| ENSRNOG00000011815 | 586,06 | 0,47 | 0,081541 | Sgk1 |
| ENSRNOG00000009535 | 148,94 | -0,58 | 0,081713 | Stoml2 |
| ENSRNOG00000001058 | 268,93 | -0,49 | 0,081753 | Timm44 |
| ENSRNOG00000001161 | 100,29 | -0,50 | 0,081947 | Gatc |
| ENSRNOG00000011849 | 2688,83 | -0,27 | 0,082116 | Ndufs1 |
| ENSRNOG00000021567 | 254,15 | 0,44 | 0,082116 |  |
| ENSRNOG00000004060 | 1137,46 | 0,24 | 0,082661 | Calm1 |
| ENSRNOG00000017882 | 7,28 | 0,86 | 0,082818 | Camk1d |
| ENSRNOG00000009449 | 187,41 | -0,56 | 0,082977 | Trim35 |
| ENSRNOG00000016207 | 283,18 | 0,35 | 0,083051 | Galnt1 |
| ENSRNOG00000000147 | 363,49 | -0,37 | 0,083105 |  |
| ENSRNOG00000015514 | 20,18 | 0,88 | 0,083117 | Bcat1 |
| ENSRNOG00000006532 | 251,70 | 0,38 | 0,083121 | H3f3b |
| ENSRNOG00000004837 | 191,41 | -0,57 | 0,083187 | Limd1 |
| ENSRNOG00000016907 | 158,70 | -0,41 | 0,083397 | Ppp5c |
| ENSRNOG00000000127 | 89,17 | 0,42 | 0,084036 | Kpna6 |
| ENSRNOG00000018395 | 46,34 | -0,64 | 0,084189 | Usp40 |
| ENSRNOG00000025792 | 448,65 | 0,27 | 0,084257 | Crk |
| ENSRNOG00000009099 | 27,96 | -0,80 | 0,084262 |  |
| ENSRNOG00000017441 | 170,75 | 0,47 | 0,0843 | Tpm3 |
| ENSRNOG00000000906 | 20,82 | 0,88 | 0,08446 | Medag |
| ENSRNOG00000020235 | 347,17 | -0,30 | 0,08457 | Hnrnpl |
| ENSRNOG00000050145 | 4549,57 | 0,30 | 0,084608 |  |
| ENSRNOG00000018677 | 175,61 | -0,40 | 0,084658 | Akt2 |
| ENSRNOG00000011901 | 185,55 | -0,61 | 0,084658 | Rrad |
| ENSRNOG00000038622 | 15,72 | -0,89 | 0,084658 |  |
| ENSRNOG00000004556 | 224,66 | 0,43 | 0,084814 | Dcaf5 |
| ENSRNOG00000020812 | 342,80 | -0,45 | 0,084814 | Gys1 |
| ENSRNOG00000000795 | 29,47 | 0,73 | 0,085121 | RT1-N3 |
| ENSRNOG00000014722 | 129,56 | 0,58 | 0,085229 | Raph1 |
| ENSRNOG00000001098 | 183,24 | 0,40 | 0,08528 | Pds5b |
| ENSRNOG00000018176 | 378,52 | 0,31 | 0,08528 | Rab6a |
| ENSRNOG00000000451 | 88,05 | 0,65 | 0,085872 | RT1-Ba |
| ENSRNOG00000011830 | 89,90 | 0,49 | 0,085872 | Plekha3 |
| ENSRNOG00000012820 | 203,68 | 0,42 | 0,085872 | Add3 |
| ENSRNOG00000011648 | 1289,82 | 0,37 | 0,085872 | Aqp1 |
| ENSRNOG00000013463 | 35,75 | 0,73 | 0,085872 | Kcnj8 |
| ENSRNOG00000008989 | 55,30 | -0,62 | 0,085872 | Cryl1 |
| ENSRNOG00000046834 | 14,93 | 0,86 | 0,085872 | C3 |
| ENSRNOG00000023209 | 6,28 | 0,86 | 0,085872 | Slamf7 |
| ENSRNOG00000008106 | 62,88 | -0,56 | 0,085872 | Shmt2 |
| ENSRNOG00000004249 | 20,78 | 0,85 | 0,085872 | Tlr7 |
| ENSRNOG00000019506 | 264,98 | -0,44 | 0,085973 | Dnajb2 |
| ENSRNOG00000005710 | 37,37 | -0,63 | 0,08598 | Rbm7 |
| ENSRNOG00000011879 | 348,24 | 0,49 | 0,086261 | Nfat5 |
| ENSRNOG00000036693 | 26,66 | -0,71 | 0,086363 | Slc25a10 |
| ENSRNOG00000012826 | 80,73 | 0,56 | 0,086613 | Creb3l2 |
| ENSRNOG00000022043 | 14,61 | -0,87 | 0,086724 | Yipf2 |
| ENSRNOG00000005094 | 27,54 | 0,78 | 0,086724 | C1qtnf7 |
| ENSRNOG00000016451 | 86,73 | -0,57 | 0,086724 | Cd1d1 |
| ENSRNOG00000000175 | 10,73 | -0,89 | 0,086785 | Mier2 |
| ENSRNOG00000018765 | 13,86 | -0,85 | 0,086821 | Pold4 |
| ENSRNOG00000008393 | 579,11 | 0,35 | 0,087172 | Tax1bp1 |
| ENSRNOG00000005569 | 267,52 | -0,42 | 0,087172 | Phospho1 |
| ENSRNOG00000011680 | 22,34 | 0,81 | 0,087172 | Il16 |
| ENSRNOG00000003706 | 75,07 | -0,52 | 0,087172 | Ufc1 |
| ENSRNOG00000015633 | 528,78 | 0,32 | 0,087172 | Cul3 |
| ENSRNOG00000019840 | 19,10 | -0,87 | 0,087398 | Mdp1 |
| ENSRNOG00000050655 | 181,24 | 0,64 | 0,087479 | P4ha1 |
| ENSRNOG00000016684 | 497,33 | -0,37 | 0,087479 | Wnk2 |
| ENSRNOG00000001442 | 448,47 | -0,36 | 0,087531 | Por |
| ENSRNOG00000013097 | 38,64 | -0,79 | 0,087549 | LOC691485 |
| ENSRNOG00000027320 | 26,35 | 0,73 | 0,087703 |  |
| ENSRNOG00000027136 | 68,30 | 0,57 | 0,087703 | Spast |
| ENSRNOG00000019213 | 552,93 | -0,40 | 0,087721 |  |
| ENSRNOG00000018656 | 8,80 | -0,88 | 0,087721 | Ampd1 |
| ENSRNOG00000019730 | 291,89 | -0,47 | 0,087946 | Inppl1 |
| ENSRNOG00000018162 | 5,85 | -0,89 | 0,088039 | Nhej1 |
| ENSRNOG00000007887 | 183,03 | 0,45 | 0,088447 | Elk4 |
| ENSRNOG00000017226 | 581,16 | -0,33 | 0,088502 | Slc2a4 |
| ENSRNOG00000014029 | 49,51 | -0,68 | 0,088502 | Klhl13 |
| ENSRNOG00000016294 | 57,07 | 0,77 | 0,088772 | Cd4 |
| ENSRNOG00000005513 | 152,71 | -0,63 | 0,088789 | Srsf5 |
| ENSRNOG00000045649 | 40,96 | 0,66 | 0,088962 | Arrdc3 |
| ENSRNOG00000011596 | 171,07 | 0,51 | 0,088962 |  |
| ENSRNOG00000010777 | 22,22 | -0,82 | 0,089028 | Tox |
| ENSRNOG00000011976 | 79,22 | -0,53 | 0,089068 | Nudt7 |
| ENSRNOG00000024705 | 46,59 | 0,61 | 0,089224 | Rarres2 |
| ENSRNOG00000014832 | 77,01 | -0,62 | 0,089666 | Mapkapk3 |
| ENSRNOG00000011587 | 406,12 | -0,46 | 0,08998 |  |
| ENSRNOG00000019996 | 1527,53 | -0,28 | 0,08998 | Slc16a1 |
| ENSRNOG00000009867 | 90,99 | 0,56 | 0,090101 | Tgfb3 |
| ENSRNOG00000007442 | 129,78 | -0,53 | 0,090413 | Ubl7 |
| ENSRNOG00000017108 | 8,50 | -0,86 | 0,090413 | Syngr1 |
| ENSRNOG00000004152 | 23,42 | 0,81 | 0,090462 | Lrp12 |
| ENSRNOG00000021224 | 49,91 | -0,65 | 0,090462 | Mrps26 |
| ENSRNOG00000011994 | 187,53 | -0,53 | 0,090915 | Perp |
| ENSRNOG00000008063 | 734,59 | -0,36 | 0,091104 | Hibadh |
| ENSRNOG00000014234 | 53,57 | 0,69 | 0,091219 | Hif1an |
| ENSRNOG00000022533 | 25,61 | 0,74 | 0,091381 | Micall2 |
| ENSRNOG00000017977 | 297,12 | -0,36 | 0,091381 | Smpd1 |
| ENSRNOG00000000479 | 7,19 | 0,83 | 0,091542 | Kifc1 |
| ENSRNOG00000016875 | 54,28 | -0,61 | 0,091613 | Cbx7 |
| ENSRNOG00000001316 | 681,30 | -0,31 | 0,091693 | Anapc5 |
| ENSRNOG00000018516 | 72,99 | -0,55 | 0,091964 | Impa2 |
| ENSRNOG00000029610 | 10,50 | -0,88 | 0,092234 |  |
| ENSRNOG00000005177 | 155,88 | -0,49 | 0,092234 | Tp53i3 |
| ENSRNOG00000007047 | 43,21 | 0,76 | 0,092234 | Eps8 |
| ENSRNOG00000002403 | 39,64 | 0,74 | 0,092234 | Fam129a |
| ENSRNOG00000007023 | 28,88 | -0,81 | 0,092405 | Galm |
| ENSRNOG00000001964 | 63,03 | 0,50 | 0,092653 | Cd47 |
| ENSRNOG00000008165 | 11,36 | 0,88 | 0,092977 | Tpx2 |
| ENSRNOG00000017660 | 68,12 | 0,52 | 0,093591 | Inpp4a |
| ENSRNOG00000005877 | 571,10 | -0,27 | 0,093591 | Lrpprc |
| ENSRNOG00000036816 | 190,53 | -0,36 | 0,09364 | Wls |
| ENSRNOG00000006103 | 29,83 | 0,83 | 0,093761 | Tbc1d31 |
| ENSRNOG00000017260 | 51,04 | 0,53 | 0,093806 | Cdr2 |
| ENSRNOG00000030364 | 8,54 | -0,86 | 0,093828 |  |
| ENSRNOG00000005041 | 3017,93 | -0,28 | 0,093859 | Crip2 |
| ENSRNOG00000000142 | 45,54 | 0,63 | 0,093859 | Plxdc2 |
| ENSRNOG00000015194 | 9,55 | 0,86 | 0,094221 |  |
| ENSRNOG00000015231 | 79,18 | -0,52 | 0,09443 | Mrpl44 |
| ENSRNOG00000014957 | 42,66 | 0,61 | 0,09443 |  |
| ENSRNOG00000008981 | 323,32 | 0,32 | 0,094642 | Pdcd6ip |
| ENSRNOG00000005302 | 15,63 | 0,87 | 0,094755 | Slc2a9 |
| ENSRNOG00000022540 | 44,12 | -0,61 | 0,094755 | Trex1 |
| ENSRNOG00000015643 | 12,86 | 0,86 | 0,09479 |  |
| ENSRNOG00000017564 | 113,96 | -0,55 | 0,094927 | Mib2 |
| ENSRNOG00000003583 | 296,13 | -0,45 | 0,095072 | Smyd2 |
| ENSRNOG00000039300 | 24,96 | 0,86 | 0,095091 | Ahcyl2 |
| ENSRNOG00000002959 | 49,77 | 0,63 | 0,095091 | Shroom4 |
| ENSRNOG00000008195 | 355,38 | 0,34 | 0,095119 | Ywhaz |
| ENSRNOG00000026493 | 31,24 | -0,75 | 0,095127 | Cdnf |
| ENSRNOG00000021155 | 28,25 | 0,78 | 0,09519 | Ctsk |
| ENSRNOG00000006756 | 174,42 | -0,44 | 0,095519 | Maged1 |
| ENSRNOG00000018566 | 706,20 | 0,39 | 0,095519 | Ctsl |
| ENSRNOG00000000470 | 115,23 | -0,60 | 0,095519 | Vps52 |
| ENSRNOG00000008632 | 15,74 | 0,86 | 0,095629 | Invs |
| ENSRNOG00000032834 | 46,32 | 0,66 | 0,095907 |  |
| ENSRNOG00000012563 | 155,36 | 0,43 | 0,095964 | Arhgap29 |
| ENSRNOG00000014258 | 14,33 | 0,84 | 0,09606 | Rab32 |
| ENSRNOG00000004526 | 290,09 | -0,50 | 0,096274 | Cox7a2l |
| ENSRNOG00000032978 | 29,63 | 0,84 | 0,096679 |  |
| ENSRNOG00000020597 | 7,73 | -0,87 | 0,096771 | RGD1305014 |
| ENSRNOG00000012386 | 117,99 | 0,46 | 0,097282 | Zbtb38 |
| ENSRNOG00000001355 | 71,37 | -0,57 | 0,097444 | Taf6 |
| ENSRNOG00000017212 | 33,59 | 0,76 | 0,097444 | Spsb1 |
| ENSRNOG00000003330 | 349,10 | -0,47 | 0,097609 | Acsf2 |
| ENSRNOG00000011058 | 482,91 | 0,32 | 0,097636 | Utrn |
| ENSRNOG00000029598 | 7,62 | 0,81 | 0,097636 | Robo2 |
| ENSRNOG00000020803 | 12,39 | 0,87 | 0,097636 | Meox1 |
| ENSRNOG00000023127 | 72,64 | -0,58 | 0,097825 |  |
| ENSRNOG00000009681 | 202,94 | -0,43 | 0,098115 | Flot2 |
| ENSRNOG00000039858 | 7,50 | -0,86 | 0,098121 | Mfsd12 |
| ENSRNOG00000021120 | 38,99 | -0,75 | 0,098139 | Prune |
| ENSRNOG00000002839 | 22,58 | -0,82 | 0,098418 | Slc19a2 |
| ENSRNOG00000002721 | 66,06 | -0,71 | 0,098671 | Ndufb4 |
| ENSRNOG00000018198 | 100,32 | 0,56 | 0,098808 | Dapk1 |
| ENSRNOG00000003954 | 84,25 | 0,54 | 0,098813 | Il2rg |
| ENSRNOG00000029885 | 294,28 | 0,45 | 0,098862 | Stag2 |
| ENSRNOG00000024159 | 75,68 | 0,59 | 0,099058 | Fcer1g |
| ENSRNOG00000036691 | 47,36 | -0,71 | 0,099058 | Mcrip1 |
| ENSRNOG00000014128 | 404,97 | -0,39 | 0,099058 | Ecsit |
| ENSRNOG00000014064 | 405,61 | 0,36 | 0,099058 | Ctsh |
| ENSRNOG00000015840 | 531,95 | -0,27 | 0,099192 | Hsd17b4 |
| ENSRNOG00000022343 | 42,09 | 0,63 | 0,099196 | Alms1 |
| ENSRNOG00000042195 | 141,29 | -0,46 | 0,099301 | Pabpn1 |
| ENSRNOG00000012603 | 100,96 | 0,50 | 0,099498 | Sestd1 |
| ENSRNOG00000049075 | 102,43 | 0,58 | 0,099498 | Fabp5 |
| ENSRNOG00000007128 | 50,27 | -0,62 | 0,099993 | Nop56 |
| ENSRNOG00000019497 | 205,73 | -0,41 | 0,099993 | Mrpl17 |

**Supplemental table 6:** Predicted activation of transcription factors in obese versus lean

| **Upstream Regulator** | **Expr Log Ratio** | **Predicted State** | **Activation z-score** | **p-value of overlap** |
| --- | --- | --- | --- | --- |
| KLF15 |  | Activated | 2,950 | 2,66E-13 |
| PPARGC1A |  |  | 0,457 | 1,05E-12 |
| TFAM |  |  | 1,414 | 6,94E-10 |
| MEF2C |  |  | 1,685 | 1,98E-08 |
| MYC |  |  | 0,834 | 1,44E-07 |
| TP53 |  | Activated | 2,805 | 3,94E-07 |
| Esrra |  |  | -0,447 | 2,18E-06 |
| HDAC5 |  |  |  | 5,25E-06 |
| NUPR1 |  |  | 0,000 | 7,15E-06 |
| HDAC4 |  |  | -1,067 | 1,07E-05 |
| MYOCD |  |  | 1,674 | 1,59E-05 |
| TBX5 |  |  | 1,976 | 1,60E-05 |
| SREBF1 |  |  | -0,992 | 3,04E-05 |
| CEBPA |  |  | 0,529 | 3,29E-05 |
| PPARGC1B |  |  | 1,294 | 4,46E-05 |
| Foxp1 |  | Inhibited | -2,236 | 7,34E-05 |
| KLF11 |  |  | 1,109 | 9,60E-05 |
| SERTAD2 |  |  | -1,982 | 9,76E-05 |
| SP1 |  |  | 0,085 | 1,02E-04 |
| HAND2 |  |  | 1,292 | 1,77E-04 |
| EPAS1 |  |  | 1,084 | 2,09E-04 |
| CLOCK |  |  |  | 2,45E-04 |
| HIF1A |  |  | -1,008 | 2,60E-04 |
| MLIP |  |  |  | 2,90E-04 |
| HNF4A |  |  | -0,696 | 3,17E-04 |
| KLF2 |  |  | 0,116 | 3,20E-04 |
| MYCN |  |  | -1,134 | 3,70E-04 |
| VHL |  |  | -0,585 | 4,43E-04 |
| FOS |  |  | 0,547 | 4,86E-04 |
| MEF2D |  |  |  | 6,47E-04 |
| CALR |  |  | -1,060 | 7,08E-04 |
| ELK3 |  |  |  | 7,31E-04 |
| RBPJ |  |  | 1,134 | 8,31E-04 |
| THAP11 |  |  |  | 8,59E-04 |
| PDX1 |  |  | -0,378 | 8,70E-04 |
| EP300 | 0,605 |  | 0,790 | 1,38E-03 |
| ATF4 | 0,431 |  | 1,238 | 1,39E-03 |
| PPRC1 |  |  | -0,447 | 1,58E-03 |
| SREBF2 |  |  | 0,749 | 1,64E-03 |
| KDM5A |  |  | -0,707 | 1,76E-03 |
| HEY2 |  |  | -0,919 | 1,81E-03 |
| NFATC4 |  |  |  | 1,81E-03 |
| MEF2A |  |  | -0,254 | 1,81E-03 |
| HDAC9 |  |  |  | 1,92E-03 |
| PLAGL1 |  |  |  | 1,92E-03 |
| PML |  |  | -1,455 | 2,04E-03 |
| SMAD3 |  |  | -1,048 | 2,36E-03 |
| SOX17 |  |  |  | 2,57E-03 |
| RFXANK |  |  |  | 2,80E-03 |
| KMT2D |  |  | 1,667 | 3,06E-03 |
| FOXO3 |  |  | 0,380 | 3,27E-03 |
| GLIS2 |  |  |  | 3,31E-03 |
| GATA4 | 0,395 |  | 0,834 | 3,70E-03 |
| NRIP1 |  |  | -1,964 | 3,81E-03 |
| NKX2-1 |  |  |  | 3,83E-03 |
| NFKBIA |  |  | -1,399 | 4,01E-03 |
| ATF6 |  |  | 0,283 | 4,38E-03 |
| PPP1R13L | 0,558 |  |  | 4,51E-03 |
| FOXO4 |  |  |  | 5,17E-03 |
| TP73 |  |  | 0,498 | 5,18E-03 |
| STAT4 |  | Activated | 2,594 | 5,26E-03 |
| MBD2 |  |  |  | 5,34E-03 |
| MYOD1 |  |  | -1,491 | 5,44E-03 |
| ASXL1 |  |  | 1,000 | 5,57E-03 |
| CEBPB |  |  | 0,044 | 5,68E-03 |
| NFE2L2 |  |  | 1,004 | 5,68E-03 |
| KDM3A |  |  |  | 5,75E-03 |
| H2AFX |  |  |  | 5,94E-03 |
| HEY1 |  |  |  | 5,94E-03 |
| SRSF2 |  |  |  | 6,06E-03 |
| SMAD4 |  |  | 0,831 | 6,49E-03 |
| SKI |  |  |  | 6,75E-03 |
| EBF1 |  |  | -0,152 | 7,25E-03 |
| HEYL |  |  |  | 7,58E-03 |
| FOXO1 |  |  | 0,414 | 7,60E-03 |
| HEXIM1 |  |  |  | 8,55E-03 |
| NCOA3 |  |  | -0,262 | 9,62E-03 |
| MKX |  |  |  | 9,63E-03 |
| NOTCH4 |  |  |  | 1,06E-02 |
| JUN |  |  | 0,081 | 1,16E-02 |
| HOXA10 |  |  | 0,707 | 1,19E-02 |
| STAT6 |  |  | -0,040 | 1,28E-02 |
| NFYA |  |  |  | 1,37E-02 |
| PLAG1 |  |  | 0,132 | 1,43E-02 |
| FOXC2 |  |  | 0,000 | 1,52E-02 |
| CITED2 |  |  |  | 1,55E-02 |
| FEV |  |  |  | 1,58E-02 |
| RUNX2 |  |  | 0,391 | 1,70E-02 |
| ASB9 |  |  |  | 1,70E-02 |
| SLC2A4RG |  |  |  | 1,70E-02 |
| PHF5A |  |  |  | 1,70E-02 |
| RBM39 |  |  |  | 1,70E-02 |
| MED4 |  |  |  | 1,70E-02 |
| GATA5 |  |  |  | 1,71E-02 |
| BHLHE41 |  |  |  | 1,71E-02 |
| CREBBP |  |  | -0,555 | 1,73E-02 |
| MAX |  |  | -1,342 | 1,73E-02 |
| NFATC3 |  |  |  | 1,81E-02 |
| ARNT |  |  | -0,218 | 1,82E-02 |
| HTT |  |  |  | 1,90E-02 |
| RUNX3 |  |  | -0,128 | 1,96E-02 |
| NCOR1 |  |  |  | 1,97E-02 |
| ZIC3 |  |  |  | 1,99E-02 |
| TEAD4 |  |  |  | 2,15E-02 |
| NANOG |  |  | -0,217 | 2,25E-02 |
| GLI1 |  |  | -0,194 | 2,35E-02 |
| SIRT1 |  |  | 0,143 | 2,42E-02 |
| MTDH |  |  |  | 2,49E-02 |
| ARNT2 |  |  | 0,707 | 2,60E-02 |
| THRAP3 |  |  |  | 2,63E-02 |
| TBX3 |  |  |  | 2,63E-02 |
| RCOR1 |  |  |  | 2,63E-02 |
| TCF3 |  |  |  | 2,78E-02 |
| TFAP4 |  |  |  | 2,85E-02 |
| MEOX2 |  |  |  | 2,85E-02 |
| ZFPM2 |  |  |  | 2,97E-02 |
| SMAD7 |  |  | 0,869 | 3,01E-02 |
| NKX2-5 |  |  |  | 3,05E-02 |
| BRCA1 |  |  | -1,709 | 3,16E-02 |
| KLF4 |  |  | 0,500 | 3,20E-02 |
| SP3 |  |  | 0,342 | 3,23E-02 |
| MXI1 |  |  |  | 3,33E-02 |
| SMAD6 | 0,525 |  |  | 3,33E-02 |
| MLXIP |  |  |  | 3,38E-02 |
| HLTF | 0,556 |  |  | 3,38E-02 |
| EDF1 |  |  |  | 3,38E-02 |
| SAP30 |  |  |  | 3,38E-02 |
| ING2 |  |  |  | 3,38E-02 |
| JARID2 |  |  |  | 3,70E-02 |
| RRP1B |  |  |  | 4,02E-02 |
| CTNNB1 |  |  | 1,911 | 4,07E-02 |
| GATA6 | 0,363 |  | 1,194 | 4,26E-02 |
| E2F1 |  |  | -0,788 | 4,36E-02 |
| SQSTM1 |  |  |  | 4,59E-02 |
| EGR2 |  |  | 0,447 | 4,62E-02 |
| FOXA1 |  |  | 0,254 | 4,74E-02 |
| FOXA2 |  |  | -0,130 | 4,79E-02 |
| SIN3B |  |  |  | 4,91E-02 |
| USF1 |  |  |  | 4,96E-02 |

Transcription factors are ordered by differentially expressed target genes (p-value of overlap). Expr Log Ratio = differential expression of transcription factor itself, Predicted State = activated or inhibited in obese rats based on Activation z-scores of >2 or <-2, respectively, Activation z-score = quantitative determination of activity based on expected and observed direction of differential target gene expression.

**Supplemental table 7:** Predicted activation of transcription factors in lean + DS versus lean

| **Upstream Regulator** | **Expr Log Ratio** | **Predicted State** | **Activation z-score** | **p-value of overlap** |
| --- | --- | --- | --- | --- |
| KLF15 |  | Inhibited | -3,965 | 5,37E-14 |
| PPARGC1A | 0,666 | Inhibited | -4,752 | 9,97E-14 |
| TP53 |  |  | 1,951 | 1,58E-11 |
| MYC |  |  | -0,182 | 5,14E-11 |
| HDAC5 |  |  | -0,707 | 1,27E-10 |
| EP300 | 1,258 |  | 1,410 | 6,30E-08 |
| TBX5 |  |  | -1,110 | 8,99E-08 |
| HTT |  |  | 1,667 | 1,01E-07 |
| KDM5A |  | Activated | 4,264 | 3,74E-07 |
| CREBBP | 0,500 |  | 0,183 | 7,71E-07 |
| NFATC4 |  |  | 1,109 | 1,39E-06 |
| MEF2A |  |  | 0,216 | 1,39E-06 |
| MEF2C | 0,898 |  | -0,978 | 1,84E-06 |
| HAND2 |  |  | -1,087 | 3,26E-06 |
| Esrra | -0,641 |  | 0,098 | 4,60E-06 |
| GATA4 |  |  | -0,716 | 8,15E-06 |
| SP1 |  | Activated | 2,145 | 9,18E-06 |
| RB1 | 0,490 | Inhibited | -3,664 | 9,57E-06 |
| SMAD7 | -0,872 | Inhibited | -3,346 | 1,28E-05 |
| SMAD3 |  | Activated | 2,878 | 1,35E-05 |
| GLIS2 |  | Inhibited | -2,646 | 1,36E-05 |
| PPARGC1B |  | Inhibited | -2,173 | 1,44E-05 |
| JUN |  |  | 0,257 | 1,56E-05 |
| BRCA1 |  |  | 0,212 | 1,91E-05 |
| FOS |  |  | 1,145 | 1,97E-05 |
| MED30 |  |  |  | 2,06E-05 |
| TFAM |  |  | -1,134 | 3,04E-05 |
| MEF2D |  | Activated | 3,105 | 4,23E-05 |
| HOXA9 |  |  | 1,000 | 5,70E-05 |
| KLF17 |  |  |  | 8,11E-05 |
| TWIST1 |  | Activated | 2,056 | 8,55E-05 |
| WT1 |  |  | -0,693 | 1,10E-04 |
| WWTR1 |  |  | 1,601 | 1,13E-04 |
| PITX2 |  |  | 1,152 | 1,24E-04 |
| NOTCH1 |  |  | 0,306 | 1,31E-04 |
| HNF4A |  | Inhibited | -2,605 | 1,45E-04 |
| TEAD1 | 1,078 |  | -0,022 | 1,52E-04 |
| LMO4 |  |  | 0,602 | 1,70E-04 |
| TP63 |  | Inhibited | -2,197 | 1,75E-04 |
| MTPN |  | Activated | 3,059 | 1,77E-04 |
| MYOD1 |  |  | 0,010 | 1,77E-04 |
| E2F1 |  |  | 0,446 | 2,08E-04 |
| TEAD4 |  |  |  | 2,42E-04 |
| EBF1 |  |  | 1,612 | 2,44E-04 |
| VHL |  |  | -0,268 | 2,68E-04 |
| MYOCD |  |  | -0,694 | 2,87E-04 |
| PML |  |  | 0,817 | 2,93E-04 |
| SMARCD3 | -0,350 |  | 1,673 | 3,01E-04 |
| HIF1A |  |  | 0,841 | 3,04E-04 |
| IKZF1 |  |  | -1,677 | 5,37E-04 |
| MSGN1 |  |  |  | 6,05E-04 |
| MYCN |  | Inhibited | -2,940 | 7,84E-04 |
| TP73 |  |  | -0,574 | 9,23E-04 |
| RUNX3 |  | Inhibited | -2,062 | 1,16E-03 |
| NFKBIA |  |  | 1,617 | 1,23E-03 |
| KLF11 |  |  | -0,291 | 1,23E-03 |
| NUPR1 |  |  | -0,469 | 1,29E-03 |
| TCL1A |  |  |  | 1,37E-03 |
| BARX2 |  |  | 1,732 | 1,44E-03 |
| TFAP2C |  |  | -1,251 | 1,55E-03 |
| CEBPB |  | Activated | 2,427 | 1,63E-03 |
| TWIST2 |  | Activated | 2,609 | 1,64E-03 |
| TAF4 |  |  | -1,964 | 1,67E-03 |
| SPDEF |  | Inhibited | -2,335 | 1,74E-03 |
| SQSTM1 |  |  | 1,938 | 1,92E-03 |
| SMARCB1 | 0,445 |  | 1,034 | 1,99E-03 |
| FOXO3 | 0,741 |  | -0,632 | 2,14E-03 |
| ING2 |  |  |  | 2,42E-03 |
| PDX1 |  |  | 1,192 | 2,57E-03 |
| FOXO1 | 0,785 |  | -0,150 | 2,81E-03 |
| YY1 |  |  | 1,966 | 2,83E-03 |
| SRA1 |  |  | 1,982 | 3,02E-03 |
| SCX | -1,026 |  | 0,000 | 3,02E-03 |
| HOXA10 |  |  | -0,570 | 3,28E-03 |
| HOXD10 |  |  | -0,333 | 3,57E-03 |
| CTNNB1 |  | Activated | 3,194 | 3,57E-03 |
| ETV5 |  |  | 1,732 | 3,69E-03 |
| ASXL1 |  |  | -0,378 | 4,11E-03 |
| NFIB | 0,819 |  |  | 4,11E-03 |
| KLF4 |  | Activated | 2,567 | 4,13E-03 |
| NEUROG1 |  | Inhibited | -2,333 | 4,41E-03 |
| CCND1 |  |  | 0,218 | 4,45E-03 |
| KLF3 |  |  | -1,512 | 4,64E-03 |
| EGR1 |  | Activated | 3,372 | 4,65E-03 |
| ATF2 |  |  | 1,681 | 5,06E-03 |
| SERTAD2 |  |  | 0,762 | 5,17E-03 |
| SPI1 |  | Activated | 2,569 | 5,22E-03 |
| TBP |  |  |  | 5,41E-03 |
| ETS1 |  | Activated | 2,519 | 5,43E-03 |
| CBX1 |  |  |  | 5,54E-03 |
| CREB1 |  |  | 0,914 | 5,75E-03 |
| MESP1 |  |  | 1,000 | 6,62E-03 |
| ZFPM2 |  |  |  | 6,62E-03 |
| HEXIM1 |  |  |  | 6,70E-03 |
| SREBF1 |  |  | 0,281 | 6,90E-03 |
| ARNT2 |  |  | 0,688 | 6,90E-03 |
| MITF |  |  | 0,107 | 6,94E-03 |
| LBX1 |  |  |  | 7,04E-03 |
| TRIM32 |  |  |  | 7,04E-03 |
| ETS2 |  |  | 1,756 | 7,22E-03 |
| REL |  |  | 1,462 | 7,27E-03 |
| DTX1 |  |  | -0,555 | 7,97E-03 |
| MESP2 |  |  | 1,000 | 8,33E-03 |
| SIRT1 |  |  | -0,553 | 8,60E-03 |
| SIM1 |  |  | 0,688 | 8,82E-03 |
| EGR3 |  |  | 0,243 | 8,84E-03 |
| CEBPA |  |  | 1,838 | 8,86E-03 |
| SNAI2 |  |  | 1,588 | 8,90E-03 |
| NOTCH4 |  |  | -1,400 | 9,39E-03 |
| SMAD2 |  |  | 1,901 | 9,61E-03 |
| MED13 | 0,621 |  |  | 1,01E-02 |
| NFIA | 0,442 |  | 1,131 | 1,03E-02 |
| SMAD4 |  |  | 1,810 | 1,06E-02 |
| STAT3 |  |  | 1,957 | 1,10E-02 |
| PRDM16 | 1,092 |  |  | 1,10E-02 |
| MYB |  | Activated | 2,933 | 1,12E-02 |
| SMARCA4 |  | Activated | 2,548 | 1,13E-02 |
| GLI1 |  |  | 0,860 | 1,18E-02 |
| ATF6 |  |  | 0,055 | 1,18E-02 |
| TBXT |  | Activated | 2,000 | 1,25E-02 |
| YAP1 |  |  | 1,794 | 1,26E-02 |
| NKX2-5 | -1,005 |  | 1,781 | 1,29E-02 |
| TCF7L2 |  |  | 1,598 | 1,42E-02 |
| ELK3 |  |  |  | 1,46E-02 |
| NCOA4 |  |  | -1,342 | 1,47E-02 |
| MECP2 |  |  |  | 1,53E-02 |
| ID1 |  |  | 0,142 | 1,55E-02 |
| CALR |  |  | 1,457 | 1,62E-02 |
| Foxp1 | 0,825 |  | -1,387 | 1,62E-02 |
| HEY2 |  |  | 0,073 | 1,68E-02 |
| ARNT |  |  | 0,056 | 1,73E-02 |
| TFE3 |  |  | 0,152 | 1,79E-02 |
| SRSF2 |  |  | 0,000 | 1,81E-02 |
| TCF4 | 0,638 |  | 1,480 | 1,88E-02 |
| GATA5 |  |  |  | 1,88E-02 |
| CRTC1 |  |  |  | 1,88E-02 |
| KEAP1 | -0,429 |  | -1,131 | 1,91E-02 |
| FBXW7 |  |  |  | 2,01E-02 |
| HDAC1 |  |  | -0,737 | 2,06E-02 |
| SOX2 |  |  | -0,399 | 2,09E-02 |
| NFIX |  |  |  | 2,11E-02 |
| ATF4 |  |  | 1,022 | 2,11E-02 |
| MSC |  |  | -0,128 | 2,14E-02 |
| TRIM29 |  |  |  | 2,20E-02 |
| MYBBP1A |  |  |  | 2,20E-02 |
| RFXANK |  |  |  | 2,20E-02 |
| MED1 |  |  | 0,083 | 2,22E-02 |
| HNRNPK | 0,346 |  | -0,762 | 2,22E-02 |
| TCF3 |  |  | -0,631 | 2,38E-02 |
| NANOG |  |  | -0,072 | 2,42E-02 |
| EPAS1 |  |  | -0,189 | 2,43E-02 |
| FLI1 | 0,779 |  | -1,756 | 2,53E-02 |
| TFCP2 |  |  |  | 2,89E-02 |
| NRIP1 | 0,554 | Activated | 2,611 | 2,98E-02 |
| TFAP2A |  |  | 0,010 | 3,05E-02 |
| XBP1 |  |  | 1,302 | 3,10E-02 |
| HNF1B |  |  | -1,193 | 3,12E-02 |
| IRX4 |  |  |  | 3,19E-02 |
| Msx3 |  |  |  | 3,19E-02 |
| ETV3 |  |  |  | 3,19E-02 |
| SOX15 |  |  |  | 3,19E-02 |
| SF1 |  |  |  | 3,19E-02 |
| RELA |  |  | 0,811 | 3,20E-02 |
| USF2 |  |  | 1,201 | 3,22E-02 |
| HAND1 |  |  | 1,181 | 3,24E-02 |
| CBFB |  |  | 1,890 | 3,32E-02 |
| BCL6 | 0,590 |  | 0,485 | 3,35E-02 |
| SATB1 |  |  | 1,224 | 3,37E-02 |
| ZNF148 |  |  |  | 3,48E-02 |
| HDAC9 |  |  |  | 3,48E-02 |
| HOXC9 |  |  |  | 3,48E-02 |
| PLAGL1 |  |  |  | 3,48E-02 |
| RCOR1 |  |  |  | 3,48E-02 |
| POU2F1 |  |  |  | 3,66E-02 |
| TAF4B |  |  |  | 3,69E-02 |
| FOXA3 |  |  | -0,894 | 3,75E-02 |
| EGR2 |  | Activated | 2,010 | 3,76E-02 |
| FHL2 | -0,644 |  | -0,737 | 3,85E-02 |
| ZBTB20 |  |  | -0,816 | 3,85E-02 |
| SP3 |  |  | -0,140 | 3,89E-02 |
| FOXO4 |  |  | 0,875 | 3,96E-02 |
| KDM3A |  |  |  | 4,32E-02 |
| ZNF746 |  |  |  | 4,32E-02 |
| TSC22D1 | 0,366 |  |  | 4,32E-02 |
| NKX3-2 |  |  |  | 4,32E-02 |
| SOX9 |  |  | 0,798 | 4,32E-02 |
| GATA6 |  |  | 0,768 | 4,54E-02 |
| DNAJB6 |  |  |  | 4,84E-02 |
| NOTCH3 |  |  | 0,926 | 4,88E-02 |
| NRF1 |  |  | -1,000 | 4,88E-02 |
| NKX2-6 |  |  |  | 4,93E-02 |
| ASB9 |  |  |  | 4,93E-02 |
| CEP290 |  |  |  | 4,93E-02 |
| FERD3L |  |  |  | 4,93E-02 |
| MEF2B |  |  |  | 4,93E-02 |
| SLC2A4RG |  |  |  | 4,93E-02 |
| Ikzf1 |  |  |  | 4,93E-02 |
| EID1 |  |  |  | 4,93E-02 |
| ALYREF |  |  |  | 4,93E-02 |
| SMAD1 |  |  | 0,958 | 4,97E-02 |

Transcription factors are ordered by differentially expressed target genes (p-value of overlap). Expr Log Ratio = differential expression of transcription factor itself, Predicted State = activated or inhibited in lean + DS based on Activation z-scores of >2 or <-2, respectively, Activation z-score = quantitative determination of activity based on expected and observed direction of differential target gene expression.

**Supplemental table 8:** Predicted activation of transcription factors in obese + DS versus obese

| **Upstream Regulator** | **Expr Log Ratio** | **Predicted State** | **Activation z-score** | **p-value of overlap** |
| --- | --- | --- | --- | --- |
| TP53 |  |  | 0,985 | 6,62E-33 |
| MYC |  | Inhibited | -2,386 | 7,40E-24 |
| KLF15 |  | Inhibited | -3,698 | 7,73E-17 |
| PPARGC1A |  | Inhibited | -4,659 | 2,36E-16 |
| HTT |  | Activated | 3,329 | 2,61E-16 |
| KDM5A |  | Activated | 4,703 | 2,03E-12 |
| Esrra | -0,599 |  | -0,918 | 7,59E-12 |
| FOS |  |  | -0,439 | 1,63E-10 |
| PPARGC1B |  | Inhibited | -2,023 | 4,57E-10 |
| MEF2A |  |  | 0,134 | 1,11E-09 |
| TP73 |  |  | -0,938 | 2,02E-09 |
| JUN |  |  | 0,018 | 3,75E-09 |
| TWIST1 |  | Activated | 2,632 | 6,43E-09 |
| RB1 |  | Inhibited | -4,185 | 8,39E-09 |
| HOXA9 |  |  | 0,378 | 3,47E-08 |
| SP1 | 0,504 | Activated | 2,995 | 3,55E-08 |
| SOX4 |  | Activated | 2,601 | 3,95E-08 |
| MED30 |  |  |  | 4,41E-08 |
| MYCN |  | Inhibited | -2,419 | 1,12E-07 |
| HDAC5 |  |  | 0,333 | 2,19E-07 |
| MTPN |  | Activated | 3,764 | 4,53E-07 |
| SPDEF |  | Inhibited | -3,035 | 5,73E-07 |
| HOXA10 |  |  | -1,826 | 5,77E-07 |
| MEF2D |  | Activated | 2,392 | 7,72E-07 |
| TFAM |  |  | -1,667 | 1,17E-06 |
| NFKBIA |  | Activated | 3,166 | 1,41E-06 |
| EBF1 |  |  | 1,397 | 1,50E-06 |
| TBX5 | 0,730 | Inhibited | -2,387 | 1,91E-06 |
| PITX2 |  | Inhibited | -2,118 | 1,98E-06 |
| MYOD1 |  |  | 0,633 | 2,09E-06 |
| FOXO3 |  |  | -0,940 | 2,68E-06 |
| HNF4A |  |  | -1,427 | 2,77E-06 |
| SMAD3 | 0,779 | Activated | 2,606 | 3,10E-06 |
| SMARCA4 |  | Activated | 3,294 | 3,25E-06 |
| HIF1A |  |  | 1,373 | 3,61E-06 |
| EP300 |  |  | 0,876 | 4,16E-06 |
| MYOCD |  |  | -1,930 | 4,32E-06 |
| CREB1 |  | Activated | 2,262 | 4,86E-06 |
| CTNNB1 |  | Activated | 3,503 | 6,13E-06 |
| ATF4 |  | Activated | 2,245 | 9,86E-06 |
| WT1 |  |  | -0,098 | 9,88E-06 |
| SMAD7 |  | Inhibited | -3,972 | 1,13E-05 |
| CEBPB |  | Activated | 2,542 | 1,33E-05 |
| FOXO1 |  |  | 1,976 | 1,54E-05 |
| MEF2C |  | Inhibited | -2,319 | 1,59E-05 |
| PML |  |  | 1,868 | 2,20E-05 |
| STAT3 |  | Activated | 2,699 | 2,54E-05 |
| TP63 |  |  | -0,415 | 2,97E-05 |
| HAND2 |  | Inhibited | -2,095 | 3,27E-05 |
| NEUROG1 |  | Inhibited | -2,673 | 3,51E-05 |
| GATA4 |  |  | -1,642 | 4,35E-05 |
| E2F1 |  |  | 0,807 | 6,19E-05 |
| NRIP1 |  | Activated | 2,375 | 7,46E-05 |
| NFATC4 |  |  |  | 7,56E-05 |
| MECP2 |  |  | 0,065 | 1,43E-04 |
| STAT6 |  |  | 1,635 | 1,72E-04 |
| ETV6 | 0,927 | Inhibited | -2,000 | 1,75E-04 |
| TFAP2A |  |  | 0,420 | 1,88E-04 |
| YAP1 |  |  | 1,353 | 1,88E-04 |
| TCF7L2 |  | Activated | 4,163 | 2,07E-04 |
| SREBF1 |  |  | -0,416 | 2,07E-04 |
| SMAD4 |  | Activated | 2,370 | 2,31E-04 |
| HNF1B |  | Inhibited | -2,398 | 3,39E-04 |
| TCL1A |  |  |  | 3,48E-04 |
| SIN3A | 0,544 | Inhibited | -2,236 | 3,63E-04 |
| CREBBP | 0,705 |  | 0,769 | 3,65E-04 |
| EPAS1 |  |  | -0,306 | 3,76E-04 |
| ATF6 |  |  | -0,517 | 4,24E-04 |
| ETS1 |  | Activated | 2,608 | 4,31E-04 |
| GATA5 |  |  | 0,152 | 4,95E-04 |
| GLIS2 |  | Inhibited | -2,449 | 5,41E-04 |
| KLF2 |  |  | -1,677 | 5,43E-04 |
| ELK1 |  |  | 1,108 | 5,54E-04 |
| STAT5B |  |  | -0,740 | 5,55E-04 |
| PAX3 |  |  |  | 5,55E-04 |
| CEBPA |  |  | 1,110 | 5,62E-04 |
| FOXO4 |  |  | 1,329 | 5,66E-04 |
| TEAD1 | 1,504 |  | 0,059 | 6,14E-04 |
| MITF |  |  | 1,568 | 7,83E-04 |
| GLI1 |  |  | 1,710 | 8,03E-04 |
| SMARCB1 |  |  | 1,224 | 9,02E-04 |
| CALR | 0,362 |  | 1,441 | 9,03E-04 |
| NOTCH1 |  |  | -0,039 | 9,70E-04 |
| MYB |  | Activated | 2,636 | 1,00E-03 |
| IKZF1 |  |  | -0,725 | 1,03E-03 |
| SRSF2 | -0,476 |  |  | 1,08E-03 |
| EGR1 |  |  | 1,814 | 1,12E-03 |
| PDX1 |  |  | 1,990 | 1,23E-03 |
| SKIL |  | Activated | 2,360 | 1,28E-03 |
| STAT4 |  |  | 0,194 | 1,53E-03 |
| ATN1 |  |  |  | 1,56E-03 |
| SERTAD2 |  | Activated | 2,219 | 1,61E-03 |
| KLF3 |  |  | -1,859 | 1,75E-03 |
| NKX2-1 |  |  | -0,246 | 1,77E-03 |
| STAT1 |  | Activated | 3,080 | 1,89E-03 |
| HDAC4 |  |  | -1,086 | 1,98E-03 |
| MKL1 |  |  | 0,585 | 2,00E-03 |
| RFXANK |  |  |  | 2,15E-03 |
| HEY2 |  |  | -0,836 | 2,42E-03 |
| NKX2-5 |  |  | 1,701 | 2,75E-03 |
| MAX |  | Inhibited | -2,804 | 2,90E-03 |
| VHL |  |  | 0,566 | 3,25E-03 |
| FOXM1 |  | Activated | 2,616 | 3,52E-03 |
| ASXL1 |  |  | 0,000 | 3,78E-03 |
| BARX2 |  | Activated | 2,000 | 3,92E-03 |
| KLF17 |  |  |  | 4,11E-03 |
| IRX4 |  |  |  | 4,11E-03 |
| YY1 |  |  | 0,517 | 4,22E-03 |
| ARNT2 |  |  | 1,043 | 4,77E-03 |
| KLF4 |  |  | 1,204 | 4,86E-03 |
| TEAD4 |  |  |  | 4,99E-03 |
| NFYB |  |  |  | 5,01E-03 |
| CDKN2A |  |  | -1,951 | 5,27E-03 |
| CCND1 |  |  | 0,228 | 5,51E-03 |
| LEF1 |  |  | 1,494 | 5,69E-03 |
| TCF4 | 0,560 | Activated | 2,398 | 5,69E-03 |
| TWIST2 |  | Activated | 2,609 | 5,87E-03 |
| PLAG1 |  |  | 0,513 | 6,26E-03 |
| CUX1 |  |  |  | 6,26E-03 |
| SPI1 |  | Activated | 2,811 | 6,27E-03 |
| RFX1 |  |  |  | 6,40E-03 |
| GATA6 | -0,584 |  | 0,960 | 6,40E-03 |
| FOXA1 |  | Inhibited | -3,179 | 6,57E-03 |
| ZIC3 |  |  | 0,958 | 6,66E-03 |
| SATB1 |  |  | -0,482 | 6,70E-03 |
| ZNF85 |  |  |  | 6,86E-03 |
| AFF1 |  |  |  | 6,86E-03 |
| RBFOX2 |  |  |  | 6,86E-03 |
| RUNX2 |  |  | 1,687 | 7,24E-03 |
| SQSTM1 |  | Activated | 2,149 | 7,66E-03 |
| SP3 |  |  | 0,724 | 7,92E-03 |
| MSC |  |  | 0,563 | 7,94E-03 |
| MED13 | 0,621 |  | 1,067 | 7,98E-03 |
| SIN3B |  |  |  | 7,98E-03 |
| TCF3 |  |  | -0,086 | 8,81E-03 |
| HOXA11 |  |  | 0,152 | 8,87E-03 |
| NFIX |  |  |  | 9,81E-03 |
| HEY1 |  |  | 0,212 | 9,81E-03 |
| NFATC3 |  |  |  | 9,93E-03 |
| TAF4 |  | Inhibited | -2,804 | 1,02E-02 |
| CBX1 |  |  |  | 1,05E-02 |
| HEYL |  |  |  | 1,05E-02 |
| ZNF91 |  |  |  | 1,05E-02 |
| MYOG |  |  | 0,440 | 1,08E-02 |
| EBF3 |  |  |  | 1,10E-02 |
| LBX1 |  |  |  | 1,10E-02 |
| ERG |  | Activated | 2,714 | 1,13E-02 |
| NFE2L2 |  |  | -0,497 | 1,16E-02 |
| HTATIP2 |  |  |  | 1,19E-02 |
| SMAD1 |  | Activated | 2,429 | 1,21E-02 |
| SIM1 |  |  | 1,279 | 1,23E-02 |
| HMGA1 | -0,941 |  | 0,191 | 1,23E-02 |
| NFATC2 |  |  | 1,659 | 1,23E-02 |
| FHL2 | -0,915 |  | -0,651 | 1,25E-02 |
| BRCA1 |  |  | 0,562 | 1,30E-02 |
| ZBTB16 | -0,384 |  | -1,266 | 1,30E-02 |
| CREM |  |  | -0,788 | 1,39E-02 |
| RAD21 |  |  |  | 1,46E-02 |
| ZFPM2 |  |  |  | 1,46E-02 |
| MKX |  |  |  | 1,50E-02 |
| RFXAP |  |  |  | 1,50E-02 |
| HOXD10 |  |  | -1,000 | 1,50E-02 |
| POU5F1 |  |  | -0,117 | 1,62E-02 |
| HAND1 |  |  | 1,459 | 1,70E-02 |
| HEXIM1 |  |  |  | 1,70E-02 |
| BTG2 |  |  | -1,965 | 1,70E-02 |
| NRF1 |  |  | -0,323 | 1,73E-02 |
| KLF11 |  |  | -0,956 | 1,76E-02 |
| DNAJB6 |  |  | -1,948 | 1,82E-02 |
| ARNT |  |  | -0,842 | 1,87E-02 |
| HNRNPK |  |  | -0,762 | 1,97E-02 |
| ID1 |  |  | 1,198 | 1,99E-02 |
| RCAN1 |  |  | -1,109 | 2,00E-02 |
| DTX1 |  |  | -1,387 | 2,00E-02 |
| TAF4B |  |  |  | 2,00E-02 |
| BCL6B |  |  |  | 2,05E-02 |
| TBR1 |  |  |  | 2,11E-02 |
| TAF12 |  |  |  | 2,11E-02 |
| EPC1 |  |  |  | 2,11E-02 |
| FOXC2 |  |  | 1,211 | 2,12E-02 |
| RUNX3 |  | Inhibited | -2,359 | 2,13E-02 |
| NFATC1 |  | Activated | 2,598 | 2,13E-02 |
| ECSIT | -0,389 |  | 0,436 | 2,33E-02 |
| RELA |  |  | 0,762 | 2,39E-02 |
| CIITA |  |  | 0,417 | 2,49E-02 |
| EGR2 |  |  | 1,083 | 2,63E-02 |
| RFX2 |  |  |  | 2,68E-02 |
| USF1 |  |  | 1,837 | 2,76E-02 |
| FLI1 | 0,726 |  | -1,807 | 2,82E-02 |
| NKX2-3 |  |  | -0,299 | 2,97E-02 |
| TFAP4 |  |  | 1,732 | 3,14E-02 |
| ZNF202 |  |  |  | 3,20E-02 |
| SMARCD3 |  |  | 1,951 | 3,20E-02 |
| HOXD3 |  | Activated | 2,000 | 3,20E-02 |
| NUPR1 | 1,464 |  | -1,718 | 3,28E-02 |
| FOXN4 |  |  |  | 3,38E-02 |
| TRIM29 |  |  |  | 3,38E-02 |
| CREB3L4 |  |  |  | 3,38E-02 |
| TAF9 |  |  |  | 3,38E-02 |
| Foxe3 |  |  |  | 3,38E-02 |
| LMO4 |  |  |  | 3,42E-02 |
| AJUBA |  |  |  | 3,42E-02 |
| WWTR1 |  |  | 1,471 | 3,51E-02 |
| SOX10 |  |  |  | 3,53E-02 |
| PAX7 |  |  | 1,172 | 3,58E-02 |
| CBL | 0,910 |  |  | 3,66E-02 |
| RUNX1 |  |  | 0,211 | 3,68E-02 |
| SNAI1 |  |  | 1,021 | 3,68E-02 |
| XBP1 | -0,534 |  | 0,184 | 3,69E-02 |
| SMAD2 |  |  | 1,628 | 3,95E-02 |
| HOXC8 |  |  | -0,555 | 4,00E-02 |
| BCL6 | 0,621 | Activated | 2,306 | 4,01E-02 |
| ETV5 |  | Activated | 2,236 | 4,02E-02 |
| TRIM24 |  |  | -1,938 | 4,23E-02 |
| HIF3A |  |  |  | 4,24E-02 |
| BHLHA15 |  |  |  | 4,24E-02 |
| CLOCK |  |  |  | 4,31E-02 |
| ATF1 |  |  |  | 4,33E-02 |
| EBF2 |  |  |  | 4,38E-02 |
| CEBPE |  |  |  | 4,39E-02 |
| JUNB |  |  | 0,277 | 4,59E-02 |
| SRF | -0,403 |  | -0,509 | 4,59E-02 |
| PAX5 |  | Inhibited | -2,213 | 4,78E-02 |
| NOTCH3 |  | Activated | 2,383 | 4,79E-02 |
| GATA1 |  |  | 0,777 | 4,84E-02 |
| Msx3 |  |  |  | 4,86E-02 |
| TAF10 |  |  |  | 4,86E-02 |
| SOX15 |  |  |  | 4,86E-02 |
| FUBP1 |  |  |  | 4,86E-02 |
| SF1 |  |  |  | 4,86E-02 |
| Hmgb2 |  |  |  | 4,86E-02 |
| SIRT1 |  |  | -0,809 | 4,94E-02 |

Transcription factors are ordered by differentially expressed target genes (p-value of overlap). Expr Log Ratio = differential expression of transcription factor itself, Predicted State = activated or inhibited in obese + DS based on Activation z-scores of >2 or <-2, respectively, Activation z-score = quantitative determination of activity based on expected and observed direction of differential target gene expression.

**Supplemental table 9:** Original data supplemented with p-values of obesity and DS effects, as well as for synergy

|  |  |  |  |  |  |  |  |  |  |  |  |  | **p-values** | | |
| --- | --- | --- | --- | --- | --- | --- | --- | --- | --- | --- | --- | --- | --- | --- | --- |
|  | **Lean** | | | **Lean+DS** | | | **Obese** | | | **Obese+DS** | | | **Obesity** | **DS** | **Interaction** |
| **ACADM (AU)** | 16.27 | ± | 2.52 | 11.29 | ± | 0.99 | 25.28 | ± | 0.87* | 15.43 | ± | 0.93^†^ | <0.05 | <0.05 | 0.11 |
| **ANGPT1 (AU)** | 0.29 | ± | 0.02 | 0.25 | ± | 0.02 | 0.45 | ± | 0.07 | 0.22 | ± | 0.05^†^ | 0.14 | <0.05 | <0.05 |
| **ANGPT2 (AU)** | 0.13 | ± | 0.01 | 0.10 | ± | 0.01 | 0.14 | ± | 0.03 | 0.12 | ± | 0.01 | 0.28 | 0.20 | 0.73 |
| **ATP1A2 (AU)** | 1.67 | ± | 0.23 | 0.95 | ± | 0.07^‡^ | 0.84 | ± | 0.04* | 0.50 | ± | 0.03^§^ | <0.05 | <0.05 | 0.15 |
| **CAT (AU)** | 1.56 | ± | 0.05 | 1.34 | ± | 0.05 | 2.34 | ± | 0.11 | 1.69 | ± | 0.12 | <0.05 | <0.05 | <0.05 |
| **COL1A1 (AU)** | 0.27 | ± | 0.03 | 0.52 | ± | 0.13 | 0.20 | ± | 0.05 | 0.92 | ± | 0.17^†^ | 0.20 | <0.05 | 0.08 |
| **COL3A1 (AU)** | 0.63 | ± | 0.05 | 1.05 | ± | 0.20 | 0.54 | ± | 0.09 | 1.97 | ± | 0.28^§†^ | 0.05 | <0.05 | <0.05 |
| **DECR1 (AU)** | 4.67 | ± | 0.92 | 2.96 | ± | 0.17 | 9.23 | ± | 1.33* | 4.89 | ± | 0.46^†^ | <0.05 | <0.05 | 0.09 |
| **FN1 (AU)** | 0.13 | ± | 0.02 | 0.25 | ± | 0.05 | 0.16 | ± | 0.01 | 0.56 | ± | 0.06^§†^ | <0.05 | <0.05 | <0.05 |
| **HADHB (AU)** | 8.05 | ± | 0.50 | 7.04 | ± | 0.41 | 13.31 | ± | 1.02* | 9.08 | ± | 0.91^†^ | <0.05 | <0.05 | <0.05 |
| **MYH6 (AU)** | 64.91 | ± | 3.65 | 50.47 | ± | 4.57^‡^ | 31.54 | ± | 1.60* | 19.77 | ± | 2.11^§^ | <0.05 | <0.05 | 0.70 |
| **MYH7 (AU)** | 43.69 | ± | 5.82 | 66.06 | ± | 4.73 | 97.76 | ± | 9.79* | 80.64 | ± | 4.70 | <0.05 | 0.67 | <0.05 |
| **NPPA (AU)** | 12.03 | ± | 2.26 | 41.94 | ± | 13.45 | 9.34 | ± | 1.04 | 69.97 | ± | 13.28^†^ | 0.26 | <0.05 | 0.17 |
| **PLN (AU)** | 57.36 | ± | 8.78 | 35.99 | ± | 3.03^‡^ | 56.24 | ± | 3.66 | 33.98 | ± | 1.36^†^ | 0.75 | <0.05 | 0.93 |
| **POSTN (AU)** | 0.08 | ± | 0.03 | 0.36 | ± | 0.16 | 0.15 | ± | 0.04 | 1.02 | ± | 0.17^§†^ | <0.05 | <0.05 | <0.05 |
| **RCAN1 (AU)** | 3.38 | ± | 0.32 | 2.44 | ± | 0.29 | 5.46 | ± | 0.72* | 3.92 | ± | 0.36 | <0.05 | <0.05 | 0.48 |
| **SERCA2A (AU)** | 99.15 | ± | 9.82 | 75.43 | ± | 4.26^‡^ | 81.26 | ± | 5.71 | 62.04 | ± | 3.24 | <0.05 | <0.05 | 0.71 |
| **SPP1 (AU)** | 0.09 | ± | 0.01 | 0.31 | ± | 0.07 | 0.18 | ± | 0.05 | 0.71 | ± | 0.14^§†^ | <0.05 | <0.05 | 0.10 |
| **TGFβ1 (AU)** | 0.21 | ± | 0.02 | 0.25 | ± | 0.01 | 0.20 | ± | 0.02 | 0.29 | ± | 0.01^†^ | 0.26 | <0.05 | 0.11 |
| **TGFβ2 (AU)** | 0.09 | ± | 0.01 | 0.13 | ± | 0.02 | 0.08 | ± | 0.01 | 0.17 | ± | 0.02^†^ | 0.38 | <0.05 | 0.21 |
| **VEGFA (AU)** | 2.23 | ± | 0.20 | 1.84 | ± | 0.18 | 2.22 | ± | 0.23 | 1.91 | ± | 0.14 | 0.88 | 0.08 | 0.83 |
| **TBARS (μmol/day)** | 0.4 | ± | 0.1 | 0.9 | ± | 0.4 | 36.5 | ± | 3.7* | 6.0 | ± | 2.4^†^ | <0.05 | <0.05 | <0.05 |
| **Casp3 (nr. of positives)** | 0.06 | ± | 0.06 | 0.44 | ± | 0.20 | 0.29 | ± | 0.12 | 0.82 | ± | 0.28 | 0.77 | <0.05 | 0.57 |
| **CD3 (nr. of positives)** | 0.88 | ± | 0.30 | 1.00 | ± | 0.34 | 0.50 | ± | 0.14 | 1.54 | ± | 0.58 | 0.84 | 0.16 | 0.27 |
| **CD68 (nr. of positives)** | 1.60 | ± | 0.51 | 4.96 | ± | 1.25 | 2.42 | ± | 0.94 | 11.54 | ± | 2.42^§†^ | <0.05 | <0.05 | 0.09 |
| **Gomori (μm^2^)** | 536.1 | ± | 27.9 | 602.7 | ± | 49.1 | 715.5 | ± | 69.7 | 830.5 | ± | 44.1^§^ | <0.05 | 0.11 | 0.66 |
| **Lectin (cap. per mm^2^)** | 2365 | ± | 158 | 2337 | ± | 197 | 2291 | ± | 127 | 2090 | ± | 163 | 0.36 | 0.51 | 0.62 |
| **Sirius Red (% positivity)** | 3.15 | ± | 0.22 | 6.28 | ± | 0.57 | 4.01 | ± | 0.52 | 9.21 | ± | 1.56^†^ | 0.07 | <0.05 | 0.31 |
| **TUNEL (% positivity)** | 0.14 | ± | 0.02 | 0.45 | ± | 0.09 | 0.22 | ± | 0.09 | 0.43 | ± | 0.08 | 0.18 | 0.05 | 0.73 |

Values are means ± SEM. AU = Arbitrary Units (expression relative to housekeeping genes). * obese vs. lean, ^§^ obese+DS vs. lean+DS, ^‡^ lean+DS vs. lean, ^†^ obese+DS vs. obese P<0.05.

**Supplemental table 10:** Predicted activation of growth factors in lean + DS versus lean

| **Upstream Regulator** | **Expr Log Ratio** | **Predicted State** | **Activation z-score** | **p-value of overlap** |
| --- | --- | --- | --- | --- |
| AGT |  | Activated | 2,492 | 1,88E-11 |
| TGFB1 |  | Activated | 4,009 | 2,81E-11 |
| LEP |  |  | 1,900 | 1,03E-07 |
| IGF1 |  |  | -0,303 | 4,17E-06 |
| VEGFA |  |  | 0,385 | 7,07E-06 |
| TGFB3 |  |  | 1,566 | 7,83E-06 |
| BMP7 |  |  | 1,149 | 1,03E-05 |
| FGF2 |  |  | 1,677 | 1,84E-05 |
| ANGPT2 |  |  | 1,651 | 2,67E-05 |
| HGF |  | Activated | 3,054 | 3,39E-05 |
| WISP2 |  | Inhibited | -2,550 | 3,70E-05 |
| CTGF | 0,842 | Activated | 2,048 | 5,42E-05 |
| NRG1 |  | Activated | 2,499 | 1,29E-04 |
| BMP2 |  | Activated | 2,956 | 1,64E-04 |
| BMP10 |  |  | 0,447 | 1,70E-04 |
| MSTN |  |  | 1,134 | 1,93E-04 |
| PDGFB |  |  | 0,674 | 1,99E-04 |
| TGFB2 | 0,705 |  | 1,745 | 2,14E-04 |
| EGF |  |  | 1,937 | 2,34E-04 |
| VEGFB |  |  | -1,688 | 5,18E-04 |
| FGF1 |  |  | 0,231 | 1,26E-03 |
| GHRL |  |  | 0,102 | 1,38E-03 |
| IGF2 |  |  | -0,216 | 3,94E-03 |
| GDF2 |  |  | 0,106 | 4,27E-03 |
| TDGF1 |  |  |  | 5,54E-03 |
| BMP6 | 0,613 |  | 1,644 | 6,47E-03 |
| INHBB |  |  | 1,987 | 6,62E-03 |
| FGF21 |  |  | -1,563 | 6,88E-03 |
| HDGF |  |  |  | 7,04E-03 |
| GDF9 |  |  | -0,200 | 8,84E-03 |
| FGF10 |  |  | 1,698 | 1,47E-02 |
| GDF15 |  |  |  | 1,51E-02 |
| NOV |  |  | -1,109 | 2,11E-02 |
| GDF11 |  |  |  | 2,20E-02 |
| DKK1 |  |  | -1,998 | 2,75E-02 |
| NRG2 |  |  |  | 4,03E-02 |
| MST1 |  |  | -1,000 | 4,17E-02 |
| ANGPT1 |  |  |  | 4,54E-02 |
| GDF10 |  |  |  | 4,93E-02 |

Growth factors are ordered by differentially expression of downstream target genes (p-value of overlap). Expr Log Ratio = differential expression of growth factor itself, Predicted State = activated or inhibited in lean + DS based on Activation z-scores of >2 or <-2, respectively, Activation z-score = quantitative determination of growth factor signaling based on expected and observed direction of differentially expressed downstream target genes.

**Supplemental table 11:** Predicted activation of growth factors in obese + DS versus obese

| **Upstream Regulator** | **Expr Log Ratio** | **Predicted State** | **Activation z-score** | **p-value of overlap** |
| --- | --- | --- | --- | --- |
| TGFB1 |  | Activated | 6,242 | 2,95E-31 |
| VEGFA | -0,537 |  | 1,478 | 7,48E-14 |
| AGT |  | Activated | 3,279 | 1,36E-10 |
| IGF1 | 0,650 |  | 0,723 | 1,83E-10 |
| NRG1 |  | Activated | 3,025 | 4,68E-09 |
| ANGPT2 |  | Activated | 2,200 | 7,48E-09 |
| TGFB3 | 0,556 | Activated | 2,121 | 2,56E-07 |
| LEP |  |  | 1,005 | 2,77E-07 |
| TGFB2 | 0,833 | Activated | 2,505 | 1,82E-06 |
| BDNF |  |  | 0,157 | 2,09E-06 |
| FGF1 |  |  | -0,931 | 2,29E-06 |
| HGF |  | Activated | 2,798 | 2,71E-06 |
| EGF |  | Activated | 3,003 | 1,15E-05 |
| IGF2 |  |  | 0,696 | 1,41E-05 |
| KITLG |  | Activated | 2,377 | 1,77E-05 |
| PDGFB |  |  | 1,420 | 1,99E-05 |
| BMP7 |  |  | 0,012 | 7,39E-05 |
| BMP2 |  | Activated | 2,265 | 1,33E-04 |
| BMP6 |  |  | 0,568 | 6,35E-04 |
| INHA |  | Inhibited | -2,867 | 7,17E-04 |
| FGF2 |  | Activated | 2,281 | 9,27E-04 |
| MSTN |  |  | 0,923 | 1,16E-03 |
| WISP2 | 0,998 | Inhibited | -2,530 | 1,16E-03 |
| PDGFC |  |  | 1,803 | 1,35E-03 |
| GDF11 |  |  |  | 2,15E-03 |
| CTGF |  | Activated | 3,106 | 5,28E-03 |
| FGF21 |  | Inhibited | -2,785 | 6,71E-03 |
| GHRL |  |  | 0,240 | 7,09E-03 |
| GDF2 |  |  | 1,630 | 8,62E-03 |
| ANGPT1 |  |  |  | 1,06E-02 |
| INHBB |  |  | 1,980 | 1,46E-02 |
| FGF19 |  |  |  | 1,55E-02 |
| HBEGF |  |  | 0,277 | 1,56E-02 |
| FGF10 |  |  | 1,000 | 1,56E-02 |
| GRP |  |  | 1,062 | 1,70E-02 |
| VEGFC |  |  |  | 2,68E-02 |
| GDF15 |  |  |  | 3,20E-02 |
| NRG4 |  |  |  | 3,42E-02 |
| VEGFB |  |  | -1,732 | 3,53E-02 |
| AREG |  |  | 1,364 | 4,52E-02 |
| PDGFD |  |  |  | 4,86E-02 |
| FGF18 |  |  |  | 4,86E-02 |

Growth factors are ordered by differentially expression of downstream target genes (p-value of overlap). Expr Log Ratio = differential expression of growth factor itself, Predicted State = activated or inhibited in obese + DS based on Activation z-scores of >2 or <-2, respectively, Activation z-score = quantitative determination of growth factor signaling based on expected and observed direction of differentially expressed downstream target genes.
